# From Boron to Bismuth: The Pre-transmetalation Complex in the Aryl Transfer

**DOI:** 10.1021/jacs.5c21293

**Published:** 2026-03-02

**Authors:** Teresa Faber, Markus Leutzsch, Nils Nöthling, Josep Cornella

**Affiliations:** 28314 Max-Planck-Institut für Kohlenforschung, Kaiser-Wilhelm-Platz 1, Mülheim an der Ruhr 45470, Germany

## Abstract

B-to-Bi transmetalation is increasingly encountered as a key organometallic step in Bi-based protocols. Yet, akin to transition metal complexes, obtaining structural evidence of pre-transmetalation intermediates that lead to the aryl transfer from B to a metal is challenging and rare. Herein, we provide structural characterization of two unprecedented pre-transmetalation complexes linking B and Bi through a F– or an OH– bridge. Inspired by earlier studies on d-block elements, we establish two general pathways by which transmetalation can occur: the *neutral* and the *ionic pathway*. These studies delineate the requirements for successful transmetalation of a wide range of unprecedented arylboron species.

## Graphical abstract

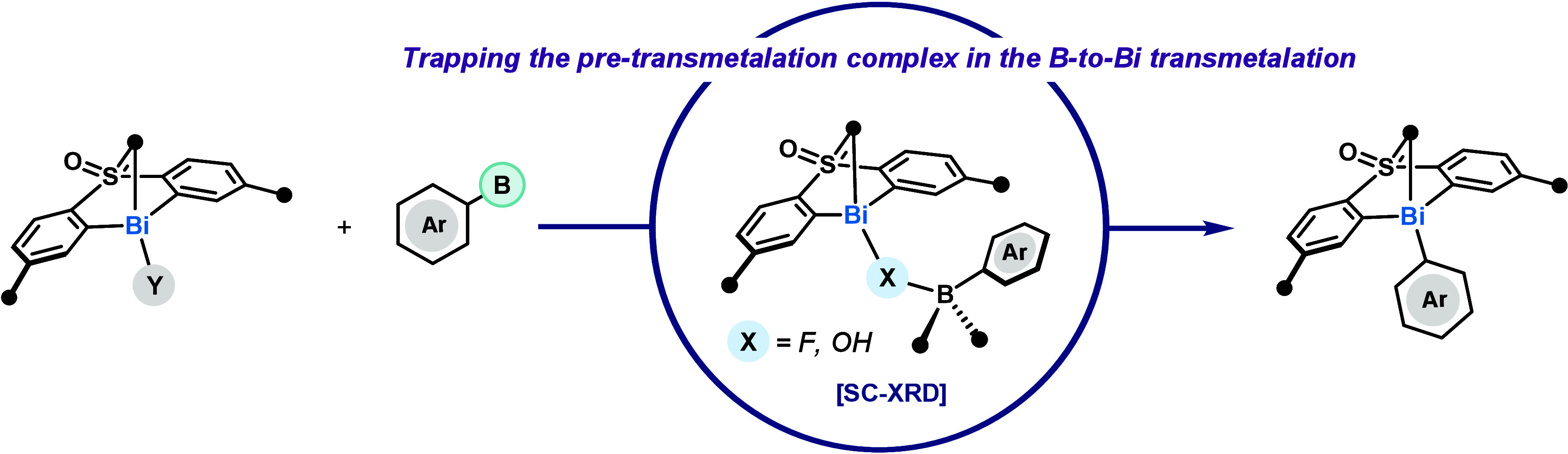

Arylboron reagents (Ar-B) are among the most recognizable and useful synthons in organic synthesis.^1^ Their use in catalysis is unavoidably linked to one of the most fundamental organometallic steps known: the transmetalation reaction.^2^ This involves the transfer of the Ar substituent from boron to the metal center, thus priming it for reactivity. Indeed, this reaction is of capital importance in transition metal catalysis, operating in processes such as Suzuki–Miyaura,^3^ Chan–Lam,^4^ or conjugate additions.^5^ Owing to its synthetic significance, great attention has been paid to understanding the mechanistic intricacies that govern the aryl transfer between arylboron species and transition metals. As a result of many investigations, a general picture has been defined where two main pathways can be operative: the *neutral* and the *ionic pathway* (Figure 1A).^6^ In the former, the external base (X^–^) interacts with the metal center to form a M–X bond and subsequently activates the Ar–B (Figure 1A, left).^7^ In the latter, the external base forms an organoboronate anion and then interacts with an electrophilic metal center (Figure 1A, right).^8^ Regardless of the pathway, both mechanisms lead to a B–X–M species, commonly known as the pre-transmetalation intermediate. From here, aryl transfer occurs to forge the M–Ar bond. Providing evidence of the existence of such pre-transmetalation species has been a major challenge for organometallic chemists, yet examples where oxygen serves as the bridging ligand X have been reported (Figure 1B).^9,10^ Fluoride anions have also been used in facilitating transmetalation with arylboron reagents,^11^ as exemplified by ArBF_3_^–^ salts^12–14^ and fluoride-promoted Suzuki cross-couplings.^15^ These studies suggest the involvement of analogous B–F–M intermediates,^11c,13b,13c^ although crystallographic confirmation of such species—either hydroxy- or fluorido-bridged—remains elusive. Beyond transition metals, main group elements capable of transmetalation with organoboron compounds are sparse,^16,17^ and catalytic variants and studies on putative pre-transmetalation intermediates are rare.^16j,18^

**Figure 1:**
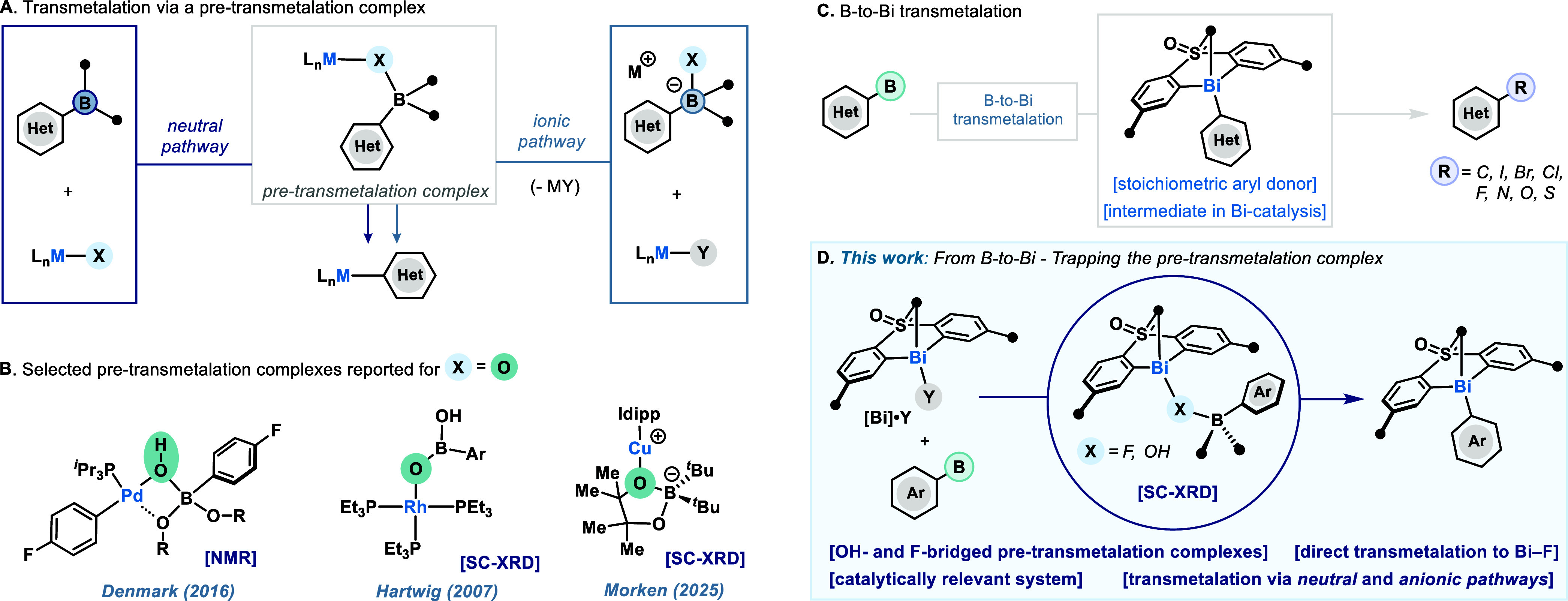
Transmetalation of arylboron to transition metals and to bismuth (X = bridging anion/ligand; Y = generic anion).

In recent years, our group has been interested in deploying the potential of bismuth redox catalysis as a versatile platform for synthesis.^19–24^ Among the Bi­(III) architectures explored as catalysts, complexes bearing bis-aryl sulfone or sulfoximine motifs (e.g. compound **[Bi]·Y**, Figure 1D)^25^ have been proven effective in enabling key transformations.^21–24^ Along this line, we demonstrated that arylboron nucleophiles can be converted to the corresponding C–F^22^ and C–O^23^ via a Bi­(III)/Bi­(V) redox platform, involving neutral tris-aryl bismine intermediates derived from **[Bi]·Y**. Additionally, analogous intermediates could facilitate the insertion of SO_2_ in the synthesis of sulfonyl fluorides from arylboronic acids.^24^ Complementary work by Ball and others has highlighted the use of arylboronic acids in arylation reactions mediated by similar Bi­(III)-aryl complexes.^17a,26,27^ Central to all of these approaches is the transmetalation step between arylboron species and **[Bi]·Y** (Figure 1C), yet information about the process is scarce. Given the growing number of applications for arylboron reagents in bismuth chemistry and catalysis, a study about the existence of a putative pre-transmetalation intermediate would be highly informative for further designs. Along this line, and inspired by Denmark’s pioneering work,^9a–9d^ Ball rightfully proposed the possibility of an oxo- or hydroxy-bridged pre-transmetalation species,^27a^ although evidence of its existence was elusive. In this study, we report two distinct pre-transmetalation intermediates between boron and bismuth: a hydroxy- (B–OH–Bi) and a fluoride-bridged (B–F–Bi) species, both characterized by single-crystal X-ray diffraction (SC-XRD) and NMR spectroscopy (Figure 1D). Based on these studies, we establish boundary conditions for successful transmetalation with various arylboron species.

The dimer **[Bi-1]**_**2**_ **·O** and/or its hydrated monomer **[Bi-1]·OH** is known to readily react with 4-fluorophenylboronic acid (**1**) to form **[Bi-1]·Ar** ^**4‑F**^ (Figure 2, *path a*).^27a^ This transformation was proposed to proceed via an oxo- or hydroxy-bridged intermediate such as **Int-1**. Drawing inspiration from the literature on transition metals,^9,10^ we hypothesized that the same **Int-1** could be accessed through a complementary *ionic pathway*. Indeed, when **[Bi-2]·Y** (Y = Cl, BF_4_, or OTf) was combined with **2**, **[Bi-2]·Ph** was obtained in high yields (84–88%) (Figure 2, *path b*). These results indicate that both *neutral* and *ionic pathways* are feasible with Bi­(III) when using OH^–^ as the activator. Nonetheless, Suzuki–Miyaura cross-couplings can also proceed using fluoride anions as base, thus presenting a complementary activation approach.^15^ In these cases, an analogous fluoride-bridged pre-transmetalation intermediate, akin to **Int-2**, is postulated.^11c,13b,13c^ In a similar manner, both an *ionic pathway* and a *neutral pathway* could potentially operate. To probe the former pathway, we synthesized the corresponding monomeric **[Bi-2]·F**. When it was combined with Ph-BF_2_ (**3**) at 25 °C, **[Bi-2]·Ph** was obtained in quantitative yield within 10 min (Figure 2, *path c*). Meanwhile, **[Bi-2]·OTf** did not react via the *neutral pathway*. On the other hand, **[Bi-2]·Y**, bearing BF_4_^–^ or OTf^–^ anions, reacted with [Ph-BF_3_]­[NBu_4_] (**4a**), thus affording **[Bi-2]·Ph** in 62% and 60% yield, respectively (Figure 2, *path d*).

**Figure 2:**
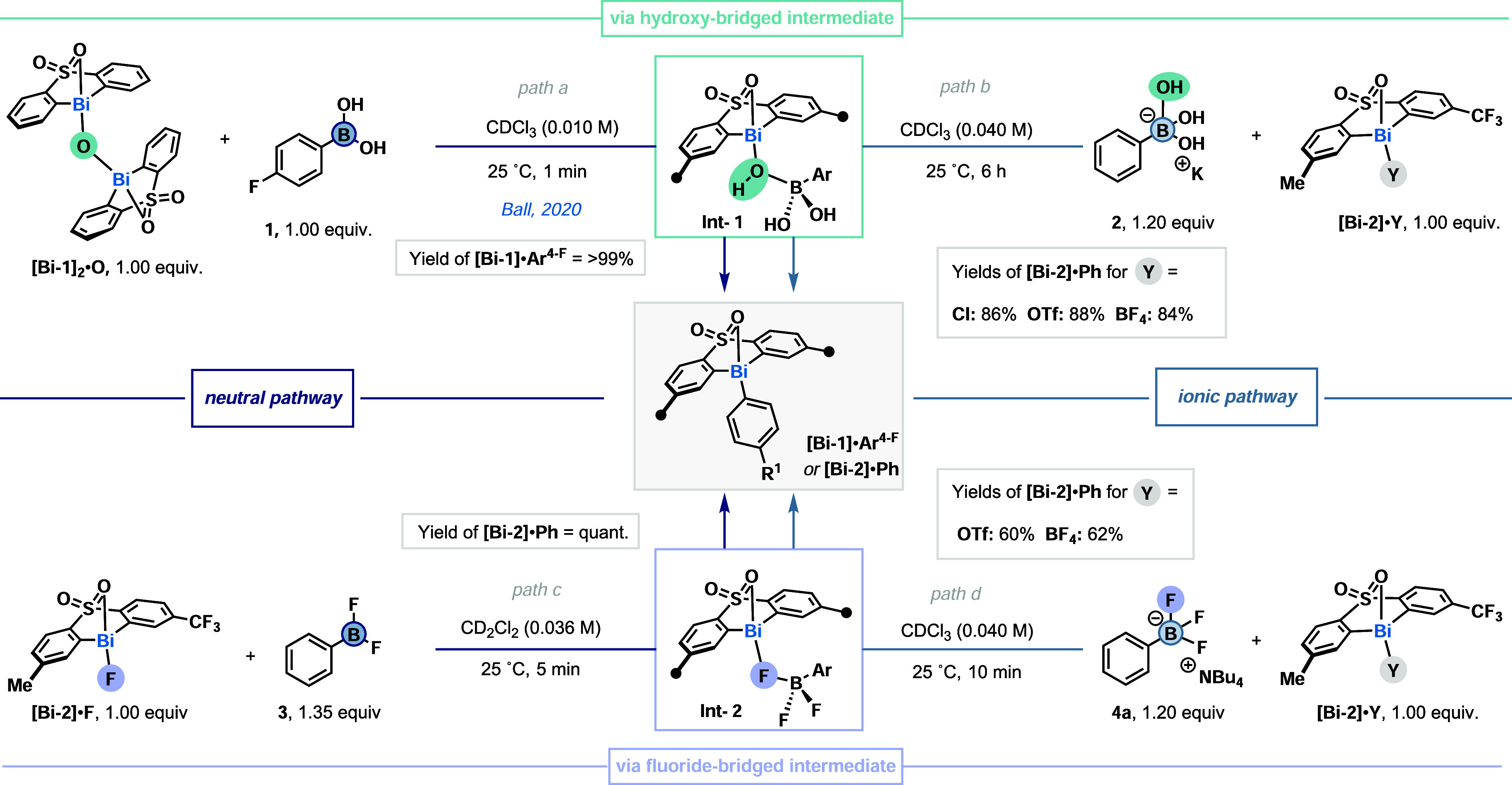
Transmetalation of arylboron species to bis-aryl Bi­(III) complexes. Yields were determined by quantitative ^1^H NMR.

These results provide evidence for a *fluoride pathway* in Bi­(III) and offer a solid head-to-head comparison with precedents involving transition metals. By analogy to transition metals, we hypothesized that, if existing, putative pre-transmetalation intermediates would resemble **Int-1** and **Int-2**. As expected, all attempts to isolate these intermediates from the reactions depicted in Figure 2 proved extremely challenging. Focusing on the *neutral fluoride pathway*, we identified two key requirements for successful observation of the pre-transmetalation intermediate: 1) the formation of the adduct between **[Bi-2]·F** and the arylboron species should be exothermic; 2) the kinetic energy barrier should be sufficiently high to prevent fast transmetalation. To this end, we identified tris­(pentafluoro­phenyl)­borane (BCF, **5**) to serve as a suitable arylboron source.^28^ Indeed, when **[Bi-2]·F** was combined with **5** in CD_2_Cl_2_ at 25 °C, complete consumption of **[Bi-2]·F** in favor of a new species was observed (Figure 3A). NMR analysis at −40 °C pointed to the formation of **[Bi-2]·B­(Ar** ^**F**^ **)**_**3**_ **F** as a close contact ion pair in solution (see SI, section 3.1.1). Stirring the mixture at 25 °C for 24 h led to an aryl transfer, and **[Bi-2]·Ar** ^**F**^ was obtained in 96% yield (84% isolated yield).

**Figure 3:**
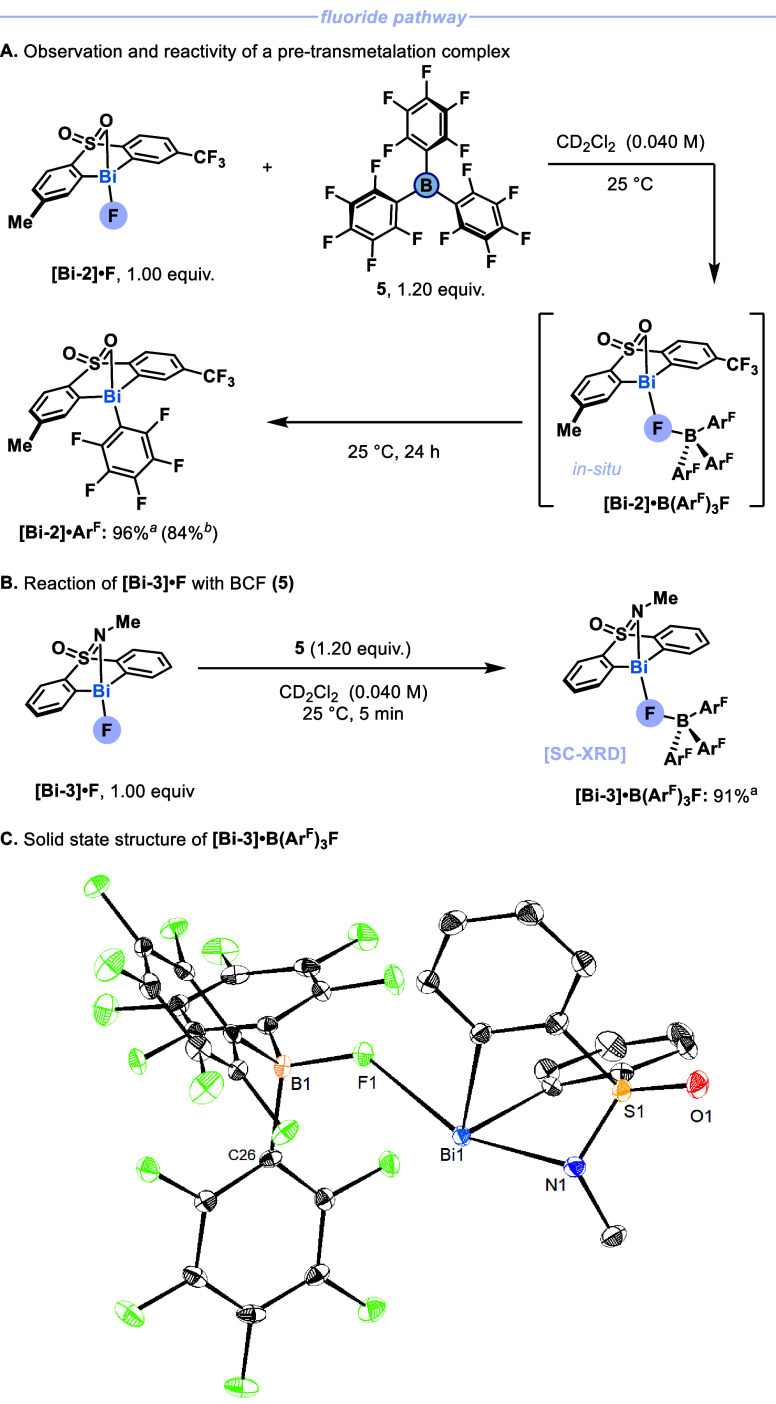
Isolation and reactivity of fluoride-bridged pre-transmetalation complex. ^*a*^Yields were determined by quantitative ^1^H NMR. ^*b*^Isolated yield.

Given the facile transmetalation of **[Bi-2]·B­(Ar** ^**F**^ **)**_**3**_ **F** at ambient conditions, we anticipated that further stabilization would be required to obtain structural information through SC-XRD. In our previous work, solid state structures of related Bi­(III) compounds with a sulfoximine moiety in the ligand backbone showed a preference in hypervalent coordination for N over O,^22a^ probably due to the more basic L-type ligand. We speculated that if the cationic character of Bi was reduced through hypervalent N-coordination, then aryl transfer would be slower. When **[Bi-3]·F** was combined with **5**, formation of **[Bi-3]·B­(Ar** ^**F**^ **)**_**3**_ **F** was observed by NMR (Figure 3B). The compound was stable enough to obtain crystals suitable for SC-XRD analysis by vapor diffusion of *n*-hexane into a saturated solution of chlorobenzene at −20 °C after 21 d (Figure 3C). The asymmetric unit was found to contain two crystallographically independent molecules.^29^ Compared to related **[Bi-4]·F** (2.1352(15) Å, see Figure 4A), the Bi–F bond is significantly elongated (2.4688(14) Å); meanwhile, the B–F bond (1.477(3) Å) is only marginally longer than the average bond lengths for [F–B­(Ar^F^)_3_]^−^ (1.458(48) Å; see SI, section 8.3).^30^ This indicates that the F has largely been abstracted from bismuth in favor of a B–F bond. The resulting buildup of positive charge at the Bi center is reflected by the Bi–N bond distance in **[Bi-3]·B­(Ar** ^**F**^ **)**_**3**_ **F** (2.341(2) Å), which is much shorter than that in neutral **[Bi-3]·Ph** (3.0551(16) Å).^22a^

**Figure 4:**
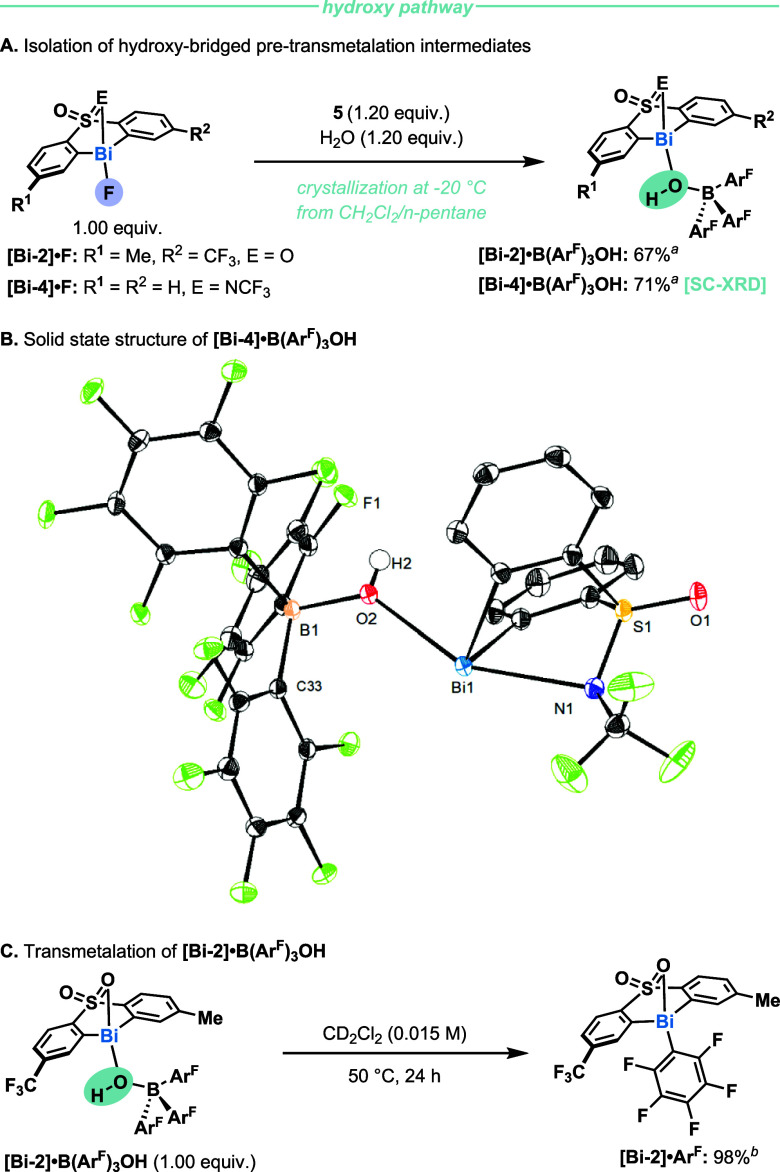
Isolation and reactivity of hydroxide-bridged pre-transmetalation intermediates. ^*a*^Isolated yield. ^*b*^Yields were determined by quantitative ^1^H NMR.

Following this strategy, we anticipated that a hydroxide-bridged intermediate could also be accessible. Therefore, we mixed *in situ*-generated **5·H**_**2**_ **O** with **[Bi-2]·F** in CH_2_Cl_2_ at 25 °C. A new species corresponding to **[Bi-2]·B­(Ar** ^**F**^ **)**_**3**_ **OH** was isolated in 67% yield (Figure 4A). When the O in the sulfone backbone was replaced by an NCF_3_ group, **[Bi-4]·B­(Ar** ^**F**^ **)**_**3**_ **OH** was obtained in 71% yield. The complexes showed signals at 4.55 and 4.26 ppm in ^1^H NMR, respectively, indicative of the presence of a hydroxy group. ^1^H–^1^H NOESY correlations between the hydroxy group and hydrogens on the sulfone/sulfoximine backbone, as well as ^1^H–^19^F HOESY correlation between the pentafluorophenyl group and the ligand backbone, reinforced the formation of a closed ion pair (see SI, section 4.1). Additionally, we could verify the structure of **[Bi-4]·B­(Ar** ^**F**^ **)**_**3**_ **OH** by SC-XRD (Figure 4B). The crystal structure revealed a bridging hydroxide connecting boron and bismuth. The Bi1–O2 distance is 2.3305(10) Å, and the Bi1–O2–B1 bond angle is 133.05(8)°. This brings one of the aryl groups bound to B in close proximity to the bismuth center (C33–Bi1 distance = 3.5544(13) Å). The Bi1–N1 distance (2.5316(12) Å) is longer than that for **[Bi-4]·BF**_**4**_ (2.4474(11) Å)^22a^ but significantly shorter than that for neutral **[Bi-4]·Ph** (3.039(3) Å),^22a^ indicative that Bi in **[Bi-4]·B­(Ar** ^**F**^ **)**_**3**_ **OH** contains significant cationic character.

Having isolated two hydroxyl-bridged complexes, we studied the transmetalation. When **[Bi-2]·B­(Ar** ^**F**^ **)**_**3**_ **OH** was heated to 50 °C for 24 h, **[Bi-2]·** **Ar** ^**F**^ was obtained in 98% yield (Figure 4C). Meanwhile, **[Bi-4]·B­(Ar** ^**F**^ **)**_**3**_ **OH** afforded a mixture of complexes due to the instability of the ligand backbone (see SI, section 4.2).

Based on these studies, we anticipated that the transmetalation through the *neutral pathway* should occur from **[Bi-2]·F** and various phenyl boranes. Indeed, when we combined **[Bi-2]·F** with triphenyl boroxine (**6**), **[Bi-2]·Ph** was obtained in 98% yield. Phenyl catechol boronic ester **7** reacted with **[Bi-2]·F** at 25 °C to give a 61% yield of product. For the transmetalation of neopentyl boronic ester **8** and pinacol boronic ester **9**, heating to 70 and 90 °C was required to obtain **[Bi-2]·Ph** in 71% and 58% yield, respectively (Figure 5A). As the *ionic pathway* should be more general in terms of the counterion on Bi­(III), combining various **[Bi-2]·Y** (Y = BF_4_, OTf, I, or SO_2_Ph) with cyclic triol boronate **10** afforded **[Bi-2]·Ph** in 48–58% yield. High yields were also obtained from Li­[(^*t*^Bu)­(Ph)­Bpin] (**11**) and various **[Bi-2]·Y** (Y = Cl, OTf, I, or SO_2_Ph) (84–93%). When K­[B­(Ph)_4_] (**12**) was combined with **[Bi-2]·Y**, where Y = OTf or BF_4_, **[Bi-2]·Ph** was obtained in 79% or 99% yield, respectively (Figure 5B). Hereby, we anticipate that the transmetalation is mediated by a bismuth–arene π interaction.^18^

**Figure 5:**
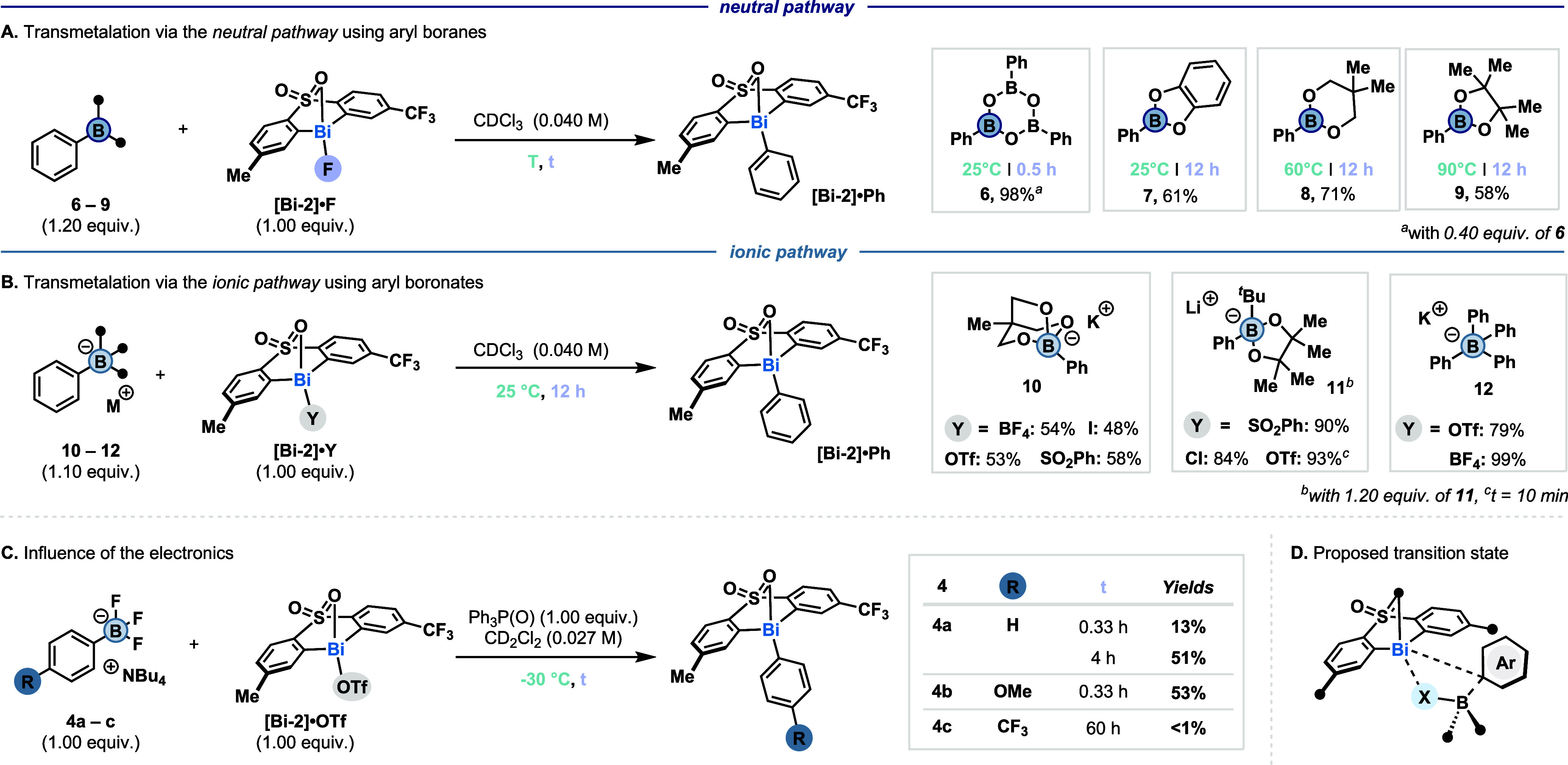
Transmetalation via the *neutral* and the *ionic pathways*. Yields were determined by quantitative ^1^H NMR.

To investigate the effect of the electronic nature of the arylboronate on the transmetalation, we prepared distinct [Ar-BF_3_]­[NBu_4_] (**4a**–**4c**). However, we found that unsubstituted **4a** reacts fast even at −60 °C (initial product formation 16%; see SI, section 6.1.1). To decelerate the reaction kinetics, Ph_3_P­(O) was added to the reaction mixture, which we anticipated would reversibly bind to **[Bi-2]** ^**+**^.^31^ Indeed, its addition slowed down the reaction, and at −30 °C a well-defined kinetic profile was obtained (see SI, section 6.1.2). Initially, 4% of **[Bi-2]·Ph** was obtained, reaching 51% yield after 4 h. When electron-rich **4b** bearing *p*-OMe was subjected to the same reaction conditions, the reaction afforded a 53% yield of **[Bi-2]·Ar** ^**OMe**^, albeit already after 0.33 h. Meanwhile, electron-poor **4c** bearing *p*-CF_3_ did not react under the same conditions within 60 h (Figure 5C). These results manifest that electron-rich substrates react faster than electron-poor substrates. The same trend was also observed for the *neutral pathway* when variants of **7** were combined with **[Bi-2]·F** (see SI, section 6.2). Considering these findings in combination with DFT studies (see SI, section 7) of the transition state, we speculate that the transmetalation occurs via an electrophilic aromatic substitution at the *ipso*-C (Figure 5D).^32^ This is also in line with the faster transmetalation observed when Bi­(III) has more δ^+^ character (*vide supra*).

Overall, this study provides mechanistic insights into the transmetalation of arylboron nucleophiles to Bi­(III). Importantly, we isolated and characterized two pre-transmetalation intermediates featuring a F– or an OH– bridge between B and Bi, which are unique within the realm of main group catalysis. B-to-Bi transmetalation can occur either via a *neutral pathway* from **[Bi]·X** (X = OH, F) and neutral boranes or via an *ionic pathway* from cationic **[Bi]·Y** (Y = OTf, BF_4_, Cl, I, SO_2_Ph) and a pre-formed ionic boronate. Overall, we believe this study will guide the design of novel bismuth-based strategies as well as shed light onto other main group elements where transmetalation is feasible.

## Supplementary Material



## Notes

Additional experimental details, materials, methods, crystallographic details, theoretical calculation, and characterization data (^1^H, ^13^C, ^19^F and ^11^B NMR and HRMS) for all new compounds (PDF)
