# Supplementary material for: From Boron to Bismuth: The Pre-transmetalation Complex in the Aryl Transfer
Source: J Am Chem Soc. 2026 Mar 2;148(9):9205–12. doi: 10.1021/jacs.5c21293 (PMC12983325; doi:10.1021/jacs.5c21293)
Supplement: Supplementary file 1 [file ja5c21293_si_001.pdf]

Supporting Information

From Boron to Bismuth: The Pre-transmetalation Complex in the Aryl Transfer

Teresa Faber, Markus Leutzsch, Nils Nöthling, Josep Cornella*

Max-Planck-Institut für Kohlenforschung, Kaiser-Wilhelm-Platz 1,
Mülheim an der Ruhr, 45470, Germany.

cornella@kofo.mpg.de

1 General methods 1

2 Synthesis of new Bi(III) complexes 3

2.1 Synthesis of [Bi]•F 3

2.1.1 Synthesis of **[Bi-2]•F** 3

2.1.2 Synthesis of **[Bi-3]•F** 4

2.1.3 Synthesis of **[Bi-4]•F** 5

2.2 Synthesis of [Bi-2]•Y 5

2.2.1 Synthesis of **[Bi-2]•SO_2Ph, [Bi-2]•Cl** and **[Bi-2]•BF_4** 5

2.2.2 Synthesis of **[Bi-2]•OTf** 6

2.2.3 Synthesis of **[Bi-2]•I** 7

3 μ-Fluorido intermediates 8

3.1 Synthesis and Characterization 8

3.1.1 **[Bi-2]•B(Ar^F)$_3$F** 8

3.1.2 **[Bi-3]•B(Ar^F)$_3$F** 10

3.2 Transmetalation of [Bi-2]•B(Ar^F)$_3$F 13

3.2.1 Quantification of the transmetalation 13

3.2.2 Isolation of **[Bi-2]•Ar^F** 14

3.2.3 Kinetic monitoring 15

4 μ-Hydroxido intermediates 19

4.1 Synthesis and Characterization 19

4.1.1 **[Bi-2]•B(Ar^F)$_3$OH** 19

4.1.2 **[Bi-4]•B(Ar^F)$_3$OH** 21

4.2 Transmetalation of the hydroxide intermediates 23

4.2.1 Transmetalation from **[Bi-2]•B(Ar^F)$_3$OH** 23

4.2.2 Transmetalation from **[Bi-4]•B(Ar^F)$_3$OH** 24

5 Transmetalation of boron nucleophiles to Bi 27

5.1 Quantification 27

5.2 Neutral pathway 28

5.2.1 Transmetalation with **3** 28

5.2.2 Transmetalation with **6** 29

5.2.3 Transmetalation with **7** 30

5.2.4 Transmetalation with **8** 31

5.2.5 Transmetalation with **9** 32

5.3 Ionic pathway ... 33
5.3.1 Transmetalation with **2** ... 33
5.3.2 Transmetalation with **4a** ... 33
5.3.3 Transmetalation with **10** ... 34
5.3.4 Transmetalation with **11** ... 34
5.3.5 Transmetalation with **12** ... 35
6 Effect of substitution on aryl boron nucleophiles ... 36
6.1 Kinetic monitoring of the transmetalation between 4 and [Bi-2]•OTf ... 36
6.1.1 Without additives ... 36
6.1.2 With triphenylphosphine oxide ... 40
6.2 Kinetic monitoring of the reaction between 7 and [Bi-2]•F ... 42
7 Computational details ... 43
7.1 General procedure ... 43
7.2 Reaction with difluorophenyl borane ... 44
7.3 Reaction with tris(pentafluoro)borane ... 45
7.4 Reaction with phenylboronic acid ... 47
7.5 Cartesian coordinates ... 48
8 X-Ray ... 63
8.1 Single crystal structure of [Bi-3]•B(Ar^F)$_3$F ... 63
8.2 Single crystal structure of [Bi-4]•B(Ar^F)$_3$OH ... 72
8.3 Database survey and structural discussions ... 80
9 References ... 86
10 NMR spectra ... 88

1 General methods

Instruments

^{1}H, ^{11}B, ^{13}C and ^{19}F spectra were recorded with Bruker Avance III HD 300, Bruker Avance III HD 400 MHz, Bruker Avance III 500 MHz, or Bruker Avance NEO 600 MHz NMR spectrometers. ^{1}H and ^{13}C chemical shifts are reported in ppm relative to the solvent residual peaks as an internal reference. ^{1}H NMR chemical shifts are given in ppm with respect to the residual solvent ($CDCl_3$, δ 7.260 ppm; CD_2Cl_2; δ 5.320 ppm; CD_3CN: δ 1.940 ppm). ^{13}C NMR chemical shifts are given in ppm with respect to the residual solvent ($CDCl_3$, δ 77.16 ppm; CD_2Cl_2; δ 54.00 ppm; CD_3CN: δ 118.26 ppm). ^{13}C spectra were acquired with broadband ^{1}H decoupling. ^{19}F NMR shifts were referenced indirectly to $CFCl_3$. ^{11}B NMR was referenced relative to BF3•OEt_2. Multiplicities are described as s = singlet, br s = broad singlet, d = doublet, t = triplet, q = quartet, dd = doublet of doublets, td = triplet of doublets, m = multiplet. Coupling constants are reported as J-values in Hz.

High-resolution mass spectra were obtained using Bruker APEX III FT-MS (ESI ionization source), Finnigan MAT 95 (EI ionization source), Thermo Scientific Q Exactive GC Orbitrap GC-MS/MS (GC-MS with EI ionization source), or Thermo Scientific Q Exactive Plus (APPI ionization source).

Solvents and reagents

All reagents were purchased from commercial vendors and used as received unless otherwise noted. Unless otherwise stated, all manipulations were performed using standard Schlenk techniques under dry argon in flame-dried glassware. Reaction solvents CD_2Cl_2 and $CDCl_3$ were degassed by three cycles of freeze-pump thaw and dried with 3 Å molecular sieves.[1] Molecular sieves were activated at 200 °C under high vacuum (2×10^{-2} mbar) for at least 24 h.

i. Bismuth complexes

Triarylbismuth compounds **[Bi-2]•Ph**[2], **[Bi-3]•Ph**[3] and **[Bi-4]•Ph**[3] that served as precursors to access **[Bi-2]•Y** (section 2) were synthesized according to previously reported protocols.

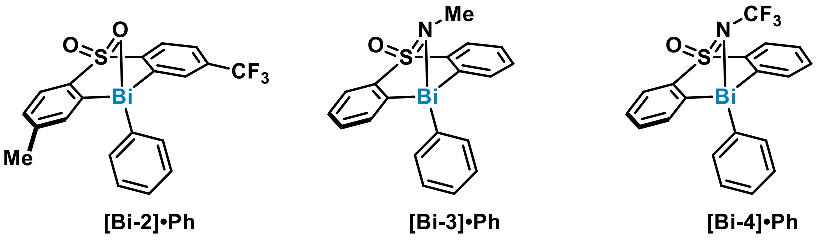

Figure S1. Overview of triarylbismuth compounds synthesized according to literature procedures.

ii. Phenyl boron reagents

Tris(pentafluorophenyl)borane (**5**, TCI), 2,4,6-triphenylboroxine (**6**, TCI) and potassium tetraphenylborate (**12**, Sigma-Aldrich) were purchased from commercial sources and used as received. Phenyl catechol boronic ester (**7**)[4], phenylboronic acid neopentylglycol ester (**8**)[5], phenylboronic acid pinacol ester (**9**)[6], $K[PhB(OH)_3]$ (**2**)[7], tetrabutylammonium aryltrifluoroborates (**4a – c**)[8], cyclictriol boronate (**10**)[9] and Li[(^{t}Bu)(Ph)Bpin] (**11**)[10] were prepared following literature procedures.

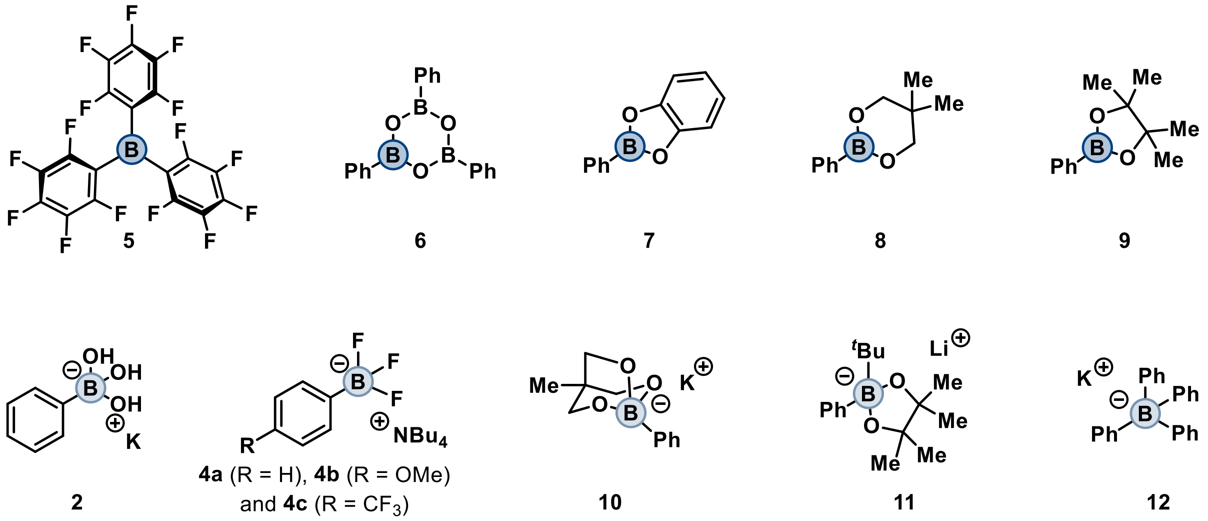

Figure S2. Overview of purchased and synthesized phenyl boron reagents.

2 Synthesis of new Bi(III) complexes

2.1 Synthesis of [Bi]•F

2.1.1 Synthesis of [Bi-2]•F

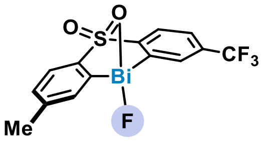

CAUTION: Due to the risk of the formation of HF, this procedure should be carried out with the appropriate safety equipment. The aqueous phase was quenched with aqueous K_2CO_3 (sat.) prior to its disposal.

A culture tube equipped with a magnetic stir bar was charged with **[Bi-2]•Ph** (500 mg, 0.86 mmol, 1.00 equiv.). The tube was evacuated and backfilled with Ar, and anhydrous CH_2Cl_2 (2.0 mL) was added. While stirring, HBF_4•Et_2O (0.14 mL, 1.03 mmol, 1.20 equiv.) was added dropwise to the solution at 25 °C, and the reaction mixture was stirred for 1 h at 25 °C. The resulting suspension was filtered, and the solids (**[Bi-2]•BF$_4$**) were collected, transferred to a 10-mL vial, and suspended in CH_2Cl_2 (3.0 mL). A solution of potassium fluoride (149 mg, 2.57 mmol, 3.00 equiv.) in water (3.0 mL) was added. The vial was closed tightly and manually agitated‡ at 25 °C for 5 min. Then, the organic phase was collected with a syringe, filtered through a PTFE filter, and the solvent was removed under reduced pressure to afford a white solid. The resulting solid was then recrystallized in EtOAc/pentane at –20 °C overnight and dried under high vacuum (2×10^{-2} mbar) for 16 h to afford **[Bi-2]•F** as a white powder (330 mg, 0.40 mmol, 73%).

^{1}H NMR (600 MHz, CD_2Cl_2, 298 K) δ 8.81 (s, 1H), 8.44 – 8.39 (m, 2H), 8.27 (d, *J* = 7.9 Hz, 1H), 7.72 (ddq, *J* = 8.0, 1.7, 0.7 Hz, 1H), 7.29 (ddq, *J* = 7.9, 1.6, 0.7 Hz, 1H), 2.45 (s, 3H).
^{13}C{^{1}H} NMR (151 MHz, CD_2Cl_2, 298 K) δ 190.7*, 147.6, 145.2 (q, *J* = 1.4 Hz), 136.9, 136.6 (q, *J* = 32.6 Hz), 135.0, 131.2 (q, *J* = 3.7 Hz), 130.0, 129.8, 129.0, 126.4 (q, *J* = 3.8 Hz), 124.3 (q, *J* = 273.6 Hz), 22.1.
^{19}F NMR (565 MHz, CD_2Cl_2, 298 K) δ −63.1 (s, 3F), −191.1 (s, 1F).
HRMS (ESI+, m/z): calculated for $C_{14}H_9Bi_1F_4Na_1O_2S_1$ [**[Bi-2]•F** + Na]$^+$ 548.9956; found 548.9955.

*corresponds to two Cs.

‡This procedure proved to be highly sensitive, as stirring or sonication of the reaction mixture resulted in the formation of an insoluble foam and substantial product loss. In contrast, this issue was not observed when the mixture was gently agitated manually.

Alternative synthesis: [Bi-2]•F can also be synthesized via oxidative ligand-ligand coupling following the procedure as described for **[Bi-4]•F** (section 2.1.3) in 51% yield.

2.1.2 Synthesis of [Bi-3]•F

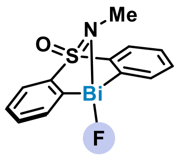

CAUTION: Due to the risk of the formation of HF, this procedure should be carried out with the appropriate safety equipment. The aqueous phase was quenched with aqueous K_2CO_3 (sat.) prior to its disposal.

A culture tube equipped with a magnetic stir bar was charged with **[Bi-3]•Ph** (250 mg, 0.49 mmol, 1.00 equiv.). The tube was evacuated and backfilled with Ar, and anhydrous CH_2Cl_2 (2.0 mL) was added. While stirring, HBF_4•Et_2O (0.13 mL, 0.97 mmol, 2.00 equiv.) was added dropwise to the solution at 25 °C, and the reaction mixture was stirred for 1 h at 25 °C. The resulting suspension was filtered, and the solids were collected, transferred to a 10-mL vial, and suspended in CH_2Cl_2 (2.0 mL). A solution of potassium fluoride (84 mg, 1.46 mmol, 3.00 equiv.) in water (2.0 mL) was added. The vial was closed tightly and manually agitated[‡] at 25 °C for 5 min. Then, the organic phase was collected with a syringe, filtered through a PTFE filter, and the solvent was removed under reduced pressure to afford a white solid. The resulting solids were resuspended in toluene (3 × 3.0 mL) and the solvent was removed *in vacuo*. Drying the resulting solid under high vacuum (2×10^{-2} mbar) for 16 h afforded **[Bi-3]•F** as a white powder (92 mg, 0.48 mmol, 43%).

^{1}H NMR (400 MHz, CD_2Cl_2, 298 K) δ 8.55 (d, *J* = 7.3 Hz, 2H), 8.21 (ddd, *J* = 7.7, 1.2, 0.5 Hz, 2H), 7.74 (td, *J* = 7.4, 1.2 Hz, 2H), 7.50 (td, *J* = 7.6, 1.2 Hz, 2H), 2.69 (s, 3H).
^{13}C{^{1}H} NMR (101 MHz, CD_2Cl_2, 298 K) δ 182.3, 139.4, 135.0, 134.9, 131.3, 129.0, 28.6.
^{19}F{^{1}H} NMR (282 MHz, CD_2Cl_2, 298 K) δ −167.9 (s, 1F).
HRMS (ESI+, m/z): calculated for $C_{13}H_{11}Bi_1F_1N_1Na_1O_1S_1$ [**[Bi-3]•F** + Na]$^+$ 480.0242; found 480.0242.

[‡]This procedure proved to be highly sensitive, as stirring or sonication of the reaction mixture resulted in the formation of an insoluble foam and substantial product loss. In contrast, this issue was not observed when the mixture was gently agitated manually.

2.1.3 Synthesis of [Bi-4]•F

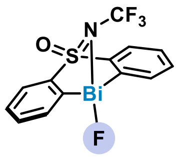

A culture tube equipped with a magnetic stir bar was charged with **[Bi-4]•Ph** (300 mg, 0.53 mmol, 1.00 equiv.) The tube was evacuated and backfilled with Ar. Anhydrous $CHCl_3$ (4.0 mL) was added and the solution was cooled to 0 °C. XeF_2 (94 mg, 0.55 mmol, 1.05 equiv.) was added in one portion and the reaction was stirred at 0 °C for 1 h. Then, the reaction mixture was distributed over two oven-dried culture tube under Ar, and the reaction mixtures were stirred at 90 °C for 16 h. After cooling to 25 °C, the mixtures were recombined. The solvent was evaporated with a positive stream of Ar, and the resulting solids were washed with pentane and dried under high vacuum (2×10^{-2} mbar) for 16 h to yield **[Bi-4]•F** as an off-white solid (246 mg, 0.48 mmol, 91%).

^{1}H NMR (400 MHz, CD_2Cl_2, 298 K) δ 8.58 (dd, J = 7.4, 1.2 Hz, 2H), 8.36 (dd, J = 7.9, 1.2 Hz, 2H), 7.82 (td, J = 7.4, 1.2 Hz, 2H), 7.55 (td, J = 7.6, 1.2 Hz, 2H).
^{13}C{^{1}H} NMR (101 MHz, CD_2Cl_2, 298 K) δ 185.8 (br s), 140.3, 136.1, 134.9, 129.7, 129.5, 122.3 (q, J = 260.5 Hz).
^{19}F NMR (376 MHz, CD_2Cl_2, 298 K) δ −41.9 (s, 3F), −183.2 (s, 1F).
HRMS (ESI+, m/z): calculated for $C_{13}H_8Bi_1F_3N_1O_1S_1$ [**[Bi-4]•F**]$^+$ 492.0077; found 492.0081.

2.2 Synthesis of [Bi-2]•Y

2.2.1 Synthesis of [Bi-2]•SO$_2$Ph, [Bi-2]•Cl and [Bi-2]•BF$_4$

Bismuth (III) complexes **[Bi-2]•Cl**[11], **[Bi-2]•BF$_4$**[2] and **[Bi-2]•SO$_2$Ph**[2] were synthesized according to previously reported protocols.

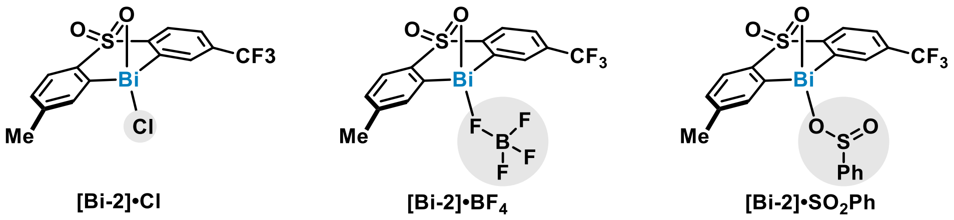

Figure S3. Overview of **[Bi-2]•Y** synthesized according to literature procedures.

2.2.2 Synthesis of [Bi-2]•OTf

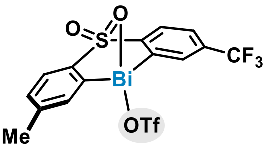

A culture tube equipped with a magnetic stir bar was charged with **[Bi-2]•Ph** (300 mg, 0.52 mmol, 1.00 equiv.). The tube was evacuated and backfilled with Ar, and anhydrous CH_2Cl_2 (2.0 mL) was added. While stirring, TfOH (50 µl, 0.56 mmol, 1.10 equiv) was added dropwise to the solution at 25 °C. The solution was stirred for 1 h at 25 °C, and the resulting solids were collected by filtration and washed with CH_2Cl_2 (3 × 2.0 mL). The resulting solids were resuspended in toluene (3 × 3.0 mL) and the solvent was removed *in vacuo*. Drying the resulting solid under high vacuum (2 × 10^{-2} mbar) for 16 h afforded **[Bi-2]•OTf** as a white solid (279 mg, 0.68 mmol, 62%).

<u>NMR data in CD_3CN:</u>

^{1}H NMR (600 MHz, CD_3CN, 298 K) δ 9.33 (s, 1H), 8.96 (s, 1H), 8.58 (dm, *J* = 8.0 Hz, 1H), 8.44 (dm, *J* = 7.9 Hz, 1H), 7.88 (ddq, *J* = 8.0, 1.7, 0.7 Hz, 1H), 7.44 (ddq, *J* = 7.9, 1.6, 0.8 Hz, 1H), 2.47 (s, 3H).
^{13}C{^{1}H} NMR (151 MHz, CD_3CN, 298 K) δ 200.3 (bs), 200.0 (bs), 150.1, 145.2, 138.0 (q, *J* = 32.2 Hz), 137.1, 136.7, 133.3 (q, *J* = 3.8 Hz), 131.4, 131.0, 130.8, 127.5 (q, *J* = 3.8 Hz), 125.1 (q, *J* = 273.1 Hz), 121.0 (q, *J* = 319.0 Hz), 22.2.
^{19}F NMR (565 MHz, CD_3CN, 298 K) δ −63.5 (s, 3F), −78.8 (s, 3F).

<u>NMR data in CD_2Cl_2:</u>

^{1}H NMR (400 MHz, CD_2Cl_2, 298 K) δ 9.23 (dt, *J* = 1.40, 0.70 Hz, 1H), 8.87 (dt, *J* = 1.39, 0.63 Hz, 1H), 8.56 (dt, *J* = 7.98, 0.71 Hz, 1H), 8.44 (d, *J* = 7.88 Hz, 1H), 7.83 (ddt, *J* = 7.97, 1.67, 0.75 Hz, 1H), 7.41 (ddt, *J* = 7.87, 1.44, 0.74 Hz, 1H), 2.50 (s, 3H).
^{13}C{^{1}H} NMR (101 MHz, CD_2Cl_2, 298 K) δ 193.4, 193.2, 149.9, 143.7, 138.4 (q, *J* = 32.78 Hz), 136.3, 135.2, 132.6 (q, *J* = 3.67 Hz), 131.3, 130.9, 130.5, 127.3 (d, *J* = 3.68 Hz), 124.2 (q, *J* = 273.95 Hz), 119.6 (q, *J* = 318.43 Hz), 22.6.
^{19}F{^{1}H} NMR (282 MHz, CD_2Cl_2, 298 K) δ −63.4 (s, 3F), −77.5 (s, 3F).

HRMS (EI, m/z): calculated for $C_{15}H_9Bi_1F_6O_5S_2$ [**[Bi-2]•OTf**]$^+$ 655.9594; found 655.9590.

2.2.3 Synthesis of [Bi-2]•I

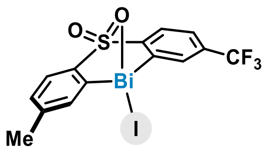

[Bi-2]•BF$_4$ (250 mg, 0.42 mmol, 1.00 equiv.) was transferred to a 10-mL vial and suspended in CH_2Cl_2 (2.0 mL). A solution of potassium iodide (210 mg, 1.26 mmol, 3.00 equiv.) in water (2.0 mL) was added. The vial was closed tightly and manually agitated at 25 °C for 5 min. Then, the organic phase was collected with a syringe, filtered through a PTFE filter, and the solvent was removed under reduced pressure to afford a white solid. The resulting solid was dried under high vacuum (2×10^{-2} mbar) for 16 h to obtain **[Bi-2]•I** (267 mg, 0.25 mmol, 59%).

^{1}H NMR (600 MHz, CD_2Cl_2, 298 K)δ 9.42 (dt, *J* = 1.8, 0.6 Hz, 1H), 9.08 (dt, *J* = 1.7, 0.5 Hz, 1H), 8.36 (dt, *J* = 8.0, 0.7 Hz, 1H), 8.21 (d, *J* = 7.9 Hz, 1H), 7.75 (ddd, *J* = 8.0, 1.7, 0.7 Hz, 1H), 7.33 (ddd, *J* = 7.9, 1.6, 0.8 Hz, 1H), 2.45 (t, *J* = 0.8 Hz, 3H).
^{13}C{^{1}H} NMR (151 MHz, CD_2Cl_2, 298 K) δ 165.8, 164.5, 148.3, 144.8 (d, *J* = 1.5 Hz), 141.6, 138.1 (q, *J* = 3.7 Hz), 137.3 (q, *J* = 32.5 Hz), 136.8, 130.3, 128.5, 127.7, 126.5 (q, *J* = 3.7 Hz), 124.0 (q, *J* = 273.8 Hz), 22.0.
^{19}F NMR (565 MHz, CD_2Cl_2, 298 K) δ −63.2 (s, 3F).
HRMS (ESI+, m/z): calculated for $C_{14}H_9Bi_1F_3I_1Na_1O_2S_1$ [**[Bi-2]•I** + Na]$^+$ 656.9016; found 656.9018.

3 μ-Fluorido intermediates

3.1 Synthesis and Characterization

3.1.1 [Bi-2]•B(Ar^F)$_3$F

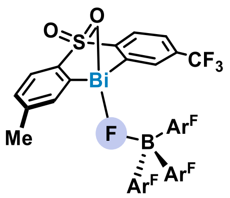

In an argon-filled glovebox, an oven-dried J. Young NMR tube was charged with **[Bi-2]•F** (7.9 mg, 0.015 mmol, 1.00 equiv.) and tris(pentafluorophenyl)borane (**5**, 9.2 mg, 0.018 mmol, 1.20 equiv.). Pre-cooled anhydrous CD_2Cl_2 (0.60 mL) was added, and the tube was sealed, removed from the glovebox, and immediately placed on dry ice until NMR measurements were performed at 233 K.

^{1}H NMR (600 MHz, CD_2Cl_2, 233 K) δ 8.73 (s, 1H), 8.60 (d, *J* = 8.0 Hz, 1H), 8.48 (d, *J* = 7.9 Hz, 1H), 8.35 (s, 1H), 7.81 (d, *J* = 8.0 Hz, 1H), 7.39 (d, *J* = 7.9 Hz, 1H), 2.23 (s, 3H).
^{13}C{^{1}H} NMR (151 MHz, CD_2Cl_2, 233 K) δ 198.6, 198.2, 149.6, 147.4 (dm, *J* = 238.9 Hz), 141.7 (q, *J* = 1.4 Hz), 139.9 (dm, *J* = 251.5 Hz), 137.9 (q, *J* = 32.9 Hz), 137.0 (dm, *J* = 248.5 Hz), 134.5, 133.2, 131.7, 131.0, 130.9 (q, *J* = 3.6 Hz), 130.5, 126.9 (q, *J* = 3.5 Hz), 123.3 (q, *J* = 274.0 Hz), 118.5 (bm), 22.0.
^{19}F NMR (565 MHz, CD_2Cl_2, 233 K) δ −63.8 (s, 3F), −135.1 – −135.3 (m, 6F), −157.5 (t, *J* = 20.5 Hz, 3F), −163.8 – −164.0 (m, 6F), −193.8 (s, 1F).

Note: *Due to to a large background from the NMR probe and the NMR tube the ^{11}B NMR signal could not be assigned.*

To obtain HRMS data the same procedure was applied but the reaction was carried out in a vial placed in a Schlenk flask. The reaction was carried out on a 0.0075 mmol scale and CH_2Cl_2 was used as a solvent. HRMS measurement was taken immediately after combination.

HRMS (ESI+, m/z): calculated for $C_{14}H_9Bi_1F_3O_2S_1$ **[Bi-2]**$^+$ 507.0074; found 507.0074. (ESI–, m/z): calculated for $C_{18}B_1F_{16}$ [B(Ar^F)$_3$F]$^-$ 530.9843; found 530.9845.

Remarks about the NMR characterization: In comparison to **[Bi-2]•F**, a new complex has formed as the major species upon the addition of **5** (**Figure S4**), which shows a 1:1 ratio between the **[Bi-2]**$^+$ and B(Ar^F)$_3$ moiety. A ^{19}F-^{19}F COSY cross peak between F15 and F102' is present (**Figure S5**). Therefore, this species was assigned as a close ion contact pair. The full assignment of the chemical shifts and 2D NMRs can be found in section 10.

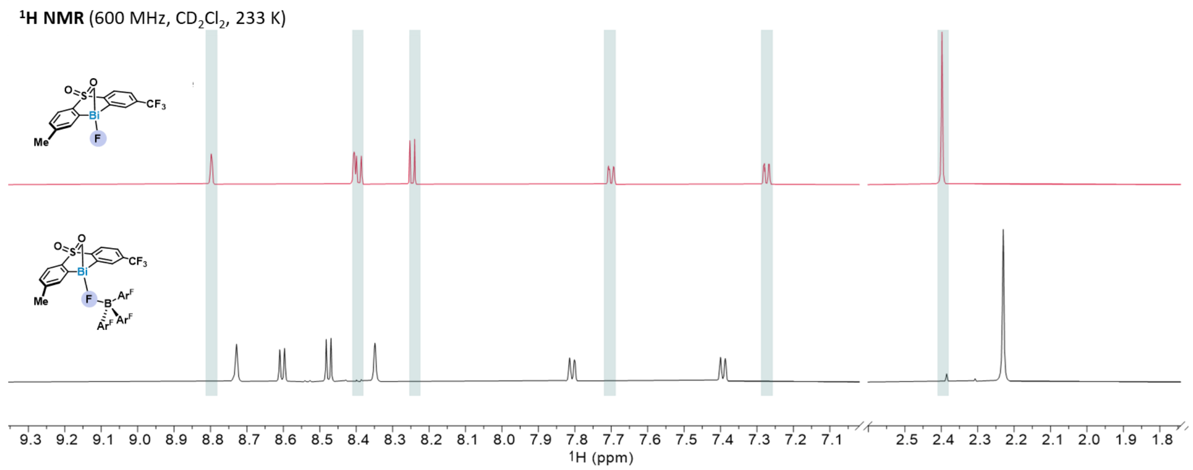

Figure S4. Comparison of the ^{1}H NMR spectra of **[Bi-2]•F** and **[Bi-2]•B(Ar^{F})$_3$F** at 233 K. The ^{1}H NMR signals of **[Bi-2]•F** are highlighted in green.

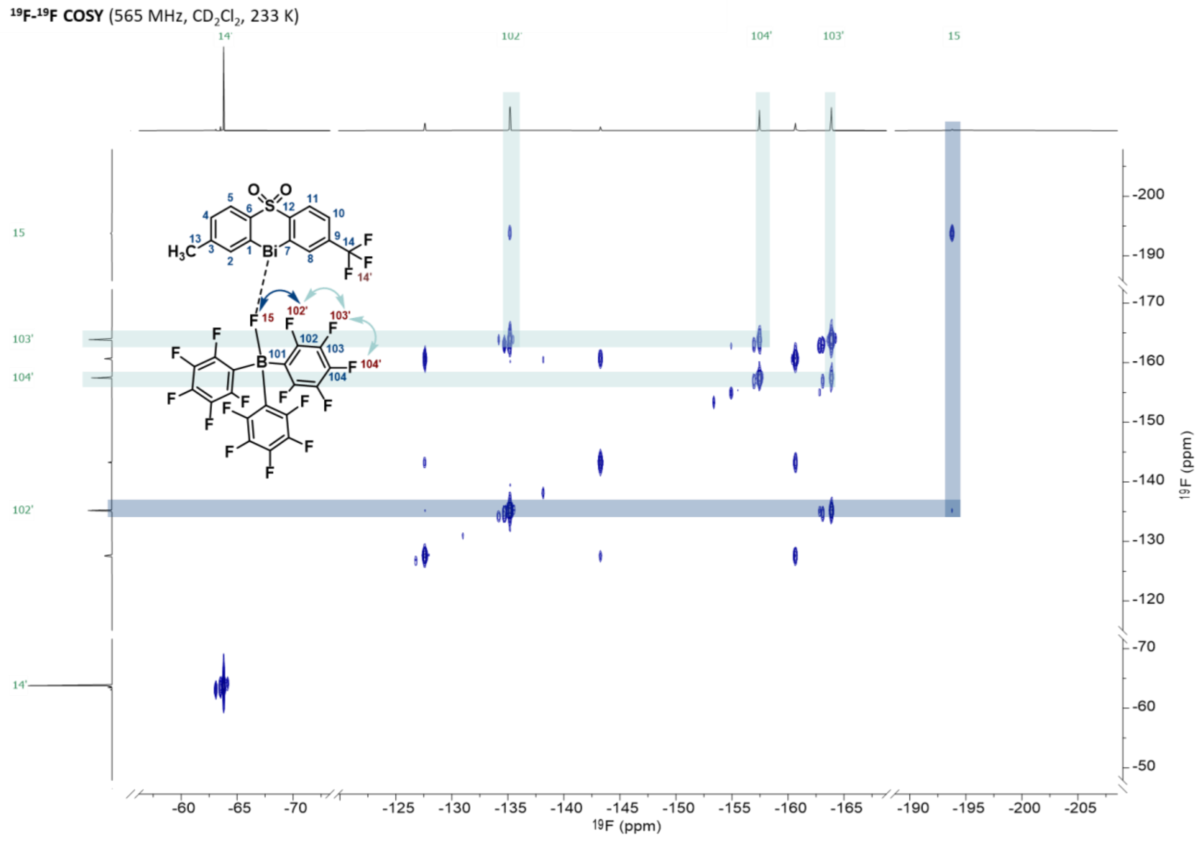

Figure S5. The ^{19}F-^{19}F COSY NMR spectrum of **[Bi-2]•B(Ar^{F})$_3$F**. The cross peak of F15 and F102’ (blue) and of F103’ to F102’ and F104’ and F104’ to F103’ (green) are highlighted.

3.1.2 [Bi-3]•B(Ar^F)$_3$F

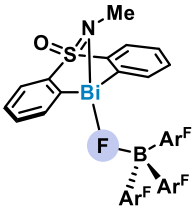

In an argon-filled glovebox, an oven-dried vial was charged with **[Bi-3]•F** (20 mg, 0.044 mmol, 1.00 equiv.) and tris(pentafluorophenyl)borane (**5**, 22 mg, 0.044 mmol, 1.00 equiv.). Anhydrous chlorobenzene (0.50 mL) was added. The solvent was taken up and dispensed with a syringe until all the solid had dissolved. The vial was fitted into a finger Schlenk containing pentane. The Schlenk flask was closed and placed in a freezer at −20 °C for 10 d until crystals had formed. The flask was warmed to 25 °C and the solvent was removed. After drying under high vacuum (2 × 10^{-2} mbar) for 24 h, 21 mg of **[Bi-3]•B(Ar^F)$_3$F** was obtained as colorless crystals.

Chlorobenzene and pentane were remaining. Considering the presence of PhCl (34% relative to **[Bi-3]•B(Ar^F)$_3$F**) and pentane (14% relative to **[Bi-3]•B(Ar^F)$_3$F**) a corrected yield of 46% was calculated.

^{1}H NMR (600 MHz, CD_2Cl_2, 298 K) δ 8.54 (dt, *J* = 7.4, 1.1 Hz, 2H), 8.35 (dd, *J* = 7.7, 1.2 Hz, 2H), 7.73 (td, *J* = 7.5, 1.2 Hz, 2H), 7.63 (td, *J* = 7.6, 1.1 Hz, 2H), 3.04 (s, 3H).
^{13}C{^{1}H} NMR (151 MHz, CD_2Cl_2, 298 K) δ 179.9, 148.2 (dm, *J* = 239.2 Hz), 140.0 (dtt, *J* = 248.4, 12.9, 5.6 Hz), 137.4 (dddd, *J* = 247.9, 20.7, 11.8, 4.2 Hz), 136.3*, 134.8, 132.5, 130.0, 121.0, 30.1.
^{19}F NMR (565 MHz, CD_2Cl_2, 298 K) δ −135.0 – −135.2 (m, 6F), −159.5 (t, *J* = 20.1 Hz, 3F), −165.1 – −165.3 (m, 6F), −183.2 (bs, 1F).
^{11}B{^{1}H} NMR (193 MHz, CD_2Cl_2, 298 K) δ 1.79.
HRMS (ESI+, m/z): calculated for $C_{13}H_{11}Bi_1N_1O_1S_1$ **[Bi-3]$^+$** 438.0360; found 438.0356. (ESI–, m/z): calculated for $C_{18}B_1F_{16}$ [B(Ar^F)$_3$F]$^-$ 530.9843; found 530.9847.

*corresponds to 2 Cs.

Suitable single crystals for X-ray diffraction were obtained by vapor diffusion of *n*-hexane into a solution of chlorobenzene (0.50 mL) containing **[Bi-3]•F** (22 mg, 0.044 mmol, 1.00 equiv.) and **5** (22 mg, 0.044 mmol, 1.00 equiv.) at –20 °C after 21 d.

Remarks about the NMR characterization: In the NMR spectra, a close contact ion pair between **[Bi-3]$^+$** and [FB(Ar^F)$_3$]$^-$ is observed. The spatial proximity of both units is supported by ^{1}H-^{19}F HOESY cross-peaks between H2 and F8, F103' and F102' (**Figure S6**). Furthermore, a ^{19}F-^{19}F COSY cross peak between F8 and F102' indicates a spatial proximity (through space or through bond coupling) (**Figure S7**). Similar to the crystal structure (section 8.1), F8 is more closely bound to the B than to Bi. The full assignment of the chemical shifts and 2D NMRs can be found in section 10.

^{1}H-^{19}F HOESY (500 MHz, CD_2Cl_2, 298 K, 300 ms mixing time)

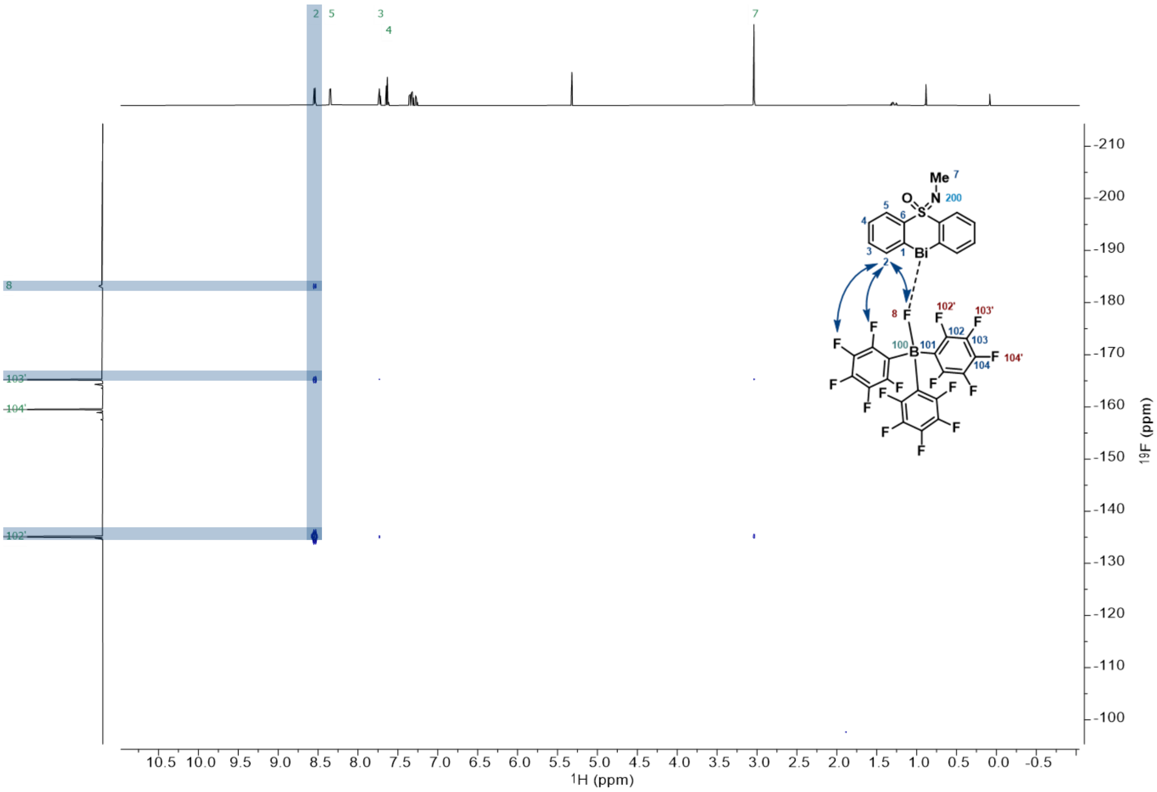

Figure S6. The ^{1}H-^{19}F HOESY NMR spectrum of **[Bi-3]•B(Ar^{F})$_3$F**. The cross-peaks between H2 and F8, F103' and F102' are highlighted in blue.

^{19}F-^{19}F COSY (565 MHz, CD_2Cl_2)

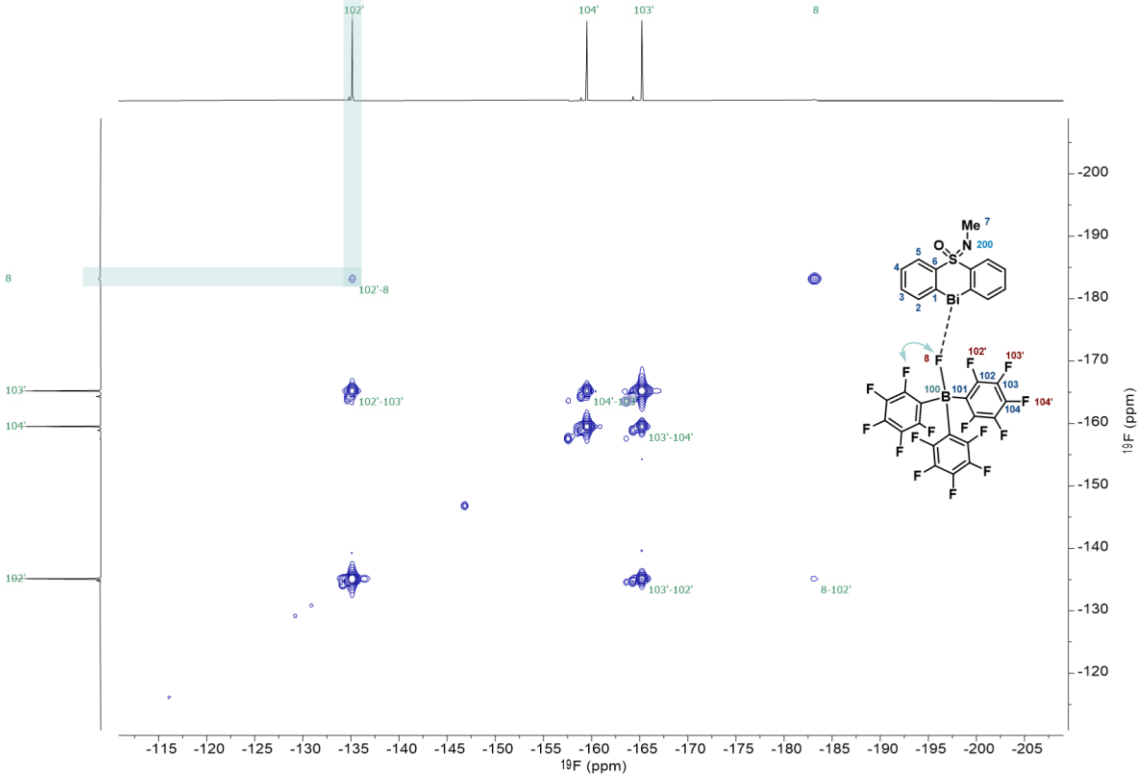

Figure S7. The ^{19}F-^{19}F-NMR COSY spectrum of **[Bi-3]•B(Ar^{F})$_3$F**. The cross-peaks between F102' and F8 are highlighted in green.

Quantification: In an argon-filled glovebox, an oven-dried NMR tube was charged with **[Bi-3]•F** (9.2 mg, 0.020 mmol, 1.00 equiv.), tris(pentafluorophenyl)borane (**5**, 12 mg, 0.024 mmol, 1.20 equiv.) and CD_2Cl_2 (0.30 mL). Then, a freshly-prepared stock solution of 1,3,5-trimethoxybenzene in anhydrous CD_2Cl_2 (0.080 mM, 0.25 mL, 0.020 mmol, 1.00 equiv.) was added. The tube was sealed, removed from the glovebox, and the sample was analyzed by quantitative ^{1}H NMR.

Quantitative ^{1}H NMR analysis revealed the formation of **[Bi-3]•B(Ar^F)$_3$F** in 91% yield.

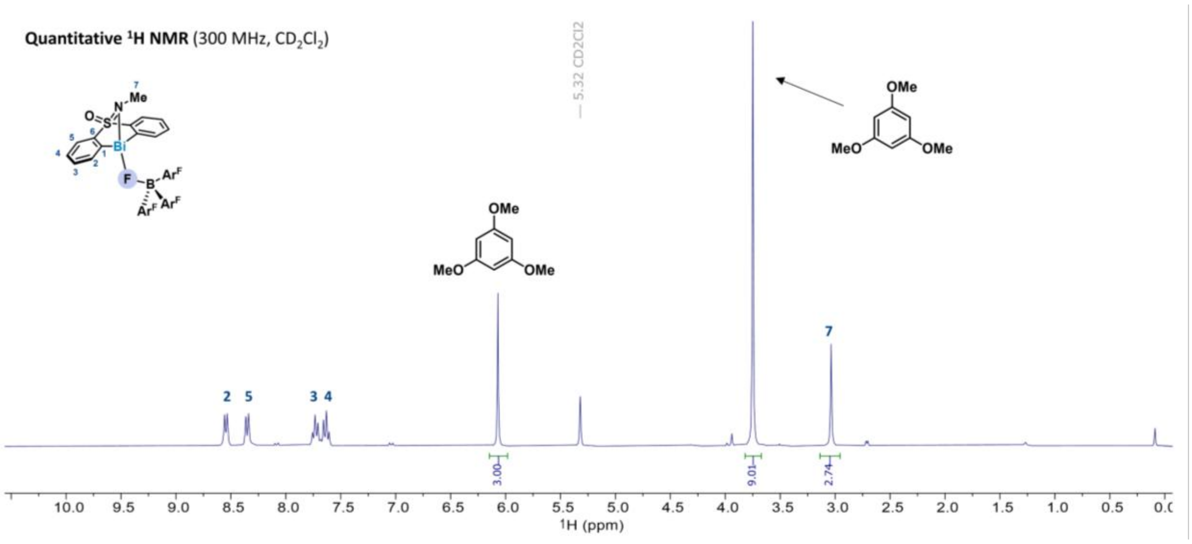

Figure S8. Quantitative ^{1}H NMR spectrum of the formation of **[Bi-2]•B(Ar^F)$_3$F.**

3.2 Transmetalation of [Bi-2]•B(Ar^F)$_3$F

3.2.1 Quantification of the transmetalation

In an argon-filled glovebox, an oven-dried NMR tube was charged with **[Bi-2]•F** (7.9 mg, 0.015 mmol, 1.00 equiv.) and tris(pentafluorophenyl)borane (**5**, 9.2 mg, 0.018 mmol, 1.20 equiv.). Anhydrous CD_2Cl_2 (0.60 mL) was added, and the tube was sealed. After 24 h at 25 °C, an NMR was measured indicating near quantitative formation of **[Bi-2]•Ar^F**. The NMR tube was opened to air and a stock solution of mesitylene in CD_2Cl_2 (0.15 M, 0.10 mL, 0.015 mmol, 1.00 equiv.) was added.

Quantitative ^{1}H NMR analysis revealed the formation **[Bi-2]•Ar^F** in 96% yield.

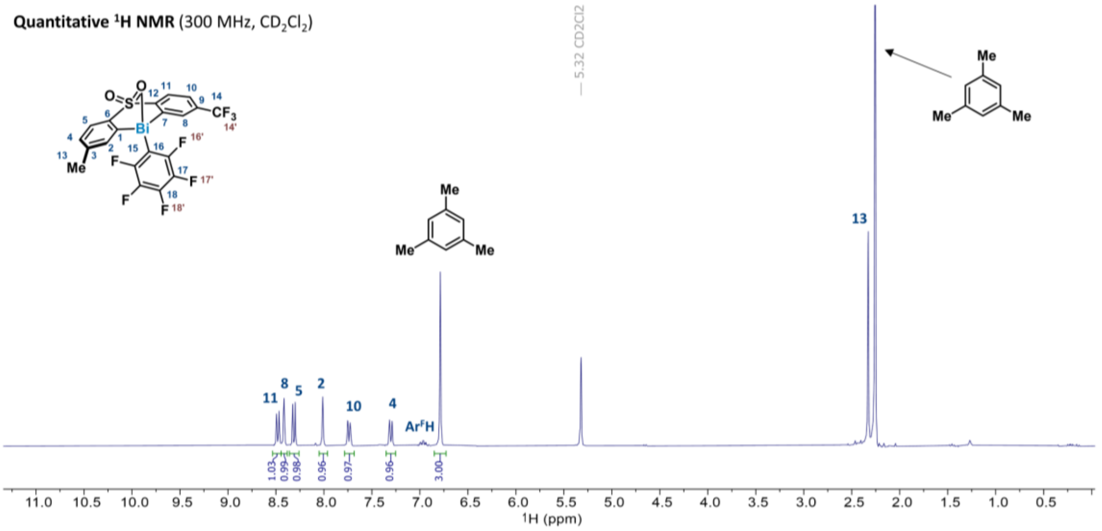

Figure S9. Quantitative ^{1}H NMR spectrum of the formation of **[Bi-2]•Ar^F** from **[Bi-2]•B(Ar^F)$_3$F.**

3.2.2 Isolation of [Bi-2]•Ar^F

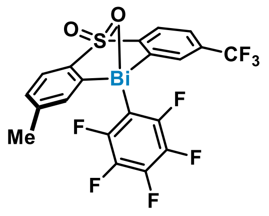

In an argon-filled glovebox, an oven-dried culture tube was charged with **[Bi-2]•F** (30 mg, 0.057 mmol, 1.00 equiv.) and tris(pentafluorophenyl)borane (**5**, 35 mg, 0.068 mmol, 1.20 equiv.). Anhydrous CH_2Cl_2 (1.8 mL) was added. After 24 h at 25 °C, the solvent was removed *in vacuo.* The reaction mixture was purified by column chromatography (pentane/ethyl acetate 9/1), and the isolated solid was dried under high vacuum (2 × 10^{-2} mbar) for 16 h to afford **[Bi-2]•Ar^F** (32 mg, 0.048 mmol, 84%) as a white solid.

^{1}H NMR (600 MHz, CD_2Cl_2, 298 K) δ 8.48 (dp, *J* = 8.0, 0.7 Hz, 1H), 8.43 – 8.40 (m, 1H), 8.32 (dt, *J* = 7.9, 0.4 Hz, 1H), 8.03 – 7.99 (m, 1H), 7.74 (ddq, 1H), 7.30 (ddq, *J* = 7.9, 1.5, 0.7 Hz, 1H), 2.32 (s, 3H).
^{13}C{^{1}H} NMR (151 MHz, CD_2Cl_2, 298 K) δ 160.6, 160.1, 148.5 (dddt, *J* = 236.9, 18.1, 9.3, 4.3 Hz), 146.3, 145.8 (q, *J* = 1.7 Hz), 142.8 (dtt, *J* = 253.5, 13.9, 5.6 Hz), 138.8 (dddd, *J* = 257.1, 22.5, 13.1, 5.8 Hz), 138.6, 137.9, 135.4 (q, *J* = 32.5 Hz), 134.9 (q, *J* = 3.6 Hz), 131.1 (t, *J* = 46.7 Hz), 130.2, 128.6, 128.0, 126.4 (q, *J* = 3.8 Hz), 123.9 (q, *J* = 273.5 Hz), 21.9.
^{19}F NMR (565 MHz, CD_2Cl_2, 298 K) δ −63.3 (s, 3F), −115.6 – −116.0 (m, 2F), −151.1 (tt, *J* = 19.6, 2.6 Hz, 1F), −159.2 – −159.4 (m, 2F).
HRMS (ESI+, m/z): calculated for $C_{20}H_9Bi_1F_8O_2S_1$ [**[Bi-2]•Ar^F** + Na]$^+$ 696.9892; found 696.9891.

3.2.3 Kinetic monitoring

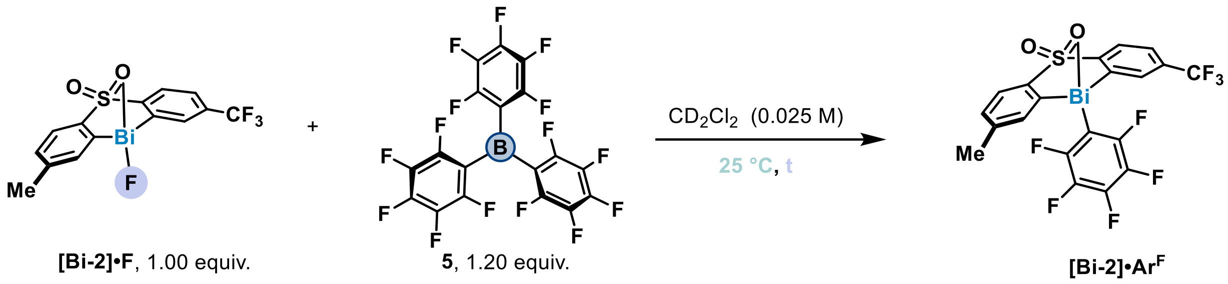

In an argon-filled glovebox, an oven-dried J. Young NMR tube was charged with **[Bi-2]•F** (7.9 mg, 0.015 mmol, 1.00 equiv.) and tris(pentafluorophenyl)borane (**5**, 9.2 mg, 0.018 mmol, 1.20 equiv.). Pre-cooled anhydrous CD_2Cl_2 (0.60 mL) was added, and the tube was sealed, removed from the glovebox, and immediately placed on dry ice until NMR measurements were performed.

^{1}H and $^{19}F\{^{1}H\}$ NMR spectra were obtained over the course of 44 h. The concentration of each component was determined relative to the sulfone ligand backbone (for ^{1}H NMR: CH_3 group (2.46 – 2.22 ppm); for ^{19}F NMR: CF_3 group (−63.20 – −64.29 ppm)).

Monitoring the reaction by ^{1}H NMR spectroscopy (**Figure S10**) showed that combination of BCF (**5**) and **[Bi-2]•F** results in the formation of **[Bi-2]•B(Ar^{F})$_3$F**. With the formation of **[Bi-2]•Ar^{F}**, signals corresponding to **[Bi-2]•B(Ar^{F})$_3$F** become broader indicating a possible equilibrium.

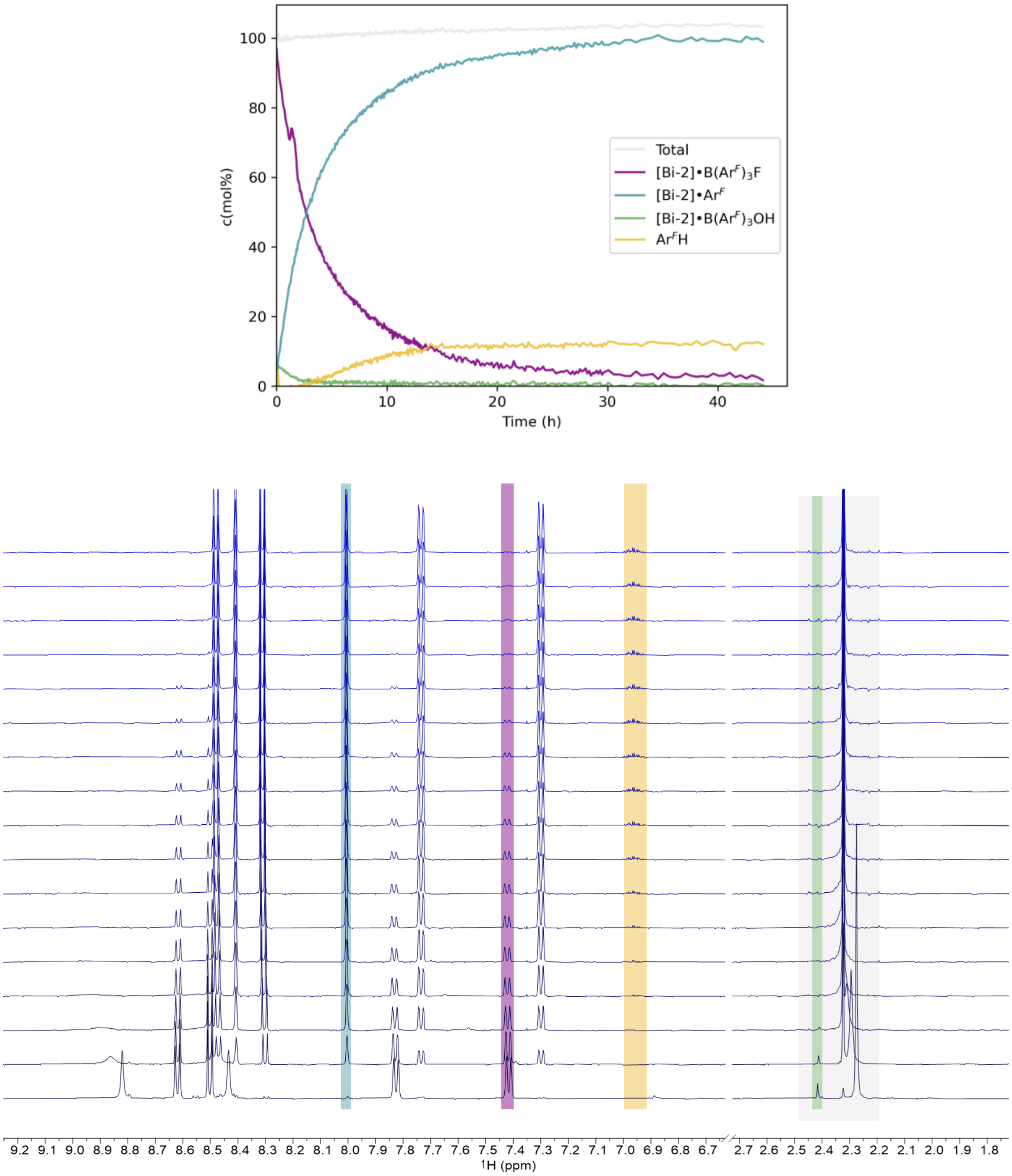

Figure S10. Conversion plot and stacked ^{1}H NMR (500 mHz) spectra of the reaction between **[Bi-2]•F** and **5** over 44 h. t = 0 refers to the time immediately after the acquisition of the first ^{1}H NMR spectrum (^{1}H NMR, 500 mHz, 298 K).

Monitoring by $^{19}F\{^{1}H\}$ NMR revealed the presence of multiple fluoride species (**Figure S11**). Interestingly, BCF (**5**) is being formed as the transmetalation proceeds. Furthermore, $(Ar^{F})BF_2$ (−74.07 ppm)[12] is observed at the end of the reaction (**Figure S12**). Notably, $(Ar^{F})_2BF$ – the expected by-product in the transmetalation – is not observed at any time in the reaction. Therefore, it is likely to be a reactive by-product.

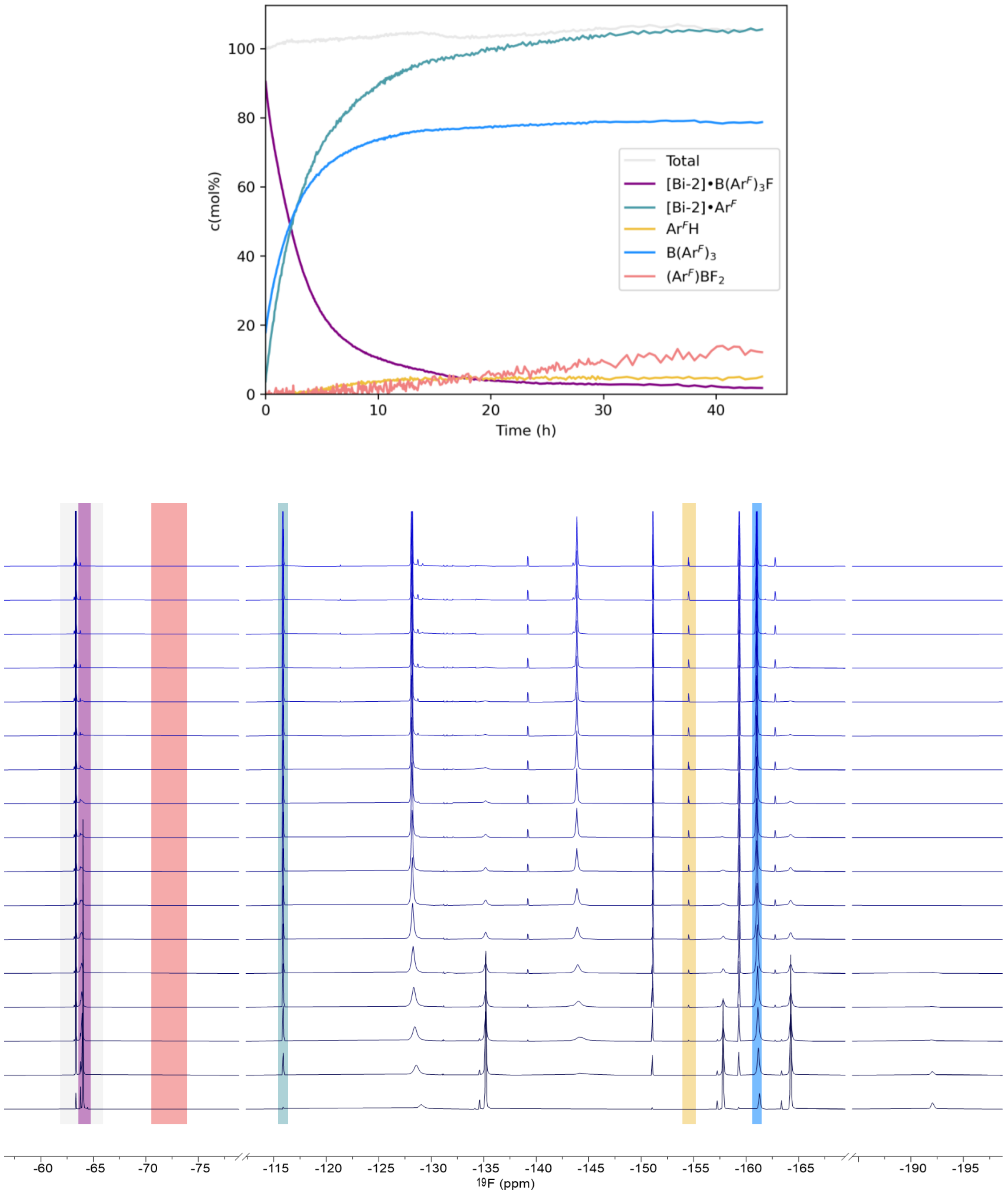

Figure S11. Conversion plot and stacked $^{19}F\{^{1}H\}$ NMR (470 mHz) spectra of the reaction between **[Bi-2]•F** and **5** over 44 h. t = 0 refers to the time immediately after the acquisition of the first $^{19}F\{^{1}H\}$ NMR spectrum.

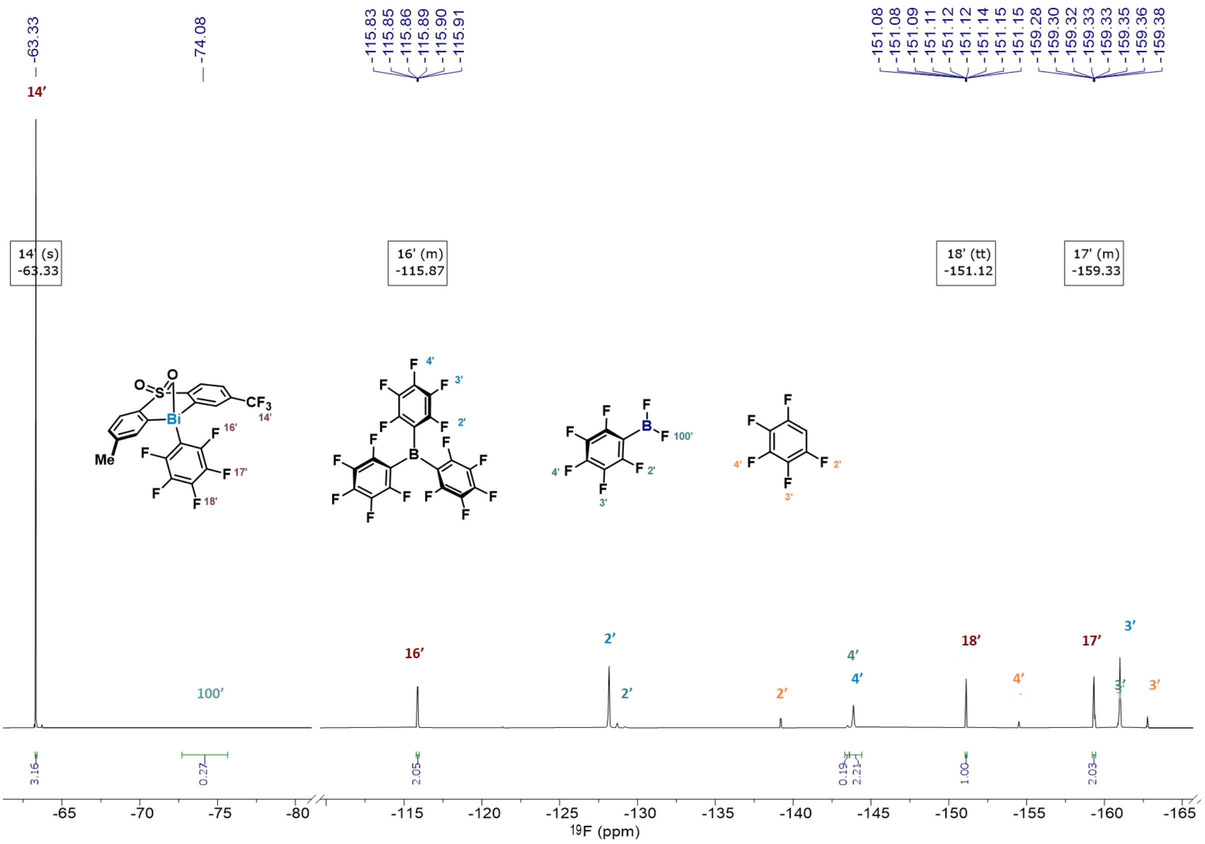

Figure S12. ^{19}F NMR (565 mHz) spectrum at the end of the reaction with the major components assigned.

4 μ-Hydroxido intermediates

4.1 Synthesis and Characterization

4.1.1 [Bi-2]•B(Ar^F)$_3$OH

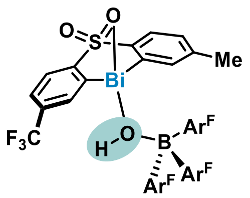

In an argon-filled glovebox, an oven-dried culture tube was charged with tris(pentafluorophenyl)borane (**5**, 136 mg, 0.27 mmol, 7.00 equiv.). The culture tube was removed from the glovebox, and anhydrous CH_2Cl_2 (4.9 mL) and H_2O (4.8 µL, 0.27 mmol, 7.00 equiv.) was added to afford a stock solution of **5**•H_2O (0.059 M).‡ Under air, a vial was charged with **[Bi-2]•F** (20 mg, 0.038 mmol, 1.00 equiv.), and the stock solution of **5**•H_2O (0.059 M, 0.70 mL, 0.042 mmol, 1.10 equiv.) was added. The solution was drawn into a syringe and repeatedly dispensed until all solids were completely dissolved. The vial was then placed inside a larger vial containing 0.70 mL of pentane. The larger vial was sealed with a cap, and the reaction mixture was stored at −20 °C for seven days to afford white crystals, which were decanted and dried under high vacuum (2 × 10^{-2} mbar) for 24 h to give **[Bi-2]•B(Ar^F)$_3$OH** (26 mg, 0.025 mmol, 67%).

‡**5**•H_2O can also be synthesized, isolated and used as a solid following the literature procedure.[13]

^{1}H NMR (600 MHz, CD_2Cl_2, 298 K) δ 8.81 – 8.78 (m, 1H), 8.56 (dm, *J* = 8.0 Hz, 1H), 8.47 (bs, 1H), 8.42 (d, *J* = 7.9 Hz, 1H), 7.84 (ddq, *J* = 8.0, 1.7, 0.7 Hz, 1H), 7.42 (dp, *J* = 7.9, 1.5 Hz, 1H), 4.55 (s, 1H), 2.42 (s, 3H).
^{13}C{^{1}H} NMR (151 MHz, CD_2Cl_2, 298 K) δ 190.1, 189.6, 149.6, 148.2 (d, *J* = 237.9 Hz), 144.0 (q, *J* = 1.4 Hz), 140.6 (dtt, *J* = 250.6, 12.9, 6.3 Hz), 138.3 (q, *J* = 33.0 Hz), 137.8 (dddd, *J* = 250.1, 21.2, 12.1, 3.7 Hz), 135.6, 135.5 (bs), 131.8 (q, *J* = 3.5 Hz), 131.3, 131.0, 130.5, 127.5 (q, *J* = 3.6 Hz), 123.8 (q, *J* = 273.9 Hz), 120.3 (bs), 22.2.
^{19}F NMR (565 MHz, CD_2Cl_2, 298 K) δ −63.8 (s, 3F), −134.6 (dd, *J* = 23.3, 6.6 Hz, 6F), −157.2 (t, *J* = 20.1 Hz, 3F), −163.4 (ddd, *J* = 24.6, 19.7, 8.6 Hz, 6F).
^{11}B{^{1}H} NMR (192 MHz, CD_2Cl_2, 298 K) δ −1.18.
HRMS (ESI+, m/z): calculated for $C_{14}H_9Bi_1F_3O_2S_1$ **[Bi-2]**$^+$ 507.0074; found 507.0071. (ESI–, m/z): calculated for $C_{18}H_1B_1F_{15}O_1$ [B(Ar^F)$_3$OH]$^-$ 528.9886; found 528.9892.

Remarks about the NMR characterization: [Bi-2]•B(Ar^F)$_3$OH exists as a close contact ion pair in solution. The signals of H13, H2, H8 and H15 show ^{1}H-^{19}F-HOESY correlations to the Ar^F-group (F102' and F103') connected to boron (**Figure S13**). Additionally, ^{1}H-^{19}F-COSY correlations of H2, H13 and H15 with F102' are observed (**Figure S14**). H2 and H8 are broadened due to through-space H-F couplings. The full assignment of the chemical shifts and 2D NMRs can be found in section 10.

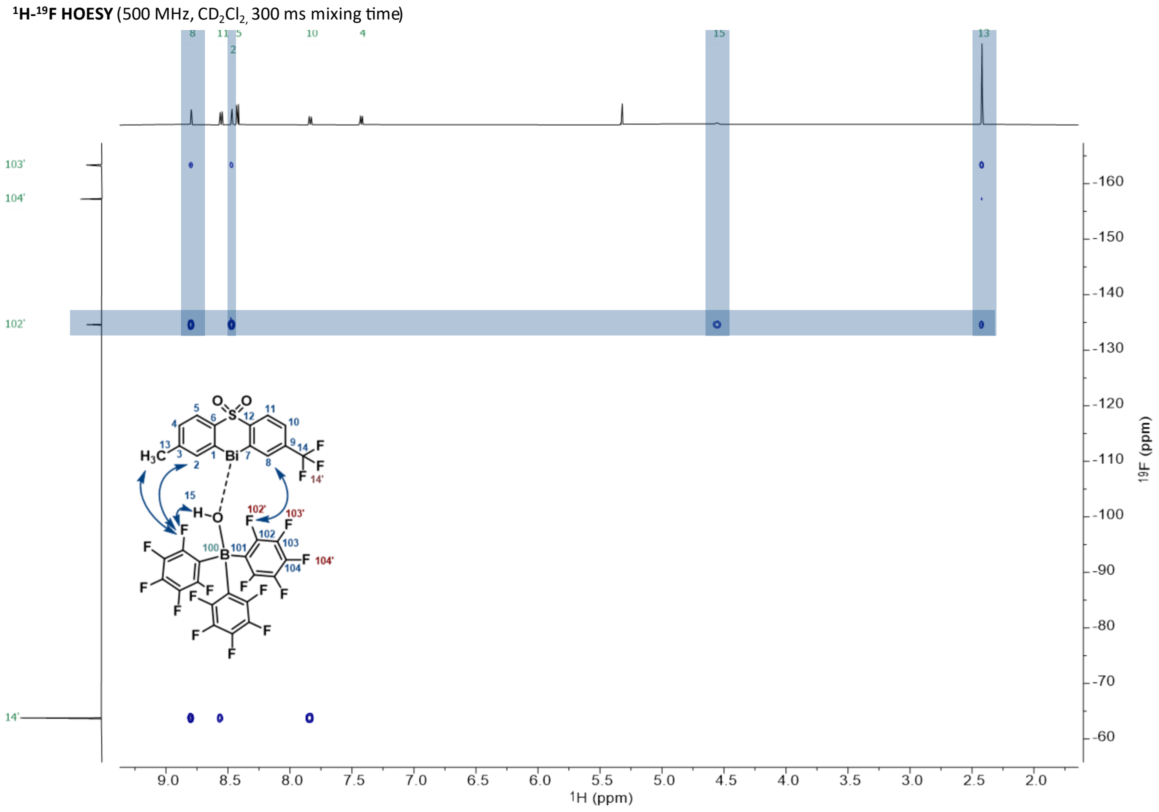

Figure S13. The ^{1}H-^{19}F HOESY NMR spectrum of **[Bi-2]•B(Ar^{F})$_3$OH**. The cross-peaks between H8, H2, H15 and H13 with F102' are highlighted in blue.

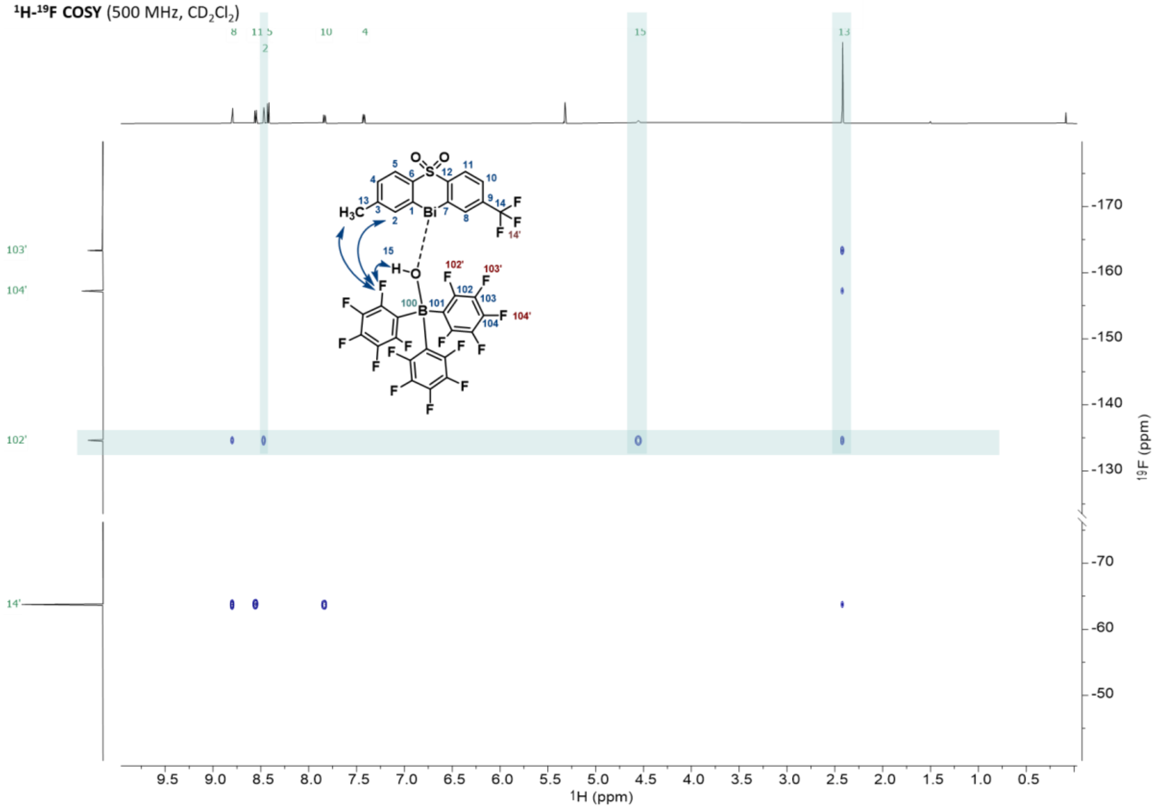

Figure S14. The ^{1}H-^{19}F COSY NMR spectrum of **[Bi-2]•B(Ar^{F})$_3$OH**. The cross-peaks between H2, H13 and H15 with F102' are highlighted in green.

4.1.2 [Bi-4]•B(Ar^F)$_3$OH

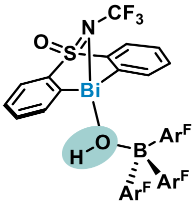

In an argon-filled glovebox, an oven-dried culture tube was charged with tris(pentafluorophenyl)borane (**5**, 136 mg, 0.27 mmol, 7.00 equiv.). The culture tube was removed from the glovebox, and anhydrous CH_2Cl_2 (4.9 mL) and H_2O (4.8 µL, 0.27 mmol, 7.00 equiv.) were added to afford a stock solution of **5**•H_2O (0.059 M).‡ Under air, a vial was charged with **[Bi-4]•F** (19 mg, 0.038 mmol, 1.00 equiv.), and the stock solution of **5**•H_2O (0.059 M, 0.70 mL, 0.042 mmol, 1.10 equiv.) was added at 25 °C. The solution was drawn into a syringe and repeatedly dispensed until all solids were completely dissolved. The vial was then placed inside a larger vial containing 0.70 mL of pentane. The larger vial was sealed with a lid, and the reaction mixture was stored at −20 °C for seven days to afford white crystals, which were decanted and dried under high vacuum (2 × 10^{-2} mbar) for 24 h to give **[Bi-2]•B(Ar^F)$_3$OH** (28 mg, 0.027 mmol, 71%).

Crystals obtained *via* this procedure were suitable for X-ray diffraction (section 8.2).

‡**5**•H_2O can also be synthesized, isolated and used as a solid following the literature procedure.[13]

^{1}H NMR (600 MHz, CD_2Cl_2, 298 K) δ 8.60 (d, *J* = 7.40 Hz, 2H), 8.48 (dd, *J* = 7.81, 1.22 Hz, 2H), 7.90 (td, *J* = 7.48, 1.22 Hz, 2H), 7.69 (td, *J* = 7.68, 1.07 Hz, 2H), 4.26 (hept, *J* = 2.90 Hz, 1H).
^{13}C{^{1}H} NMR (151 MHz, CD_2Cl_2, 298 K) δ 182.0, 148.2 (dm, *J* = 238.07 Hz), 140.4 (d, *J* = 250.30 Hz), 139.2, 137.7 (ddd, *J* = 250.05, 21.12, 12.11 Hz), 137.4, 134.9, 130.9, 130.4, 121.6 (q, *J* = 263.89 Hz), 120.7 (bs).
^{19}F NMR (565 MHz, CD_2Cl_2, 298 K) δ −42.3 (s, 3F), −134.6 – −134.7 (m, 6F), −157.6 (t, *J* = 20.25 Hz, 3F), −163.6 (m, 6F).
^{11}B{^{1}H} NMR (192 MHz, CD_2Cl_2) δ −1.70.
HRMS (ESI+, m/z): calculated for $C_{13}H_8Bi_1F_3N_1O_2S_1$ **[Bi-4]**$^+$ 492.0077; found 492.0082. (ESI–, m/z): calculated for $C_{18}H_1B_1F_{15}O_1$ [B(Ar^F)$_3$OH]$^-$ 528.9886; found 528.9893.

Comment on the stability: When stored as a solid in an argon-glovebox at 25 °C **[Bi-4]•B(Ar^F)$_3$OH** underwent complete decomposition within 49 d. Under crystallization conditions, the compound is stable for at least three weeks at −20 °C.

Remarks about the NMR characterization: The OH signal (H8) shows ^{19}F couplings to F102'. ^{1}H-^{19}F HOESY correlations of H2 to F102' and F103' and of H8 to F103’ are observed (**Figure S15**). An ^{1}H-^{13}C-HMBC cross peak of H8 to C101 but not to C1 is observed, indicating that H8 is closer to B than to Bi.

^{1}H-^{19}F HOESY (600 MHz, CD_2Cl_2, 298K, 300 ms mixing time)

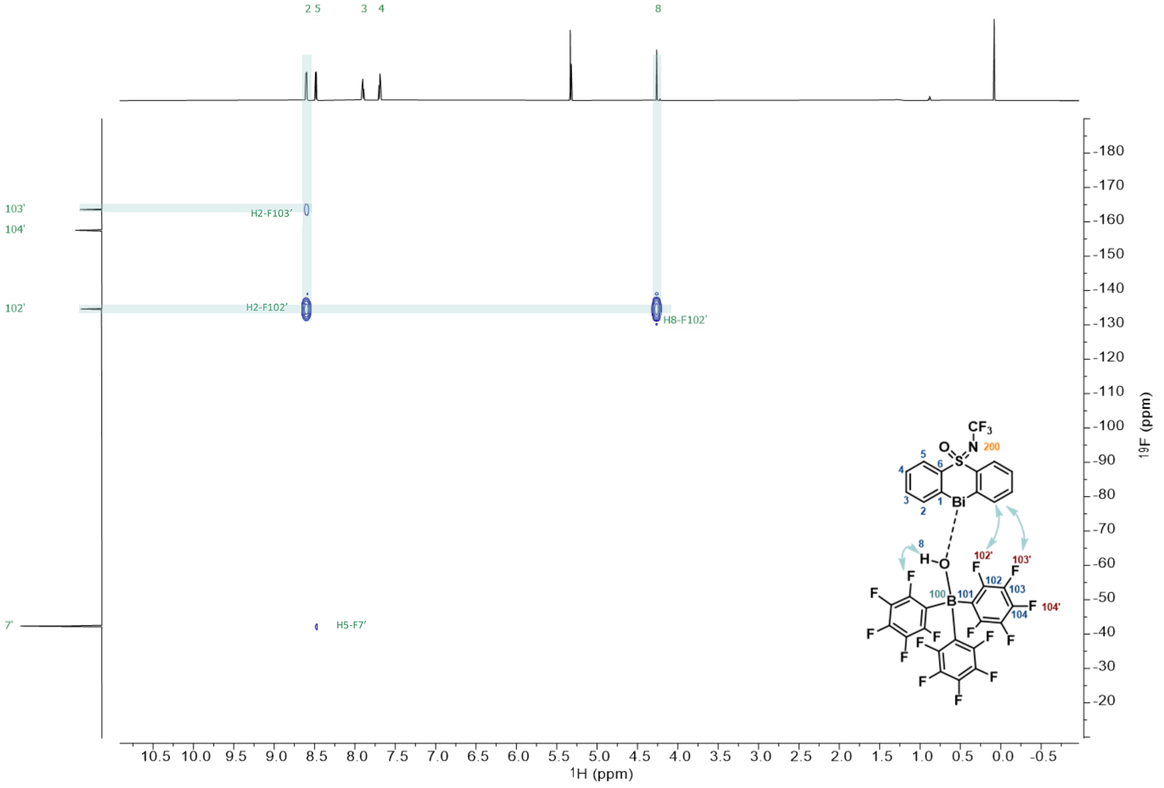

Figure S15. The ^{1}H-^{19}F HOESY NMR spectrum of **[Bi-4]•B(Ar^F)$_3$OH**. The cross-peaks between H8 and H2 with F102', and between H2 and F103', are highlighted in green.

4.2 Transmetalation of the hydroxide intermediates

4.2.1 Transmetalation from [Bi-2]•B(Ar^F)$_3$OH

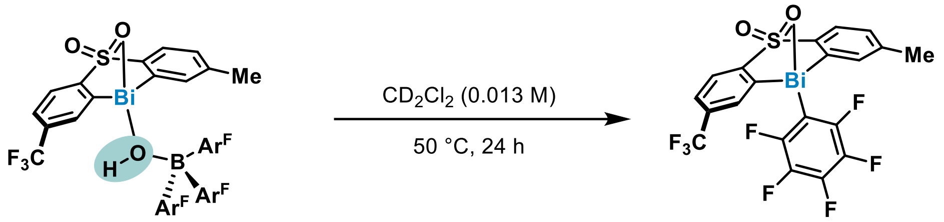

[Bi-2]•B(Ar^F)$_3$OH (1.00 equiv.) **[Bi-2]•Ar^F**

In an argon-filled glovebox, an oven-dried J. Young NMR tube was charged with freshly-prepared **[Bi-2]•B(Ar^F)$_3$OH** (7.8 mg, 0.0075 mmol, 1.00 equiv.). CD_2Cl_2 (0.60 mL) was added, and the tube was sealed, removed from the glovebox, and placed in a pre-heated oil bath at 50 °C. After 24 h, the tube was open to air, a stock solution of mesitylene in CD_2Cl_2 (0.17 M, 0.050 mL, 0.0086 mmol, 1.15 equiv.) was added, and the reaction mixture was analyzed by quantitative ^{1}H NMR.

Quantitative ^{1}H NMR analysis revealed the formation of **[Bi-2]•Ar^F** in 98% yield.

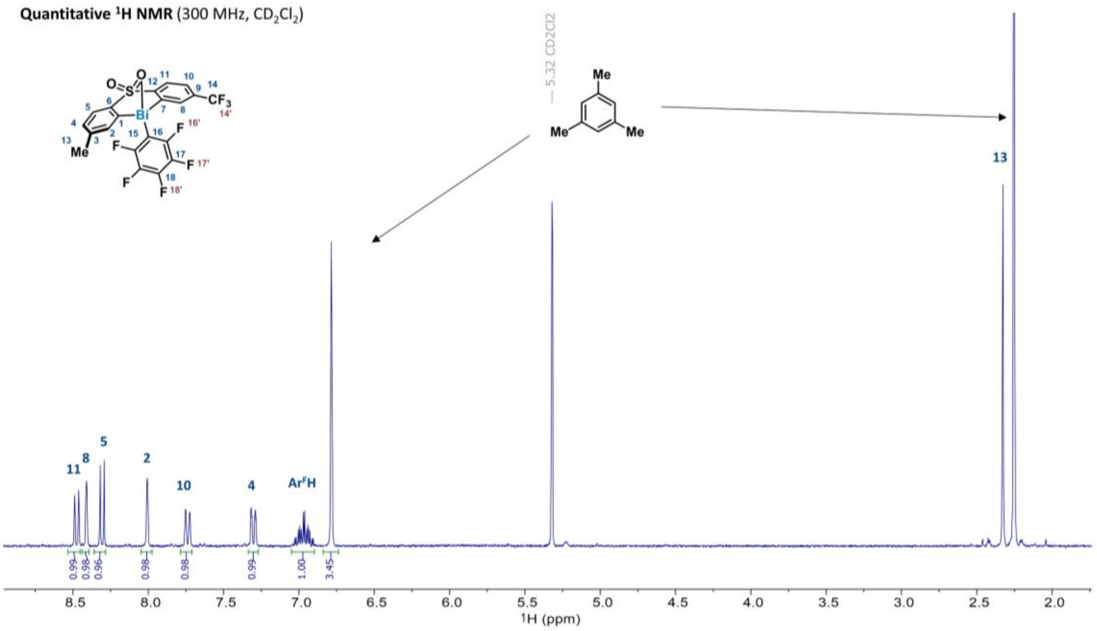

Figure S16. Quantitative ^{1}H NMR spectra after 24 h of reaction indicating the formation **[Bi-2]•Ar^F** in 98% yield.

4.2.2 Transmetalation from [Bi-4]•B(Ar^F)$_3$OH

*i. Synthesis of reference compound **[Bi-4]•Ar^F***

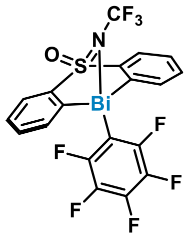

A culture tube equipped with a magnetic stir bar was charged with **[Bi-4]•F** (50 mg, 0.098 mmol, 1.00 equiv.) and pentafluorophenylboronic acid (21 mg, 0.098 mmol, 1.00 equiv.). The tube was evacuated and backfilled with Ar, and anhydrous CH_2Cl_2 (1.0 mL) was added. The solution was stirred for 2 h at 25 °C. Then, the solvent was removed *in vacuo* and the reaction was purified by column chromatography (pentane/ethyl acetate 9/1) to afford **[Bi-4]•Ar^F** as a white solid (20 mg, 0.098 mmol, 31%).

^{1}H NMR (600 MHz, CD_2Cl_2, 298 K) δ 8.47 – 8.44 (m, 2H), 8.23 – 8.20 (m, 2H), 7.58 – 7.52 (m, 4H).
^{13}C{^{1}H} NMR (151 MHz, CD_2Cl_2, 298 K) δ 158.5, 148.2 (dm, J = 236.2, 18.3, 9.3, 4.3 Hz), 142.4 (dm, J = 252.9 Hz), 139.8, 138.4 (dm, J = 257.2, 22.8, 13.4, 5.8 Hz), 138.0, 134.8, 132.3 – 131.3 (m), 129.4, 128.7, 122.1 (q, J = 259.0 Hz).
^{19}F NMR (565 MHz, CD_2Cl_2, 298 K) δ −42.2 (s, 3F), −115.6 – −115.8 (m, 2F), −151.8 (tt, J = 19.5, 2.5 Hz, 1F), −159.4 – −159.6 (m, 2F).
HRMS (ESI+, m/z): calculated for $C_{19}H_9Bi_1F_8N_1O_1S_1$ [**[Bi-4]•Ar^F** + H]$^+$ 660.0076; found 660.0077.

*ii. Transmetalation of **[Bi-4]•B(Ar^F)$_3$OH***

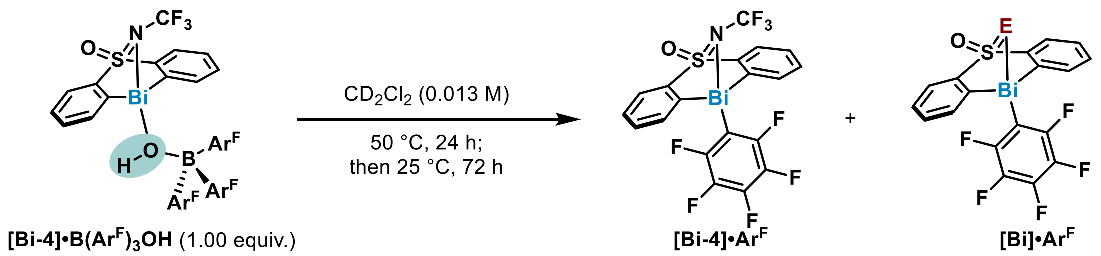

In an argon-filled glovebox, an oven-dried J. Young NMR tube was charged with **[Bi-4]•B(Ar^F)$_3$OH** (10 mg, 0.038 mmol, 1.00 equiv.). CD_2Cl_2 (0.60 mL) was added, and the tube was sealed, removed from the glovebox, and placed in a pre-heated oil bath at 50 °C. ^{1}H and ^{19}F NMR were recorded after heating the reaction to 50 °C for 24 h, and after the reaction was left for an additional 72 h at 25 °C.

After heating the complex at 50 °C for 16 h, ^{1}H NMR (**Figure S17**) and ^{19}F NMR (**Figure S18**) spectroscopy indicate the presence of trace amount of **[Bi-4]•Ar^F** alongside with **[Bi-4]•B(Ar^F)$_3$OH** and another Bi aryl species (highlighted in yellow in ^{1}H NMR spectrum **C**). Besides a new set of broad signals appear (both in ^{19}F and ^{1}H NMR). When the sample was left at 25 °C for three more days, **[Bi-4]•Ar^F** and **[Bi-4]•B(Ar^F)$_3$OH** have completely decomposed. ^{19}F NMR indicate the decomposition of the NCF_3 group. Various **[Bi]•Ar^F** species are present which are indicated by signals at around −116 ppm in ^{19}F NMR that show ^{1}H-^{19}F HOESY correlations to the signals at around 8.4 ppm in ^{1}H NMR. HRMS suggests the presence of **[Bi]•Ar^F** (m/z = 592.0200) where the ligand backbone is a detrifluoromethylated sulfoximine (**Figure S19**). In ESI$^-$ $[B(C_6F_5)_3F]^-$ and $[B(C_6F_5)_3(OH)]^-$ are observed.

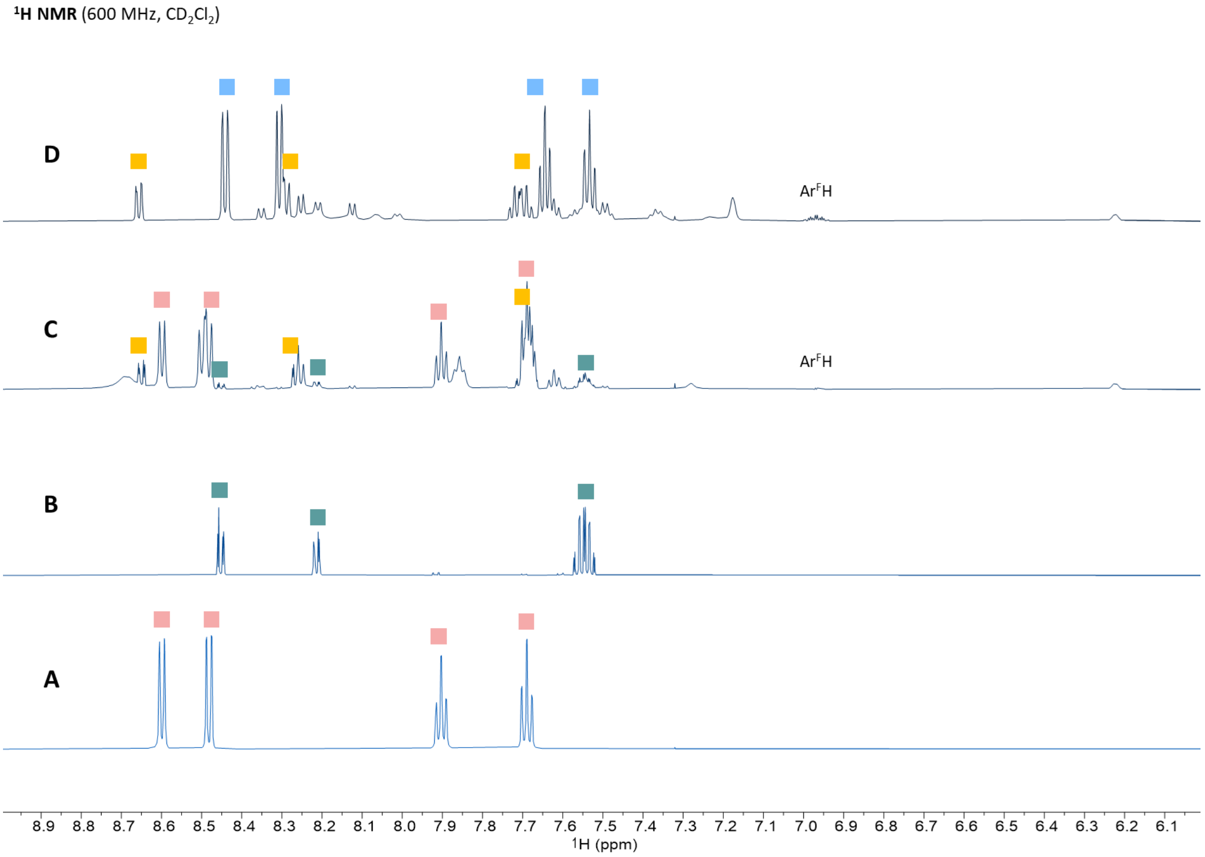

Figure S17. Stacked ^{1}H NMR spectra. **A.** Isolated **[Bi-4]•B(Ar^F)$_3$OH** (in pink) **B.** Independently synthesized **[Bi-4]• Ar^F** (in green). **C.** After heating **[Bi-4]•B(Ar^F)$_3$OH** at 50°C for 16 h**. D.** After leaving the reaction mixture for 3 more days at 25 °C. Signals marked in blue and yellow indicate the formation of two other **[Bi]•Ar^F** species. The Bi-C connectivity of the new species was confirmed by ^{1}H-^{19}F-HOESY.

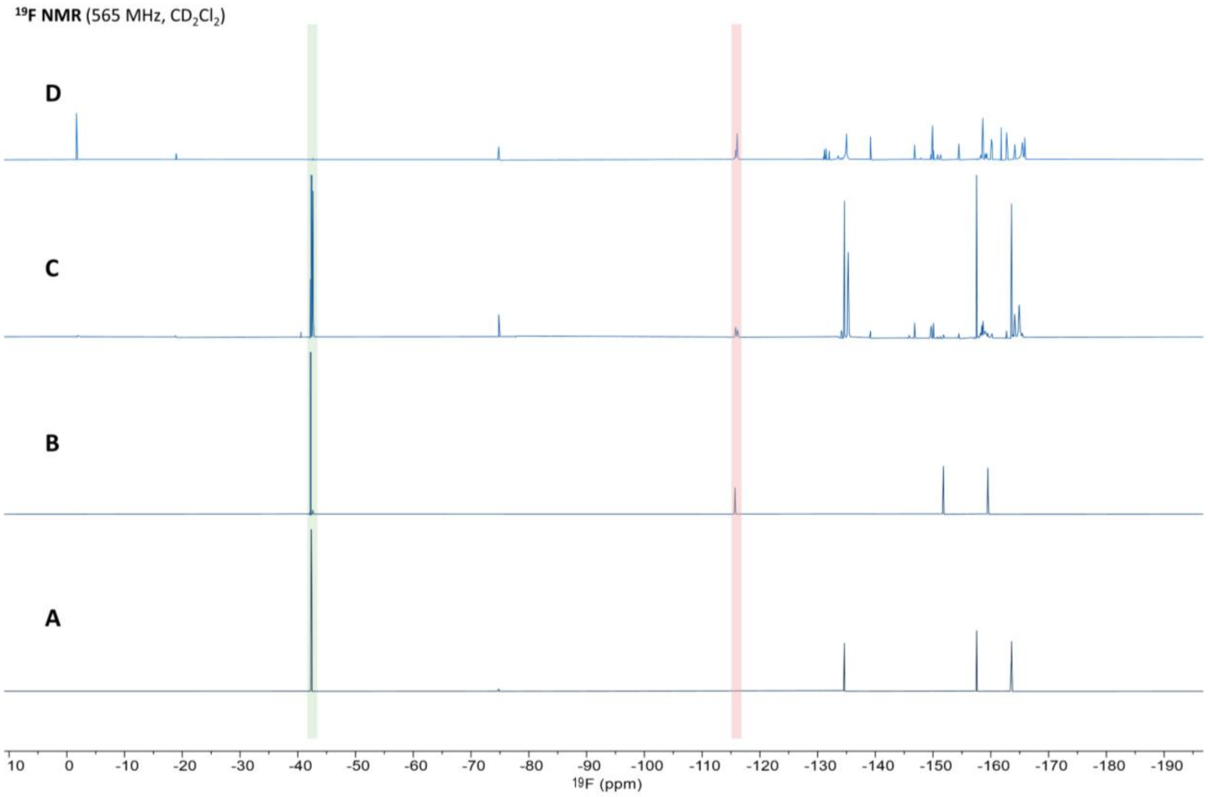

Figure S18. Stacked ^{19}F NMR spectra. **A.** Isolated **[Bi-4]•B(Ar^{F})$_3$OH** **B.** Independently synthesized **[Bi-4]•Ar^{F}** **C.** After heating **[Bi-4]•B(Ar^{F})$_3$OH** at 50 °C for 16 h. **D.** After leaving the reaction mixture for 3 more days at 25 °C. The fingerprint region of the NCF_3 group (in green) and of **[Bi]•Ar^{F}** (in red) is highlighted.

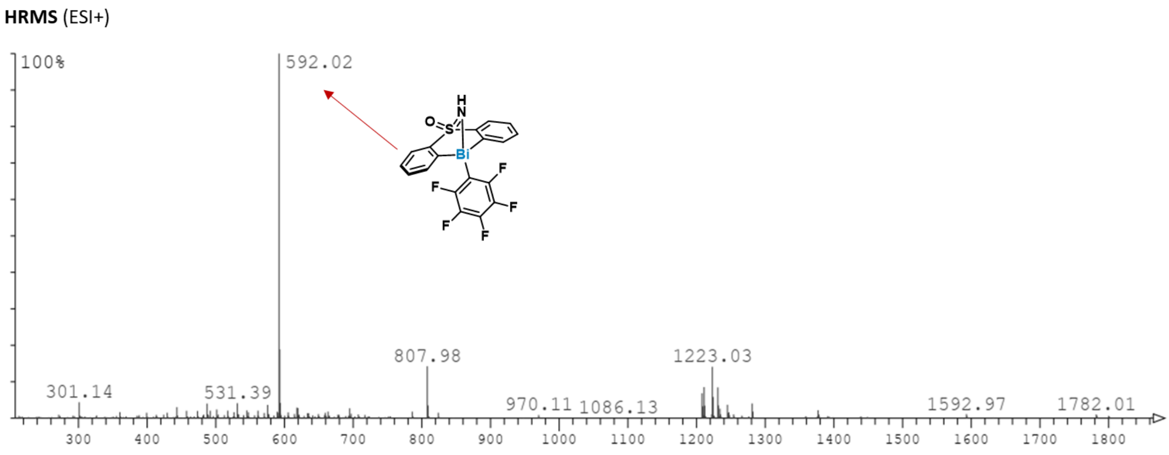

Figure S19. HRMS of the final reaction mixture (**D**)

5 Transmetalation of boron nucleophiles to [Bi-2]•F and [Bi-2]•Y

5.1 Quantification

The yields for the formation of **[Bi-2]•Ph** was determined using quantitative ^{1}H NMR. In particular the ligand backbone of **[Bi-2]•Ph** have a very distinct set of signals when compared to other **[Bi-2]•Y** species (**Figure S20**). Integration of the signals corresponding to H13, H8 or H4 were used for quantification (**Figure S21**).

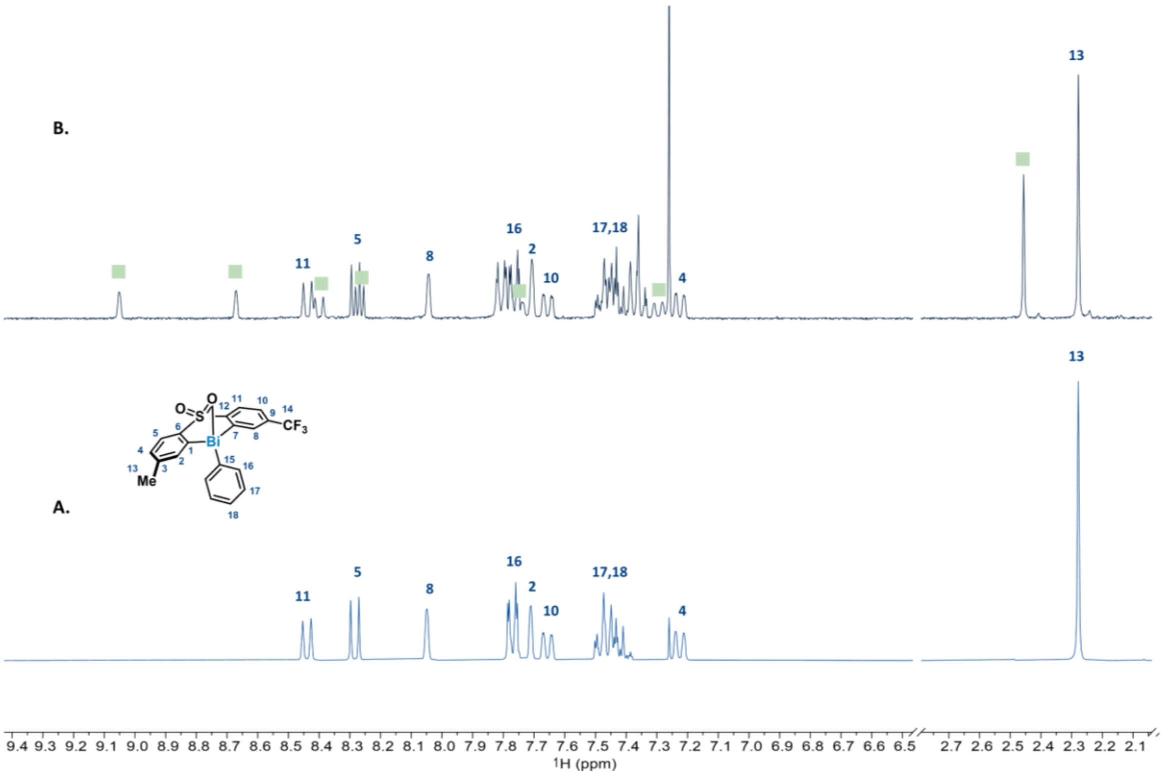

Figure S20. A. Clean ^{1}H NMR spectrum of **[Bi-2]•Ph B.** Crude NMR of the reaction between PhBpin (**9**) and **[Bi-2]•F**. **[Bi-2]•Cl**, which forms upon the work-up with brine, is present and has a distinct set of signal (highlighted in green)

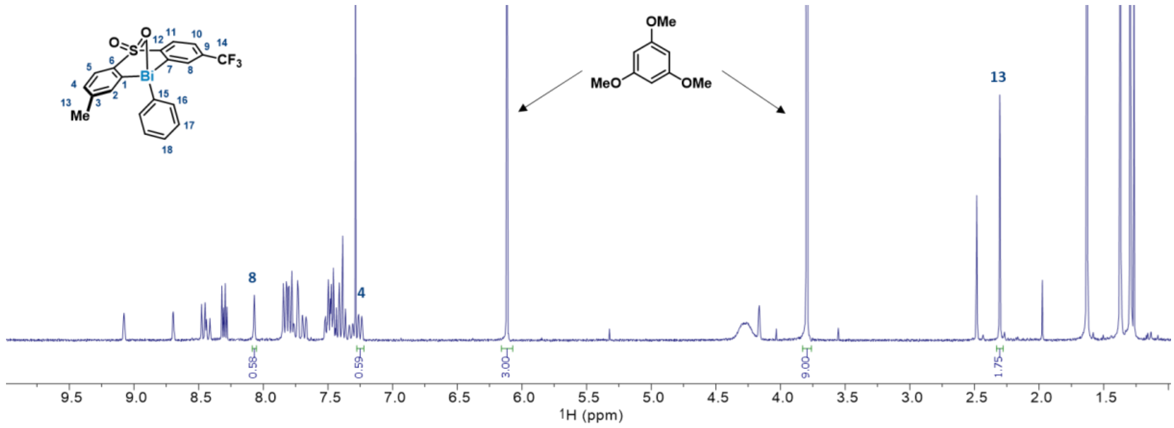

Figure S21. Quantification of the formation of **[Bi-2]•Ph** from **[Bi-2]•F** and PhBpin.

5.2 Neutral pathway

5.2.1 Transmetalation with 3

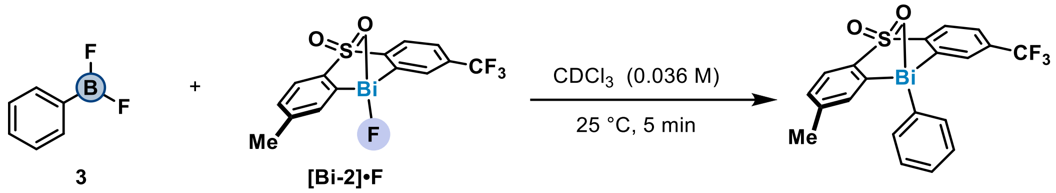

Preparation of the stock solution of **3** in $CDCl_3$: A culture tube equipped with a magnetic stir bar was charged with potassium phenyltrifluoroborate (28 mg, 0.15 mmol, 1.00 equiv.). The tube was evacuated and backfilled with Ar, and anhydrous $CDCl_3$ (1.5 mL) was added. While stirring, boron trifluoride etherate (0.076 mL, 0.60 mmol, 4.00 equiv) was added in one portion. The reaction mixture was stirred for 2 h at 25 °C which resulted in a 0.090 M solution of $PhBF_2$ in $CDCl_3$ as determined by quantitative ^1H NMR using 1,3,5-trimethoxybenzene as an internal standard.

Transmetalation: In an argon-filled glovebox, an oven-dried NMR tube was charged with **[Bi-2]•F** (11 mg, 0.020 mmol, 1.00 equiv.). A freshly-prepared stock solution of 1,3,5-trimethoxybenzene in anhydrous $CDCl_3$ (0.080 M, 0.25 mL, 0.020 mmol, 1.00 equiv.) was added, followed by the freshly-prepared stock solution of $PhBF_2$ (**3**) in anhydrous $CDCl_3$ (0.090 M, 0.30 mL, 0.027 mmol, 1.35 equiv.). The tube was sealed, removed from the glovebox, and the sample was analyzed by quantitative ^1H NMR (5 min after the addition of **3**).

Quantitative ^1H NMR analysis of the sample indicated quantitative formation **[Bi-2]•Ph**.

Note: When the reaction mixture was exposed to air after the transmetalation, **[Bi-2]•Ph** completely decomposes within 24 h *via* protodebismuthation to form benzene and **[Bi-2]•Y** cation.

5.2.2 Transmetalation with 6

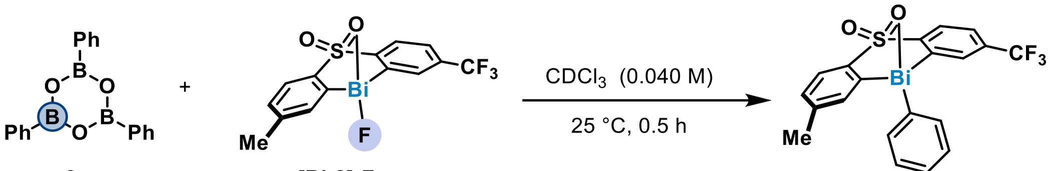

6 (0.40 equiv.) **[Bi-2]•F** (1.00 equiv.) **[Bi-2]•Ph:** 98%

In an argon-filled glovebox, an oven-dried culture tube was charged with **[Bi-2]•F** (11 mg, 0.020 mmol, 1.00 equiv.), 2,4,6-triphenylboroxine (**6**, 2.5 mg, 0.0080 mmol, 0.40 equiv.) and $CDCl_3$ (0.50 mL). The tube was removed from the glovebox and stirred for 0.5 h at 25 °C. The reaction was open to air, and brine (2.0 mL), followed by a freshly prepared stock solution of 1,3,5-trimethoxybenzene in $CDCl_3$ (0.080 mM, 0.25 mL, 0.020 mmol, 1.00 equiv.) was added. The organic phase was transferred to an NMR tube, and the yield was determined by quantitative ^{1}H NMR.

Quantitative ^{1}H NMR analysis of the sample indicated the formation of 98% of **[Bi-2]•Ph**.

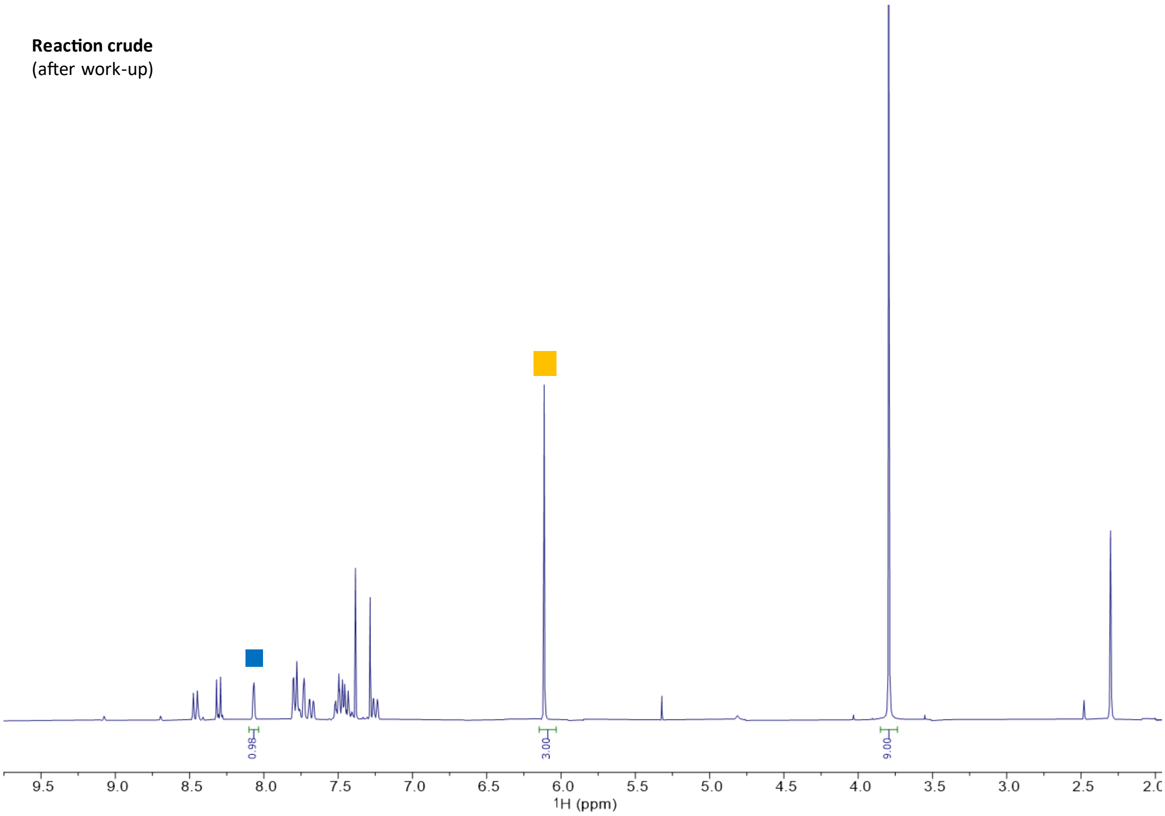

Figure S22. ^{1}H NMR of the reaction with **3** (in blue: **[Bi-2]•Ph**; in yellow: 1,3,5-trimethoxybenzene).

5.2.3 Transmetalation with 7

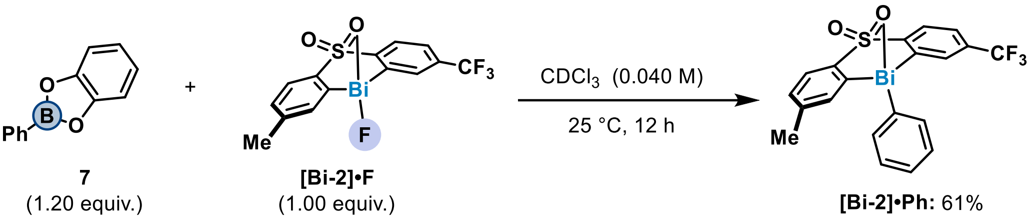

7 (1.20 equiv.) **[Bi-2]•F** (1.00 equiv.) **[Bi-2]•Ph:** 61%

In an argon-filled glovebox, an oven-dried culture tube was charged with **[Bi-2]•F** (11 mg, 0.020 mmol, 1.00 equiv.), 2-phenyl-1,3,2-benzodioxaborole (**7**, 4.7 mg, 0.024 mmol, 1.20 equiv.) and $CDCl_3$ (0.50 mL). After 12 h at 25 °C, the reaction mixture was quenched by the addition of brine (2.0 mL), and a freshly-prepared stock solution of 1,3,5-trimethoxybenzene in $CDCl_3$ (0.080 mM, 0.25 mL, 0.020 mmol, 1.00 equiv.) was added. The organic phase was transferred to an NMR tube, and the yield was determined by quantitative ^{1}H NMR.

Quantitative ^{1}H NMR analysis of the sample indicated the formation of 61% of **[Bi-2]•Ph**.

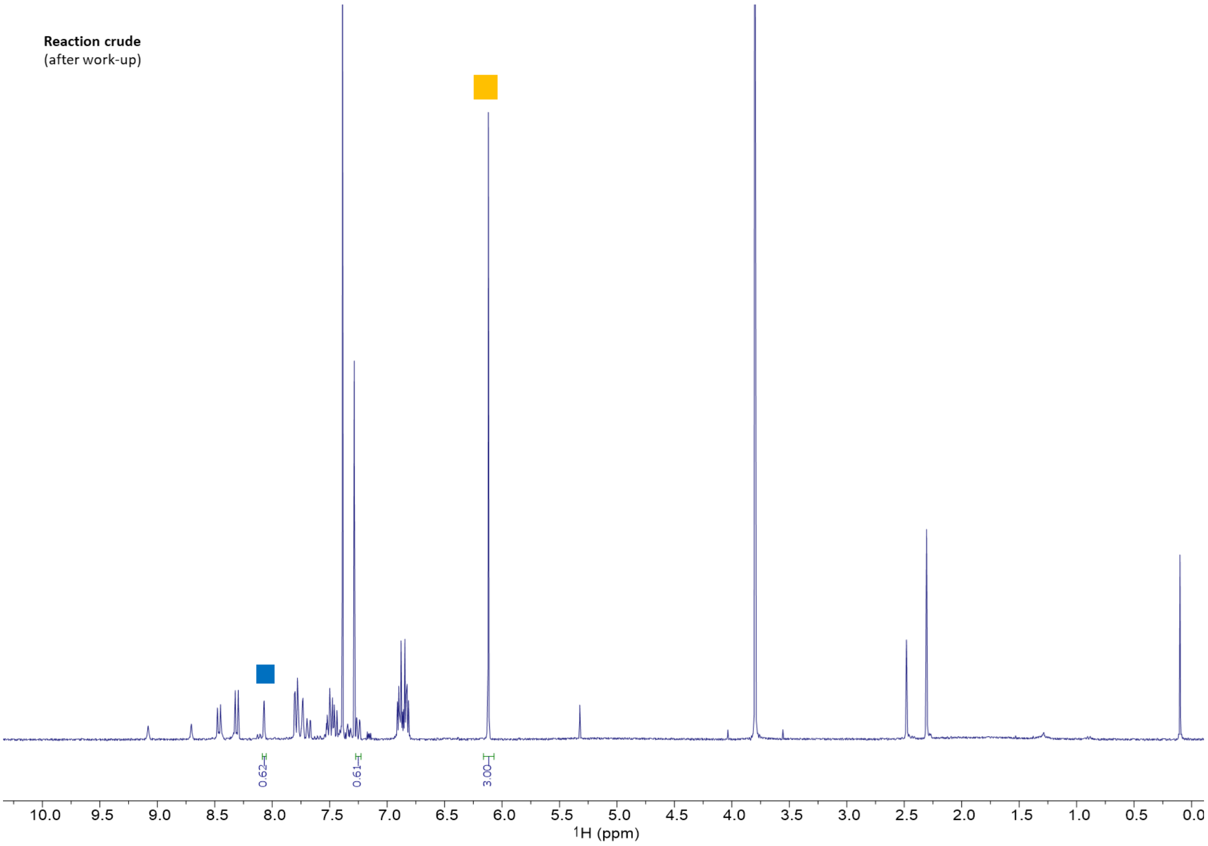

Figure S23. ^{1}H NMR of the reaction with **7** (in blue: **[Bi-2]•Ph**; in yellow: 1,3,5-trimethoxybenzene).

5.2.4 Transmetalation with 8

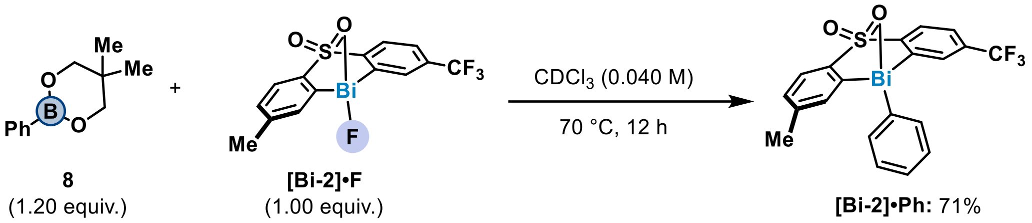

In an argon-filled glovebox, an oven-dried culture tube equipped with a magnetic stir bar was charged with **[Bi-2]•F** (11 mg, 0.020 mmol, 1.00 equiv.), phenylboronic acid neopentyl glycol ester (**8**, 4.6 mg, 0.024 mmol, 1.20 equiv.) and $CDCl_3$ (0.50 mL). The tube was removed from the glovebox and placed in a preheated oil bath at 70 °C. The reaction mixture was stirred at 70 °C for 12 h and then cooled to 25 °C. The reaction was open to air, and brine (2.0 mL), followed by a freshly prepared stock solution of 1,3,5-trimethoxybenzene in $CDCl_3$ (0.080 mM, 0.25 mL, 0.020 mmol, 1.00 equiv.) was added. The organic phase was transferred to an NMR tube, and the yield was determined by quantitative ^{1}H NMR.

Quantitative ^{1}H NMR analysis of the sample indicated the formation of 71% of **[Bi-2]•Ph**.

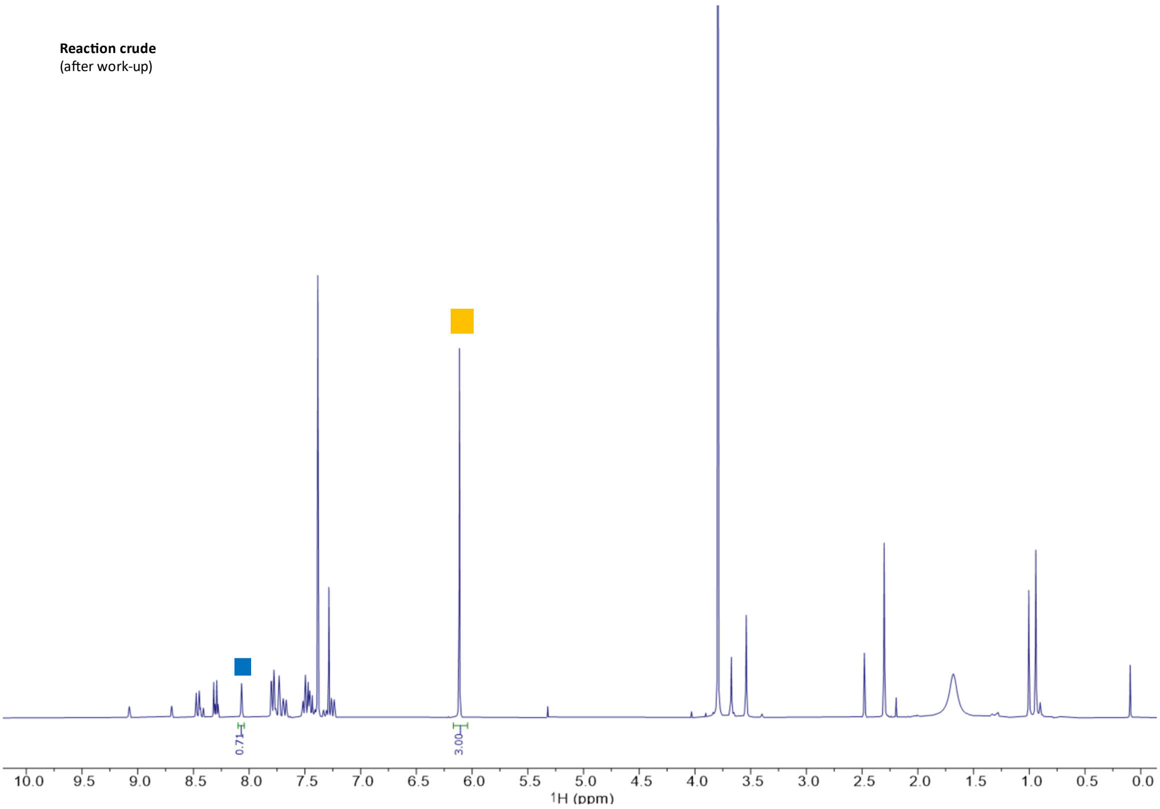

Figure S24. ^{1}H NMR of the reaction with **8** (in blue: **[Bi-2]•Ph**; in yellow: 1,3,5-trimethoxybenzene).

5.2.5 Transmetalation with 9

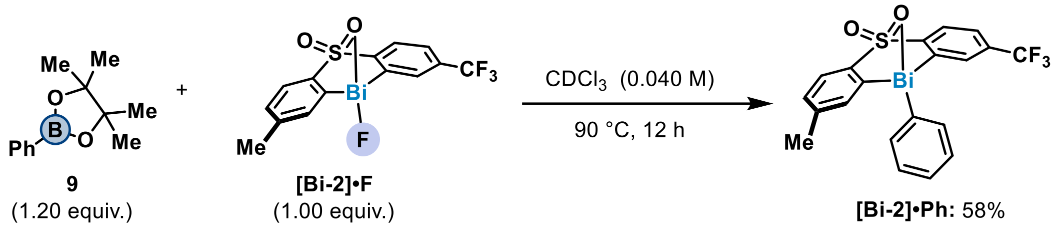

In an argon-filled glovebox, an oven-dried culture tube equipped with a magnetic stir bar was charged with **[Bi-2]•F** (11 mg, 0.020 mmol, 1.00 equiv.), phenylboronic acid pinacol ester (**9**, 4.9 mg, 0.024 mmol, 1.20 equiv.) and $CDCl_3$ (0.50 mL). The tube was removed from the glovebox and placed in a preheated oil bath at 90 °C. The reaction mixture was stirred at 90 °C for 12 h and then cooled to 25 °C. The reaction mixture was opened to air, a freshly prepared stock solution of 1,3,5-trimethoxybenzene in $CDCl_3$ (0.080 mM, 0.25 mL, 0.020 mmol, 1.00 equiv.), and the yield was determined by quantitative ^{1}H NMR.

Quantitative ^{1}H NMR analysis of the sample indicated the formation of 58% of **[Bi-2]•Ph**.

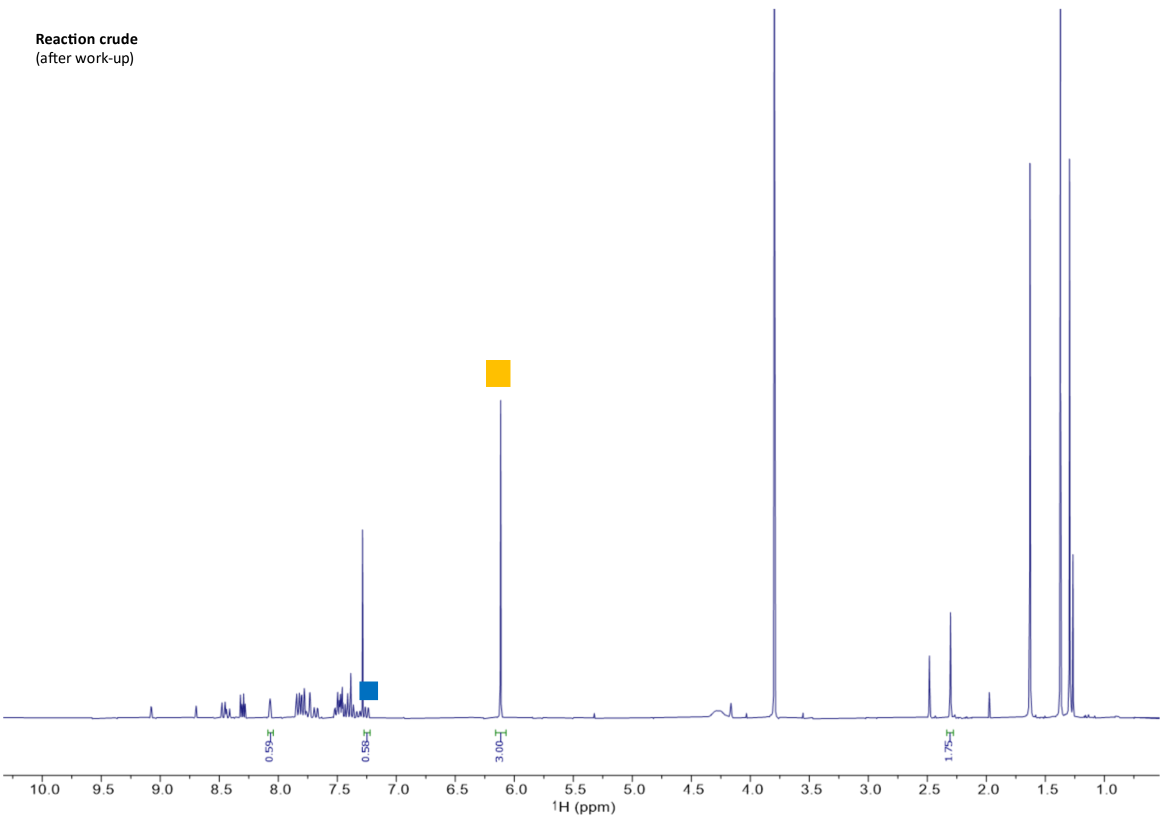

Figure S25. ^{1}H NMR spectrum of the reaction with **9** (in blue: **[Bi-2]•Ph**; in yellow: 1,3,5-trimethoxybenzene).

5.3 Ionic pathway

5.3.1 Transmetalation with 2

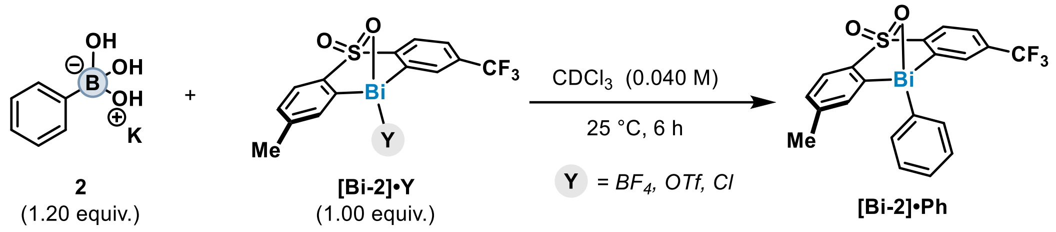

In an argon-filled glovebox, an oven-dried culture tube equipped with a magnetic stir bar was charged with **[Bi-2]•Y** (**Y = Cl:** 11 mg; **OTf:** 13 mg; **BF_4:** 12 mg, 0.020 mmol, 1.00 equiv.), K[Ph-B(OH)]$_3$ (**2**, 4.3 mg, 0.024 mmol, 1.20 equiv) and $CDCl_3$ (0.50 mL). The tube was removed from the glovebox and stirred for 6 h at 25 °C. The reaction was open to air, and brine (2.0 mL), followed by a freshly prepared stock solution of 1,3,5-trimethoxybenzene in $CDCl_3$ (0.080 mM, 0.25 mL, 0.020 mmol, 1.00 equiv.) was added. The organic phase was transferred to an NMR tube, and the yield was determined by quantitative ^{1}H NMR.

Quantitative ^{1}H NMR analysis of the sample indicated the formation of **[Bi-2]•Ph** in 86% (Y = Cl), 88% (Y = OTf) and 84% (Y = BF_4) yield.

5.3.2 Transmetalation with 4a

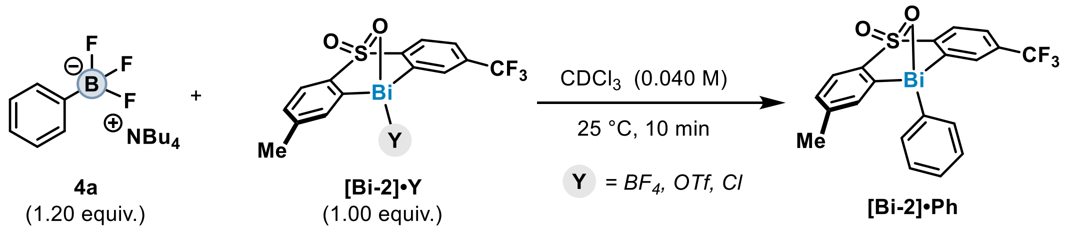

In an argon-filled glovebox, an oven-dried culture tube equipped with a magnetic stir bar was charged with **[Bi-2]•Y** (**Y = Cl:** 11 mg; **OTf:** 13 mg; **BF_4:** 12 mg, 0.020 mmol, 1.00 equiv.), [Ph-BF_3][NBu_4] (**4a**, 9.3 mg, 0.024 mmol, 1.20 equiv.) and $CDCl_3$ (0.50 mL). The tube was removed from the glovebox and stirred for 10 min at 25 °C. The reaction was open to air, and brine (2.0 mL), followed by a freshly prepared stock solution of 1,3,5-trimethoxybenzene in $CDCl_3$ (0.080 mM, 0.25 mL, 0.020 mmol, 1.00 equiv.) was added. The organic phase was transferred to an NMR tube, and the yield was determined by quantitative ^{1}H NMR.

Quantitative ^{1}H NMR analysis of the sample indicated the formation of **[Bi-2]•Ph** in 0% (Y = Cl), 60% (Y = OTf) and 62% (Y = BF_4) yield.

5.3.3 Transmetalation with 10

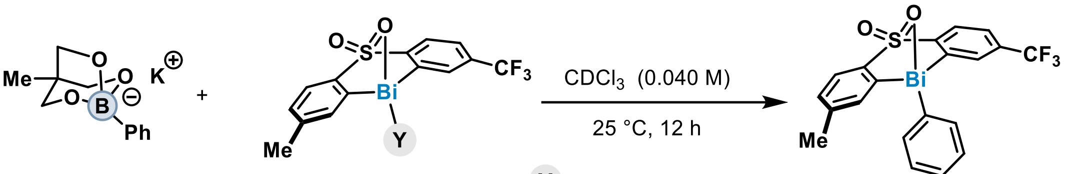

10
(1.10 equiv.)

[Bi-2]•Y
(1.00 equiv.)

[Bi-2]•Ph

In an argon-filled glovebox, an oven-dried culture tube equipped with a magnetic stir bar was charged with **[Bi-2]•Y** (**Y = Cl:** 11 mg; **OTf:** 13 mg; **I**: 13 mg; **BF_4:** 12 mg, **SO_2Ph:** 13 mg, 0.020 mmol, 1.00 equiv.), boronate **10** (5.4 mg, 0.022 mmol, 1.10 equiv.) and $CDCl_3$ (0.50 mL). The tube was removed from the glovebox and stirred for 12 h at 25 °C. The reaction was open to air, and brine (2.0 mL), followed by a freshly prepared stock solution of 1,3,5-trimethoxybenzene in $CDCl_3$ (0.080 mM, 0.25 mL, 0.020 mmol, 1.00 equiv.) was added. The organic phase was transferred to an NMR tube, and the yield was determined by quantitative ^{1}H NMR.

Quantitative ^{1}H NMR analysis of the sample indicated the formation of **[Bi-2]•Ph** in 58% (Y = $PhSO_2$), 48% (Y = I), 53% (Y = OTf) and 54% (Y = BF_4) yield.

5.3.4 Transmetalation with 11

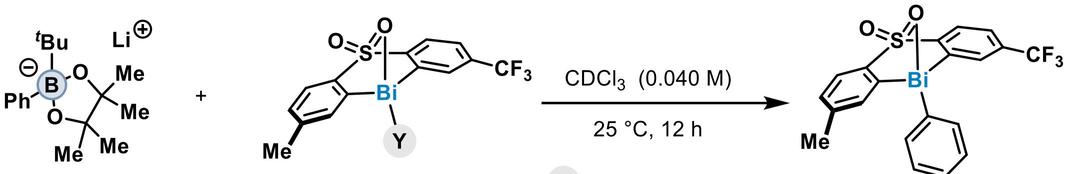

11
(1.20 equiv.)

[Bi-2]•Y
(1.00 equiv.)

[Bi-2]•Ph

In an argon-filled glovebox, an oven-dried culture tube equipped with a magnetic stir bar was charged with **[Bi-2]•Y** (**Y = Cl:** 11 mg; **OTf:** 13 mg; **SO_2Ph:** 13 mg, 0.020 mmol, 1.00 equiv.), boronate **11** (6.4 mg, 0.024 mmol, 1.20 equiv.) and $CDCl_3$ (0.50 mL). The tube was removed from the glovebox and stirred for 12 h (for Y = OTf for 10 min) at 25 °C. The reaction was open to air, and brine (2.0 mL), followed by a freshly prepared stock solution of 1,3,5-trimethoxybenzene in $CDCl_3$ (0.080 mM, 0.25 mL, 0.020 mmol, 1.00 equiv.) was added. The organic phase was transferred to an NMR tube, and the yield was determined by quantitative ^{1}H NMR.

Quantitative ^{1}H NMR analysis of the sample indicated the formation of **[Bi-2]•Ph** in 90% (Y = $PhSO_2$), 84% (Y = Cl) and 93% (Y = OTf) yield.

5.3.5 Transmetalation with 12

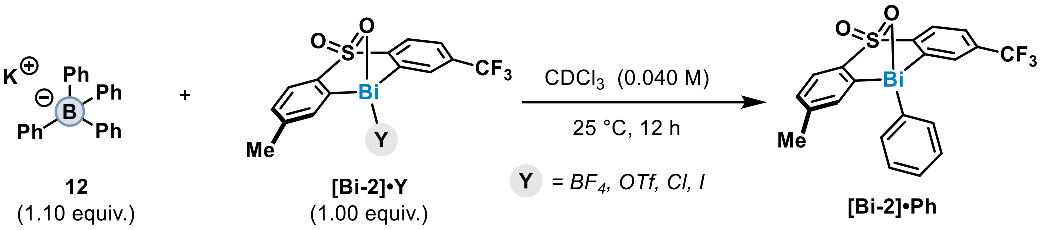

In an argon-filled glovebox, an oven-dried culture tube equipped with a magnetic stir bar was charged with **[Bi-2]•Y** (**Y = Cl:** 11 mg; **OTf:** 13 mg; **BF_4:** 12 mg, 0.020 mmol, 1.00 equiv.), boronate **12** (7.9 mg, 0.022 mmol, 1.10 equiv.) and $CDCl_3$ (0.50 mL). The tube was removed from the glovebox and stirred for 12 h (for Y = OTf for 10 min) at 25 °C. The reaction was open to air, and brine (2.0 mL), followed by a freshly prepared stock solution of 1,3,5-trimethoxybenzene in $CDCl_3$ (0.080 mM, 0.25 mL, 0.020 mmol, 1.00 equiv.) was added. The organic phase was transferred to an NMR tube, and the yield was determined by quantitative ^1H NMR.

Quantitative ^1H NMR analysis of the sample indicated the formation of **[Bi-2]•Ph** in 79% (Y = OTf), 99% (Y = BF_4) and 0% (Y = Cl) yield.

6 Effect of substitution on aryl boron nucleophiles

6.1 Kinetic monitoring of the transmetalation between 4 and [Bi-2]•OTf

6.1.1 Without additives

*i. Transmetalation with **4a***

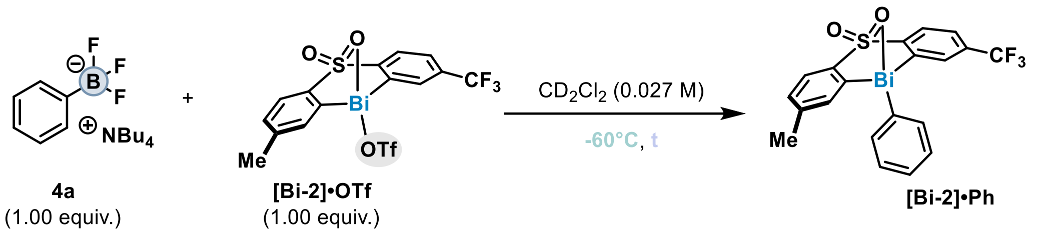

In an argon-filled glovebox, an oven-dried J. Young NMR tube was charged with **[Bi-2]•OTf** (9.8 mg, 0.015 mmol, 1.00 equiv.) and dry CD_2Cl_2 (0.50 mL). The tube was shaken until all the solids have dissolved. A separate oven-dried vial, was charged with **4a** (35 mg, 0.090 mmol, 6.00 equiv) and dry CD_2Cl_2 (0.30 mL). The J. Young NMR tube and the vial were sealed, removed from the glovebox, and immediately placed on dry ice. After 5 min and while under dry ice, the J. Young NMR tube was opened under a positive stream of Ar. **4a** in CD_2Cl_2 (0.30 M, 0.050 mL, 0.015 mmol, 1.00 equiv.) was added with a Hamilton syringe, and the tube was closed. Just before the NMR measurement, the tube was removed from the dry ice, quickly shaken and immediately transferred to the NMR probe pre-cooled to −60 °C. After fast shimming, a single scan ^{1}H NMR spectra were acquired every 5 min over the course of 7.5 h. The concentration of each component was determined relative to $[NBu_4]^+$ signal (3.25 – 2.83 ppm).

^{1}H NMR (**Figure S26**) shows an initial formation of 17% of **[Bi-2]•Ph**. After 6 h, the formation of **[Bi-2]•Ph** plateaus at 50%.

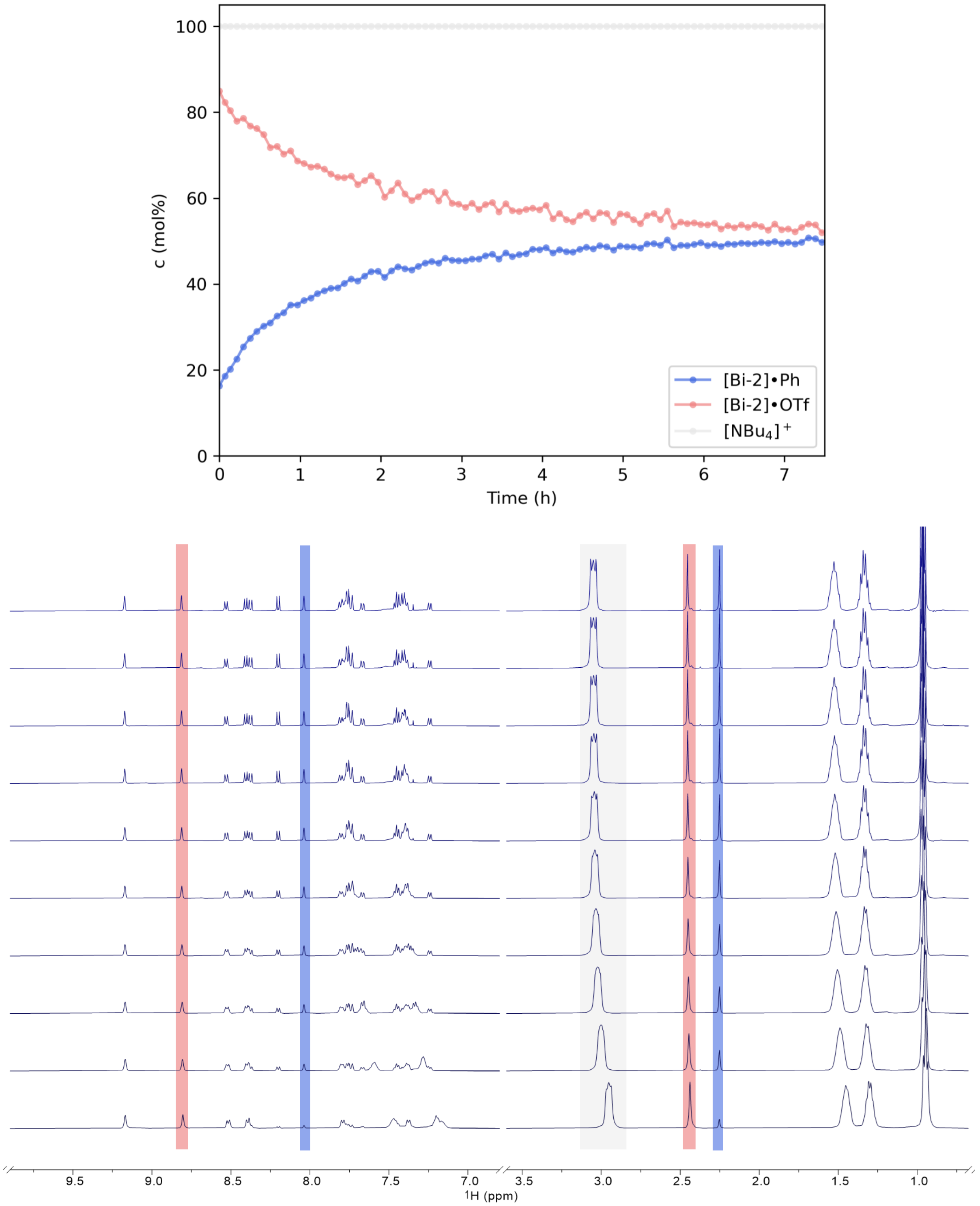

Figure S26. Time vs. concentration plot based on ^{1}H NMR showing the formation of **[Bi-2]•Ph** and disappearance of **[Bi-2]•OTf** over time using $[NBu_4]^+$ as an internal reference (top). Stacked ^{1}H NMR spectra from reaction monitoring over time at 213 K (bottom) (blue: **[Bi-2]•OTf**; pink: **[Bi-2]•Ph**; grey: $[NBu_4]^+$). t = 0 h refers to the time immediately after the acquisition of the first ^{1}H NMR spectrum.

Aside from **[Bi-2]•Ph**, we observe **[Bi-2]•OTf** in the reaction mixture which disappears at a similar rate as **[Bi-2]•Ph** is formed. Therefore we suggest the presence of a fast equilibrium between **[Bi-2]•OTf** and **[Bi-2]•PhBF3** (**Figure S27**, A), followed by the aryl transfer from B-to-Bi in **[Bi-2]•PhBF3** (**Figure S27**, B).

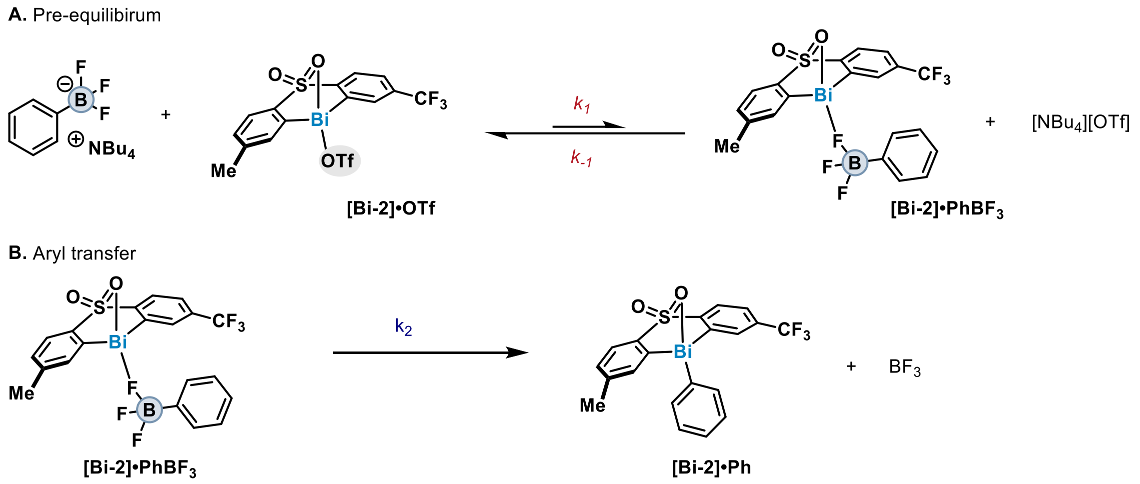

Figure S27. Postulated presence of a pre-equilibrium.

ii. *Transmetalation with **4c***

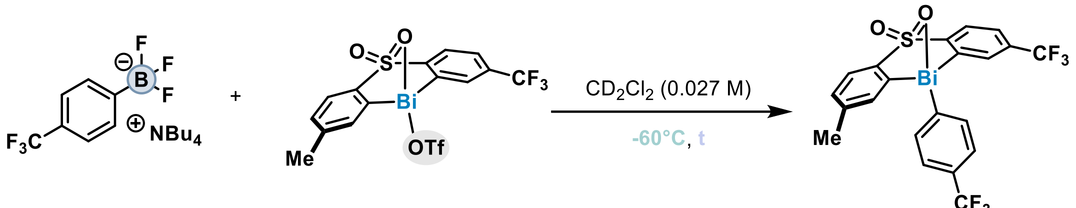

4c
(1.00 equiv.)

[Bi-2]•OTf
(1.00 equiv.)

[Bi-2]•Ar^{CF3}

In an argon-filled glovebox, an oven-dried J. Young NMR tube was charged with **[Bi-2]•OTf** (9.8 mg, 0.015 mmol, 1.00 equiv.) and dry CD_2Cl_2 (0.50 mL). The tube was shaken until all the solids have dissolved. A separate oven-dried vial, was charged with **4c** (27 mg, 0.060 mmol, 4.00 equiv) and dry CD_2Cl_2 (0.20 mL). The J. Young NMR tube and the vial were sealed, removed from the glovebox, and immediately placed on dry ice. After 5 min and while under dry ice, the J. Young NMR tube was opened under a positive stream of Ar. **4c** in CD_2Cl_2 (0.30 M, 0.050 mL, 0.015 mmol, 1.00 equiv.) was added with a Hamilton syringe, and the tube was closed. Just before the NMR measurement, the tube was removed from the dry ice, quickly shaken and immediately transferred to the NMR probe pre-cooled to −60 °C. After fast shimming, a single scan ^{1}H NMR spectra were acquired every 5 min over the course of 8.4 h however no formation of **[Bi-2]•Ar^{CF3}** was observed during this time (**Figure S28**). When the sample is heated to −20 °C the transmetalation is initiated.

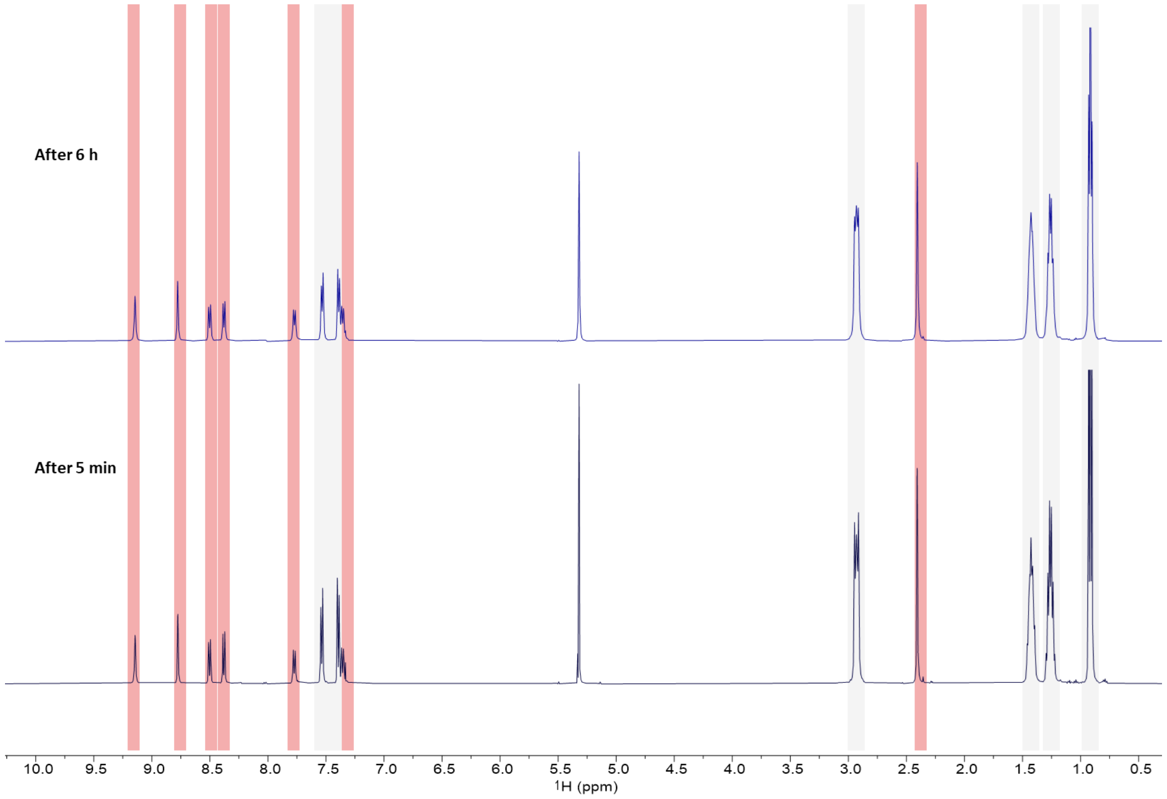

Figure S28. Stacked ^{1}H NMR spectra of the reaction between **4c** (grey) and **[Bi-2]•OTf** (pink) after 5 min and after 6 h at −60 °C.

6.1.2 With triphenylphosphine oxide

*i. Transmetalation with **4a – 4c***

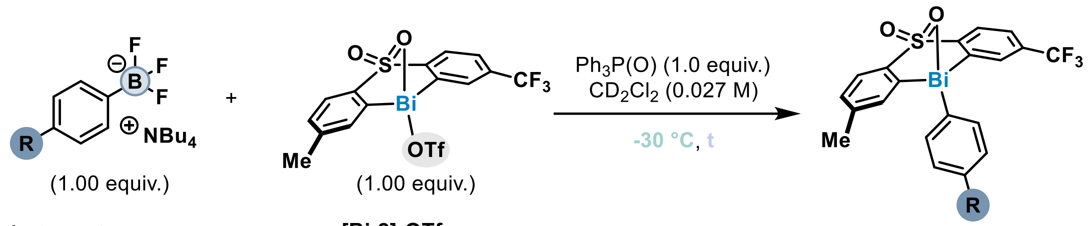

4a (R = H)
4b (R = OMe)
4c (R = CF_3)

[Bi-2]•OTf

[Bi-2]•Ph (R = H)
[Bi-2]•Ar^{OMe} (R = OMe)
[Bi-2]•Ar^{CF3} (R = CF_3)

In an argon-filled glovebox, an oven-dried J. Young NMR tube was charged with **[Bi-2]•OTf** (9.8 mg, 0.015 mmol, 1.00 equiv.), $Ph_3P(O)$ (4.2 mg, 0.015 mmol, 1.00 equiv.) and dry CD_2Cl_2 (0.50 mL). The tube was shaken until all the solids have dissolved. A separate oven-dried vial, was charged with **4a**, **4b** or **4c** (**4a:** 35 mg; **4b:** 38 mg; **4c:** 41 mg, 0.090 mmol, 6.00 equiv) and dry CD_2Cl_2 (0.30 mL). The J. Young NMR tube and the vial were sealed, removed from the glovebox, and immediately placed on dry ice. After 5 min and while under dry ice, the J. Young NMR tube was opened under a positive stream of Ar. The stock solution of **4** in CD_2Cl_2 (0.30 M, 0.050 mL, 0.015 mmol, 1.00 equiv.) was added with a Hamilton syringe, and the tube was closed. Just before the NMR measurement, the tube was removed from the dry ice, quickly shaken and immediately transferred to the NMR probe pre-cooled to −30 °C. After fast shimming, a single scan ^{1}H NMR spectra were acquired.

Note: The reaction temperature of −30 °C for this reaction was determined following the same procedure using model substrate **4a**. Hereby the sample was gradually heated from −60 °C to 25 °C. The transmetalation initiated at −30 °C.

^{19}F NMR spectra were obtained for the indicated time. The concentration of each component was determined by the integration of signal corresponding to the CF_3 group on the ligand backbone relative to $[OTf]^-$ signal (−78.55 – −79.44 ppm).

ii. Comparison of the kinetic reaction profiles

The kinetic profiles shows clear differences depending on the subsitutents on $[ArBF_3]NBu_4$:

i. Starting from **4a** (R = H), the initial product formation is 4%. After 6 h a plateau is reached at 51%.

ii. Starting from **4b** (R = OMe), the initial conversion to product is 49%. After 0.33 h a plateau is reached at 53%.

iii. Starting from **4c** (R = CF_3), no reaction is observed. The reaction was left at the same temperature for 60 h in total, but no conversion to product was observed.

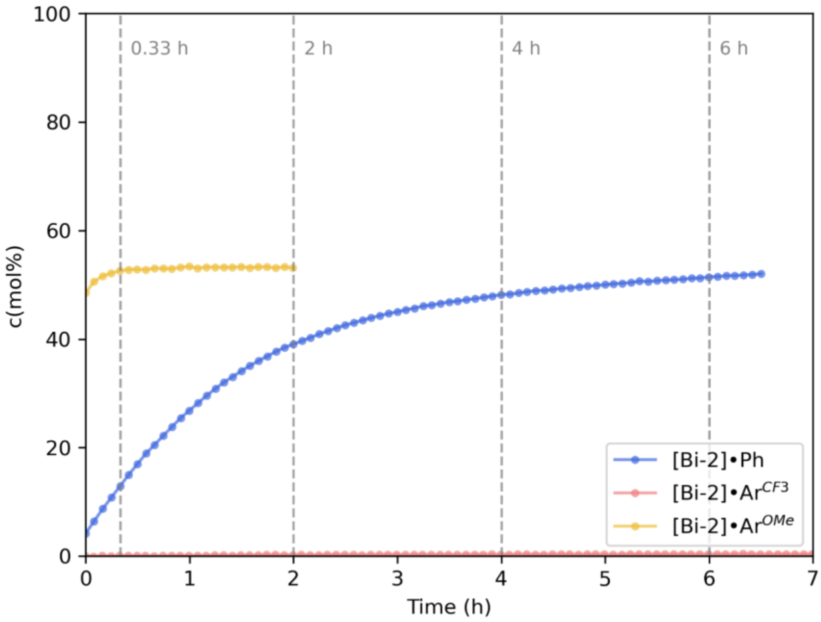

Time (in h)	Yield of [Bi-2]•Ar^{OMe} (in %)	Yield of [Bi-2]•Ph (in %)	Yield of [Bi-2]•Ar^{CF3} (in %)[a]
**0**	49	4	0
**0.33**	53	13	0
**2.00**	53	39	0
**4.00**	---	48	0
**6.00**	---	51	0

Figure S29. Conversion to product at selected time points starting from borane **4a – 4c**. The table below show conversions at different time points. The kinetic data were abstracted from the $^{19}F\{^1H\}$ NMR using $[OTf]^-$ as an internal reference. t = 0 refers to the time immediately after the acquisition of the first ^{19}F NMR spectrum. [a]**Note:** The data was consolidated using ^1H NMR, which did not show any formation of **[Bi-2]•Ar^{CF3}**.

6.2 Kinetic monitoring of the reaction between 7 and [Bi-2]•F

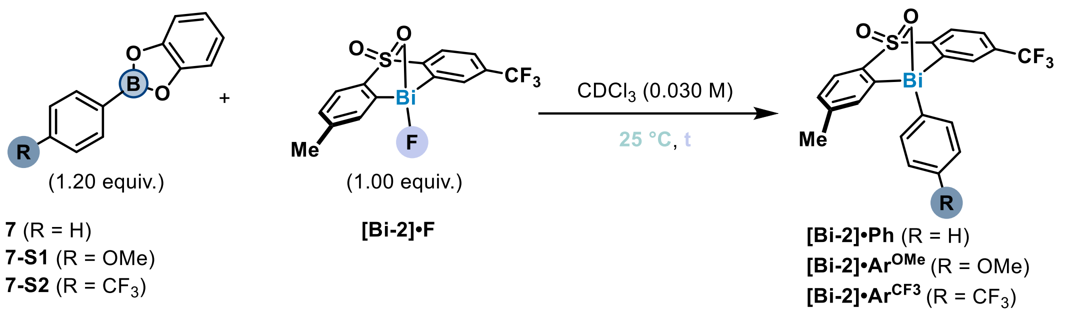

In an argon-filled glovebox, a 2-mL volumetric flask was charged with 1,3,5-trimethoxybenzene (10 mg, 0.060 mmol, 4.00 equiv.) and **[Bi-2]•F** (32 mg, 0.060 mmol, 4.00 equiv.). CD_2Cl_2 was added to afford a 0.030 M stock solution relative to **[Bi-2]•F**. An oven-dried NMR tube was charged with **7**, **7-S1** or **7-S2** (**7:** 3.5 mg; **7-S1:** 4.1 mg; **7-S2:** 4.7 mg, 0.018 mmol, 1.20 equiv.) and the freshly-prepared stock solution containing **[Bi-2]•F** and 1,3,5-trimethoxybenzene (0.030 M relative to **[Bi-2]•F**, 0.50 mL, 0.015 mmol, 1.00 equiv.). The NMR tube was shaken until all the solids have dissolved and analyzed by quantitative ^{1}H NMR.

^{1}H NMR spectra were recorded at the indicated times (**Figure S30**). The concentration of each component was determined relative to 1,3,5-trimethoxybenzene (6.08 – 6.15 ppm).

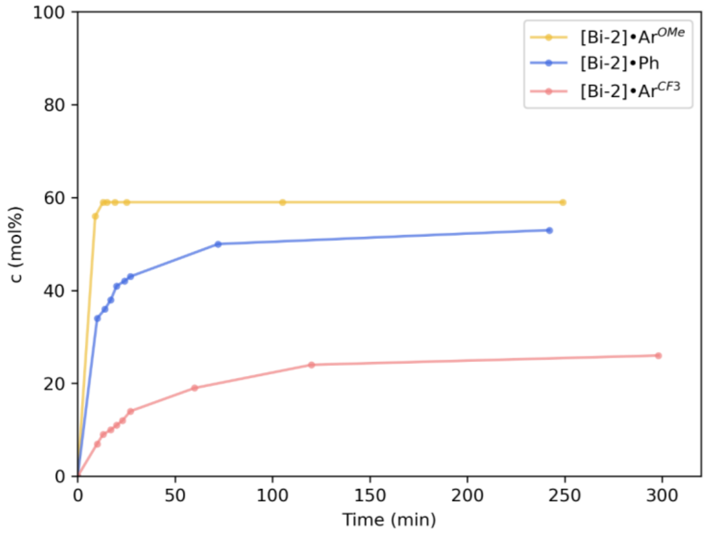

From **7-S2**		From **7-S1**		From **7**	
**Time (in min)**	Yield of **[Bi-2]•Ar^{CF3}** (in %)	**Time (in min)**	Yield of **[Bi-2]•Ar^{OMe}** (in %)	**Time (in min)**	Yield of **[Bi-2]•Ph** (in %)
**0**	0	**0**	0	**0**	0
**10**	7	**9**	56	**10**	34
**13**	9	**13**	59	**14**	36
**17**	10	**15**	59	**17**	38
**20**	11	**19**	59	**20**	41
**23**	12	**25**	59	**24**	42
**27**	14	**105**	59	**27**	43
**60**	19	**249**	59	**72**	50
**120**	24	**--**	--	**242**	53
**298**	26	**--**	--	**--**	--

Figure S30. Conversion to **[Bi-2]•Ar** at selected time points starting from aryl catechol boronic esters. The table below show conversions at different time points. t = 0 refers to the time after the stock solution was added to the NMR tube.

7 Computational details

7.1 General procedure

All quantum chemical calculations were carried out using the ORCA 5.0[14,15] and ORCA 6.0[16]. Geometries of intermediates and transition states were optimized using the PBE0 density functional[17,18] with the D3BJ[19,20] dispersion correction and the Def2-SVP[21] basis set and in chloroform solvent with the CPCM solvation model.[22,23] The relativistic effective core potential of Bi was represented using Def2-ECP (Bi).[24] Vibrational frequency calculations were performed for all stationary points to confirm if each optimized structure is a local minimum or a transition state structure. All optimized transition state structures have only one imaginary (negative) frequency, and all minima (reactants, products, and intermediates) have no imaginary frequency. For transition state structures, intrinsic reaction coordinate (IRC) calculations were carried out to ensure transit from reactants to products. The PBE0 functional with the D3BJ correction, the Def2-TZVP basis set, and Def2-ECP (Bi) was used for single-point energy calculations. The reported Gibbs free energies were calculated at the standard conditions (298 K, 1 atm). Cartesian coordinates for stationary points and the imaginary frequencies are given in section 7.5.

We investigated the reaction between **[Bi-1]•F** and three different aryl boron reagents – difluorophenyl borane (**3**), tris(pentafluorophenyl)borane (**5**) and phenylboronic acid (**1-S1**).

1) For simplicity, the unsubstituted diarylsulfone was used as model ancillary ligand for bismuth (**[Bi-1]**).
2) Only the neutral pathways were considered.

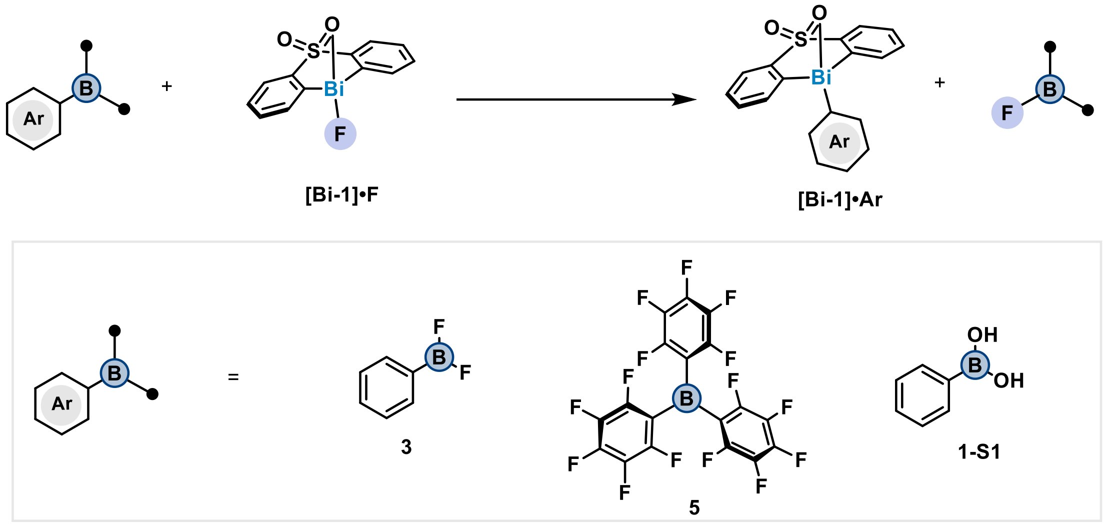

7.2 Reaction with difluoro(phenyl)borane

Starting from $PhBF_2$ (**3**) and **[Bi-1]•F**, we found **TS-1A – C** to consider for analysis (**Figure S31**). Transmetalation where the BF_3 group points away from Bi (**TS-1A**) exhibits a higher energy barrier (21.0 kcal·mol^{-1}). When the BF_3 group points toward the Bi center (**TS-1C**) the energy barrier is lower (12.7 kcal·mol^{-1}). A cyclic transition state **TS-1B** which has a notably shorter Bi-F bond (Bi-F bond lengths for **TS-1B:** 2.872 Å; for **TS-1A:** 3.198 Å) was also found. The energy for **TS-1B** is only slightly higher than for **TS-1A** ($\Delta\Delta G^{\ddagger}$ = 1.4 kcal·mol^{-1}) and therefore both transition states may be considered. Applying IRC to **TS-1A** and **TS-1C** allowed us to find the intermediates **Int-1A** and Wheland-type intermediate **Int-1B** whose formation is slightly exergonic when compared to the starting material.

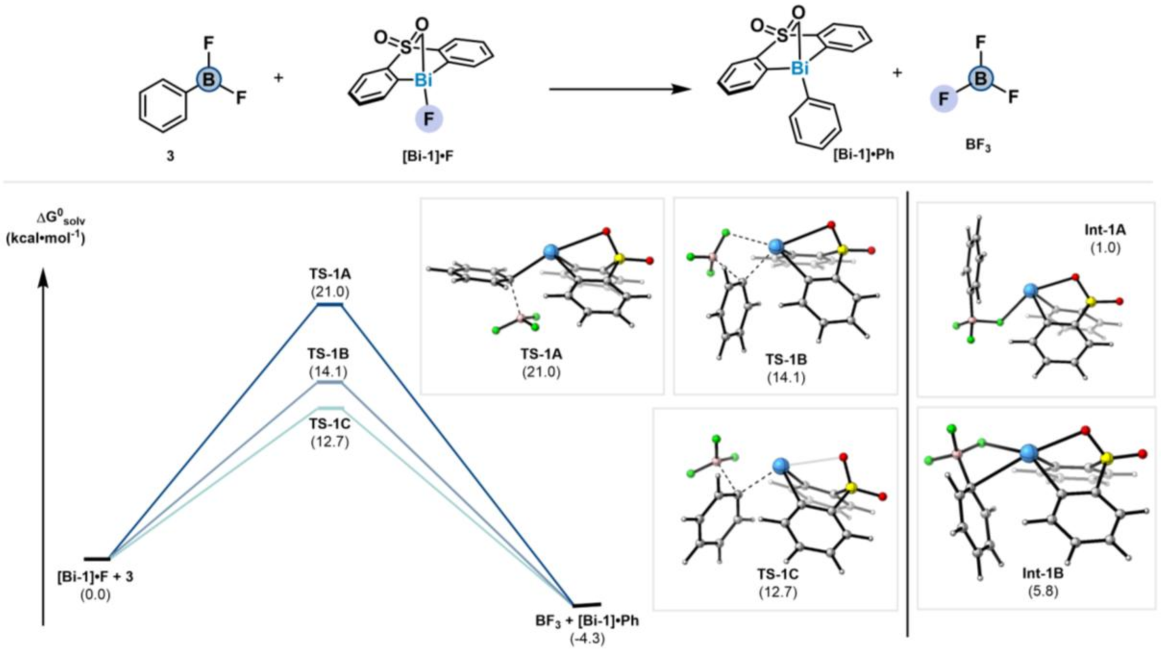

Figure S31. Reaction energy profile of the reaction between **3** and **[Bi-1]•F** at the CPCM(chloroform)-PBE0-D3BJ/def2-TZVP//CPCM(chloroform)-PBE0-D3BJ/def2-SVP level of theory. Possible pre-transmetalation complexes were calculated (on the right).

Next, Hirshfeld charges[25] of **TS-1C** were calculated. Hereby, positive Hirshfeld charge at Bi (0.414) was calculated while C(ipso) has the lowest Hirshfeld (−0.094) among all carbon atoms in **TS-1C** (**Figure S32**).

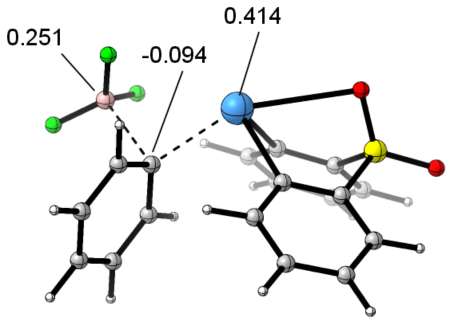

Figure S32. Hirshfeld charges of transition state **TS-1C**.

7.3 Reaction with tris(pentafluoro)borane

The energies of the pre-transmetalation complex formation from BCF (**5**) and **[Bi]•F** bearing different chemical groups (Y) in the ligand backbone were calculated. Hereby three Bi complexes were considered: **[Bi-1]•F** (Y = O), **[Bi-3]•F** (Y = NCH_3) or **[Bi-4]•F** (Y = NCF_3) (**Figure S33**). Comparing the energies for the formation of the pre-transmetalation complex, we found that its formation become more exergonic for E = O < NCF_3 < NCH_3. The Hirshfeld charges[25] (for **Int-2A** (E = O): 0.605; for **Int-2B** (E = NCF_3): 0.579; for **Int-2C** (E = NMe): 0.552)) and natural charges derived from Natural Poulation Analysis[26] (for **Int-2A** (E = O): 1.571; for **Int-2B** (E = NCF_3): 1.538; for **Int-2C** (E= NMe): 1.487) decrease in the same order. This aligns to our hypothesis that the cationic character of Bi is reduced through hypervalent N-coordination when compared to O-coordination, and provide a possible explanation on why **[Bi-3]•B(Ar^F)$_3$F** (**Int-2C**) is less reactive than **[Bi-2]•B(Ar^F)$_3$F** (**Int-2A**) toward transmetalation.

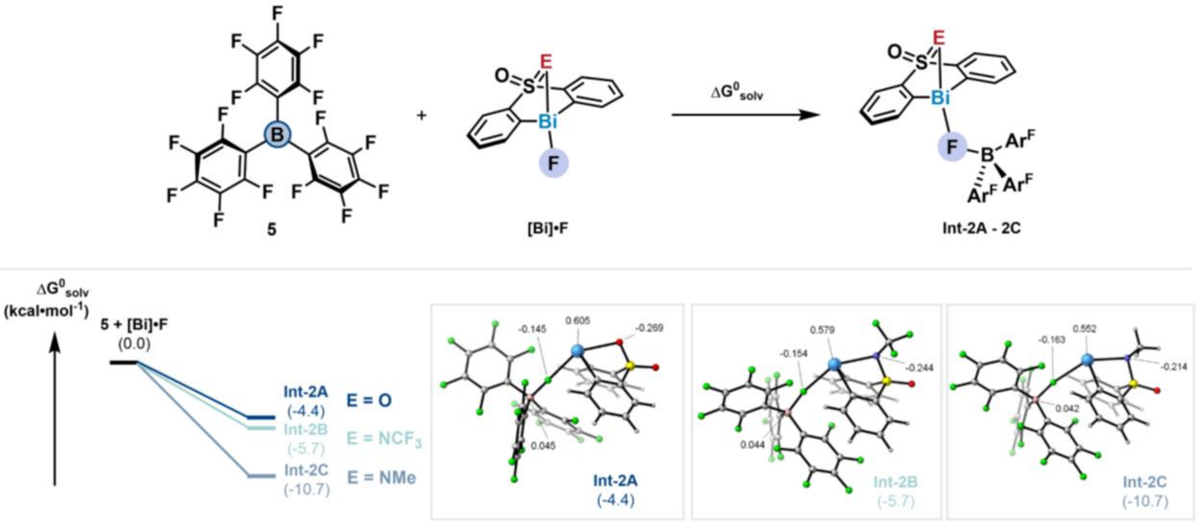

Figure S33. Stabilization energies for the formation of pre-transmetalation complexes formed from **5** and **[Bi]•F** at the CPCM(chloroform)-PBE0-D3BJ/def2-TZVP//CPCM(chloroform)-PBE0-D3BJ/def2-SVP level of theory. Hirshfeld charges are depicted next to the atoms.

Comparing fluoride-bridge intermediate **Int-2A,** hydroxide-bridged intermediate **Int-3A** and triarylated bismuth **[Bi-1]•Ar^F** revealed that the Hirshfeld charges[25] and natural charges derived from Natural Population analysis[26] on Bi increase in the following order: **[Bi-1]•Ar^F** << **Int-3A** < **Int-2A**. With increasing charge density on Bi, the transannular Bi-O bond distance decreases which is consistent with related solid state structures (section 8.3).

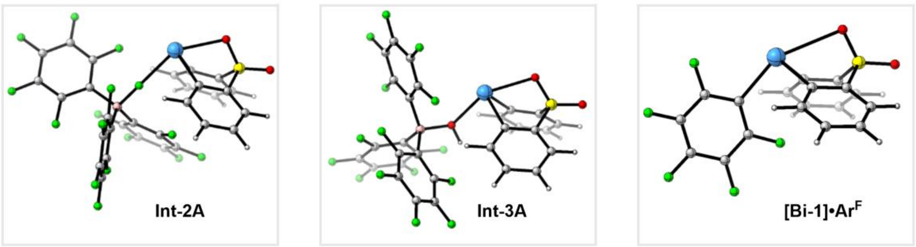

	Int-2A	Int-3A	[Bi-1]•Ar^F
Hirshfeld charge of Bi	0.605	0.599	0.411
Natural charge of Bi	1.571	1.525	1.217
Transannular Bi-O distance	2.411	2.495	2.876

Figure S34. Comparison of calculated Hirshfeld charges, natural charges and Bi-O bond distances in **Int-2A**, **Int-3A** and **[Bi-1]•Ar^F** at the CPCM(chloroform)-PBE0-D3BJ/def2-TZVP//CPCM(chloroform)-PBE0-D3BJ/def2-SVP level of theory.

The energy barrier for the transmetalation from **Int-2A** was calculated to be 19.8 kcal·mol^{-1} (**Figure S35**). The overall reaction was exothermic (ΔG = −5.4 kcal·mol^{-1}). In comparison, the transition state energy for the aryl transfer from hydroxide bound pre-transmetalation complex **Int-3A** was 23.9 kcal·mol^{-1}. The reaction is also exothermic (ΔG = −8.4 kcal·mol^{-1}).

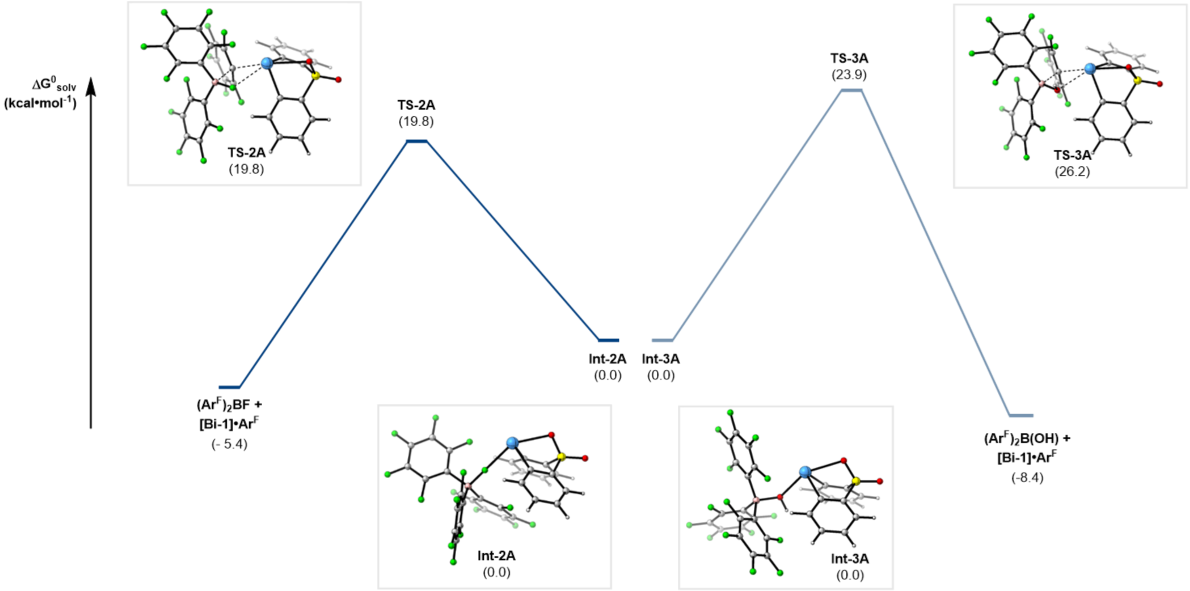

Figure S35. Reaction energy profile of transmetalation from fluoride-bridged pre-transmetalation complex Int-2A (dark blue) and hydroxide-bridged pre-transmetalation complex **Int-3A** (steel blue) at the CPCM(chloroform)-PBE0-D3BJ/def2-TZVP//CPCM(chloroform)-PBE0-D3BJ/def2-SVP level of theory. For better comparison of the energies of the transition states, the energies of each pre-transmetalation intermediates **Int-2A** and **Int-3A** were set to 0.0 kcal·mol^{-1}.

7.4 Reaction with phenylboronic acid

The reaction profile using phenylboronic acid (**1-S1**) and **[Bi-1]•F** was also calculated (**Figure S36**). We found two transition states with an oxygen-bridge (**TS-4A**) or a fluoride bridge (**TS-4B**). **TS-4A** is slightly lower in energy ($\Delta\Delta G^{\ddagger}$ = 3.1 kcal mol^{-1}). Applying IRC allowed us to identify an F-bound (**Int-4A**) and an O-bound (**Int-4B)** pre-transmetalation complex that are directly linked to the transition states however their formation in comparison to dissociated **[Bi-1]•F** and **1-S1** is highly endergonic (ΔG = 20.5 kcal mol^{-1} for **Int-4A**; and ΔG = 25.2 kcal mol^{-1} for **Int-4B**). Searching for pre-transmetalation complexes with lower energy was successful (e.g. F-bound intermediate **Int-4C**) however their formation was still endergonic (ΔG = 8.8 kcal mol^{-1}).

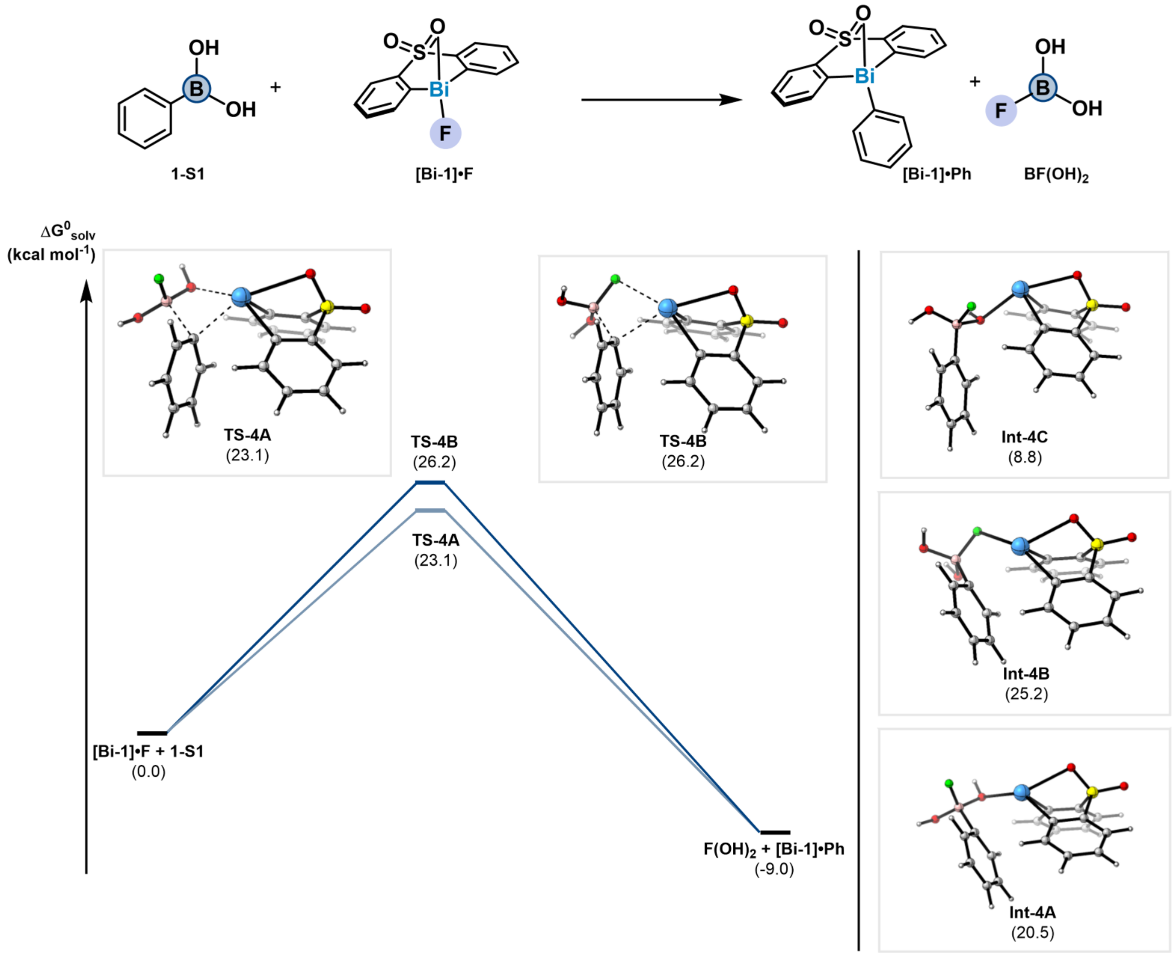

Figure S36. Reaction energy profile of the transmetalation from **1-S1** and **[Bi-1]•F** comparing hydroxide-bridged (steel-blue) and fluoride bridged (dark blue) pathways at the CPCM(chloroform)-PBE0-D3BJ/def2-TZVP//CPCM(chloroform)-PBE0-D3BJ/def2-SVPlevel of theory. Possible pre-transmetalation complexes were calculated (on the right).

7.5 Cartesian coordinates

[Bi-1]•F

Bi 6.500742 5.785154 4.961044
S 5.503979 6.677331 1.990142
F 5.384397 5.358982 6.673536
O 6.918468 6.521768 2.431580
O 5.207558 7.087607 0.621031
C 5.126537 7.553015 4.504458
C 4.787130 7.791740 3.173513
C 3.907735 8.792747 2.775138
H 3.669333 8.932554 1.718385
C 3.350505 9.599007 3.768611
H 2.663548 10.402241 3.493025
C 3.660509 9.373077 5.110946
C 4.538557 8.349283 5.483764
H 4.750651 8.168527 6.541090
C 5.074765 4.584293 3.631109
C 4.743746 5.119503 2.385993
C 3.850635 4.517882 1.505985
H 3.621924 4.983406 0.544654
C 3.269042 3.310756 1.895961
H 2.571031 2.803781 1.225954
C 3.571644 2.757301 3.141100
C 4.464973 3.391614 4.012140
H 4.673425 2.952922 4.991900
H 3.100834 1.818234 3.443205
H 3.206057 9.999660 5.882688

$PhBF_2$ (3)

C 1.233684 5.585286 -2.996023
C -0.099024 6.013462 -3.119296
C 1.865777 5.693109 -1.745688
C -0.782215 6.533204 -2.022915
C 1.186072 6.214846 -0.648249
C -0.138734 6.633556 -0.787694
H -0.603889 5.936377 -4.085911
H 2.902389 5.363928 -1.635149
H -1.819062 6.861729 -2.128158
H 1.686931 6.295107 0.319578
H -0.674068 7.041509 0.073706
B 1.991152 5.001091 -4.208693
F 3.237498 4.561447 -4.108645
F 1.433623 4.906168 -5.407226

Int-1A

Bi 7.539535 7.181266 3.132135
S 4.721570 7.165888 1.734430
O 6.088633 7.547807 1.219385
O 3.575094 7.257678 0.843702
C 5.699640 8.273900 3.968342
C 4.548585 8.168737 3.187823
C 3.333772 8.758935 3.515855
H 2.462179 8.639144 2.868352
C 3.281609 9.507285 4.692561
H 2.349426 9.997640 4.981460
C 4.414901 9.627038 5.498739
C 5.622406 9.012260 5.145845
H 6.493480 9.112244 5.798622
C 6.171797 5.349952 3.061023
C 4.973126 5.531634 2.372061
C 4.002314 4.545464 2.239766
H 3.076818 4.743367 1.694446
C 4.263100 3.306357 2.825603
H 3.531811 2.500217 2.734997
C 5.448473 3.100256 3.533260
C 6.404441 4.115399 3.660549
H 7.311690 3.939099 4.243617
H 5.634127 2.130874 4.002427
H 4.359572 10.209495 6.421836
C 10.779046 7.386885 4.270457
C 10.173768 6.127030 4.423710
C 10.251192 5.230896 3.344345
C 11.402778 7.752151 3.076965
C 10.873284 5.587919 2.145170
C 11.439919 6.855771 2.006149
H 10.741534 8.102219 5.097883

H 9.797646 4.239948 3.441232

H 11.855919 8.741887 2.975213

H 10.910816 4.878072 1.314635

H 11.916516 7.144076 1.065655

B 9.232663 5.818555 5.688850

F 7.908231 6.521069 5.349155

F 8.898501 4.480097 5.830242

F 9.633504 6.376631 6.881448

Int-1B

H -1.613193 -1.899373 -2.129311

H -3.745894 -0.767151 -2.683247

C -2.201882 -1.434736 -1.336932

C -3.413500 -0.804970 -1.642886

Bi 0.119916 -2.538571 0.785042

F 1.748398 -4.634880 -1.132586

C -1.766580 -1.494090 -0.014991

C -4.205681 -0.237619 -0.644331

H -5.152222 0.243045 -0.900607

C -2.591135 -0.933236 0.961326

O -1.492216 -2.539782 2.633679

C -3.798392 -0.300633 0.687921

C 0.527238 -0.616830 2.017344

H 2.623239 -0.247645 1.631903

S -2.031346 -1.131640 2.626801

C 1.725113 0.081806 2.154545

H -4.403751 0.112911 1.497464

C -0.566634 -0.148638 2.748966

O -3.040360 -0.753824 3.605092

C 1.788084 1.215942 2.973117

C -0.539236 0.974503 3.567275

H 2.735226 1.752676 3.069224

C 0.666931 1.667229 3.669517

H -1.431762 1.283764 4.115911

H 0.732982 2.552175 4.306191

B 1.280557 -3.558409 -1.897398

C 1.874429 -2.142992 -1.300480

C 3.153107 -2.131380 -0.690571

C 1.316005 -0.890599 -1.647047

C 3.854565 -0.947937 -0.496272

C 3.280359 0.273923 -0.869126

C 2.003931 0.304273 -1.424620

H 3.603322 -3.085811 -0.403692

H 4.852804 -0.967550 -0.051470

H 3.830045 1.205365 -0.711355

H 1.548235 1.257241 -1.703562

H 0.341376 -0.855366 -2.140236

F 1.634970 -3.711826 -3.231287

F -0.150473 -3.545185 -1.802360

TS-1A

Imaginary frequency: -186.86 cm^{-1}

Bi 6.781247 7.650899 2.325624

S 3.938181 6.222436 3.227890

O 4.120601 6.798454 1.873740

O 2.662378 5.604237 3.585239

C 5.441696 8.324962 4.063177

C 4.310288 7.553976 4.339618

C 3.420586 7.836601 5.372640

H 2.556432 7.190060 5.539242

C 3.660963 8.962508 6.157368

H 2.980706 9.212579 6.974437

C 4.761872 9.773763 5.883513

C 5.645255 9.459906 4.847344

H 6.501814 10.113194 4.677858

C 6.543767 5.474799 3.021801

C 5.266148 5.057376 3.403974

C 4.972711 3.759924 3.814513

H 3.952623 3.496029 4.101557

C 6.004302 2.823164 3.824373

H 5.803820 1.794578 4.132300

C 7.285390 3.201545 3.424327

C 7.555574 4.514736 3.029344

H 8.573788 4.770936 2.733729

H 4.944019 10.664527 6.489825

H 8.092990 2.465219 3.420086

C 9.972028 7.427651 2.574542

C 8.940917 8.171144 3.203737
C 9.144533 9.570308 3.314813
C 11.156318 8.022979 2.158630
C 10.325831 10.174416 2.903273
C 11.333683 9.398104 2.326746
H 9.861936 6.347145 2.449116
H 8.374973 10.195145 3.776274
H 11.945352 7.419135 1.704339
H 10.466784 11.250150 3.030908
H 12.265818 9.869255 2.003887
B 9.008472 7.532696 5.099070
F 7.998182 8.169902 5.735851
F 8.880620 6.187573 5.014848
F 10.244925 7.955138 5.465483

TS-1B

Imaginary frequency: -220.69 cm^{-1}

H -1.482073 -2.161568 -1.983531
H -3.567097 -1.060478 -2.685125
C -2.110242 -1.690404 -1.226319
C -3.293831 -1.060536 -1.626959
Bi 0.163582 -2.674334 0.942604
F 0.160531 -4.054195 -1.576489
C -1.739209 -1.702009 0.118590
C -4.123243 -0.435468 -0.696974
H -5.046239 0.050959 -1.020220
C -2.594165 -1.071653 1.027635
O -1.783526 -2.544570 3.007141
C -3.773665 -0.435691 0.652803
C 0.451479 -0.740674 2.158034
H 2.536151 -0.388987 1.692679
S -2.158323 -1.134712 2.751141
C 1.651283 -0.031705 2.226933
H -4.400679 0.039732 1.410161
C -0.644143 -0.213488 2.843388
O -3.196468 -0.499754 3.562085
C 1.731027 1.151924 2.967306
C -0.597586 0.968606 3.577290
H 2.679002 1.694105 3.012654
C 0.615342 1.652677 3.640085
H -1.492523 1.333462 4.086025
H 0.688443 2.579463 4.213545
B 1.057284 -3.260478 -2.207778
C 1.591828 -1.770840 -0.679724
C 2.950168 -2.049975 -0.410500
C 1.281548 -0.503924 -1.218615
C 3.955549 -1.137295 -0.719310
C 3.616516 0.096813 -1.276559
C 2.278827 0.415055 -1.525794
H 3.230204 -3.014203 0.027122
H 5.001546 -1.383722 -0.522015
H 4.401043 0.819605 -1.515539
H 2.017265 1.385128 -1.955211
H 0.238519 -0.239371 -1.414622
F 2.252365 -3.809435 -2.445993
F 0.552183 -2.452560 -3.149818

TS-1C

Imaginary frequency: -163.40 cm^{-1}

H -1.594751 -2.046525 -2.021512
H -3.650757 -0.853865 -2.680912
C -2.193931 -1.554298 -1.253760
C -3.362665 -0.880751 -1.626973
Bi 0.078829 -2.651995 0.878190
F 1.879869 -4.387925 -1.108715
C -1.802182 -1.599386 0.085220
C -4.160654 -0.248038 -0.675084
H -5.071372 0.274299 -0.976490
C -2.634175 -0.971693 1.017079
O -1.892505 -2.554492 2.935595
C -3.798901 -0.293688 0.670384
C 0.415935 -0.789002 2.182231
H 2.518595 -0.489705 1.765968
S -2.213539 -1.122638 2.737570
C 1.639542 -0.132051 2.308197
H -4.406196 0.176655 1.446711

C -0.668830 -0.263072 2.886598
O -3.237601 -0.486022 3.564943
C 1.754883 1.000617 3.119969
C -0.585813 0.869353 3.691993
H 2.723031 1.500084 3.208253
C 0.650710 1.502016 3.809988
H -1.472661 1.235028 4.214041
H 0.751451 2.387780 4.441081
B 1.435808 -3.411825 -1.929077
C 1.569100 -1.738216 -0.688814
C 2.933776 -1.864774 -0.342895
C 1.188130 -0.622517 -1.464811
C 3.883942 -0.960294 -0.807471
C 3.480716 0.113424 -1.602159
C 2.132580 0.286305 -1.927336
H 3.262717 -2.716366 0.261386
H 4.938451 -1.092912 -0.554644
H 4.224090 0.823564 -1.973587
H 1.822139 1.132926 -2.544213
H 0.138002 -0.476055 -1.730191
F 2.243025 -3.063246 -2.940002
F 0.112598 -3.463436 -2.215040

BF_3

B 1.929087 0.069995 -1.486984
F 0.938999 -0.488929 -2.140458
F 1.929067 0.069985 -0.175923
F 2.919166 0.628913 -2.140475

[Bi-1]•Ph

Bi 0.840696 3.492039 3.604899
S 4.125581 3.499325 4.445320
O 5.557246 3.502073 4.754636
O 3.140335 3.504925 5.543218
C 2.397928 5.061965 3.028306
C 2.087310 6.178571 2.250952
C 3.083615 7.092618 1.894937
C 4.405476 6.899708 2.298438
C 4.740995 5.786226 3.066942
H 5.765869 5.602235 3.396262
C 3.728380 4.897202 3.419023
C -0.015146 3.480163 1.504524
C -1.405469 3.479811 1.346597
H -2.064271 3.483837 2.221587
C -1.975642 3.474516 0.070116
H -3.063368 3.474409 -0.041087
C -1.156646 3.469565 -1.058160
H -1.600297 3.465594 -2.057109
C 0.232248 3.469786 -0.909313
H 0.877155 3.465962 -1.792316
C 0.799546 3.475077 0.365136
H 1.889101 3.475452 0.467271
C 2.400353 1.918152 3.045849
C 2.091959 0.793596 2.279098
C 3.090308 -0.121116 1.930686
C 4.411970 0.078778 2.331441
C 4.745218 1.200029 3.089552
H 5.769880 1.389658 3.416311
C 3.730654 2.089836 3.434108
H 1.062394 6.341084 1.905208
H 5.178462 7.617168 2.013954
H 1.067263 0.625400 1.935429
H 5.186536 -0.639261 2.052767
H 2.831675 -0.998208 1.331842
H 2.823174 7.963661 1.288117

[Bi-4]•F

Bi 6.600092 5.976678 4.863579
S 5.427387 6.947870 1.912137
F 5.498757 5.500346 6.579265
O 5.008871 7.382707 0.577802
C 5.151863 7.688209 4.501555
C 4.759877 7.967151 3.195650
C 3.831415 8.945235 2.856968
H 3.555295 9.114622 1.814007
C 3.279406 9.692729 3.897746
H 2.556139 10.479405 3.673067

C 3.641522 9.430230 5.219927

C 4.566602 8.426826 5.526423

H 4.819255 8.212541 6.568195

C 5.226133 4.752302 3.484076

C 4.812032 5.312575 2.275851

C 3.974989 4.672239 1.369733

H 3.688361 5.162958 0.437070

C 3.535808 3.388120 1.693014

H 2.889997 2.845753 0.998931

C 3.921187 2.800477 2.898534

C 4.754097 3.478876 3.795982

H 5.032223 3.002449 4.740286

H 3.568567 1.796036 3.146161

H 3.190817 10.010958 6.028600

N 6.956022 6.930708 2.334070

C 7.884698 6.302921 1.507775

F 8.222334 7.041515 0.441623

F 9.001750 6.079343 2.204907

F 7.487842 5.109583 1.015445

[Bi-3]•F

Bi 6.655405 6.076202 4.794209

S 5.470695 6.985685 1.917632

F 5.602588 5.574362 6.556688

O 5.043781 7.401344 0.572472

C 5.139503 7.733323 4.490397

C 4.748032 7.994490 3.181413

C 3.786271 8.938836 2.839579

H 3.508213 9.095347 1.795085

C 3.203635 9.667721 3.877201

H 2.453584 10.428187 3.649366

C 3.567956 9.419754 5.202412

C 4.525885 8.449579 5.514301

H 4.781327 8.239916 6.556342

C 5.274471 4.790525 3.475092

C 4.844581 5.342055 2.268853

C 4.005556 4.683229 1.377168

H 3.704437 5.165764 0.444570

C 3.577502 3.399011 1.716599

H 2.927472 2.845347 1.035131

C 3.977609 2.825039 2.924792

C 4.815527 3.517095 3.807002

H 5.106055 3.054100 4.754431

H 3.631785 1.821366 3.185566

H 3.091721 9.984843 6.007758

N 6.981977 6.980365 2.351655

C 7.856572 6.157957 1.520416

H 8.867594 6.200840 1.948302

H 7.551646 5.094457 1.473562

H 7.913586 6.538898 0.487378

BCF (5)

C 1.318713 7.642824 4.116352

C 0.180928 8.049822 4.816894

C 2.418929 7.246983 4.880496

C 0.122157 8.053384 6.206004

C 2.405804 7.266526 6.270424

C 1.246666 7.666817 6.932599

B 1.357427 7.646442 2.549512

C 1.921462 6.406906 1.784499

C 2.638829 6.536536 0.587608

C 1.757405 5.101528 2.268777

C 3.177057 5.446960 -0.085847

C 2.264713 3.988132 1.608673

C 2.981507 4.166538 0.427728

C 0.840987 8.906711 1.780807

C 1.077109 10.201848 2.257991

C 0.121691 8.806538 0.584050

C 0.643750 11.337906 1.583135

C -0.344566 9.919874 -0.104208

C -0.071915 11.191439 0.397674

F 1.769488 10.392077 3.372475

F 0.902140 12.546826 2.050092

F -0.488841 12.257648 -0.251665

F -1.036773 9.789564 -1.222127

F -0.159313 7.615425 0.073298

F -0.909697 8.419990 4.156236

F -0.980136 8.415972 6.839149

F 1.214733 7.677815 8.249548
F 3.472849 6.914028 6.966571
F 3.541282 6.863602 4.283723
F 2.857398 7.733305 0.062011
F 3.868480 5.611017 -1.199438
F 3.475346 3.123562 -0.203844
F 2.078294 2.770482 2.086639
F 1.072068 4.876804 3.380436

Int-2A

Bi 0.848385 3.928553 4.526566
S 3.908765 3.595992 3.943188
O 5.360942 3.536272 3.971999
O 3.163470 4.003803 5.197615
C 1.950084 5.091501 2.902207
C 1.427686 6.096307 2.093883
C 2.252048 6.712368 1.145150
C 3.584546 6.327158 0.987323
C 4.131560 5.338906 1.806377
H 5.178719 5.038967 1.727364
C 3.296235 4.760577 2.754843
C -0.614658 4.040529 0.526401
C -0.884460 5.174651 -0.234588
C 0.018734 5.700686 -1.157486
C 1.249098 5.082444 -1.342156
C 1.555796 3.937954 -0.614837
C 0.620722 3.439413 0.283618
C 1.789816 1.991123 3.784964
C 1.122719 0.796853 3.531150
C 1.839579 -0.296408 3.035527
C 3.211086 -0.207684 2.785577
C 3.897302 0.979860 3.039131
H 4.970891 1.075615 2.862975
C 3.159742 2.044975 3.543807
H 0.385667 6.406226 2.191726
H 4.206938 6.809099 0.230618
H 0.048806 0.710536 3.706577
H 3.752801 -1.072211 2.395998
H 1.314990 -1.234150 2.836269
H 1.840647 7.501116 0.510593
F -2.035398 5.834156 -0.107772
F -0.269445 6.807073 -1.830155
F 2.139025 5.601176 -2.172344
F 2.739583 3.358154 -0.761581
F 0.966102 2.334005 0.942698
C -2.828268 4.376880 2.106314
C -4.111234 4.231205 1.582830
C -2.678188 5.414454 3.021648
C -5.185626 5.032820 1.961011
C -3.721045 6.238423 3.433780
C -4.989473 6.041521 2.898452
B -1.585275 3.419011 1.676469
C -2.036408 1.884593 1.368956
C -2.126838 1.343292 0.088391
C -2.343786 1.009423 2.408553
C -2.463370 0.013914 -0.155182
C -2.671049 -0.329645 2.211855
C -2.734041 -0.830516 0.916419
F -1.900691 2.097364 -0.986751
F -2.533639 -0.451104 -1.394790
F -3.050736 -2.097552 0.704806
F -2.923958 -1.123708 3.243644
F -2.319584 1.422529 3.679532
F -4.362297 3.309722 0.654055
F -6.386957 4.847980 1.434142
F -5.998149 6.810775 3.272060
F -3.516607 7.204107 4.318036
F -1.474161 5.697452 3.553143
F -0.720778 3.307861 2.911705

Int-2B

Bi 7.052880 5.528288 2.896633
S 4.156566 6.229168 4.012661
O 2.736256 6.368413 4.312798
C 6.233802 7.636064 3.067522
C 4.961026 7.748310 3.615576
C 4.324804 8.956396 3.874666
H 3.326033 8.984439 4.315560

C 5.015287 10.119566 3.533289
H 4.554227 11.092809 3.713836
C 6.283076 10.041827 2.953563
C 6.896284 8.807529 2.718233
H 7.891034 8.769595 2.273231
C 6.362981 5.098268 5.035009
C 5.075421 5.519686 5.358991
C 4.503871 5.379821 6.618137
H 3.492649 5.739778 6.819049
C 5.267955 4.742058 7.595564
H 4.858229 4.605627 8.598455
C 6.546175 4.270132 7.292128
C 7.104064 4.453447 6.021409
H 8.113288 4.091978 5.813894
H 6.810603 10.960001 2.683373
H 7.127271 3.758029 8.062819
C 10.480740 9.600009 5.455370
C 10.268758 8.591379 4.517089
C 10.311465 9.002373 3.187273
C 10.669932 10.936295 5.111241
C 10.485680 10.328141 2.797615
C 10.666808 11.304979 3.770148
B 9.962639 7.035187 4.904757
F 8.925837 6.585538 3.913061
C 11.246515 6.062489 4.665966
C 11.101849 4.731320 4.281882
C 12.561211 6.490340 4.838570
C 12.176901 3.878723 4.044007
C 13.667192 5.675279 4.609639
C 13.472542 4.359474 4.203932
C 9.267109 6.898103 6.372627
C 8.034101 7.509592 6.597276
C 9.797719 6.213363 7.462564
C 7.346064 7.438213 7.801098
C 9.145153 6.121967 8.692139
C 7.908300 6.732908 8.858959
F 10.153158 8.120352 2.193975
F 10.470818 10.661757 1.513991
F 10.836374 12.571296 3.425066
F 10.525925 9.322227 6.758188
F 9.884066 4.184836 4.138736
F 11.979349 2.620213 3.675333
F 14.511206 3.571802 3.978337
F 14.897785 6.137651 4.779686
F 12.821644 7.725836 5.265700
F 10.980360 5.604195 7.386878
F 7.445013 8.202848 5.622411
F 6.154593 8.004308 7.941785
F 7.255575 6.622818 10.004566
F 9.686724 5.446508 9.697111
F 10.856092 11.856516 6.047605
N 4.633943 5.354776 2.735219
C 3.986119 4.138995 2.458610
F 2.720894 4.313364 2.078953
F 4.639294 3.537852 1.465639
F 3.957440 3.281959 3.492784

Int-2C

Bi 6.998657 5.541754 2.861533
S 4.172131 6.229523 3.968392
O 2.746645 6.356237 4.274221
C 6.226212 7.663750 3.031208
C 4.950381 7.761980 3.573202
C 4.298552 8.964913 3.819219
H 3.297822 8.982457 4.256248
C 4.975328 10.133877 3.471433
H 4.501928 11.103119 3.641871
C 6.247795 10.067748 2.898609
C 6.877706 8.840034 2.675290
H 7.875605 8.810470 2.236663
C 6.365278 5.121558 5.023922
C 5.080916 5.551660 5.347542
C 4.506424 5.398396 6.604128
H 3.496002 5.760977 6.804856
C 5.264651 4.747383 7.577452
H 4.852462 4.602209 8.578237
C 6.541466 4.270767 7.272051
C 7.102536 4.462412 6.004269
H 8.108897 4.095323 5.792700

H 6.764855 10.990910 2.624709
H 7.117284 3.747244 8.039182
C 10.552270 9.577883 5.501352
C 10.300078 8.611502 4.529150
C 10.302951 9.080108 3.217934
C 10.737435 10.926927 5.210507
C 10.468524 10.421786 2.881490
C 10.689292 11.354605 3.888037
B 9.968876 7.046250 4.870911
F 8.942837 6.632428 3.876908
C 11.247151 6.061014 4.632567
C 11.083377 4.735290 4.236430
C 12.568437 6.466780 4.807620
C 12.145639 3.869381 3.988724
C 13.662369 5.637541 4.570203
C 13.448384 4.328708 4.152677
C 9.263372 6.901433 6.336774
C 8.036402 7.527060 6.554763
C 9.773474 6.199781 7.425124
C 7.335235 7.453616 7.751149
C 9.107073 6.104949 8.646841
C 7.876894 6.730670 8.807281
F 10.115946 8.244898 2.189691
F 10.409976 10.811770 1.614564
F 10.854905 12.634323 3.592428
F 10.641352 9.244026 6.789062
F 9.858352 4.206978 4.090487
F 11.929697 2.617226 3.607080
F 14.475307 3.527302 3.919426
F 14.900251 6.080332 4.743039
F 12.850373 7.694799 5.243125
F 10.948903 5.574511 7.354383
F 7.465179 8.237045 5.581772
F 6.149619 8.034190 7.885164
F 7.208924 6.614401 9.944204
F 9.625978 5.408549 9.650302
F 10.960424 11.803340 6.180693
N 4.641567 5.337895 2.714900
C 4.075843 3.987061 2.703388
H 4.402036 3.497846 1.776219
H 4.396109 3.366606 3.560610
H 2.976082 4.030458 2.694485

Int-3A

Bi 7.507408 8.143265 3.275703
S 4.606832 7.120837 2.480736
O 5.567208 7.986504 1.714219
O 3.317637 6.783050 1.895944
C 5.635552 8.491302 4.540097
C 4.447755 7.967517 4.029120
C 3.219312 8.076521 4.669478
H 2.320243 7.644685 4.224198
C 3.184724 8.760310 5.885493
H 2.236677 8.875307 6.415288
C 4.356171 9.295783 6.421953
C 5.581725 9.164459 5.757718
H 6.485643 9.581768 6.206516
C 6.902772 5.939251 3.205194
C 5.574696 5.684789 2.863274
C 5.024715 4.409143 2.814748
H 3.977474 4.265496 2.539523
C 5.859606 3.333648 3.121564
H 5.464273 2.316065 3.092163
C 7.196186 3.556335 3.455285
C 7.723095 4.852535 3.492572
H 8.774499 5.000982 3.744008
H 4.319866 9.825408 7.377138
H 7.844312 2.709199 3.693787
C 11.327282 9.745889 4.776319
C 10.783976 8.478249 4.568817
C 10.883033 8.010010 3.258959
C 11.963677 10.475547 3.771859
C 11.500056 8.701145 2.223238
C 12.051077 9.950944 2.487617
B 9.991656 7.602555 5.700721
C 10.370686 5.997775 5.714266
C 9.476082 5.110626 6.310163
C 11.528976 5.410641 5.209161
C 9.660793 3.734041 6.366013

C 11.763505 4.036507 5.247053
C 10.821250 3.191850 5.823648
C 10.229844 8.108234 7.241284
C 9.237193 8.457604 8.150230
C 11.525411 8.102169 7.757201
C 9.499315 8.803313 9.475898
C 11.837297 8.443324 9.067687
C 10.809102 8.795829 9.938230
F 10.331376 6.829970 2.928604
F 11.551114 8.195831 1.000489
F 12.641872 10.636941 1.525510
F 11.258661 10.342920 5.963364
F 8.338718 5.575751 6.856020
F 8.748199 2.942401 6.911020
F 11.025282 1.885380 5.855679
F 12.875928 3.528357 4.736776
F 12.480194 6.144594 4.635879
F 12.545019 7.771638 6.962575
F 7.944233 8.471182 7.796670
F 8.509092 9.130199 10.295375
F 11.077962 9.120155 11.192627
F 13.091596 8.432167 9.495932
F 12.470114 11.672013 4.028076
O 8.528748 7.750652 5.308253
H 7.944697 7.295625 5.932421

TS-2A

Imaginary frequency: -166.16 cm^{-1}

Bi 0.858911 4.538558 3.412699
S 3.979609 4.116757 4.159508
O 5.351249 4.016166 4.646145
O 2.967214 4.876393 4.950361
C 2.636718 5.140416 2.064935
C 2.559051 5.779791 0.828401
C 3.722808 6.092594 0.117536
C 4.981778 5.774095 0.626562
C 5.089164 5.145142 1.866341
H 6.057710 4.893403 2.303931
C 3.913984 4.854481 2.550756
C -0.464140 4.103742 1.360555
C -0.911833 5.317350 0.814158
C -0.857060 5.625533 -0.539630
C -0.254056 4.715512 -1.405458
C 0.260500 3.513625 -0.917111
C 0.163622 3.246785 0.440332
C 1.881380 2.488860 3.653016
C 1.266511 1.239870 3.596551
C 2.014923 0.076682 3.804078
C 3.381372 0.137880 4.076048
C 4.014529 1.376836 4.160922
H 5.077303 1.467244 4.395885
C 3.245253 2.517367 3.954468
H 1.593689 6.055924 0.400487
H 5.883096 6.023940 0.062700
H 0.200046 1.152635 3.399917
H 3.953811 -0.778203 4.236956
H 1.513177 -0.892884 3.752512
H 3.639933 6.595052 -0.849328
F -1.413401 6.253553 1.611837
F -1.321333 6.771110 -1.005956
F -0.169363 4.989143 -2.686744
F 0.822354 2.655659 -1.750032
F 0.653076 2.084461 0.846727
C -3.272468 4.096396 2.548420
C -4.521201 3.976815 1.927827
C -3.161127 5.158283 3.451608
C -5.561838 4.879000 2.139611
C -4.174348 6.077648 3.692018
C -5.388433 5.934195 3.027759
B -2.079469 3.043986 2.390383
C -2.221442 1.794034 1.403919
C -2.493829 1.945602 0.043263
C -2.041053 0.481775 1.838536
C -2.527370 0.886529 -0.852024
C -2.076229 -0.612321 0.974735
C -2.319895 -0.407881 -0.377579
F -2.703699 3.161908 -0.457088
F -2.760293 1.090784 -2.137901

F -2.356212 -1.431494 -1.208948
F -1.887489 -1.838108 1.435783
F -1.818356 0.206567 3.120756
F -4.800878 2.966740 1.113923
F -6.722645 4.719408 1.525026
F -6.367346 6.790920 3.241312
F -3.998383 7.072412 4.545921
F -2.029328 5.348225 4.126414
F -1.456127 2.762455 3.580937

TS-3A

Imaginary frequency: -176.10 cm^{-1}

Bi 0.823513 4.454375 3.390096
S 3.914571 4.110017 4.221815
O 5.259825 4.057152 4.783156
O 2.834759 4.837843 4.959799
C 2.636534 5.096188 2.076056
C 2.597591 5.735547 0.837282
C 3.782105 6.082831 0.178009
C 5.026878 5.800093 0.740808
C 5.098209 5.172094 1.983677
H 6.053371 4.948048 2.464020
C 3.901475 4.847795 2.613158
C -0.539456 4.074413 1.284429
C -0.920805 5.297290 0.705096
C -0.760102 5.602597 -0.641113
C -0.122507 4.680091 -1.466946
C 0.336851 3.471861 -0.942317
C 0.142976 3.212111 0.406057
C 1.889133 2.428410 3.635587
C 1.308580 1.164654 3.547558
C 2.081361 0.020775 3.774364
C 3.434894 0.115733 4.098317
C 4.032196 1.369905 4.215538
H 5.082424 1.485751 4.492306
C 3.239755 2.489941 3.986505
H 1.644879 5.987057 0.368081
H 5.944329 6.078138 0.217569
H 0.249324 1.061369 3.317595
H 4.024526 -0.786414 4.275705
H 1.609880 -0.962385 3.698614
H 3.727421 6.586092 -0.790545
F -1.477736 6.246939 1.446137
F -1.169202 6.758614 -1.134980
F 0.056477 4.951541 -2.739609
F 0.944707 2.603717 -1.731677
F 0.589399 2.042593 0.843940
C -3.261858 4.098852 2.544428
C -4.547314 3.862872 2.043300
C -3.156719 5.233114 3.355422
C -5.630503 4.704616 2.293428
C -4.205890 6.104253 3.622783
C -5.460195 5.832680 3.086359
B -2.002797 3.093048 2.364523
C -2.219817 1.836018 1.365643
C -2.545180 2.013612 0.019446
C -2.076352 0.509465 1.768115
C -2.655221 0.974286 -0.892428
C -2.186694 -0.568315 0.887315
C -2.476707 -0.335166 -0.449983
F -2.755874 3.244446 -0.452334
F -2.941492 1.209981 -2.163857
F -2.589270 -1.343215 -1.296629
F -2.027181 -1.809787 1.323175
F -1.818821 0.186569 3.034039
F -4.827996 2.789726 1.311750
F -6.826011 4.424078 1.798543
F -6.480537 6.631527 3.335398
F -4.024358 7.173385 4.381748
F -1.990176 5.537660 3.931622
O -1.347425 2.772665 3.581719
H -1.853933 3.011373 4.365445

$B(Ar^F)_2F$

C 1.368426 7.710089 4.169910
C 0.333265 8.288882 4.913051
C 2.354260 7.027378 4.892356

C 0.277535 8.213876 6.299060
C 2.333883 6.943230 6.280857
C 1.288267 7.539611 6.982335
B 1.433517 7.814035 2.611876
C 0.857568 9.010714 1.783607
C 0.879941 10.324225 2.263369
C 0.292406 8.813778 0.519542
C 0.366811 11.392131 1.538159
C -0.244700 9.858632 -0.226078
C -0.201939 11.152379 0.288827
F 1.417742 10.597244 3.444145
F 0.414071 12.623081 2.015488
F -0.702136 12.153590 -0.403896
F -0.792396 9.637458 -1.407805
F 0.218903 7.596624 0.003353
F -0.657417 8.924788 4.303543
F -0.717796 8.768204 6.968117
F 1.255196 7.466691 8.295782
F 3.289493 6.307943 6.935771
F 3.370838 6.456384 4.266181
F 2.019757 6.826208 1.950011

$B(Ar^F)_2OH$

C 1.444228 7.749971 4.169849
C 0.412484 8.383281 4.873856
C 2.299717 6.951227 4.937325
C 0.246146 8.248301 6.248956
C 2.165854 6.789049 6.310519
C 1.127482 7.445647 6.967473
B 1.637278 7.902291 2.609758
C 0.937030 9.054832 1.786241
C 1.056756 10.393725 2.150535
C 0.171860 8.774164 0.656661
C 0.448293 11.419614 1.436567
C -0.464247 9.772989 -0.076628
C -0.321164 11.100527 0.318975
F 1.776789 10.723019 3.219735
F 0.586816 12.683577 1.802707
F -0.918668 12.060452 -0.361499
F -1.201253 9.473774 -1.134086
F -0.001042 7.517720 0.261924
F -0.475094 9.141335 4.247448
F -0.741986 8.864447 6.873487
F 0.978976 7.303599 8.267176
F 2.997510 6.021911 6.992174
F 3.308906 6.302401 4.352848
O 2.410843 7.066676 1.896648
H 2.834161 6.367246 2.412224

[Bi-3]•Ar^F

Bi 0.861745 3.492051 3.642665
S 4.090656 3.499330 4.466263
O 5.503042 3.502550 4.844561
O 3.046263 3.504865 5.514403
C 2.399783 5.067723 3.032451
C 2.094565 6.177410 2.246136
C 3.100183 7.077495 1.880862
C 4.420564 6.877129 2.286208
C 4.747604 5.770972 3.068991
H 5.770620 5.583678 3.402446
C 3.726311 4.896141 3.429383
C 0.003060 3.479390 1.505540
C -1.375027 3.477665 1.341487
C -1.978698 3.473584 0.086023
C -1.171071 3.471135 -1.048405
C 0.216131 3.472511 -0.918381
C 0.781975 3.476420 0.354538
C 2.402191 1.912175 3.050312
C 2.099012 0.794468 2.274579
C 3.106451 -0.106664 1.917077
C 4.426577 0.100399 2.319893
C 4.751542 1.214483 3.092230
H 5.774364 1.407142 3.423192
C 3.728519 2.090503 3.444908
H 1.070740 6.346627 1.900277
H 5.199149 7.584740 1.992716
H 1.075434 0.619857 1.930674
H 5.206593 -0.608109 2.032410

H 2.856162 -0.979581 1.308808
H 2.848218 7.944299 1.264616
F -2.179035 3.480171 2.407339
F -3.297315 3.472208 -0.041235
F -1.718923 3.467453 -2.250694
F 0.976596 3.470309 -2.003291
F 2.109532 3.477499 0.424379

PhB(OH)$_2$ (1-S1)

C -2.691815 2.192556 -0.209283
C -3.383352 3.394744 -0.049960
C -2.677931 4.576717 0.182656
C -1.286033 4.552066 0.256374
C -1.299650 2.177053 -0.137091
C -0.571032 3.354248 0.097200
H -0.737127 5.479763 0.439858
H -3.241853 1.265275 -0.390480
H -0.761884 1.233422 -0.263591
H -4.475099 3.410560 -0.107150
H -3.217012 5.519658 0.306836
B 0.996263 3.333536 0.178226
O 1.635354 2.149887 -0.045413
O 1.639273 4.501031 0.467727
H 2.600748 4.436541 0.504210
H 2.596510 2.188534 0.019909

Int-4A

H -1.615417 -1.885063 -2.189303
H -3.698820 -0.647213 -2.769628
C -2.208101 -1.404116 -1.408017
C -3.384957 -0.716490 -1.724950
Bi 0.061047 -2.614041 0.689451
F 2.022507 -4.530460 -1.179612
C -1.796715 -1.501019 -0.080230
C -4.166184 -0.126595 -0.730202
H -5.085871 0.400377 -0.993742
C -2.607258 -0.911866 0.890769
O -1.581069 -2.558416 2.574355
C -3.781015 -0.222574 0.606821
C 0.504750 -0.691511 1.964740
H 2.615116 -0.388686 1.614156
S -2.068093 -1.135556 2.560144
C 1.724544 -0.038414 2.135726
H -4.377669 0.211096 1.412329
C -0.575777 -0.198284 2.698971
O -3.076057 -0.717127 3.526233
C 1.821890 1.071726 2.983018
C -0.518142 0.902787 3.546261
H 2.787676 1.569364 3.102957
C 0.710464 1.548741 3.678657
H -1.405206 1.228420 4.094387
H 0.801958 2.414327 4.338473
H 2.686494 -3.668956 -3.444422
O 1.736077 -3.575928 -3.323799
B 1.426092 -3.462797 -1.930905
O -0.030817 -3.482816 -1.749357
H -0.405645 -4.320953 -2.047920
C 1.957526 -2.030694 -1.272728
C 3.234330 -1.972916 -0.664350
C 1.354935 -0.796564 -1.608646
C 3.900431 -0.766644 -0.478412
C 3.281859 0.435875 -0.841343
C 1.999064 0.421828 -1.384017
H 3.716876 -2.911939 -0.378832
H 4.902499 -0.752997 -0.041419
H 3.800231 1.385545 -0.685672
H 1.506408 1.359433 -1.653358
H 0.377504 -0.796794 -2.098630

Int-4B

H -1.443271 -1.949604 -2.126781
H -3.624734 -0.921272 -2.684169
C -2.074944 -1.549621 -1.325782
C -3.307397 -0.962468 -1.638963
Bi 0.271690 -2.554145 0.800216
F 0.167905 -4.059576 -1.374065
C -1.661577 -1.606602 0.004243

C -4.135271 -0.433966 -0.648217
H -5.094894 0.014983 -0.913949
C -2.514850 -1.076821 0.974707
O -1.363441 -2.595714 2.701341
C -3.740065 -0.485445 0.688045
C 0.571931 -0.577579 2.008822
H 2.641200 -0.095139 1.605516
S -1.955384 -1.215586 2.646588
C 1.728452 0.192452 2.127627
H -4.364926 -0.089879 1.491780
C -0.540407 -0.155828 2.740615
O -2.984556 -0.843302 3.608683
C 1.733671 1.345664 2.921209
C -0.571853 0.984801 3.535556
H 2.649395 1.937532 2.998488
C 0.592562 1.748051 3.615436
H -1.478200 1.255658 4.081860
H 0.611116 2.649940 4.231328
H 2.628542 -4.398154 -2.237542
O 2.013701 -4.035697 -2.881779
B 0.994969 -3.249090 -2.263873
O 0.103234 -2.693133 -3.238770
H 0.604346 -2.375607 -3.995787
C 1.726337 -1.990837 -1.347244
C 2.995659 -2.178473 -0.734044
C 1.359553 -0.649037 -1.610132
C 3.858155 -1.118941 -0.473661
C 3.469462 0.186475 -0.791994
C 2.208649 0.422671 -1.337824
H 3.331160 -3.193397 -0.491110
H 4.838646 -1.305429 -0.028489
H 4.148186 1.021063 -0.597788
H 1.894516 1.443046 -1.570795
H 0.398721 -0.459634 -2.095183

Int-4C

H -2.436560 -2.174282 -2.085890
H -4.297625 -0.635422 -2.586917
C -2.919123 -1.638258 -1.264474
C -3.972521 -0.762685 -1.551192
Bi -0.775768 -3.183283 0.766413
F 1.840286 -3.462268 0.163583
C -2.495570 -1.816266 0.051700
C -4.611294 -0.049003 -0.535862
H -5.433796 0.628739 -0.774794
C -3.155028 -1.084137 1.039223
O -2.302303 -2.804273 2.761482
C -4.197424 -0.198624 0.787506
C 0.021405 -1.340444 1.866200
H 2.090834 -1.388186 1.253724
S -2.542619 -1.327269 2.684573
C 1.329852 -0.865324 1.834389
H -4.669812 0.349907 1.605471
C -0.907056 -0.643637 2.641485
O -3.379204 -0.680656 3.687831
C 1.665498 0.291062 2.548152
C -0.600613 0.506318 3.359938
H 2.693779 0.659582 2.511601
C 0.712105 0.975487 3.302299
H -1.368265 1.010089 3.951521
H 0.991308 1.874248 3.856609
H 2.781426 -3.757653 -2.606960
O 1.933704 -3.888357 -2.173528
B 1.709533 -2.895724 -1.181780
O 0.220382 -2.565015 -1.221475
H -0.155626 -2.995345 -2.002788
C 2.598516 -1.555505 -1.279252
C 3.952500 -1.562276 -0.904393
C 2.060878 -0.339427 -1.725416
C 4.735482 -0.408726 -0.970383
C 4.175678 0.790193 -1.416171
C 2.833094 0.821413 -1.795424
H 4.405606 -2.489721 -0.538297
H 5.786407 -0.441588 -0.668948
H 4.784228 1.697258 -1.465534
H 2.385062 1.756962 -2.142646
H 1.004687 -0.301767 -2.008027

TS-4A

Imaginary frequency: -280.23 cm^{-1}

H -1.563781 -1.929703 -2.140079
H -3.616492 -0.672542 -2.753519
C -2.164581 -1.452670 -1.363298
C -3.327958 -0.753067 -1.702254
Bi 0.100568 -2.673590 0.718533
F 2.022014 -4.392610 -1.165014
C -1.772872 -1.564487 -0.028686
C -4.123290 -0.163719 -0.719971
H -5.031805 0.376399 -0.995708
C -2.599016 -0.975044 0.932017
O -1.772649 -2.621246 2.756200
C -3.761606 -0.275940 0.621784
C 0.457892 -0.789187 2.043971
H 2.542563 -0.426445 1.597284
S -2.143483 -1.188271 2.635167
C 1.665003 -0.098335 2.158948
H -4.366358 0.159363 1.420234
C -0.620047 -0.295265 2.782341
O -3.167892 -0.629346 3.516675
C 1.767130 1.028073 2.982063
C -0.555256 0.828073 3.601802
H 2.723379 1.552405 3.057050
C 0.665634 1.493199 3.701629
H -1.440763 1.162038 4.147121
H 0.756093 2.374282 4.340937
H 2.825930 -3.234904 -3.179300
O 1.863370 -3.190812 -3.183405
B 1.335920 -3.447477 -1.925150
O -0.075906 -3.578566 -1.866735
H -0.367769 -4.421366 -2.236638
C 1.684831 -1.803153 -0.874922
C 3.023593 -1.888079 -0.427057
C 1.280972 -0.589620 -1.474279
C 3.930481 -0.852594 -0.636335
C 3.502621 0.324183 -1.254318
C 2.173729 0.459519 -1.664954
H 3.367290 -2.809177 0.053078
H 4.968957 -0.956418 -0.312021
H 4.208456 1.143668 -1.413578
H 1.839301 1.385079 -2.139945
H 0.249935 -0.472784 -1.818201

TS-4B

Imaginary frequency: -131.84 cm^{-1}

H -1.490886 -2.002722 -2.110965
H -3.646139 -0.939936 -2.688948
C -2.120405 -1.594520 -1.314686
C -3.337047 -0.982814 -1.641282
Bi 0.219021 -2.623565 0.791368
F 0.156451 -4.091035 -1.430441
C -1.710754 -1.652945 0.017398
C -4.156476 -0.428996 -0.658090
H -5.104703 0.039971 -0.930375
C -2.553038 -1.090768 0.980603
O -1.513949 -2.605279 2.796508
C -3.763869 -0.476370 0.679071
C 0.530903 -0.666394 2.000666
H 2.597147 -0.236138 1.530160
S -2.019468 -1.207606 2.667519
C 1.707345 0.078808 2.078309
H -4.380031 -0.056027 1.476858
C -0.561074 -0.203432 2.737137
O -3.043501 -0.720661 3.587425
C 1.758910 1.240839 2.856203
C -0.545138 0.949699 3.515079
H 2.689075 1.812911 2.904052
C 0.642036 1.679146 3.568934
H -1.437004 1.255704 4.066548
H 0.695678 2.587187 4.173664
H 2.558414 -4.450885 -2.069352
O 2.123881 -3.905643 -2.732348
B 0.995106 -3.240000 -2.212845
O 0.204785 -2.595820 -3.188229
H 0.759008 -2.185592 -3.859396

C 1.662268 -1.893970 -1.090201
C 2.984560 -2.088147 -0.608719
C 1.322881 -0.569825 -1.458181
C 3.921507 -1.061795 -0.577352
C 3.551574 0.222465 -0.989089
C 2.245542 0.471802 -1.412912
H 3.296983 -3.087825 -0.285650
H 4.940331 -1.255833 -0.233377
H 4.283585 1.034133 -0.967514
H 1.951492 1.478836 -1.718775
H 0.315139 -0.367563 -1.830465

$BF(OH)_2$

F 2.375944 -2.951365 -0.260519
H 2.909045 -4.199223 -2.279113
O 2.138567 -3.653959 -2.479076
B 1.731420 -2.897220 -1.433662
O 0.673884 -2.062541 -1.514334
H 0.257612 -2.076505 -2.384235

8 X-Ray

8.1 Single crystal structure of [Bi-3]•B(Ar^F)$_3$F

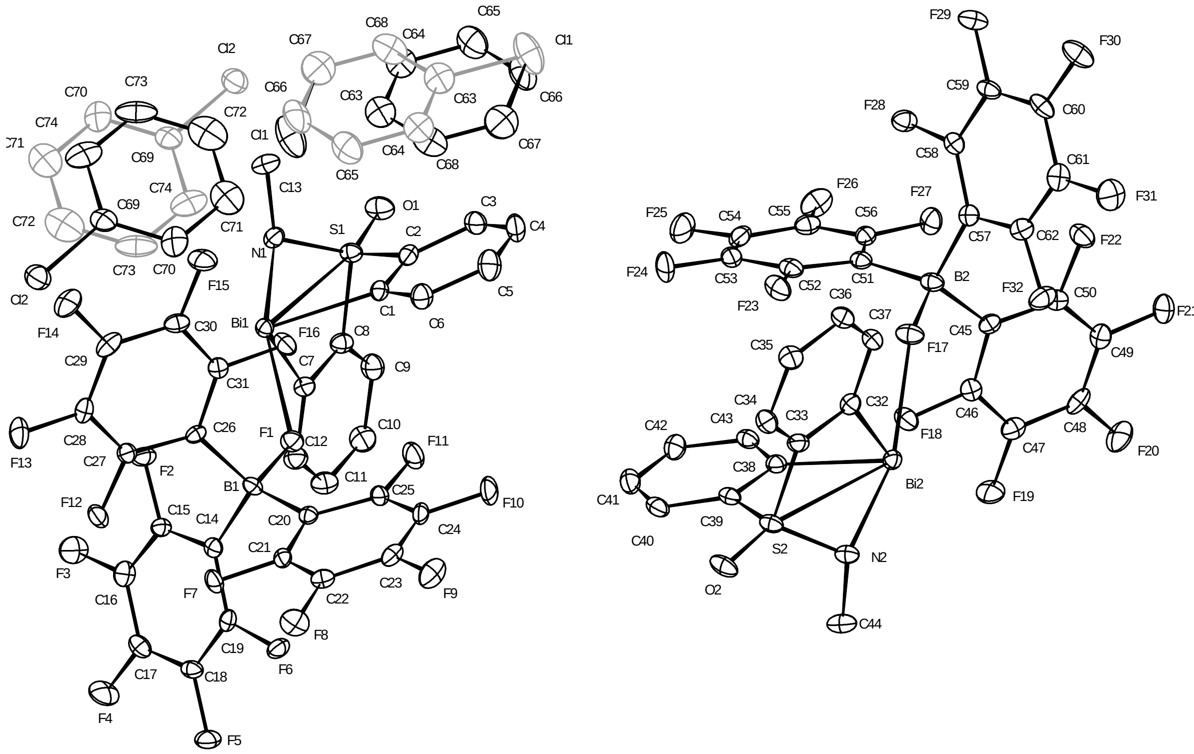

Figure S37. The molecular structure of **[Bi-3]•B(Ar^F)$_3$F**. The H atoms have been omitted for clarity, and the minor disorder is shown in grey.

X-ray Crystal Structure Analysis of **[Bi-3]•B(Ar^F)$_3$F**:

C_{68} H_{27} B_2 Bi_2 Cl F_{32} N_2 O_2 S_2, M_r = 2051.06 g mol^{-1}, colourless prism, crystal size 0.165 x 0.083 x 0.082 mm^3, triclinic, space group *P*-1 [2], a = 14.1799(6) Å, b = 16.1675(7) Å, c = 17.2448(7) Å, α = 85.454(2)°, β = 66.801(2)°, γ = 65.489(2)°, *V* = 3289.5(2) Å^3, *T* = 100(2) K, *Z* = 2, D_{calc} = 2.071 g·cm^3, λ = 0.71073 Å, $\mu(Mo\text{-}K_\alpha)$ = 5.588 mm^{-1}, Numerical correction (T_{min} = 0.60069, T_{max} = 0.76514), Bruker-AXS Mach3 Goniometer with APEXII detector and IµS microfocus Mo-anode X-ray source, 1.291 to 31.759°, 115569 measured reflections, 22178 independent reflections, 17405 reflections with $I > 2\sigma(I)$, R_{int} = 0.0569. The structure was solved by *SHELXT* and refined by full-matrix least-squares (*SHELXL*) against F^2 to R_1 = 0.0306 [$I > 2\sigma(I)$], wR_2 = 0.0550 [all data], 1065 parameters and 266.

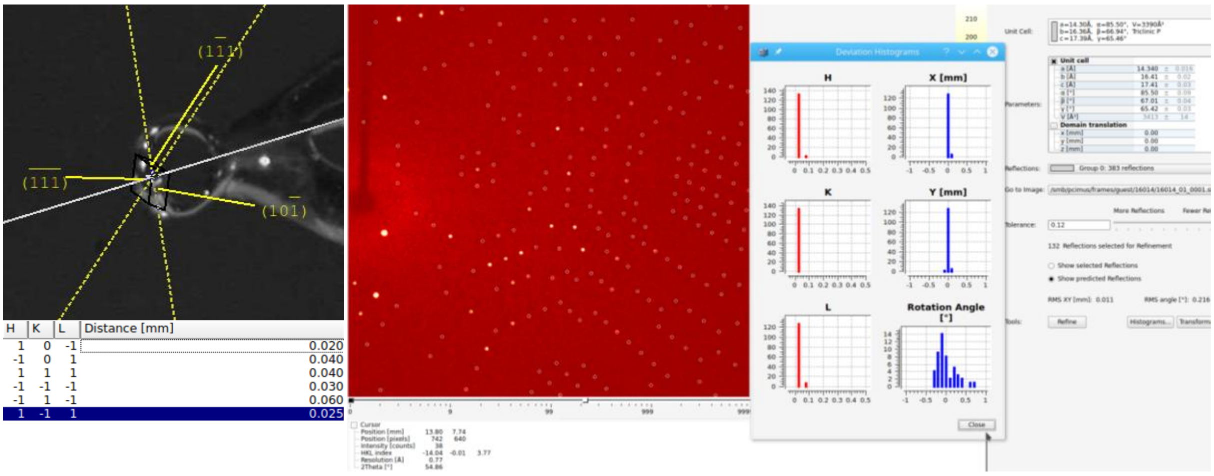

Figure S38. Crystal faces and unit cell determination/refinement of **[Bi-3]•B(Ar^F)$_3$F**.

INTENSITY STATISTICS FOR DATASET

Resolution	#Data	#Theory	%Complete	Redundancy	Mean I	Mean I/s	Rmerge	Rsigma
Inf - 2.74	333	333	100.0	9.44	93.60	51.76	0.0237	0.0127
2.74 - 1.83	795	795	100.0	9.62	67.66	46.88	0.0282	0.0141
1.83 - 1.45	1125	1125	100.0	9.62	47.24	40.90	0.0353	0.0164
1.45 - 1.27	1093	1093	100.0	9.47	37.18	36.09	0.0424	0.0192
1.27 - 1.15	1163	1163	100.0	9.09	31.55	31.87	0.0492	0.0221
1.15 - 1.07	1107	1107	100.0	8.07	26.00	26.35	0.0594	0.0276
1.07 - 1.01	1062	1062	100.0	6.30	25.34	22.18	0.0628	0.0343
1.01 - 0.96	1107	1107	100.0	5.32	21.04	17.71	0.0704	0.0429
0.96 - 0.91	1347	1347	100.0	4.67	18.52	15.11	0.0802	0.0512
0.91 - 0.88	972	972	100.0	4.17	16.27	12.97	0.0873	0.0601
0.88 - 0.85	1123	1123	100.0	4.01	14.56	11.49	0.0934	0.0684
0.85 - 0.82	1253	1253	100.0	3.82	13.84	10.65	0.0992	0.0750
0.82 - 0.80	1004	1004	100.0	3.74	11.80	9.25	0.1137	0.0884
0.80 - 0.78	1045	1045	100.0	3.60	11.46	8.60	0.1199	0.0947
0.78 - 0.76	1175	1175	100.0	3.48	10.49	7.68	0.1325	0.1065
0.76 - 0.74	1304	1305	99.9	3.41	10.37	7.43	0.1410	0.1114
0.74 - 0.72	1458	1461	99.8	3.28	9.32	6.49	0.1585	0.1284
0.72 - 0.71	759	761	99.7	3.24	8.65	6.03	0.1762	0.1394
0.71 - 0.70	855	856	99.9	3.09	7.67	5.32	0.1901	0.1580
0.70 - 0.68	2098	2288	91.7	2.59	7.11	4.68	0.2116	0.1931
0.78 - 0.68	7649	7846	97.5	3.10	8.82	6.16	0.1640	0.1392
Inf - 0.68	22178	22375	99.1	5.17	20.75	16.86	0.0558	0.0490

Complete .cif-data are available under the CCDC number CCDC-**2504150**.

The 'Chlorobenzene' fragment from the DSR tool[27, 28], which is implemented in Olex2[29], was used to describe the disordered part containing the solute.

Table S1. Crystal data and structure refinement **[Bi-3]•B(Ar^{F})$_3$F**.

Identification code	16014	
Empirical formula	$C_{68} H_{27} B_2 Bi_2 Cl F_{32} N_2 O_2 S_2$	
Color	colourless	
Formula weight	2051.06 g·mol^{-1}	
Temperature	100(2) K	
Wavelength	0.71073 Å	
Crystal system	Triclinic	
Space group	*P*-1, (no. 2)	
Unit cell dimensions	a = 14.1799(6) Å	α= 85.454(2)°.
	b = 16.1675(7) Å	β= 66.801(2)°.
	c = 17.2448(7) Å	γ = 65.489(2)°.
Volume	3289.5(2) Å^3	
Z	2	
Density (calculated)	2.071 mg·m^{-3}	
Absorption coefficient	5.588 mm^{-1}	
F(000)	1956 e	
Crystal size	0.165 x 0.083 x 0.082 mm^3	
θ range for data collection	1.291 to 31.759°.	
Index ranges	$-20 \leq h \leq 20$, $-23\leq k \leq 23$, $-25 \leq l \leq 24$	
Reflections collected	115569	
Independent reflections	22178 [R_{int} = 0.0569]	
Reflections with I>2σ(I)	17405	
Completeness to θ = 25.242°	100.0%	
Absorption correction	Gaussian	
Max. and min. transmission	0.76514 and 0.60069	
Refinement method	Full-matrix least-squares on F^2	
Data / restraints / parameters	22178 / 266 / 1065	
Goodness-of-fit on F^2	1.020	
Final R indices [I>2σ(I)]	R_1 = 0.0306	wR^2 = 0.0507
R indices (all data)	R_1 = 0.0503	wR^2 = 0.0550
Extinction coefficient	n/a	
Largest diff. peak and hole	0.859 and -1.085 e·Å^{-33}	

Table S2. Bond lengths [Å] and angles [°] **[Bi-3]•B(Ar^{F})$_{3}$F**.

Bi(1)-F(1)	2.4678(13)	Bi(1)-C(1)	2.273(2)
Bi(1)-C(7)	2.242(2)	S(1)-O(1)	1.4382(19)
S(1)-N(1)	1.576(2)	S(1)-C(2)	1.768(3)
S(1)-C(8)	1.767(2)	F(1)-B(1)	1.480(3)
F(2)-C(15)	1.365(3)	F(3)-C(16)	1.344(3)
F(4)-C(17)	1.339(3)	F(5)-C(18)	1.341(3)
F(6)-C(19)	1.344(3)	F(7)-C(21)	1.347(3)
F(8)-C(22)	1.350(3)	F(9)-C(23)	1.345(3)
F(10)-C(24)	1.348(3)	F(11)-C(25)	1.356(3)
F(12)-C(27)	1.345(3)	F(13)-C(28)	1.341(3)
F(14)-C(29)	1.340(3)	F(15)-C(30)	1.343(3)
F(16)-C(31)	1.352(3)	N(1)-C(13)	1.495(3)
C(1)-C(2)	1.393(4)	C(1)-C(6)	1.380(3)
C(2)-C(3)	1.385(4)	C(3)-H(3)	0.9500
C(3)-C(4)	1.386(4)	C(4)-H(4)	0.9500
C(4)-C(5)	1.384(4)	C(5)-H(5)	0.9500
C(5)-C(6)	1.386(4)	C(6)-H(6)	0.9500
C(7)-C(8)	1.379(4)	C(7)-C(12)	1.386(3)
C(8)-C(9)	1.388(4)	C(9)-H(9)	0.9500
C(9)-C(10)	1.394(4)	C(10)-H(10)	0.9500
C(10)-C(11)	1.375(4)	C(11)-H(11)	0.9500
C(11)-C(12)	1.387(4)	C(12)-H(12)	0.9500
C(13)-H(13A)	0.9800	C(13)-H(13B)	0.9800
C(13)-H(13C)	0.9800	C(14)-C(15)	1.390(3)
C(14)-C(19)	1.384(3)	C(14)-B(1)	1.637(4)
C(15)-C(16)	1.374(3)	C(16)-C(17)	1.383(3)
C(17)-C(18)	1.371(3)	C(18)-C(19)	1.384(3)
C(20)-C(21)	1.398(3)	C(20)-C(25)	1.381(3)
C(20)-B(1)	1.631(4)	C(21)-C(22)	1.365(3)
C(22)-C(23)	1.370(4)	C(23)-C(24)	1.366(4)
C(24)-C(25)	1.378(3)	C(26)-C(27)	1.392(4)
C(26)-C(31)	1.391(3)	C(26)-B(1)	1.648(4)
C(27)-C(28)	1.381(3)	C(28)-C(29)	1.377(4)
C(29)-C(30)	1.378(4)	C(30)-C(31)	1.378(3)
Bi(2)-F(17)	2.4697(14)	Bi(2)-C(32)	2.243(2)
Bi(2)-C(38)	2.277(3)	S(2)-O(2)	1.4352(19)

S(2)-N(2)	1.580(2)	S(2)-C(33)	1.764(2)
S(2)-C(39)	1.784(3)	F(17)-B(2)	1.471(3)
F(18)-C(46)	1.358(3)	F(19)-C(47)	1.346(3)
F(20)-C(48)	1.341(3)	F(21)-C(49)	1.336(3)
F(22)-C(50)	1.354(3)	F(23)-C(52)	1.358(3)
F(24)-C(53)	1.345(3)	F(25)-C(54)	1.347(3)
F(26)-C(55)	1.345(3)	F(27)-C(56)	1.353(3)
F(28)-C(58)	1.354(3)	F(29)-C(59)	1.349(3)
F(30)-C(60)	1.339(3)	F(31)-C(61)	1.343(3)
F(32)-C(62)	1.362(3)	N(2)-C(44)	1.490(3)
C(32)-C(33)	1.382(4)	C(32)-C(37)	1.389(3)
C(33)-C(34)	1.383(4)	C(34)-H(34)	0.9500
C(34)-C(35)	1.392(3)	C(35)-H(35)	0.9500
C(35)-C(36)	1.385(4)	C(36)-H(36)	0.9500
C(36)-C(37)	1.388(4)	C(37)-H(37)	0.9500
C(38)-C(39)	1.386(4)	C(38)-C(43)	1.389(3)
C(39)-C(40)	1.380(4)	C(40)-H(40)	0.9500
C(40)-C(41)	1.388(4)	C(41)-H(41)	0.9500
C(41)-C(42)	1.386(4)	C(42)-H(42)	0.9500
C(42)-C(43)	1.387(4)	C(43)-H(43)	0.9500
C(44)-H(44A)	0.9800	C(44)-H(44B)	0.9800
C(44)-H(44C)	0.9800	C(45)-C(46)	1.394(3)
C(45)-C(50)	1.383(4)	C(45)-B(2)	1.646(4)
C(46)-C(47)	1.374(3)	C(47)-C(48)	1.371(4)
C(48)-C(49)	1.381(4)	C(49)-C(50)	1.382(3)
C(51)-C(52)	1.382(4)	C(51)-C(56)	1.397(3)
C(51)-B(2)	1.633(4)	C(52)-C(53)	1.377(4)
C(53)-C(54)	1.375(4)	C(54)-C(55)	1.362(4)
C(55)-C(56)	1.372(3)	C(57)-C(58)	1.378(3)
C(57)-C(62)	1.386(3)	C(57)-B(2)	1.640(4)
C(58)-C(59)	1.382(3)	C(59)-C(60)	1.367(4)
C(60)-C(61)	1.378(4)	C(61)-C(62)	1.377(3)
Cl(1)-C(63)	1.736(6)	C(63)-C(64)	1.377(8)
C(63)-C(68)	1.378(8)	C(64)-H(64)	0.9500
C(64)-C(65)	1.383(8)	C(65)-H(65)	0.9500
C(65)-C(66)	1.381(9)	C(66)-H(66)	0.9500
C(66)-C(67)	1.380(9)	C(67)-H(67)	0.9500
C(67)-C(68)	1.383(8)	C(68)-H(68)	0.9500

Cl(2)-C(69)	1.733(6)	C(69)-C(70)	1.378(7)
C(69)-C(74)	1.394(7)	C(70)-H(70)	0.9500
C(70)-C(71)	1.389(7)	C(71)-H(71)	0.9500
C(71)-C(72)	1.392(8)	C(72)-H(72)	0.9500
C(72)-C(73)	1.389(8)	C(73)-H(73)	0.9500
C(73)-C(74)	1.386(8)	C(74)-H(74)	0.9500
C(1)-Bi(1)-F(1)	90.13(7)	C(7)-Bi(1)-F(1)	83.55(7)
C(7)-Bi(1)-C(1)	87.06(9)	O(1)-S(1)-N(1)	120.10(11)
O(1)-S(1)-C(2)	111.88(12)	O(1)-S(1)-C(8)	113.09(12)
N(1)-S(1)-C(2)	105.08(12)	N(1)-S(1)-C(8)	100.31(12)
C(8)-S(1)-C(2)	104.75(11)	B(1)-F(1)-Bi(1)	133.81(14)
C(13)-N(1)-S(1)	113.60(18)	C(2)-C(1)-Bi(1)	113.69(18)
C(6)-C(1)-Bi(1)	128.7(2)	C(6)-C(1)-C(2)	117.5(2)
C(1)-C(2)-S(1)	114.13(19)	C(3)-C(2)-S(1)	122.2(2)
C(3)-C(2)-C(1)	123.7(2)	C(2)-C(3)-H(3)	121.3
C(2)-C(3)-C(4)	117.3(3)	C(4)-C(3)-H(3)	121.3
C(3)-C(4)-H(4)	119.9	C(5)-C(4)-C(3)	120.2(3)
C(5)-C(4)-H(4)	119.9	C(4)-C(5)-H(5)	119.4
C(4)-C(5)-C(6)	121.2(3)	C(6)-C(5)-H(5)	119.4
C(1)-C(6)-C(5)	120.0(3)	C(1)-C(6)-H(6)	120.0
C(5)-C(6)-H(6)	120.0	C(8)-C(7)-Bi(1)	112.77(17)
C(8)-C(7)-C(12)	118.0(2)	C(12)-C(7)-Bi(1)	129.2(2)
C(7)-C(8)-S(1)	116.25(19)	C(7)-C(8)-C(9)	124.0(2)
C(9)-C(8)-S(1)	119.7(2)	C(8)-C(9)-H(9)	121.6
C(8)-C(9)-C(10)	116.7(3)	C(10)-C(9)-H(9)	121.6
C(9)-C(10)-H(10)	119.9	C(11)-C(10)-C(9)	120.1(3)
C(11)-C(10)-H(10)	119.9	C(10)-C(11)-H(11)	119.0
C(10)-C(11)-C(12)	122.0(2)	C(12)-C(11)-H(11)	119.0
C(7)-C(12)-C(11)	119.1(3)	C(7)-C(12)-H(12)	120.5
C(11)-C(12)-H(12)	120.5	N(1)-C(13)-H(13A)	109.5
N(1)-C(13)-H(13B)	109.5	N(1)-C(13)-H(13C)	109.5
H(13A)-C(13)-H(13B)	109.5	H(13A)-C(13)-H(13C)	109.5
H(13B)-C(13)-H(13C)	109.5	C(15)-C(14)-B(1)	119.5(2)
C(19)-C(14)-C(15)	113.6(2)	C(19)-C(14)-B(1)	126.9(2)
F(2)-C(15)-C(14)	119.3(2)	F(2)-C(15)-C(16)	115.8(2)
C(16)-C(15)-C(14)	124.9(2)	F(3)-C(16)-C(15)	121.1(2)
F(3)-C(16)-C(17)	120.1(2)	C(15)-C(16)-C(17)	118.8(2)

F(4)-C(17)-C(16)	120.3(2)	F(4)-C(17)-C(18)	120.7(2)
C(18)-C(17)-C(16)	119.0(2)	F(5)-C(18)-C(17)	119.8(2)
F(5)-C(18)-C(19)	120.2(2)	C(17)-C(18)-C(19)	120.0(2)
F(6)-C(19)-C(14)	121.1(2)	F(6)-C(19)-C(18)	115.3(2)
C(18)-C(19)-C(14)	123.6(2)	C(21)-C(20)-B(1)	119.4(2)
C(25)-C(20)-C(21)	113.5(2)	C(25)-C(20)-B(1)	126.6(2)
F(7)-C(21)-C(20)	118.5(2)	F(7)-C(21)-C(22)	117.4(2)
C(22)-C(21)-C(20)	124.1(2)	F(8)-C(22)-C(21)	120.4(2)
F(8)-C(22)-C(23)	120.0(2)	C(21)-C(22)-C(23)	119.6(2)
F(9)-C(23)-C(22)	120.7(2)	F(9)-C(23)-C(24)	120.3(2)
C(24)-C(23)-C(22)	119.1(2)	F(10)-C(24)-C(23)	119.2(2)
F(10)-C(24)-C(25)	120.9(2)	C(23)-C(24)-C(25)	119.9(2)
F(11)-C(25)-C(20)	121.0(2)	F(11)-C(25)-C(24)	115.3(2)
C(24)-C(25)-C(20)	123.7(2)	C(27)-C(26)-B(1)	126.0(2)
C(31)-C(26)-C(27)	113.5(2)	C(31)-C(26)-B(1)	120.0(2)
F(12)-C(27)-C(26)	120.4(2)	F(12)-C(27)-C(28)	115.9(2)
C(28)-C(27)-C(26)	123.8(2)	F(13)-C(28)-C(27)	120.8(2)
F(13)-C(28)-C(29)	119.3(2)	C(29)-C(28)-C(27)	119.8(2)
F(14)-C(29)-C(28)	120.4(2)	F(14)-C(29)-C(30)	120.6(2)
C(28)-C(29)-C(30)	119.1(2)	F(15)-C(30)-C(29)	120.1(2)
F(15)-C(30)-C(31)	120.8(2)	C(31)-C(30)-C(29)	119.1(2)
F(16)-C(31)-C(26)	119.9(2)	F(16)-C(31)-C(30)	115.4(2)
C(30)-C(31)-C(26)	124.7(2)	F(1)-B(1)-C(14)	104.6(2)
F(1)-B(1)-C(20)	109.4(2)	F(1)-B(1)-C(26)	108.08(19)
C(14)-B(1)-C(26)	113.3(2)	C(20)-B(1)-C(14)	116.13(19)
C(20)-B(1)-C(26)	105.06(19)	C(32)-Bi(2)-F(17)	80.83(7)
C(32)-Bi(2)-C(38)	86.54(9)	C(38)-Bi(2)-F(17)	93.90(7)
O(2)-S(2)-N(2)	120.12(11)	O(2)-S(2)-C(33)	113.31(12)
O(2)-S(2)-C(39)	111.82(12)	N(2)-S(2)-C(33)	100.22(12)
N(2)-S(2)-C(39)	104.95(12)	C(33)-S(2)-C(39)	104.78(11)
B(2)-F(17)-Bi(2)	149.85(14)	C(44)-N(2)-S(2)	112.99(18)
C(33)-C(32)-Bi(2)	113.18(17)	C(33)-C(32)-C(37)	118.1(2)
C(37)-C(32)-Bi(2)	128.7(2)	C(32)-C(33)-S(2)	115.72(19)
C(32)-C(33)-C(34)	124.0(2)	C(34)-C(33)-S(2)	120.3(2)
C(33)-C(34)-H(34)	121.5	C(33)-C(34)-C(35)	117.1(2)
C(35)-C(34)-H(34)	121.5	C(34)-C(35)-H(35)	120.0
C(36)-C(35)-C(34)	120.0(2)	C(36)-C(35)-H(35)	120.0
C(35)-C(36)-H(36)	119.2	C(35)-C(36)-C(37)	121.7(2)

C(37)-C(36)-H(36)	119.2	C(32)-C(37)-H(37)	120.4
C(36)-C(37)-C(32)	119.1(2)	C(36)-C(37)-H(37)	120.4
C(39)-C(38)-Bi(2)	113.78(18)	C(39)-C(38)-C(43)	118.0(2)
C(43)-C(38)-Bi(2)	128.27(19)	C(38)-C(39)-S(2)	114.19(19)
C(40)-C(39)-S(2)	122.2(2)	C(40)-C(39)-C(38)	123.6(2)
C(39)-C(40)-H(40)	121.2	C(39)-C(40)-C(41)	117.5(3)
C(41)-C(40)-H(40)	121.2	C(40)-C(41)-H(41)	120.0
C(42)-C(41)-C(40)	120.0(3)	C(42)-C(41)-H(41)	120.0
C(41)-C(42)-H(42)	119.3	C(41)-C(42)-C(43)	121.4(3)
C(43)-C(42)-H(42)	119.3	C(38)-C(43)-H(43)	120.3
C(42)-C(43)-C(38)	119.4(2)	C(42)-C(43)-H(43)	120.3
N(2)-C(44)-H(44A)	109.5	N(2)-C(44)-H(44B)	109.5
N(2)-C(44)-H(44C)	109.5	H(44A)-C(44)-H(44B)	109.5
H(44A)-C(44)-H(44C)	109.5	H(44B)-C(44)-H(44C)	109.5
C(46)-C(45)-B(2)	119.4(2)	C(50)-C(45)-C(46)	113.0(2)
C(50)-C(45)-B(2)	127.6(2)	F(18)-C(46)-C(45)	119.4(2)
F(18)-C(46)-C(47)	115.7(2)	C(47)-C(46)-C(45)	124.9(3)
F(19)-C(47)-C(46)	120.7(2)	F(19)-C(47)-C(48)	120.0(2)
C(48)-C(47)-C(46)	119.3(2)	F(20)-C(48)-C(47)	120.7(2)
F(20)-C(48)-C(49)	120.3(2)	C(47)-C(48)-C(49)	119.1(2)
F(21)-C(49)-C(48)	119.9(2)	F(21)-C(49)-C(50)	120.8(2)
C(48)-C(49)-C(50)	119.3(2)	F(22)-C(50)-C(45)	120.7(2)
F(22)-C(50)-C(49)	114.8(2)	C(49)-C(50)-C(45)	124.5(2)
C(52)-C(51)-C(56)	113.3(2)	C(52)-C(51)-B(2)	124.5(2)
C(56)-C(51)-B(2)	122.0(2)	F(23)-C(52)-C(51)	120.4(2)
F(23)-C(52)-C(53)	115.4(2)	C(53)-C(52)-C(51)	124.2(2)
F(24)-C(53)-C(52)	120.7(3)	F(24)-C(53)-C(54)	120.1(2)
C(54)-C(53)-C(52)	119.2(3)	F(25)-C(54)-C(53)	120.3(3)
F(25)-C(54)-C(55)	120.1(3)	C(55)-C(54)-C(53)	119.7(2)
F(26)-C(55)-C(54)	120.2(2)	F(26)-C(55)-C(56)	120.5(2)
C(54)-C(55)-C(56)	119.3(3)	F(27)-C(56)-C(51)	118.9(2)
F(27)-C(56)-C(55)	116.8(2)	C(55)-C(56)-C(51)	124.2(2)
C(58)-C(57)-C(62)	113.8(2)	C(58)-C(57)-B(2)	126.5(2)
C(62)-C(57)-B(2)	119.6(2)	F(28)-C(58)-C(57)	121.2(2)
F(28)-C(58)-C(59)	115.2(2)	C(57)-C(58)-C(59)	123.6(2)
F(29)-C(59)-C(58)	120.4(2)	F(29)-C(59)-C(60)	119.5(2)
C(60)-C(59)-C(58)	120.0(2)	F(30)-C(60)-C(59)	120.2(2)
F(30)-C(60)-C(61)	120.8(2)	C(59)-C(60)-C(61)	119.0(2)

F(31)-C(61)-C(60)	120.0(2)	F(31)-C(61)-C(62)	121.1(2)
C(62)-C(61)-C(60)	118.9(2)	F(32)-C(62)-C(57)	119.6(2)
F(32)-C(62)-C(61)	115.8(2)	C(61)-C(62)-C(57)	124.6(2)
F(17)-B(2)-C(45)	106.27(19)	F(17)-B(2)-C(51)	107.6(2)
F(17)-B(2)-C(57)	105.9(2)	C(51)-B(2)-C(45)	109.7(2)
C(51)-B(2)-C(57)	114.3(2)	C(57)-B(2)-C(45)	112.6(2)
C(64)-C(63)-Cl(1)	120.0(5)	C(64)-C(63)-C(68)	120.8(6)
C(68)-C(63)-Cl(1)	119.2(5)	C(63)-C(64)-H(64)	120.3
C(63)-C(64)-C(65)	119.4(7)	C(65)-C(64)-H(64)	120.3
C(64)-C(65)-H(65)	119.9	C(66)-C(65)-C(64)	120.1(7)
C(66)-C(65)-H(65)	119.9	C(65)-C(66)-H(66)	120.0
C(67)-C(66)-C(65)	120.0(8)	C(67)-C(66)-H(66)	120.0
C(66)-C(67)-H(67)	120.0	C(66)-C(67)-C(68)	119.9(7)
C(68)-C(67)-H(67)	120.0	C(63)-C(68)-C(67)	119.6(7)
C(63)-C(68)-H(68)	120.2	C(67)-C(68)-H(68)	120.2
C(70)-C(69)-Cl(2)	119.2(5)	C(70)-C(69)-C(74)	121.6(5)
C(74)-C(69)-Cl(2)	119.2(5)	C(69)-C(70)-H(70)	120.4
C(69)-C(70)-C(71)	119.2(6)	C(71)-C(70)-H(70)	120.4
C(70)-C(71)-H(71)	120.1	C(70)-C(71)-C(72)	119.9(6)
C(72)-C(71)-H(71)	120.1	C(71)-C(72)-H(72)	119.9
C(73)-C(72)-C(71)	120.2(6)	C(73)-C(72)-H(72)	119.9
C(72)-C(73)-H(73)	119.9	C(74)-C(73)-C(72)	120.2(6)
C(74)-C(73)-H(73)	119.9	C(69)-C(74)-H(74)	120.6
C(73)-C(74)-C(69)	118.8(5)	C(73)-C(74)-H(74)	120.6

Symmetry transformations used to generate equivalent atoms: #1 -x+1,-y+1,z

8.2 Single crystal structure of [Bi-4]•B(Ar^F)$_3$OH

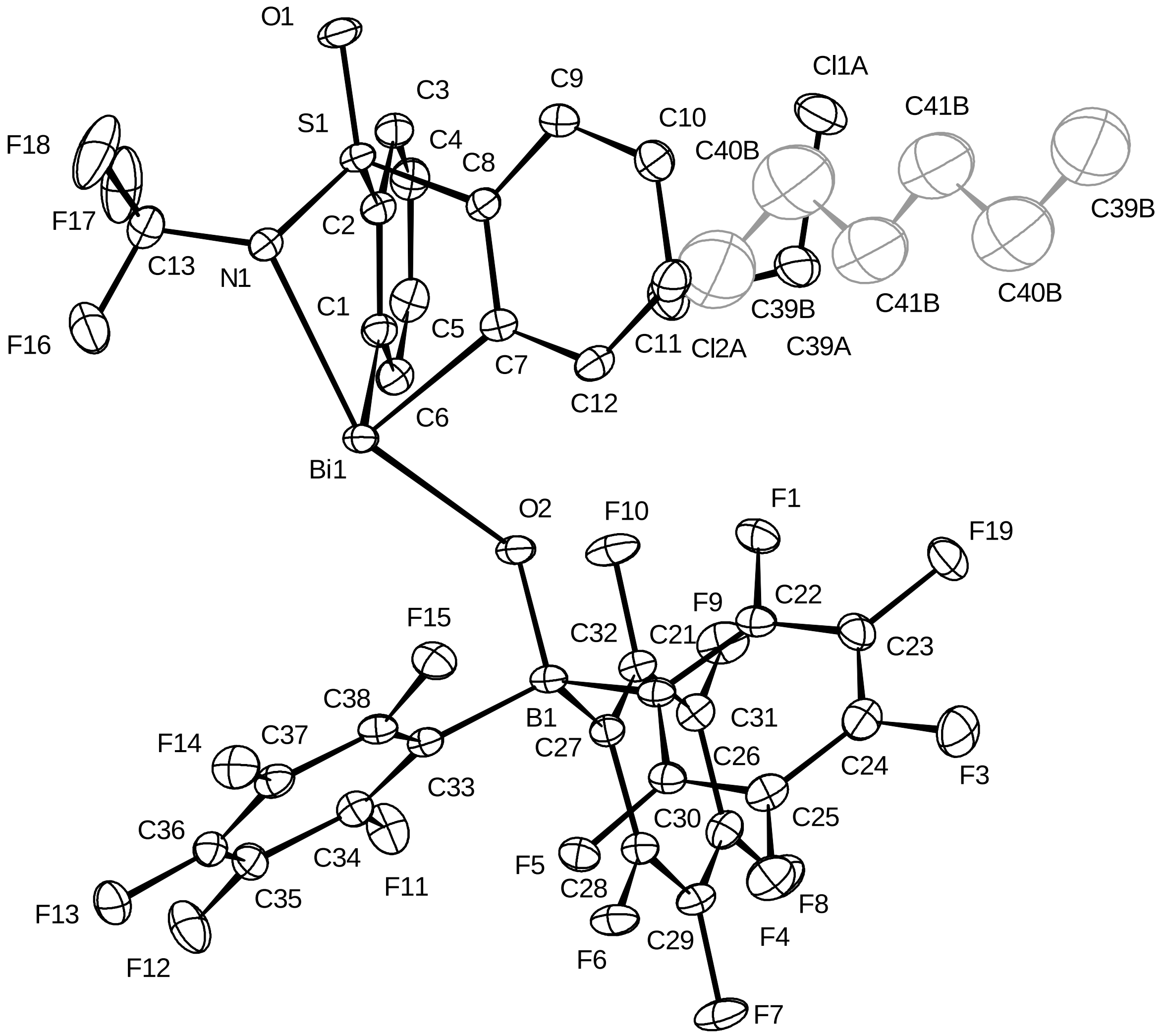

Figure S39. The molecular structure of **[Bi-4]•B(Ar^F)$_3$OH**. The H atoms have been omitted for clarity, and the minor disorder is shown in grey.

X-ray Crystal Structure Analysis of **[Bi-4]•B(Ar^F)$_3$OH**:

$C_{32.52}$ $H_{12.29}$ B Bi $Cl_{1.48}$ F_{18} N O_2 S, M_r = 1095.40 g mol^{-1}, colourless prism, crystal size 0.277 x 0.148 x 0.123 mm^3, triclinic, space group *P*-1 [2], a = 11.3306(13) Å, b = 11.6601(11) Å, c = 13.3998(9) Å, α = 92.266(4)°, β = 95.481(5)°, γ = 101.558(5)°, *V* = 1723.4(3) Å^3, *T* = 100(2) K, *Z* = 2, D_{calc} = 2.111 g·cm^3, λ = 0.71073 Å, μ(*Mo-K$_\alpha$*) = 5.424 mm^{-1}, Numerical correction (T_{min} = 0.4195, T_{max} = 0.9950), Bruker-AXS D8 Venture with Photon III detector and IµS Diamond microfocus Mo-anode X-ray source, 2.269 to 30.999°, 208549 measured reflections, 10977 independent reflections, 10651reflections with $I > 2\sigma(I)$, R_{int} = 0.0589. The structure was solved by *SHELXT* and refined by full-matrix least-squares (*SHELXL*) against F^2 to R_1 = 0.0156 [$I > 2\sigma(I)$], wR_2 = 0.0370 [all data], 556 parameters and 25 restraints.

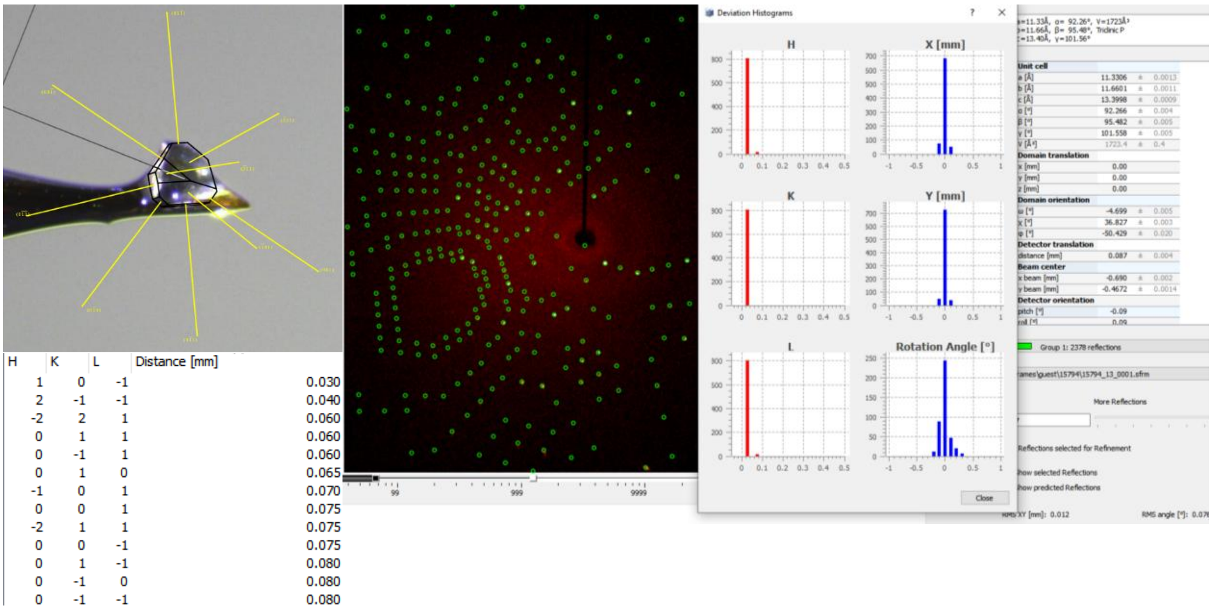

Figure S40. Crystal faces and unit cell determination/refinement of **[Bi-4]•B(Ar^{F})$_3$OH**.

INTENSITY STATISTICS FOR DATASET

Resolution	#Data	#Theory	%Complete	Redundancy	Mean I	Mean I/s	Rmerge	Rsigma
Inf - 2.62	373	400	93.2	5.90	157.06	32.73	0.0464	0.0561
2.62 - 1.76	879	930	94.5	8.93	109.79	42.60	0.0437	0.0412
1.76 - 1.40	1257	1276	98.5	11.90	79.07	48.93	0.0445	0.0251
1.40 - 1.23	1234	1236	99.8	11.48	59.54	46.63	0.0439	0.0213
1.23 - 1.12	1252	1252	100.0	11.16	51.39	44.23	0.0444	0.0201
1.12 - 1.04	1240	1240	100.0	11.00	45.06	42.27	0.0455	0.0197
1.04 - 0.98	1268	1268	100.0	10.37	39.22	38.99	0.0514	0.0211
0.98 - 0.93	1302	1302	100.0	10.04	33.01	36.67	0.0540	0.0226
0.93 - 0.89	1268	1268	100.0	9.74	27.32	33.60	0.0624	0.0246
0.89 - 0.85	1500	1500	100.0	9.53	24.06	30.62	0.0650	0.0263
0.85 - 0.82	1314	1314	100.0	9.32	22.54	29.40	0.0690	0.0279
0.82 - 0.80	984	984	100.0	9.21	19.85	26.70	0.0725	0.0299
0.80 - 0.78	1132	1132	100.0	9.02	19.53	25.84	0.0743	0.0310
0.78 - 0.76	1184	1184	100.0	8.63	17.35	23.63	0.0790	0.0340
0.76 - 0.74	1404	1404	100.0	8.53	16.63	22.94	0.0831	0.0357
0.74 - 0.72	1500	1500	100.0	8.08	15.10	20.67	0.0903	0.0398
0.72 - 0.71	840	840	100.0	8.06	13.59	19.64	0.0996	0.0426
0.71 - 0.69	1758	1758	100.0	7.92	12.33	18.25	0.1024	0.0459
0.69 - 0.68	994	994	100.0	7.92	11.71	17.60	0.1100	0.0484
0.68 - 0.67	1004	1004	100.0	7.51	10.94	15.95	0.1158	0.0528
0.67 - 0.66	1178	1366	86.2	5.88	10.30	14.37	0.1215	0.0629
0.76 - 0.66	8678	8866	97.9	7.70	13.12	18.69	0.0991	0.0450
Inf - 0.66	24865	25152	98.9	9.16	32.87	30.02	0.0588	0.0312

Complete .cif-data are available under the CCDC number CCDC-**2504151**.

Four reflections (0 -1 1; 1 0 0; 1 0 1; 1 0 4) were presumably affected by the primary beam stop and were therefore excluded from the dataset prior to the final refinement cycles. Additionally, a SHEL card (999 0.69) was inserted to eliminate incomplete data at high diffraction angles.

The 'pentane' fragment from the DSR tool[27, 28] in Olex2[29], was used to describe the disordered part containing the solute.

The presence of positive residual electron density in the vicinity of the oxygen atom was identified during the later refinement cycles. This was then described by a free, refined hydrogen atom. Once the final refinement cycles had been completed, the model had converged and the residual electron density at that point had disappeared.

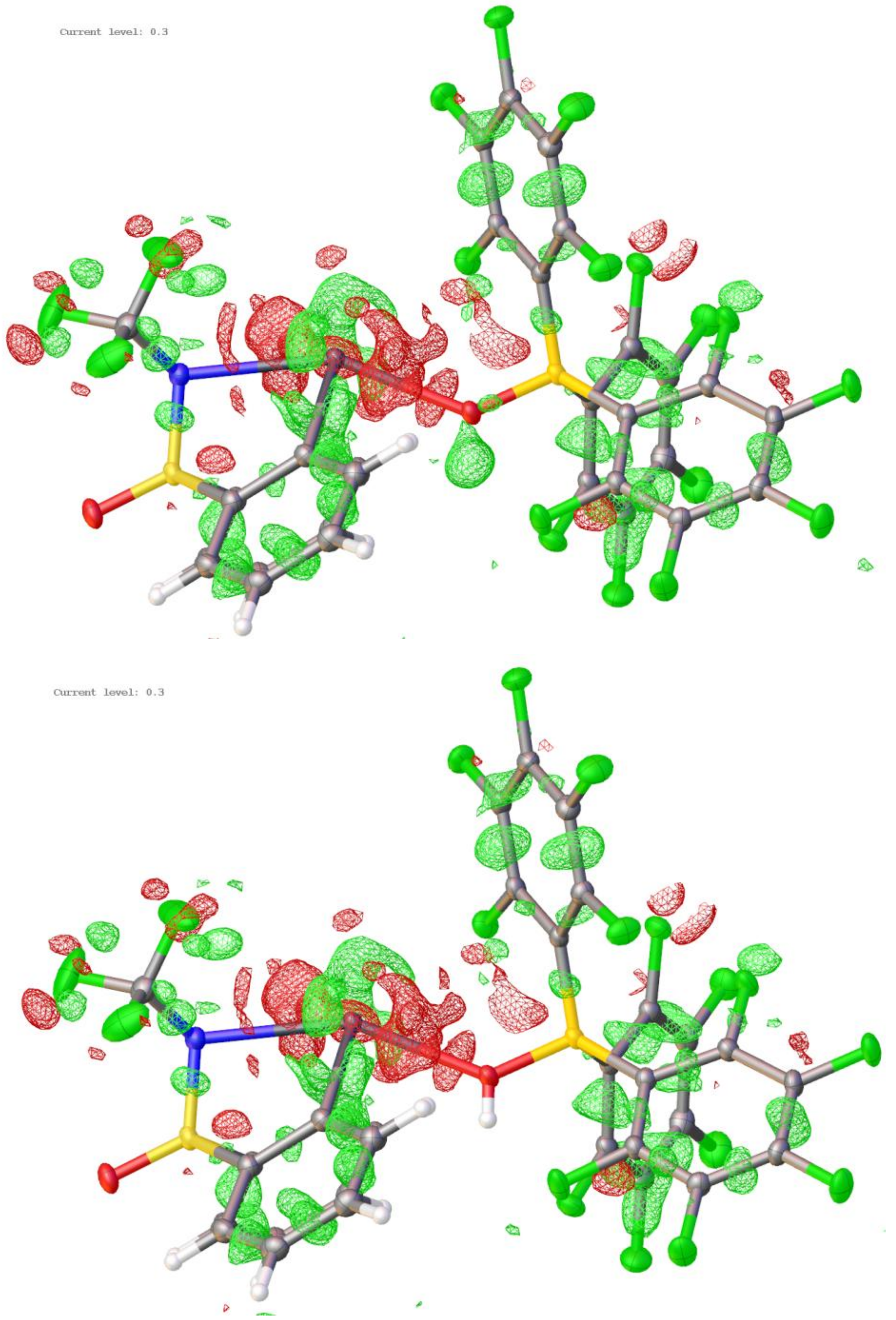

Figure S41. Structure and difference density distribution of compound **[Bi-4]•B(Ar^F)$_3$OH** (source: Olex2, diff-map, level 0.3 e/Å3, 0.1 Å resolution) indicating the presence of a H atom attached to the O atom. The model is shown above without the H atom, and below with the H atom bridging the O atom.

Table 3. Crystal data and structure refinement **[Bi-4]•B(Ar^F)$_3$OH.**

Identification code	15794	
Empirical formula	$C_{32.52} H_{12.29} B Bi Cl_{1.48} F_{18} N O_2 S$	
Color	colourless	
Formula weight	1095.40 g·mol^{-1}	
Temperature	100(2) K	
Wavelength	0.71073 Å	
Crystal system	Triclinic	
Space group	*P*-1, (no. 2)	
Unit cell dimensions	a = 11.3306(13) Å	α= 92.266(4)°.
	b = 11.6601(11) Å	β= 95.481(5)°.
	c = 13.3998(9) Å	γ = 101.558(5)°.
Volume	1723.4(3) Å^3	
Z	2	
Density (calculated)	2.111 mg·m^{-3}	
Absorption coefficient	5.424 mm^{-1}	
F(000)	1043 e	
Crystal size	0.277 x 0.148 x 0.123 mm^3	
θ range for data collection	2.269 to 30.999°.	
Index ranges	$-16 \le h \le 16$, $-16\le k \le 16$, $-19 \le l \le 19$	
Reflections collected	208549	
Independent reflections	10977 [R_{int} = 0.0589]	
Reflections with I>2σ(I)	10651	
Completeness to θ = 25.242°	99.9%	
Absorption correction	Numerical	
Max. and min. transmission	0.9950 and 0.4195	
Refinement method	Full-matrix least-squares on F^2	
Data / restraints / parameters	10977 / 25 / 556	
Goodness-of-fit on F^2	1.065	
Final R indices [I>2σ(I)]	R_1 = 0.0156	wR^2 = 0.0368
R indices (all data)	R_1 = 0.0163	wR^2 = 0.0370
Extinction coefficient	n/a	
Largest diff. peak and hole	0.788 and -0.710 e·Å^{-3}	

Table 4. Bond lengths [Å] and angles [°] **[Bi-4]•B(Ar^F)$_3$OH.**

Bi(1)-N(1)	2.5316(12)	Bi(1)-O(2)	2.3305(10)
Bi(1)-C(1)	2.2596(14)	Bi(1)-C(7)	2.2520(14)
S(1)-O(1)	1.4388(11)	S(1)-N(1)	1.5856(13)
S(1)-C(2)	1.7731(14)	S(1)-C(8)	1.7623(14)
F(1)-C(22)	1.3600(16)	F(19)-C(23)	1.3446(17)
F(3)-C(24)	1.3427(17)	F(4)-C(25)	1.3507(17)
F(5)-C(26)	1.3518(16)	F(6)-C(28)	1.3529(16)
F(7)-C(29)	1.3433(16)	F(8)-C(30)	1.3424(16)
F(9)-C(31)	1.3430(16)	F(10)-C(32)	1.3581(15)
F(11)-C(34)	1.3464(15)	F(12)-C(35)	1.3417(16)
F(13)-C(36)	1.3367(16)	F(14)-C(37)	1.3486(16)
F(15)-C(38)	1.3635(15)	F(16)-C(13)	1.324(2)
F(17)-C(13)	1.3290(19)	F(18)-C(13)	1.3383(19)
N(1)-C(13)	1.4086(18)	O(2)-B(1)	1.5255(17)
O(2)-H(2)	0.69(3)	C(1)-C(2)	1.4017(19)
C(1)-C(6)	1.3923(19)	C(2)-C(3)	1.3900(19)
C(3)-H(3)	0.9500	C(3)-C(4)	1.399(2)
C(4)-H(4)	0.9500	C(4)-C(5)	1.394(2)
C(5)-H(5)	0.9500	C(5)-C(6)	1.402(2)
C(6)-H(6)	0.9500	C(7)-C(8)	1.3951(19)
C(7)-C(12)	1.3942(19)	C(8)-C(9)	1.391(2)
C(9)-H(9)	0.9500	C(9)-C(10)	1.395(2)
C(10)-H(10)	0.9500	C(10)-C(11)	1.393(2)
C(11)-H(11)	0.9500	C(11)-C(12)	1.400(2)
C(12)-H(12)	0.9500	C(21)-C(22)	1.3998(19)
C(21)-C(26)	1.3916(19)	C(21)-B(1)	1.660(2)
C(22)-C(23)	1.384(2)	C(23)-C(24)	1.383(2)
C(24)-C(25)	1.378(2)	C(25)-C(26)	1.389(2)
C(27)-C(28)	1.3965(18)	C(27)-C(32)	1.3870(18)
C(27)-B(1)	1.650(2)	C(28)-C(29)	1.3888(19)
C(29)-C(30)	1.385(2)	C(30)-C(31)	1.380(2)
C(31)-C(32)	1.3903(19)	C(33)-C(34)	1.3959(19)
C(33)-C(38)	1.3966(18)	C(33)-B(1)	1.639(2)
C(34)-C(35)	1.391(2)	C(35)-C(36)	1.381(2)
C(36)-C(37)	1.383(2)	C(37)-C(38)	1.386(2)
Cl(1A)-C(39A)	1.767(3)	Cl(2A)-C(39A)	1.761(2)

C(39A)-H(39D)	0.9900	C(39A)-H(39E)	0.9900
C(39B)-H(39A)	0.9800	C(39B)-H(39B)	0.9800
C(39B)-H(39C)	0.9800	C(39B)-C(40B)	1.509(14)
C(40B)-H(40A)	0.9900	C(40B)-H(40B)	0.9900
C(40B)-C(41B)	1.517(12)	C(41B)-C(41B)#1	1.47(3)
C(41B)-H(41A)	0.9900	C(41B)-H(41B)	0.9900
O(2)-Bi(1)-N(1)	152.16(4)	C(1)-Bi(1)-N(1)	75.76(5)
C(1)-Bi(1)-O(2)	86.07(4)	C(7)-Bi(1)-N(1)	72.48(4)
C(7)-Bi(1)-O(2)	86.38(4)	C(7)-Bi(1)-C(1)	88.65(5)
O(1)-S(1)-N(1)	120.44(7)	O(1)-S(1)-C(2)	112.37(7)
O(1)-S(1)-C(8)	113.28(7)	N(1)-S(1)-C(2)	105.61(7)
N(1)-S(1)-C(8)	98.23(7)	C(8)-S(1)-C(2)	105.06(6)
S(1)-N(1)-Bi(1)	99.71(6)	C(13)-N(1)-Bi(1)	119.57(9)
C(13)-N(1)-S(1)	118.38(10)	Bi(1)-O(2)-H(2)	111(2)
B(1)-O(2)-Bi(1)	133.05(8)	B(1)-O(2)-H(2)	116(2)
C(2)-C(1)-Bi(1)	116.99(10)	C(6)-C(1)-Bi(1)	124.84(10)
C(6)-C(1)-C(2)	118.15(13)	C(1)-C(2)-S(1)	115.10(10)
C(3)-C(2)-S(1)	121.39(11)	C(3)-C(2)-C(1)	123.50(13)
C(2)-C(3)-H(3)	121.4	C(2)-C(3)-C(4)	117.23(13)
C(4)-C(3)-H(3)	121.4	C(3)-C(4)-H(4)	119.7
C(5)-C(4)-C(3)	120.65(13)	C(5)-C(4)-H(4)	119.7
C(4)-C(5)-H(5)	119.6	C(4)-C(5)-C(6)	120.88(14)
C(6)-C(5)-H(5)	119.6	C(1)-C(6)-C(5)	119.58(13)
C(1)-C(6)-H(6)	120.2	C(5)-C(6)-H(6)	120.2
C(8)-C(7)-Bi(1)	115.51(9)	C(12)-C(7)-Bi(1)	126.57(10)
C(12)-C(7)-C(8)	117.91(13)	C(7)-C(8)-S(1)	117.23(10)
C(9)-C(8)-S(1)	118.83(11)	C(9)-C(8)-C(7)	123.93(13)
C(8)-C(9)-H(9)	121.4	C(8)-C(9)-C(10)	117.14(14)
C(10)-C(9)-H(9)	121.4	C(9)-C(10)-H(10)	119.8
C(11)-C(10)-C(9)	120.33(14)	C(11)-C(10)-H(10)	119.8
C(10)-C(11)-H(11)	119.3	C(10)-C(11)-C(12)	121.32(14)
C(12)-C(11)-H(11)	119.3	C(7)-C(12)-C(11)	119.36(13)
C(7)-C(12)-H(12)	120.3	C(11)-C(12)-H(12)	120.3
F(16)-C(13)-F(17)	108.05(15)	F(16)-C(13)-F(18)	106.47(15)
F(16)-C(13)-N(1)	109.24(13)	F(17)-C(13)-F(18)	105.31(14)
F(17)-C(13)-N(1)	115.43(13)	F(18)-C(13)-N(1)	111.89(13)
C(22)-C(21)-B(1)	119.26(12)	C(26)-C(21)-C(22)	113.47(12)

C(26)-C(21)-B(1)	126.80(12)	F(1)-C(22)-C(21)	119.25(12)
F(1)-C(22)-C(23)	116.08(12)	C(23)-C(22)-C(21)	124.66(13)
F(19)-C(23)-C(22)	121.11(14)	F(19)-C(23)-C(24)	119.83(13)
C(24)-C(23)-C(22)	119.06(13)	F(3)-C(24)-C(23)	120.45(14)
F(3)-C(24)-C(25)	120.54(14)	C(25)-C(24)-C(23)	119.00(13)
F(4)-C(25)-C(24)	119.70(13)	F(4)-C(25)-C(26)	120.21(13)
C(24)-C(25)-C(26)	120.08(13)	F(5)-C(26)-C(21)	120.89(12)
F(5)-C(26)-C(25)	115.38(12)	C(25)-C(26)-C(21)	123.72(13)
C(28)-C(27)-B(1)	119.73(11)	C(32)-C(27)-C(28)	113.64(12)
C(32)-C(27)-B(1)	126.43(12)	F(6)-C(28)-C(27)	118.96(12)
F(6)-C(28)-C(29)	116.59(12)	C(29)-C(28)-C(27)	124.43(13)
F(7)-C(29)-C(28)	121.12(13)	F(7)-C(29)-C(30)	119.76(12)
C(30)-C(29)-C(28)	119.12(12)	F(8)-C(30)-C(29)	120.15(13)
F(8)-C(30)-C(31)	120.91(13)	C(31)-C(30)-C(29)	118.93(12)
F(9)-C(31)-C(30)	120.26(12)	F(9)-C(31)-C(32)	119.96(12)
C(30)-C(31)-C(32)	119.78(13)	F(10)-C(32)-C(27)	121.12(12)
F(10)-C(32)-C(31)	114.80(12)	C(27)-C(32)-C(31)	124.07(12)
C(34)-C(33)-C(38)	113.21(12)	C(34)-C(33)-B(1)	125.00(12)
C(38)-C(33)-B(1)	121.73(12)	F(11)-C(34)-C(33)	120.78(12)
F(11)-C(34)-C(35)	114.84(12)	C(35)-C(34)-C(33)	124.36(13)
F(12)-C(35)-C(34)	120.41(13)	F(12)-C(35)-C(36)	120.04(13)
C(36)-C(35)-C(34)	119.53(13)	F(13)-C(36)-C(35)	120.98(13)
F(13)-C(36)-C(37)	120.29(13)	C(35)-C(36)-C(37)	118.73(13)
F(14)-C(37)-C(36)	119.36(13)	F(14)-C(37)-C(38)	120.84(13)
C(36)-C(37)-C(38)	119.79(13)	F(15)-C(38)-C(33)	119.53(12)
F(15)-C(38)-C(37)	116.17(12)	C(37)-C(38)-C(33)	124.29(12)
O(2)-B(1)-C(21)	109.54(10)	O(2)-B(1)-C(27)	111.47(11)
O(2)-B(1)-C(33)	103.62(11)	C(27)-B(1)-C(21)	103.01(10)
C(33)-B(1)-C(21)	115.07(11)	C(33)-B(1)-C(27)	114.29(11)
Cl(1A)-C(39A)-H(39D)	109.3	Cl(1A)-C(39A)-H(39E)	109.3
Cl(2A)-C(39A)-Cl(1A)	111.78(14)	Cl(2A)-C(39A)-H(39D)	109.3
Cl(2A)-C(39A)-H(39E)	109.3	H(39D)-C(39A)-H(39E)	107.9
H(39A)-C(39B)-H(39B)	109.5	H(39A)-C(39B)-H(39C)	109.5
H(39B)-C(39B)-H(39C)	109.5	C(40B)-C(39B)-H(39A)	109.5
C(40B)-C(39B)-H(39B)	109.5	C(40B)-C(39B)-H(39C)	109.5
C(39B)-C(40B)-H(40A)	110.2	C(39B)-C(40B)-H(40B)	110.2
C(39B)-C(40B)-C(41B)	107.5(12)	H(40A)-C(40B)-H(40B)	108.5
C(41B)-C(40B)-H(40A)	110.2	C(41B)-C(40B)-H(40B)	110.2

C(40B)-C(41B)-H(41A)	110.0	C(40B)-C(41B)-H(41B)	110.0
C(41B)#1-C(41B)-C(40B)	108.2(13)	C(41B)#1-C(41B)-H(41A)	110.0
C(41B)#1-C(41B)-H(41B)	110.0	H(41A)-C(41B)-H(41B)	108.4

Symmetry transformations used to generate equivalent atoms:

#1 -x,-y,-z+1

8.3 Database survey and structural discussions

A database (WebCSD Version 1.9.61, https://www.ccdc.cam.ac.uk/structures/WebCSD/StructureSearch) survey was performed on 28th of June 2025 to search for related structural motives. The following search motive was used:

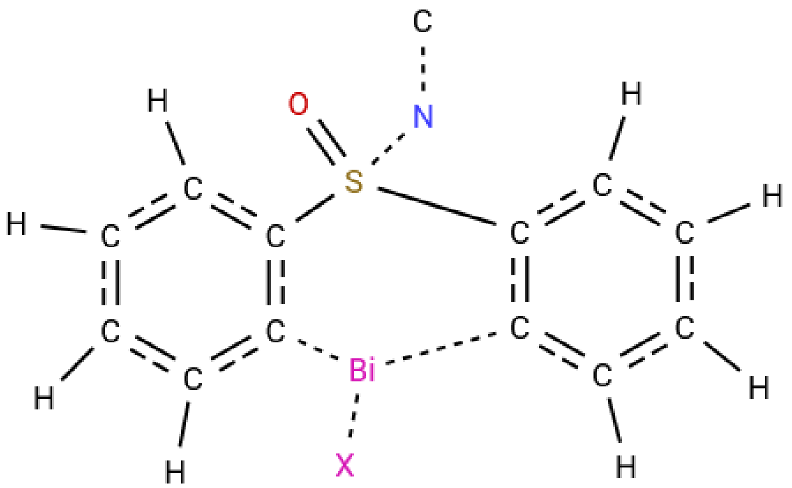

Figure S42. Search motif for data survey on related Bi complexes

A total of 14 related structures were found. These consist of a similar backbone structure and show variations in substitution patterns. The main functionalities identified are N-CF_3 and N-CH_3. Some of the structures also have an aryl ligand or different anions near the central Bi atom.

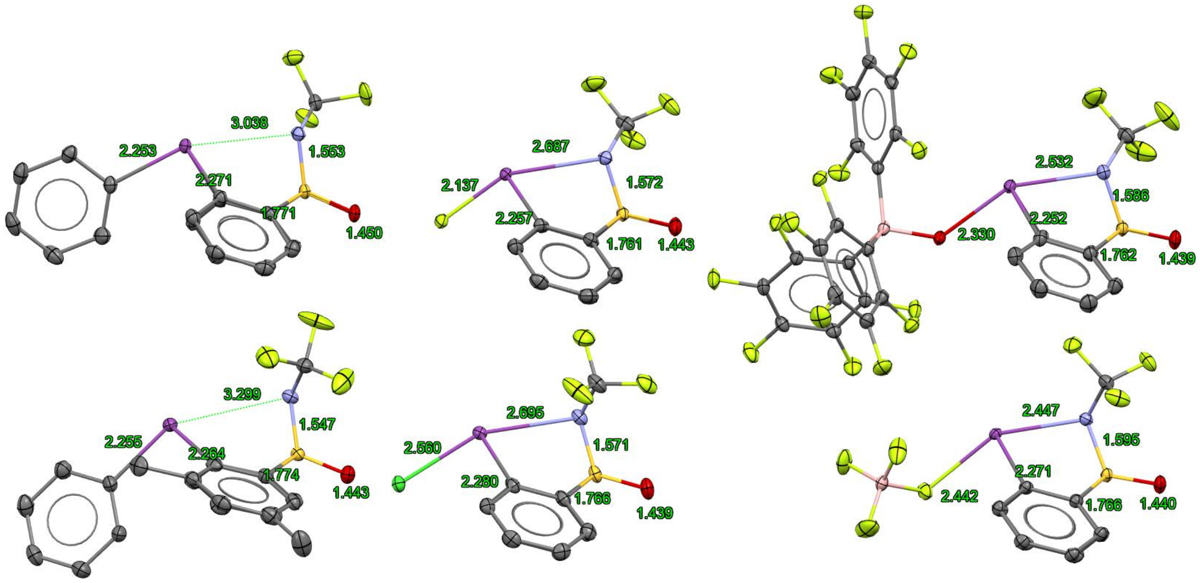

Figure S43. The -CF3-substituted structures found with selected distances. Left top: FUBLAS; left bottom: FUBLEW, middle top: FUBLIA; middle bottom: FUBLOG; right top: [Bi-4]•B(ArF)3OH; right bottom: FUBLUM.

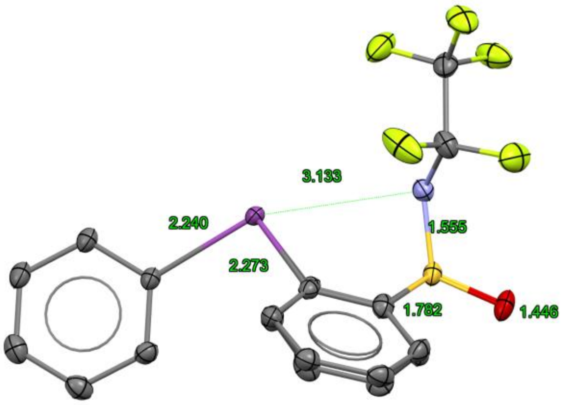

Figure S44. The only structure with a perfluorinated ethyl chain is PEHDIT and its selected distances.

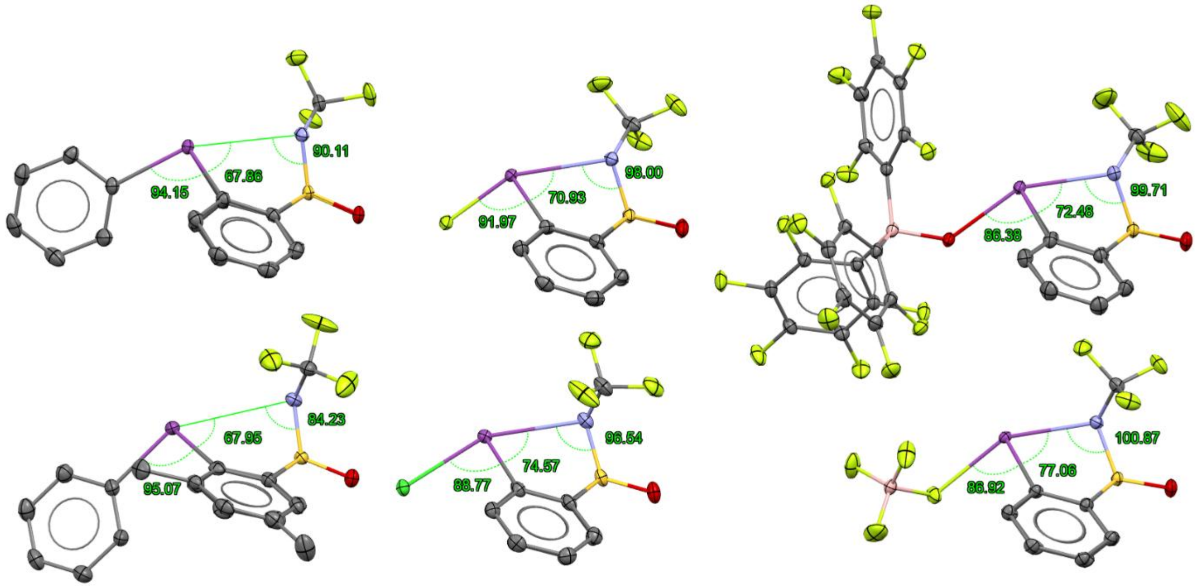

Figure S45. The CF_3-substituted structures found with selected angles. Left top: FUBLAS; left bottom: FUBLEW, middle top: FUBLIA; middle bottom: FUBLOG; right top: **[Bi-4]•B(ArF)3OH**; right bottom: FUBLUM.

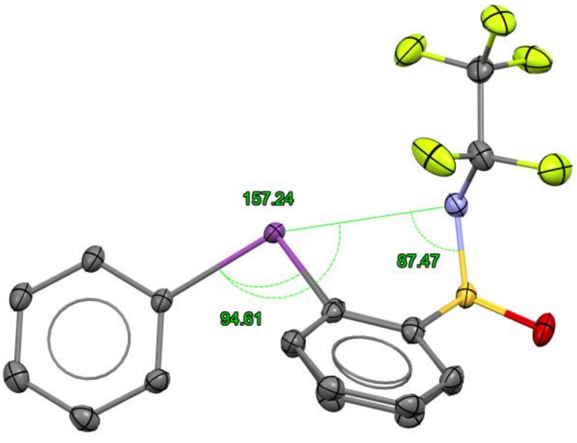

Figure S46. The only structure with a perfluorinated ethyl chain is PEHDIT and its selected angles.

The transannular distance between Bi and the N atom of the sulfonamide group is an indirect parameter of the electronic environment of the central Bi atom. Examining the structures presented in this study reveals some

interesting details. Firstly, the distances differ between structures with a third Bi-C bond (aryl-substituted) and ionic species. Purely covalently bonded aryl species have Bi···N distances between 3.038 and 3.299 Å, whereas ionic species (e.g. with Cl or F at Bi) have significantly shorter distances ranging from 2.447 to 2.687 Å. The Bi-N-S angle is influenced similarly. In pure aryl species, this angle is relatively small. In ionic species, however, it is correspondingly flatter, exceeding 90°. These two geometric properties demonstrate electron density donation from the sulfonamide nitrogen to the positive Bi(III) central atom.

Comparing the ionic structures reveals that the weakly coordinating anions ($[BF_4]^-$ and $[B(Ar^F)_3OH]^-$) exhibit an even shorter Bi···N distance (2.532 and 2.447 Å, respectively) than the analogous F- and Cl-containing structures (2.687 and 2.695 Å, respectively). This suggests that there is stronger electron density donation from the sulfonamide N to the central Bi in these structures with weakly coordinating anions.

A final comparison of the **[Bi-4]•B(Ar^F)$_3$OH** hydroxytris(perfluorophenyl)-λ^4-borane and **[Bi-3]•B(Ar^F)$_3$F** fluorotris(perfluorophenyl)borate structures reveals slight differences. Notably, the Bi···N distance is an important parameter once again, differing by 0.193 Å between the two structures. The Bi···N distance in **[Bi-3]•B(Ar^F)$_3$F**, at 2.339 Å, is the shortest of all the structures compared. Due to the increased electron density compared to the -CF_3 (electron-withdrawing group) substitution of the sulfonamide, the donor properties appear to be optimal here.

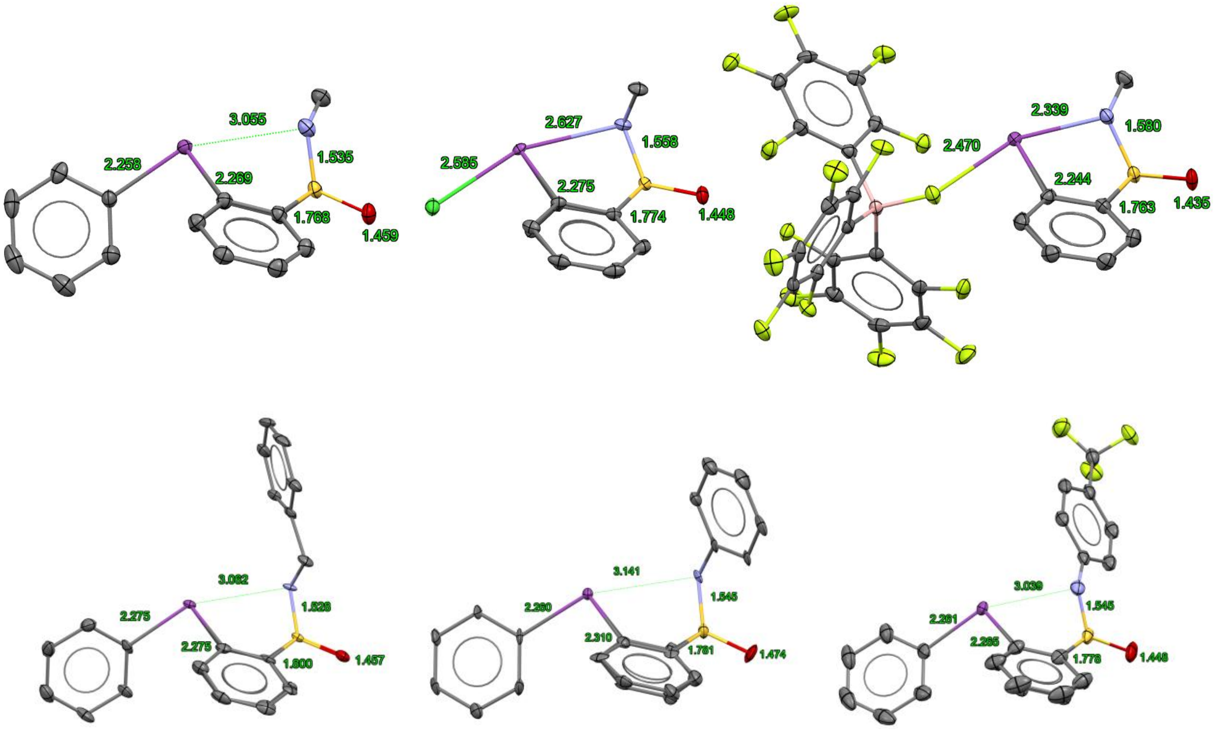

Figure S47. The CH_3- and aryl- substituted structures found with selected distances. Top left: FUBKUL; Top middle: EZESOU; Top right: **[Bi-3]•B(Ar^F)$_3$F**; bottom left: BUNXOB; bottom middle: BUNXAN; bottom right: PEGZOU.

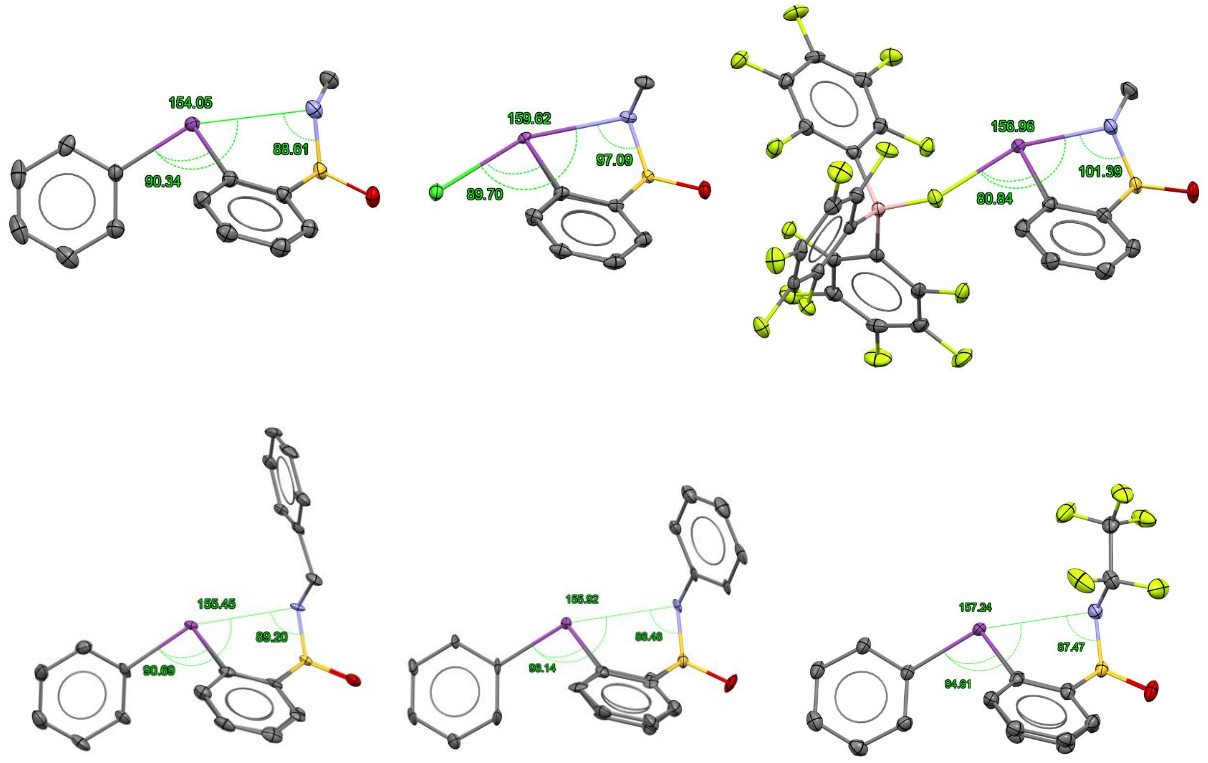

Figure S48. The CH_3- and aryl- substituted structures found with selected angles. Top left: FUBKUL; Top middle: EZESOU; Top right: **[Bi-3]•B(Ar^F)$_3$F**; bottom left: BUNXOB; bottom middle: BUNXAN; bottom right: PEGZOU.

A further database (WebCSD Version 1.9.61, https://www.ccdc.cam.ac.uk/structures/WebCSD/StructureSearch) survey was performed on 19th of November 2025 to search for **B(Ar^F)$_3$F** containing compounds. The following search motive was used:

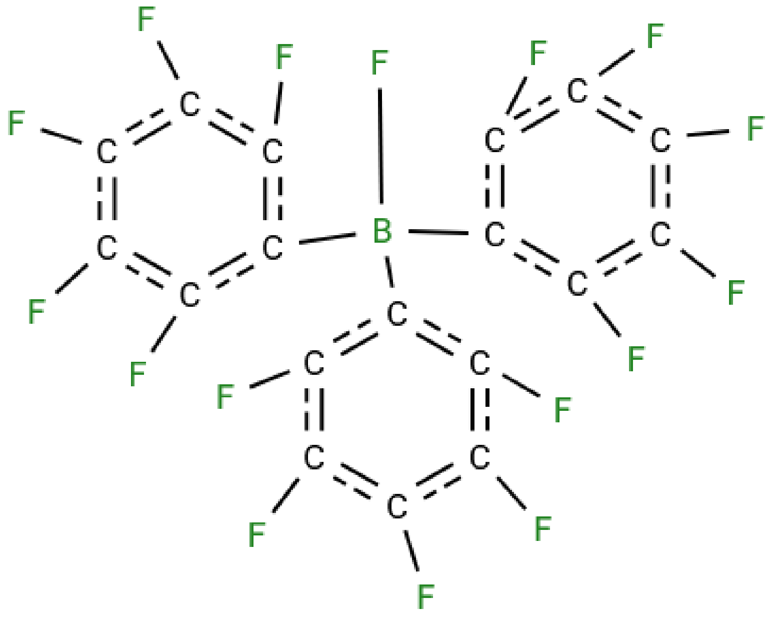

Figure S49: Graphical representation of the B-F distances determined from the database search. [Bi$_3$]•B(ArF)$_3$F is indicated by a red data point.

The B-F distances where selected as a relevant 3D parameter (DIST1 (D)). A total of 31 related structures were found.

**Refcode**	**DIST1 (D)**	**Refcode**	**DIST1 (D)**
UFITUB	1,413	GAKTUK	1,44
ZAFYOX	1,418	RUBNUZ	1,442
JUDXUD	1,42	RUBPIP	1,444
SILKUV	1,421	CEZHOI	1,447
XITDIP	1,422	IMUCEB	1,447
GEKCUY	1,427	IMUCEB	1,451
JUFWOY	1,427	TAHTUT	1,455
LAZBET	1,428	IMUCEB	1,46
LENBEN	1,429	IMUCEB	1,466
YISLAR	1,43	IMUCEB	1,466
MAQKIB	1,431	IKORUZ	1,47
MAPWOR	1,432	IMUCEB	1,47
XOHVIC	1,432	URAFOL	1,472
RUBPAH	1,433	BOCDAA	1,476
UFITOV	1,433	**[Bi-3]•B(Ar^{F})$_3$F**	1,477
EMAMIR	1,434	QUGQOB	1,499
IYOHUA	1,434	IMUCEB	1,503
MAPWIL	1,435	GIZCAW	1,512
RILVUG	1,435	IMUCEB	1,524
IMUCEB	1,436	IMUCEB	1,578
WIPQAQ	1,438	IMUCEB	1,616
IMUCEB	1,439	IYOHUA01	1,623
average distance		1,458	
standard deviation		0,048	

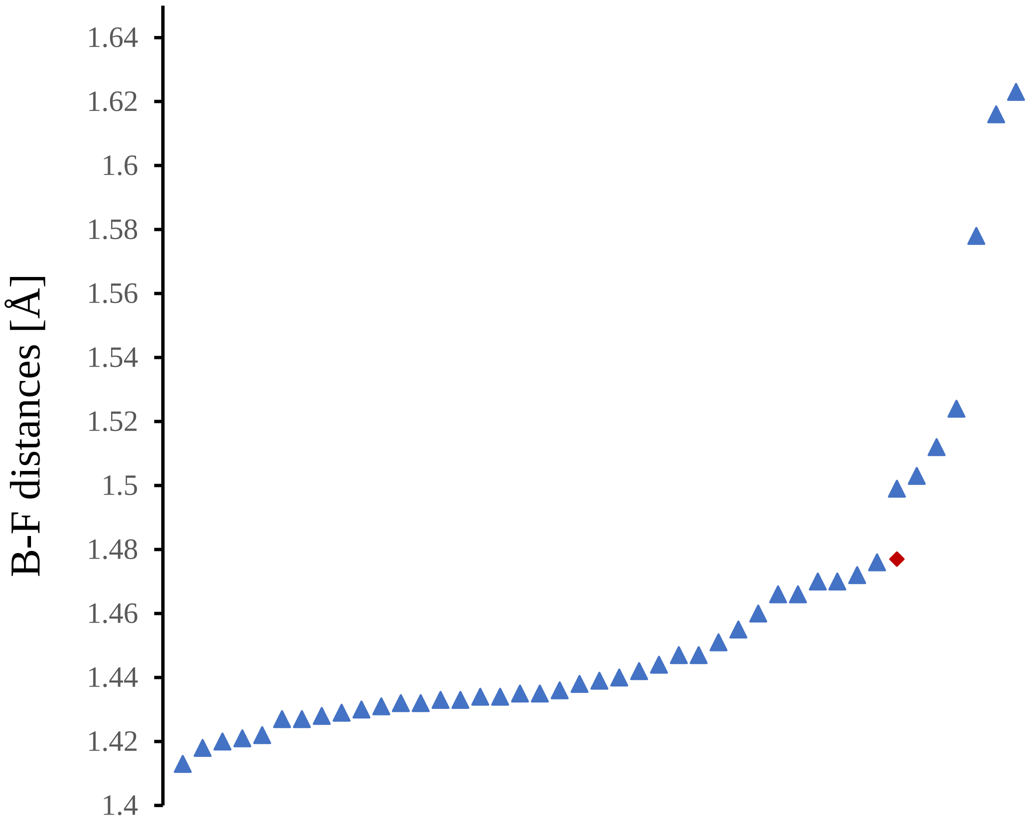

Figure S50: Graphical representation of the B-F distances determined from the database search. **[Bi-3]•B(Ar^{F})$_3$F** is indicated by a red data point.

9 References

[1] Williams, D. B. G.; Lawton, M. Drying of Organic Solvents: Quantitative Evaluation of the Efficiency of Several Desiccants. *J. Org. Chem.* **2010**, *75*, 8351–8354.

[2] Magre, M.; Cornella, J. Redox-Neutral Organometallic Elementary Steps at Bismuth: Catalytic Synthesis of Aryl Sulfonyl Fluorides. *J. Am. Chem. Soc.* **2021**, *143*, 21497–21502.

[3] Planas, O.; Wang, F.; Leutzsch, M.; Cornella, J. Fluorination of Arylboronic Esters Enabled by Bismuth Redox Catalysis. *Science* **2020**, *367*, 313–317.

[4] Wang, H.; Wu, J.; Noble, A.; Aggarwal, V. K. Selective Coupling of 1,2-Bis-Boronic Esters at the More Substituted Site through Visible-Light Activation of Electron Donor–Acceptor Complexes. *Angew. Chem. Int. Ed.* **2022**, *61*, e202202061.

[5] Castro-Godoy, W. D.; Schmidt, L. C.; Argüello, J. E. A Green Alternative for the Conversion of Arylboronic Acids/Esters into Phenols Promoted by a Reducing Agent, Sodium Sulfite. *Eur. J. Org. Chem.* **2019**, *2019*, 3035–3039.

[6] Lovinger, G. J.; Morken, J. P. Ni-Catalyzed Enantioselective Conjunctive Coupling with C(sp^3) Electrophiles: A Radical–Ionic Mechanistic Dichotomy. *J. Am. Chem. Soc.* **2017**, *139*, 17293–17296.

[7] Fuentes-Rivera, J. J.; Zick, M. E.; Düfert, M. A.; Milner, P. J. Overcoming Halide Inhibition of Suzuki–Miyaura Couplings with Biaryl Monophosphine-Based Catalysts. *Org. Process Res. Dev.* **2019**, *23*, 1631–1637.

[8] Prakash, G. K. S.; Pertusati, F.; Olah, G. A. HF-Free, Direct Synthesis of Tetrabutylammonium Trifluoroborates. *Synthesis* **2011**, *2011*, 292–302.

[9] Peramo, A.; Abdellah, I.; Pecnard, S.; Mougin, J.; Martini, C.; Couvreur, P.; Huc, V.; Desmaële, D. A Self-Assembling NHC-Pd-Loaded Calixarene as a Potent Catalyst for the Suzuki–Miyaura Cross-Coupling Reaction in Water. *Molecules* **2020**, *25*, 1459.

[10] Procter, R. J.; Dunsford, J. J.; Rushworth, P. J.; Hulcoop, D. G.; Layfield, R. A.; Ingleson, M. J. A Zinc-Catalyzed C(sp^3)–C(sp^2) Suzuki–Miyaura Cross-Coupling Reaction Mediated by Aryl-Zincates. *Chem. Eur. J.* **2017**, *23*, 15889–15893.

[11] Faber, T.; Engelhardt, S.; Cornella, J. Aryl Silicon Nucleophiles in Bismuth Catalysis. *Angew. Chem. Int. Ed.* **2025**, *64*, e202424698.

[12] Bardin, V. V.; Prikhod'ko, S. A.; Shmakov, M. M.; Shabalin, A. Y.; Adonin, N. Y. Synthesis of Fluorine-Containing Aryl(halo)boranes from Potassium Aryl(fluoro)borates. *Russ. J. Gen. Chem.* **2020**, *90*, 50–61.

[13] Lesnichin, S. B.; Shenderovich, I. G.; Muljati, T.; Silverman, D.; Limbach, H.-H. Intrinsic Proton-Donating Power of Zinc-Bound Water in a Carbonic Anhydrase Active Site Model Estimated by NMR. *J. Am. Chem. Soc.* **2011**, *133*, 11331–11338.

[14] Neese, F. The ORCA Program System. *WIREs Comput. Mol. Sci.* **2012**, *2*, 73–78.

[15] Neese, F. Software Update: The ORCA Program System—Version 5.0. *WIREs Comput. Mol. Sci.* **2022**, *12*, e1606.

[16] Neese, F. Software Update: The ORCA Program System—Version 6.0. *WIREs Comput. Mol. Sci.* **2025**, *15*, e70019.

[17] Perdew, J. P.; Ernzerhof, M.; Burke, K. Rationale for Mixing Exact Exchange with Density Functional Approximations. *J. Chem. Phys.* **1996**, *105*, 9982–9985.

[18] Adamo, C.; Barone, V. Toward Reliable Density Functional Methods without Adjustable Parameters: The PBE0 Model. *J. Chem. Phys.* **1999**, *110*, 6158–6170.

[19] Grimme, S.; Antony, J.; Ehrlich, S.; Krieg, H. A Consistent and Accurate *Ab Initio* Parametrization of Density Functional Dispersion Correction (DFT-D) for the 94 Elements H–Pu. *J. Chem. Phys.* **2010**, *132*, 154104.

[20] Grimme, S.; Ehrlich, S.; Goerigk, L. Effect of the Damping Function in Dispersion-Corrected Density Functional Theory. *J. Comput. Chem.* **2011**, *32*, 1456–1465.

[21] Weigend, F.; Ahlrichs, R. Balanced Basis Sets of Split Valence, Triple Zeta Valence and Quadruple Zeta Valence Quality for H to Rn: Design and Assessment of Accuracy. *Phys. Chem. Chem. Phys.* **2005**, *7*, 3297–3305.

[22] Barone, V.; Cossi, M. Quantum Calculation of Molecular Energies and Energy Gradients in Solution by a Conductor Solvent Model. *J. Phys. Chem. A* **1998**, *102*, 1995–2001.

[23] Garcia-Ratés, M.; Neese, F. Effect of the Solute Cavity on the Solvation Energy and Its Derivatives within the Framework of the Gaussian Charge Scheme. *J. Comput. Chem.* **2020**, *41*, 922–939.

[24] Küchle, W.; Dolg, M.; Stoll, H.; Preuss, H. *Ab Initio* Pseudopotentials for Hg through Rn. *Mol. Phys.* **1991**, *74*, 1245–1263.

[25] Hirshfeld, F. L. Bonded-Atom Fragments for Describing Molecular Charge Densities. *Theor. Chim. Acta* **1977**, *44*, 129–138.

[26] Glendening, E. D.; Badenhoop, J. K.; Reed, A. E.; Carpenter, J. E.; Bohmann, J. A.; Morales, C. M.; Karafiloglou, P.; Landis, C. R.; Weinhold, F., NBO 7.0; Theoretical Chemistry Institute, University of Wisconsin: Madison, WI, 2018.

[27] Kratzert, D.; Holstein, J. J.; Krossing, I. DSR: Enhanced Modelling and Refinement of Disordered Structures with SHELXL. *J. Appl. Crystallogr.* **2015**, *48*, 933–938.

[28] Kratzert, D.; Krossing, I. Recent Improvements in DSR. *J. Appl. Crystallogr.* **2018**, *51*, 928–934.

[29] Dolomanov, O. V.; Bourhis, L. J.; Gildea, R. J.; Howard, J. A. K.; Puschmann, H. OLEX2: A Complete Structure Solution, Refinement and Analysis Program. *J. Appl. Crystallogr.* **2009**, *42*, 339–341.

10 NMR spectra

10.1 [Bi-2]•F

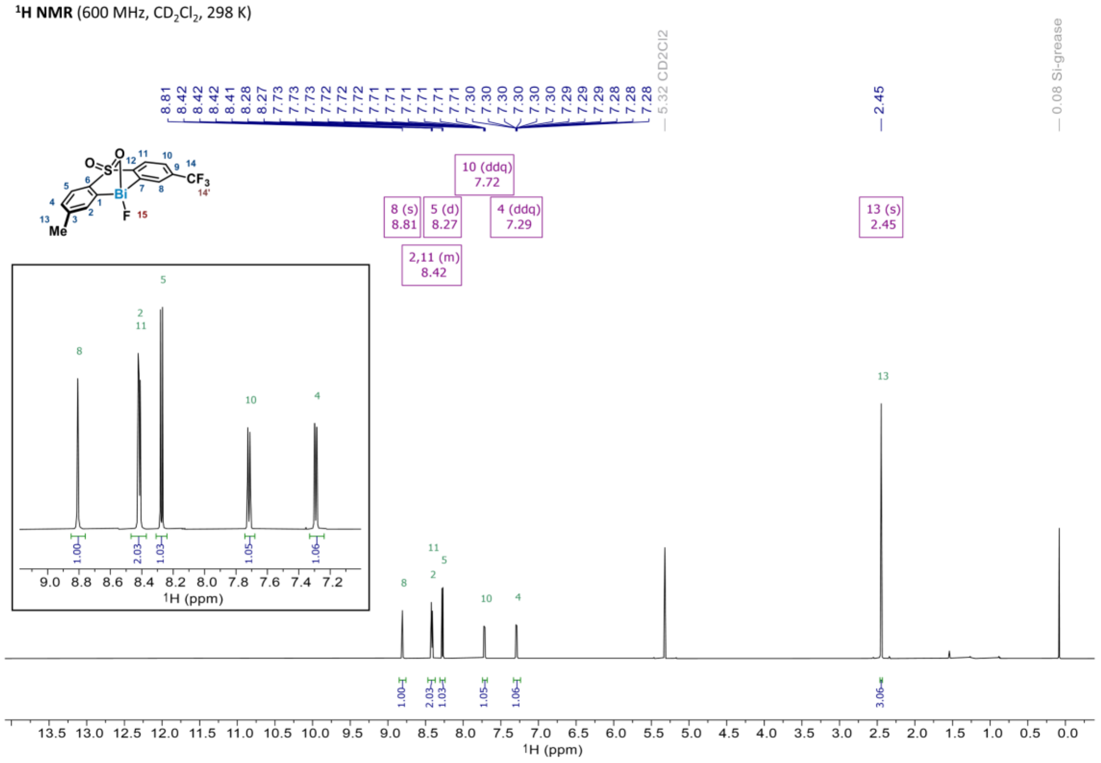

Figure S51. ¹H NMR spectrum of **[Bi-2]•F**

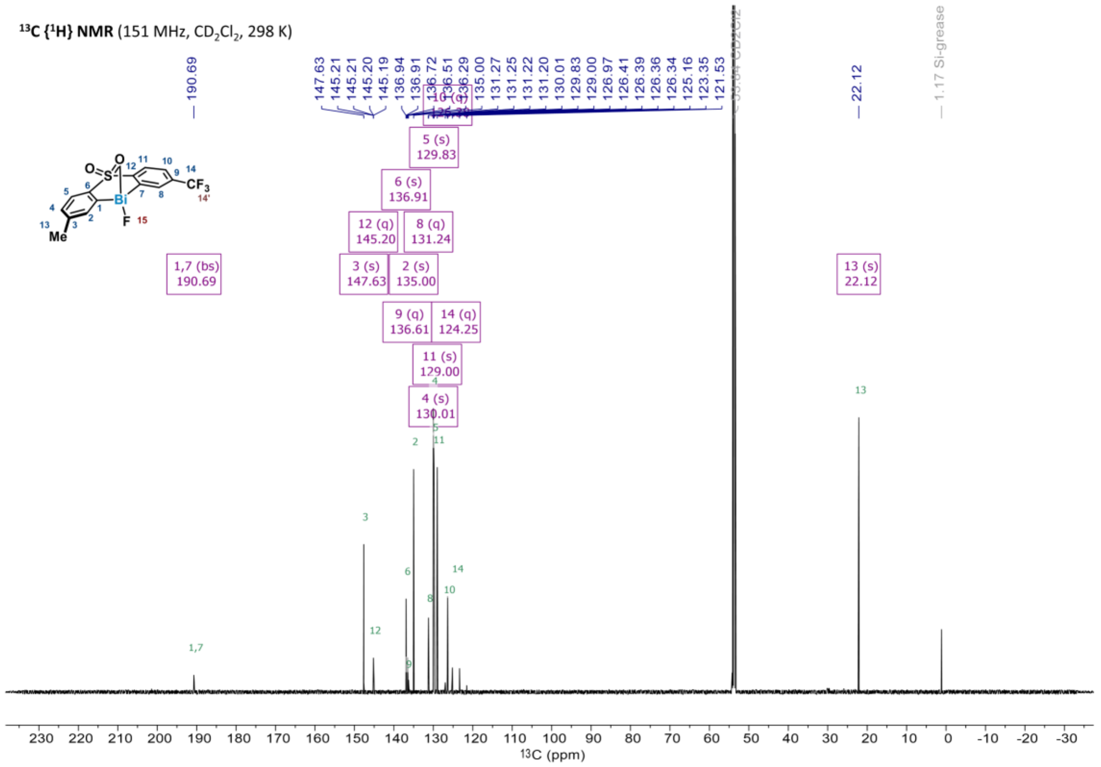

Figure S52. $^{13}C\{^{1}H\}$ NMR spectrum of **[Bi-2]•F**

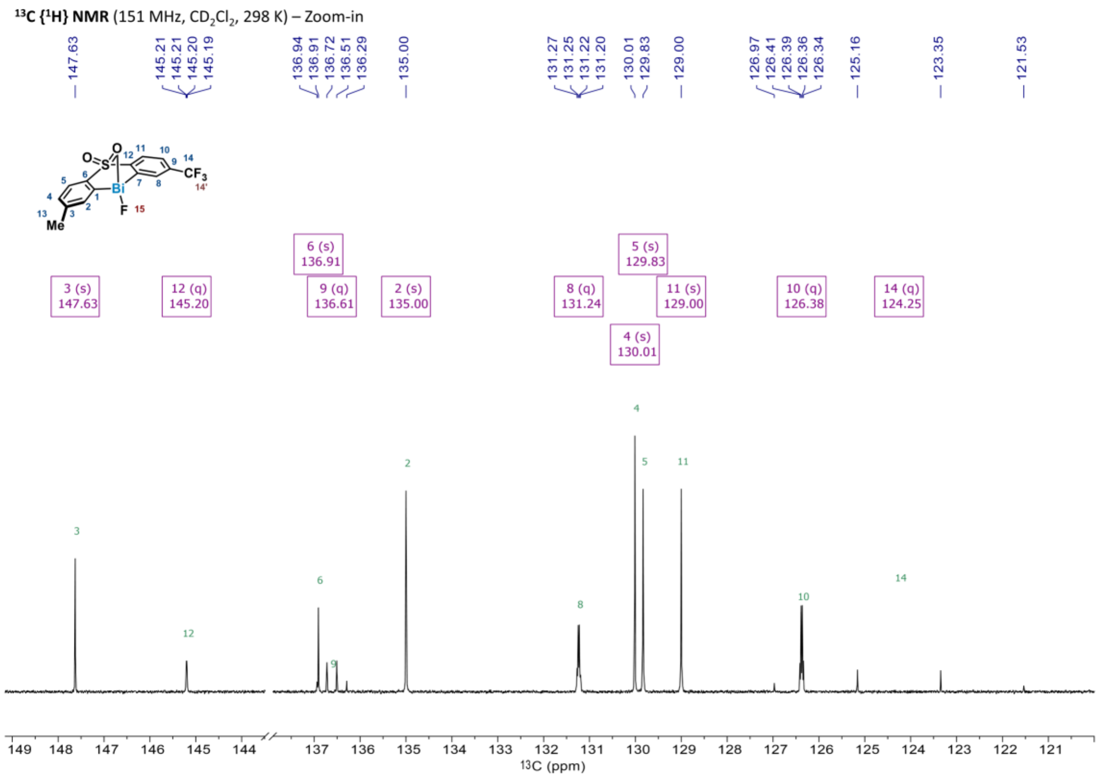

Figure S53. Zoom-in of the $^{13}C\{^{1}H\}$ NMR spectrum of **[Bi-2]•F**

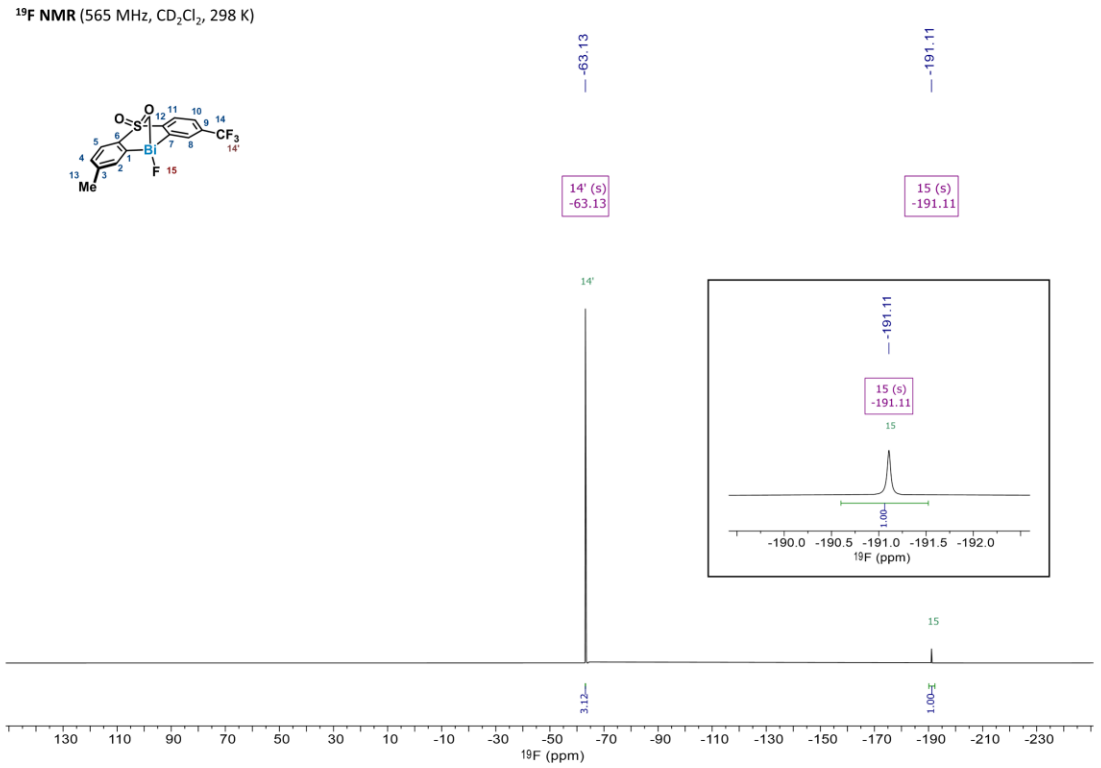

Figure S54. ^{19}F NMR spectrum of **[Bi-2]•F**

10.2 [Bi-3]•F

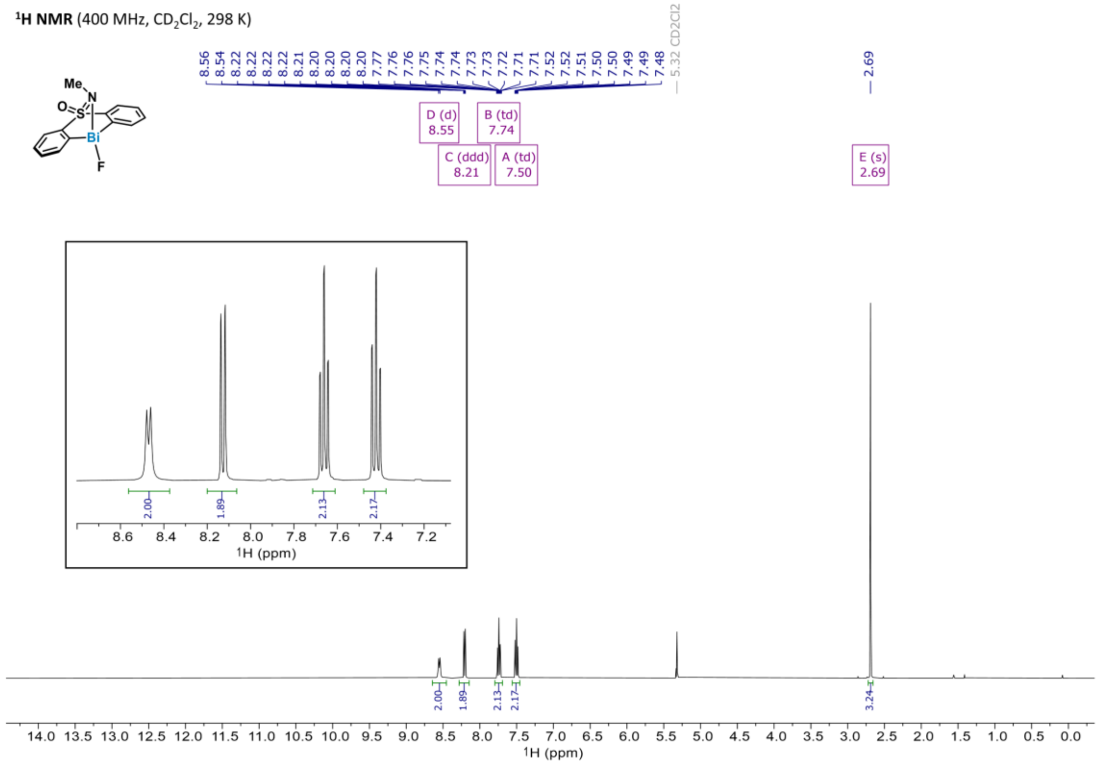

Figure S55. ^{1}H NMR spectrum of **[Bi-3]•F**

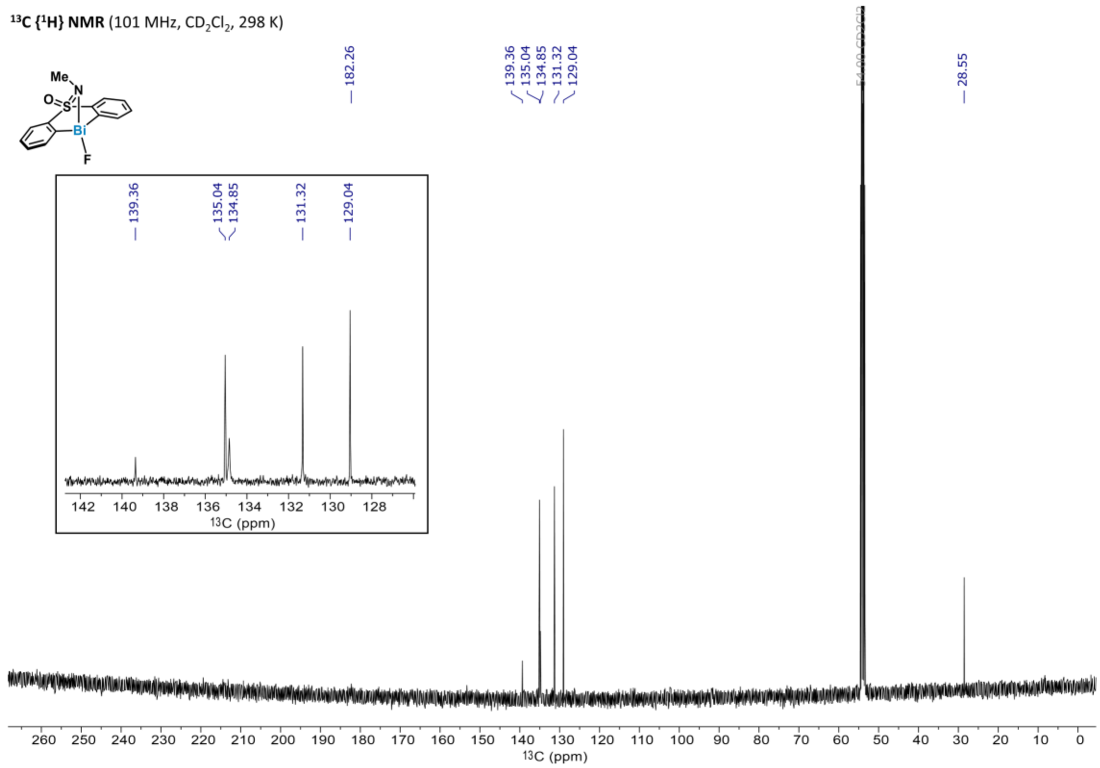

Figure S56. ^{13}C{^{1}H} NMR spectrum of **[Bi-3]•F**

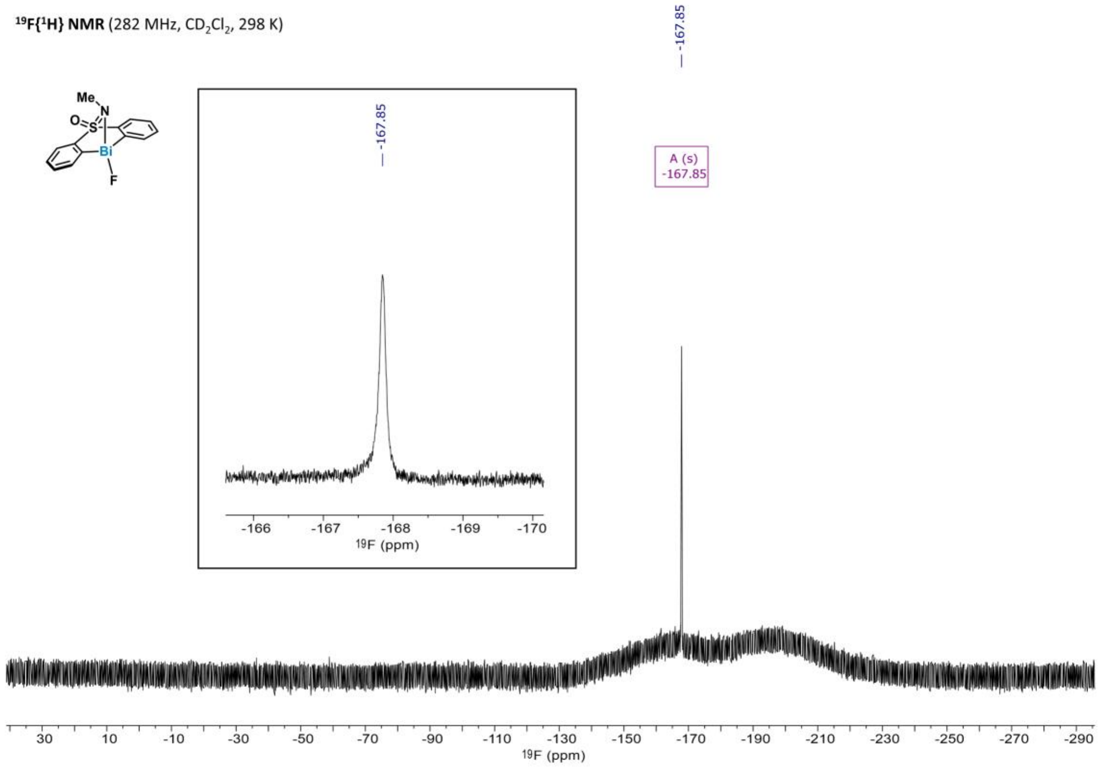

Figure S57. $^{19}F\{^{1}H\}$ NMR spectrum of **[Bi-3]•F**

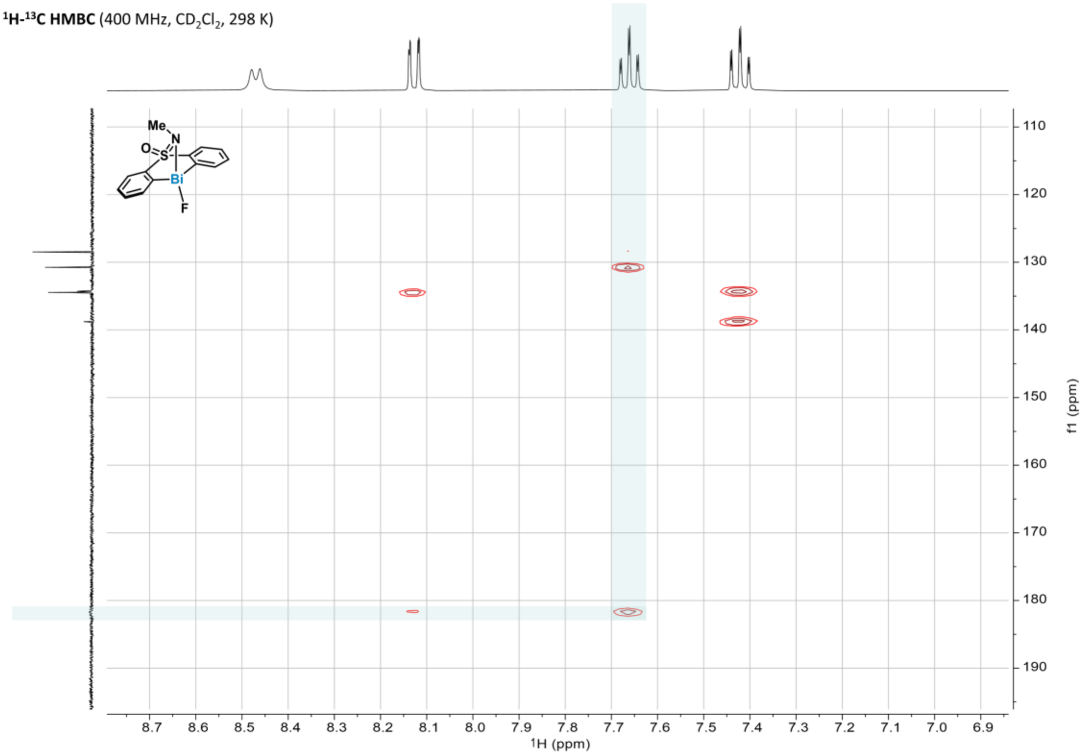

Figure S58. ^{1}H-^{13}C HMBC spectrum of **[Bi-3]•F**.

10.3 [Bi-4]•F

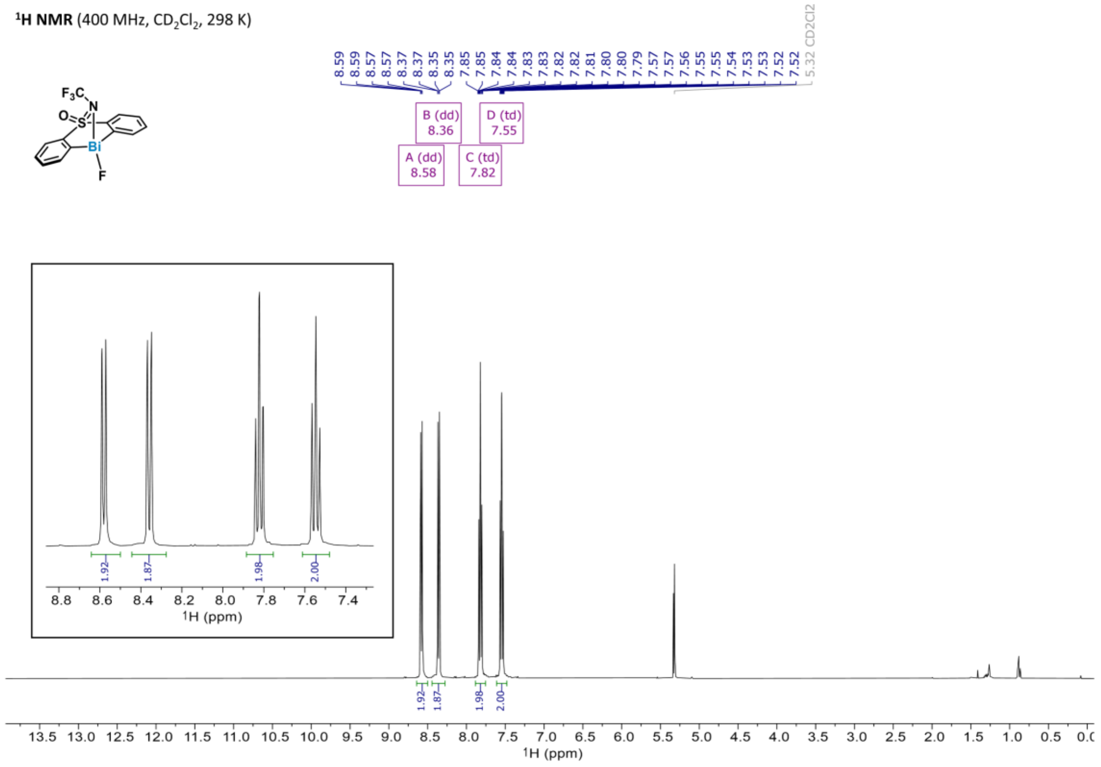

Figure S59. ^{1}H NMR spectrum of **[Bi-4]•F**.

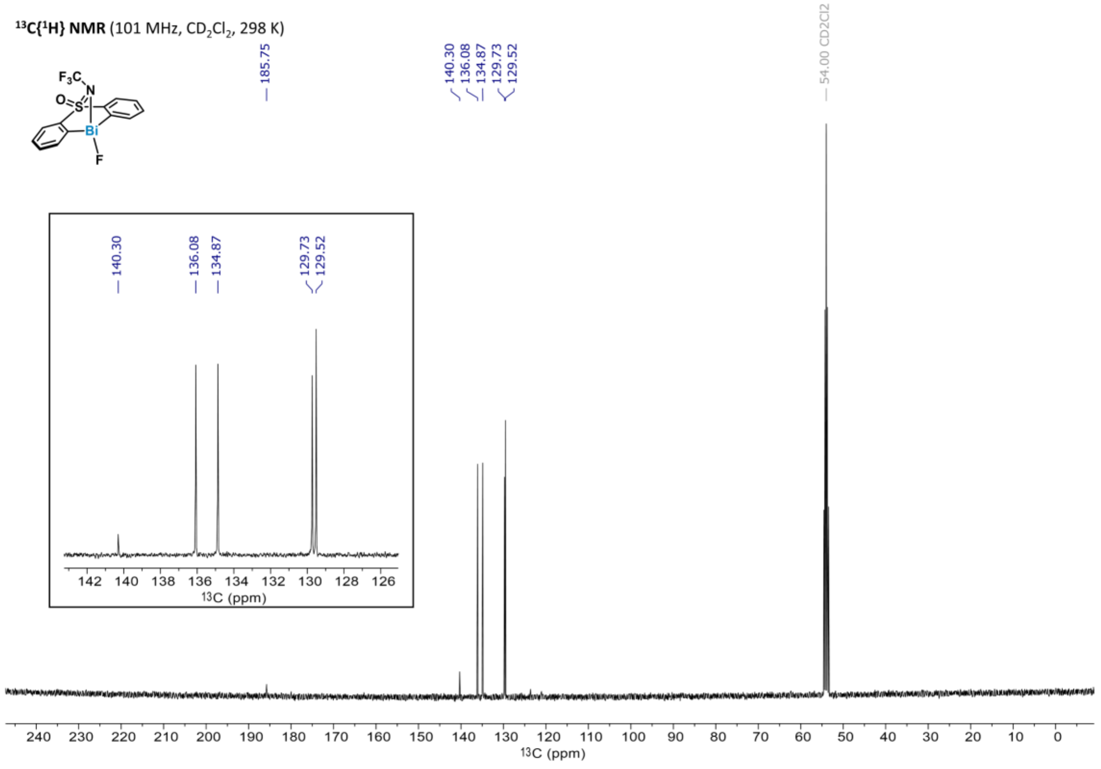

Figure S60. ^{13}C{^{1}H} NMR spectrum of **[Bi-3]•F**

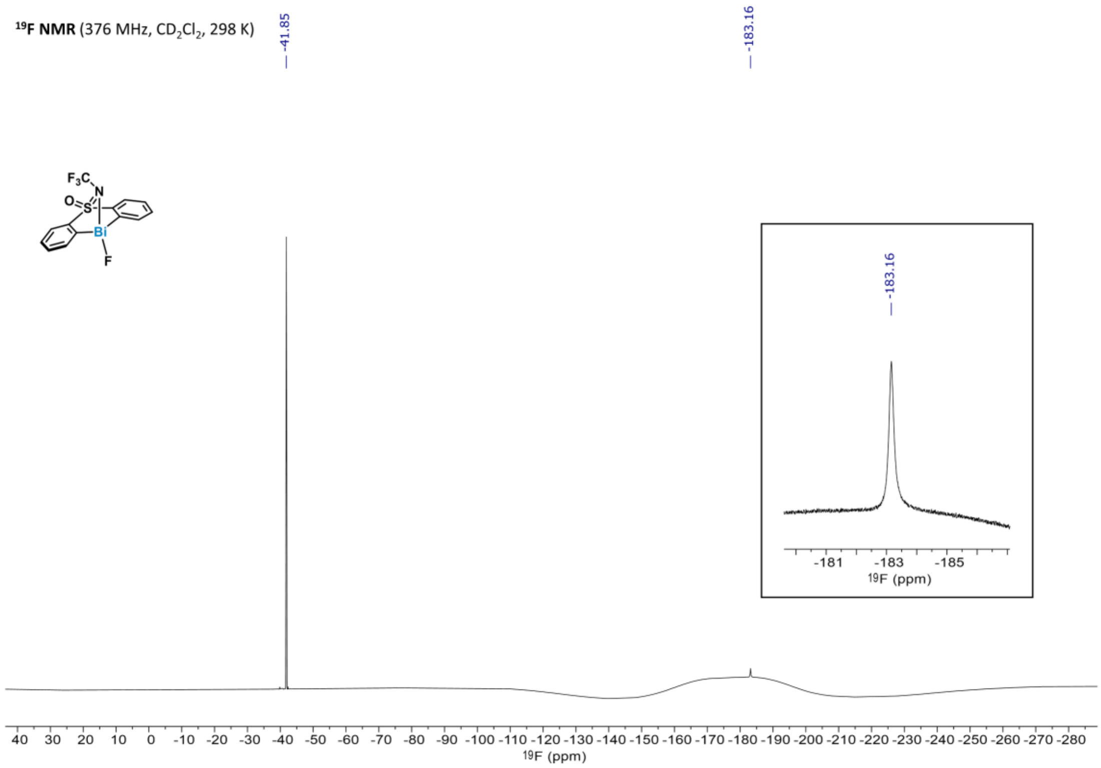

Figure S61. ^{19}F NMR spectrum of **[Bi-4]•F**

10.4 [Bi-2]•OTf

In CD_3CN

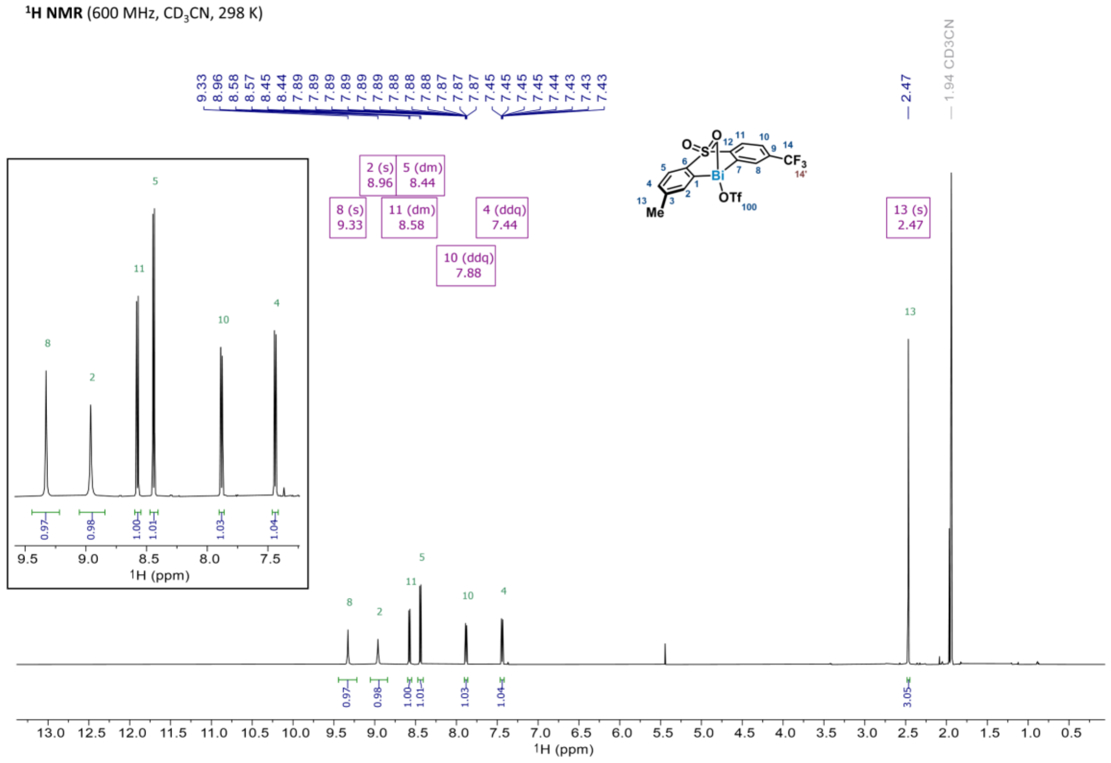

Figure S62. ¹H NMR spectrum of **[Bi-2]•OTf**.

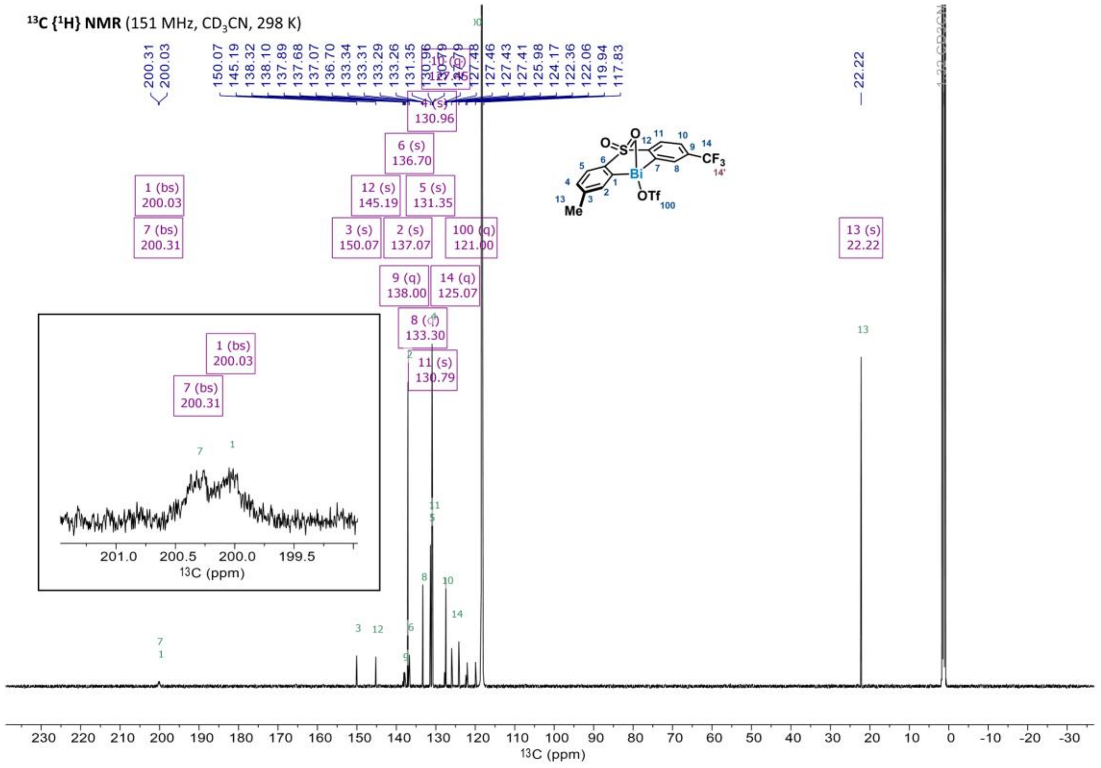

Figure S63. $^{13}C\{^1H\}$ NMR spectrum of **[Bi-2]•OTf**.

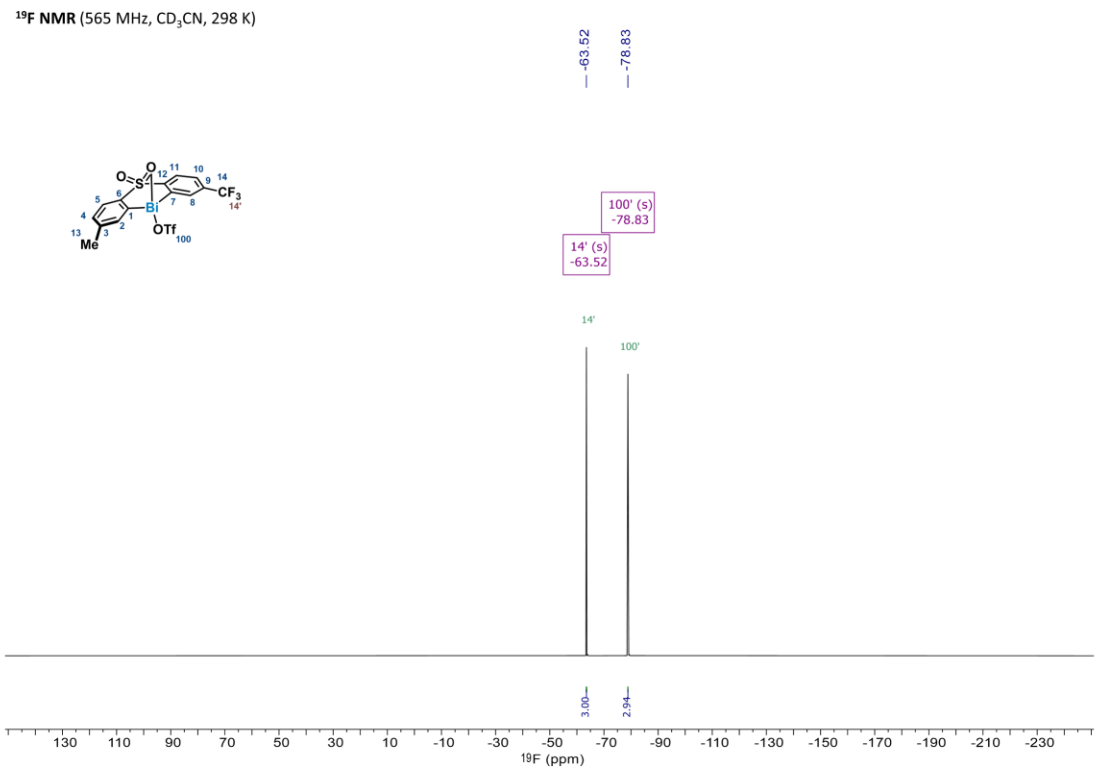

Figure S64. ^{19}F NMR spectrum of **[Bi-1]•OTf**.

In CD_2Cl_2

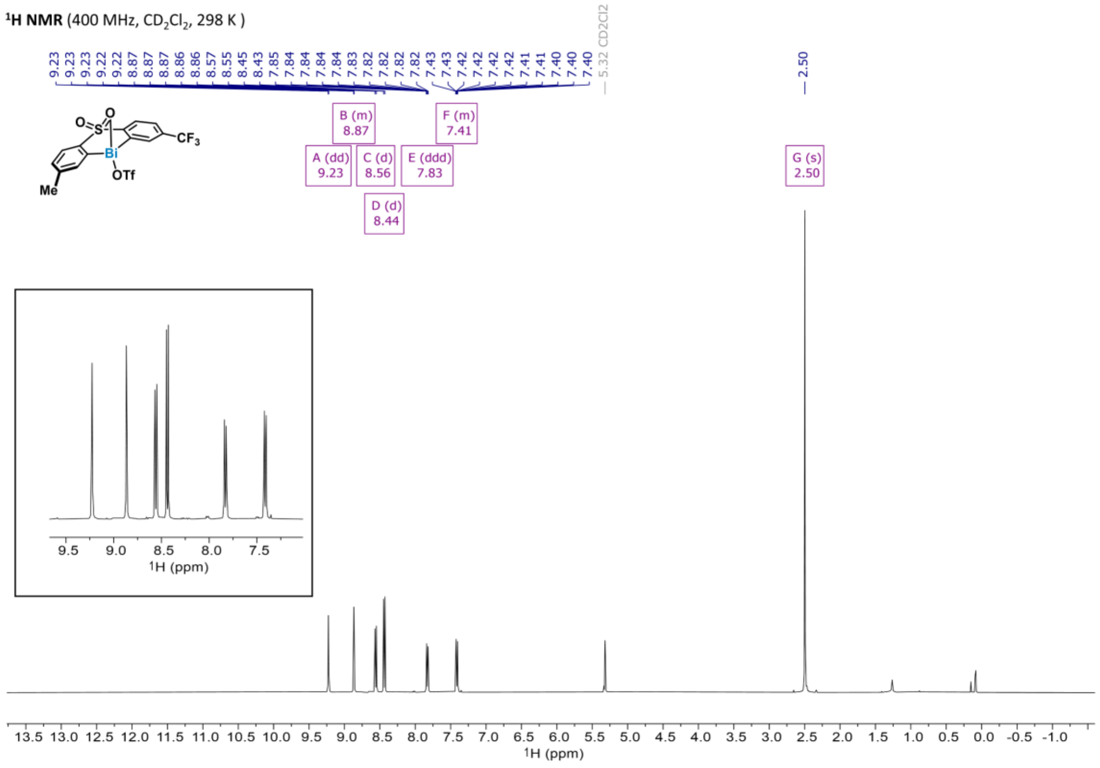

Figure S65. ^{1}H NMR spectrum of **[Bi-2]•OTf**.

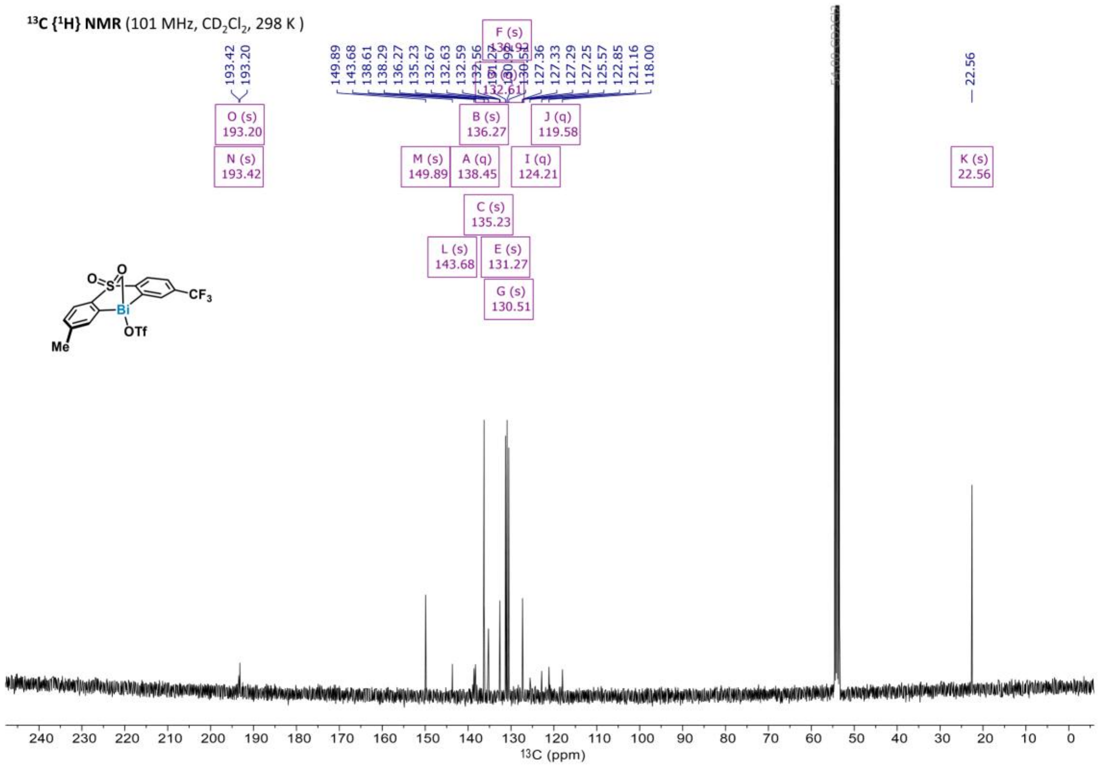

Figure S66. $^{13}C\{^{1}H\}$ NMR spectrum of **[Bi-2]•OTf**.

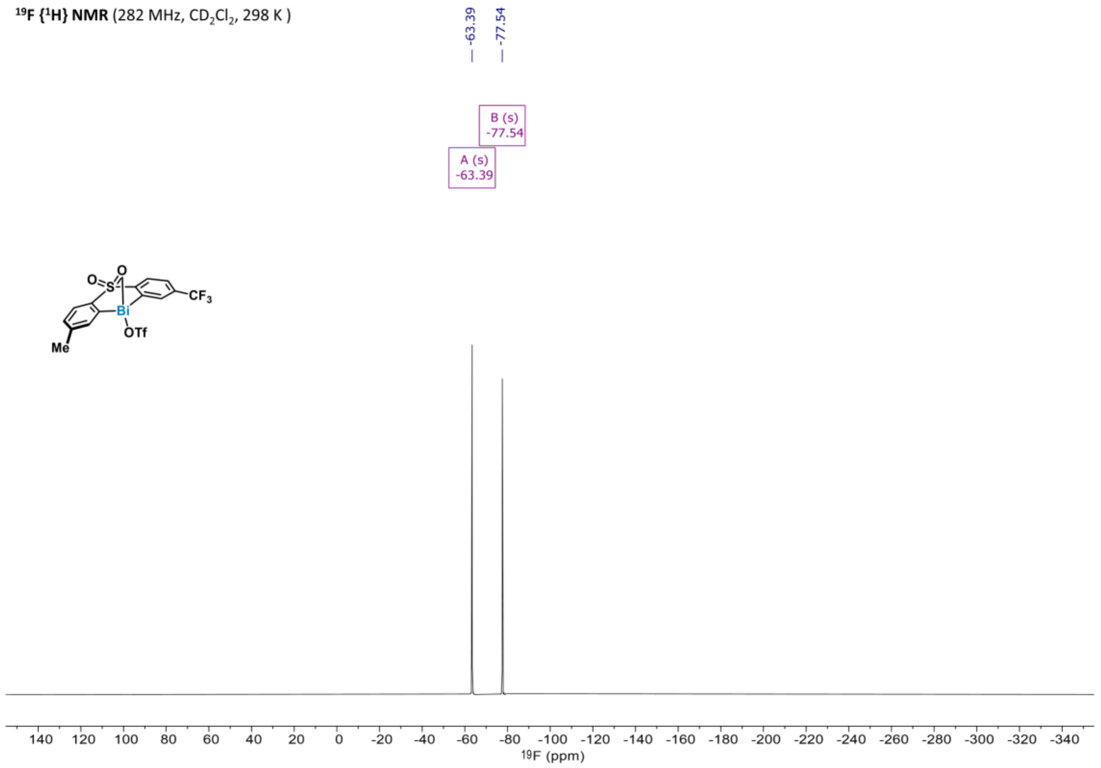

Figure S67. $^{19}F\{^{1}H\}$ NMR spectrum of **[Bi-1]•OTf**.

10.5 [Bi-2]•I

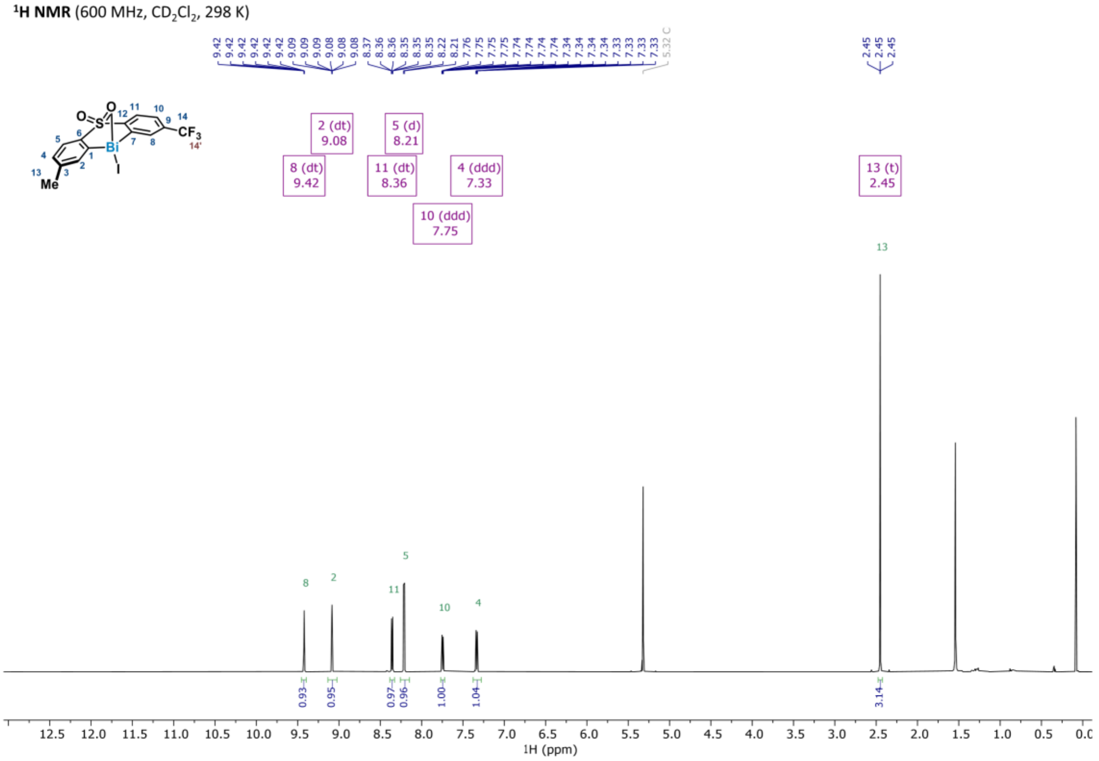

Figure S68. ^{1}H NMR spectrum of **[Bi-2]•I**.

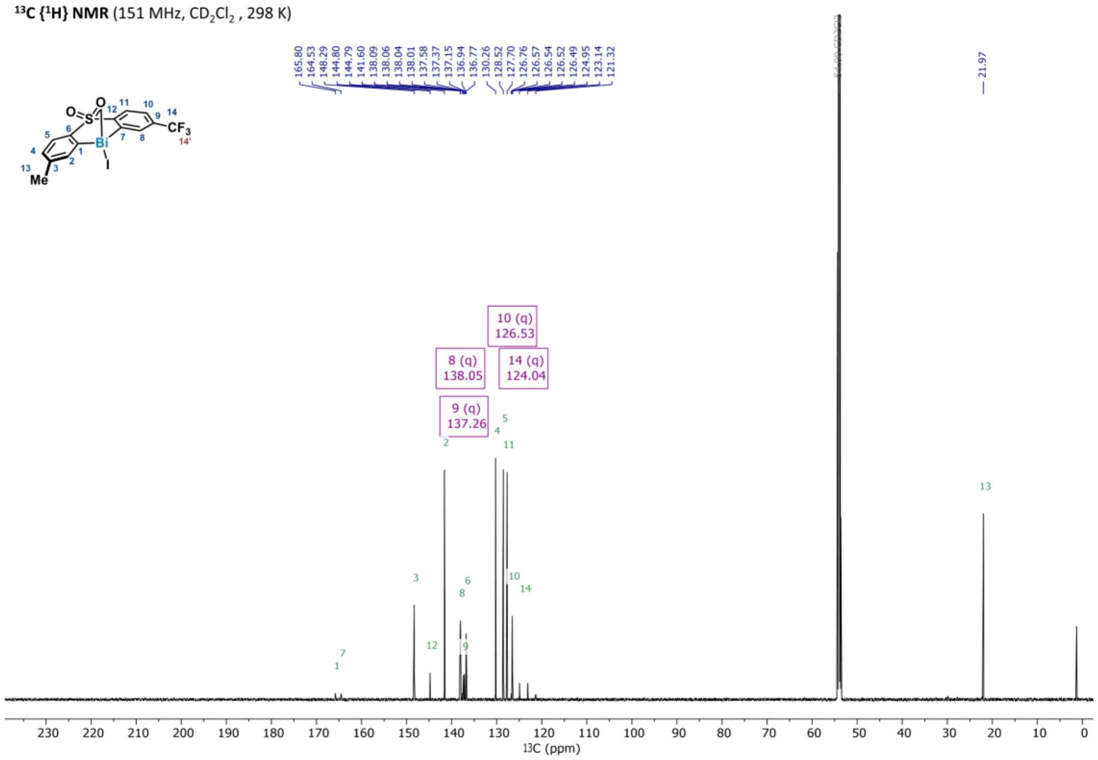

Figure S69. ^{13}C{^{1}H} NMR spectrum of **[Bi-2]•I**.

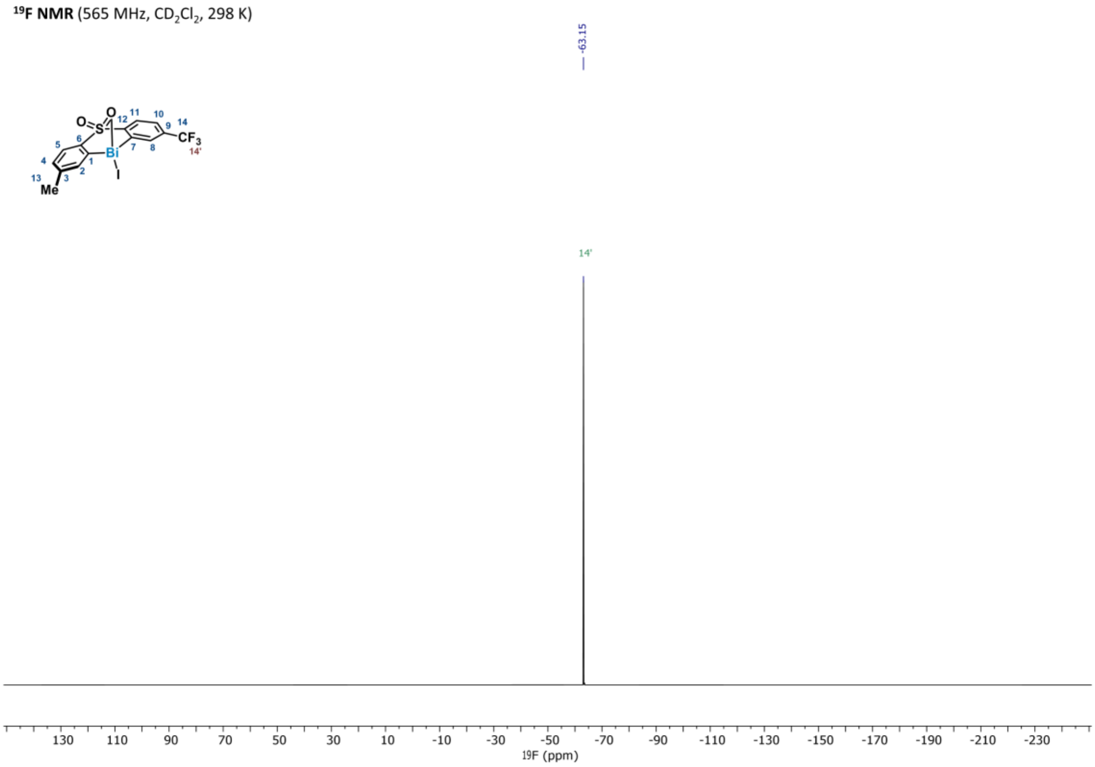

Figure S70. ^{19}F NMR spectrum of **[Bi-1]•I**.

10.6 [Bi-2]•B(Ar^F)$_3$F

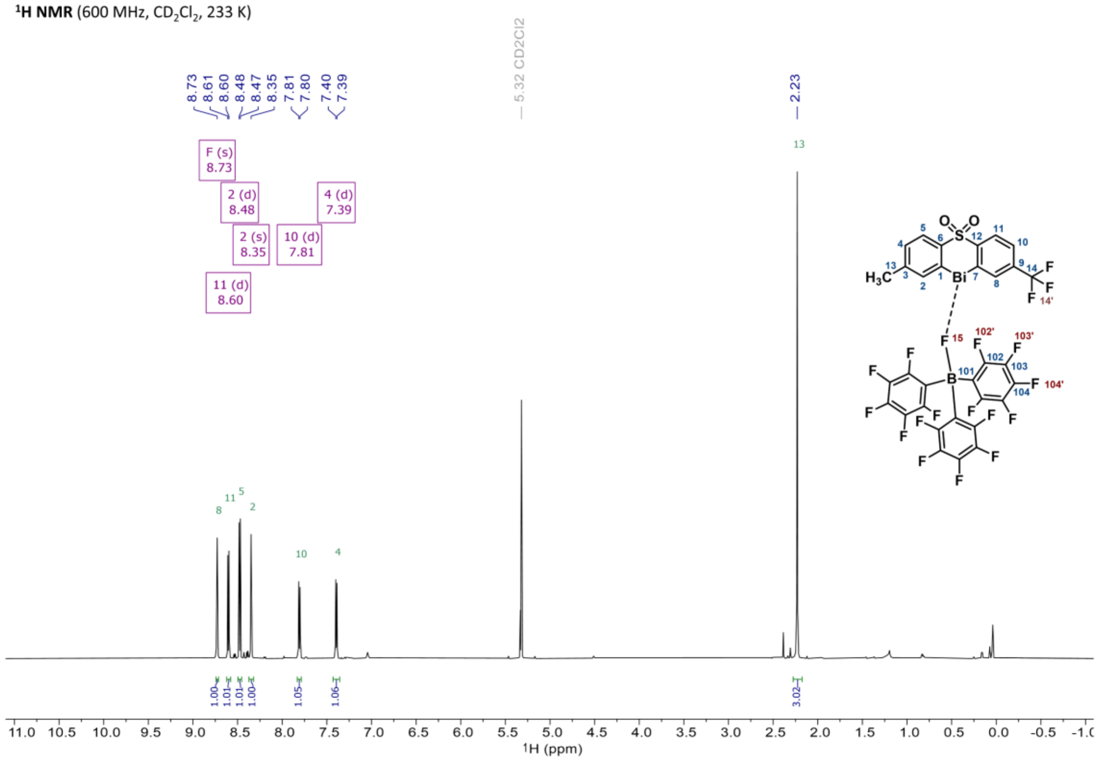

Figure S71. ^{1}H NMR spectrum of **[Bi-2]•B(Ar^F)$_3$F**.

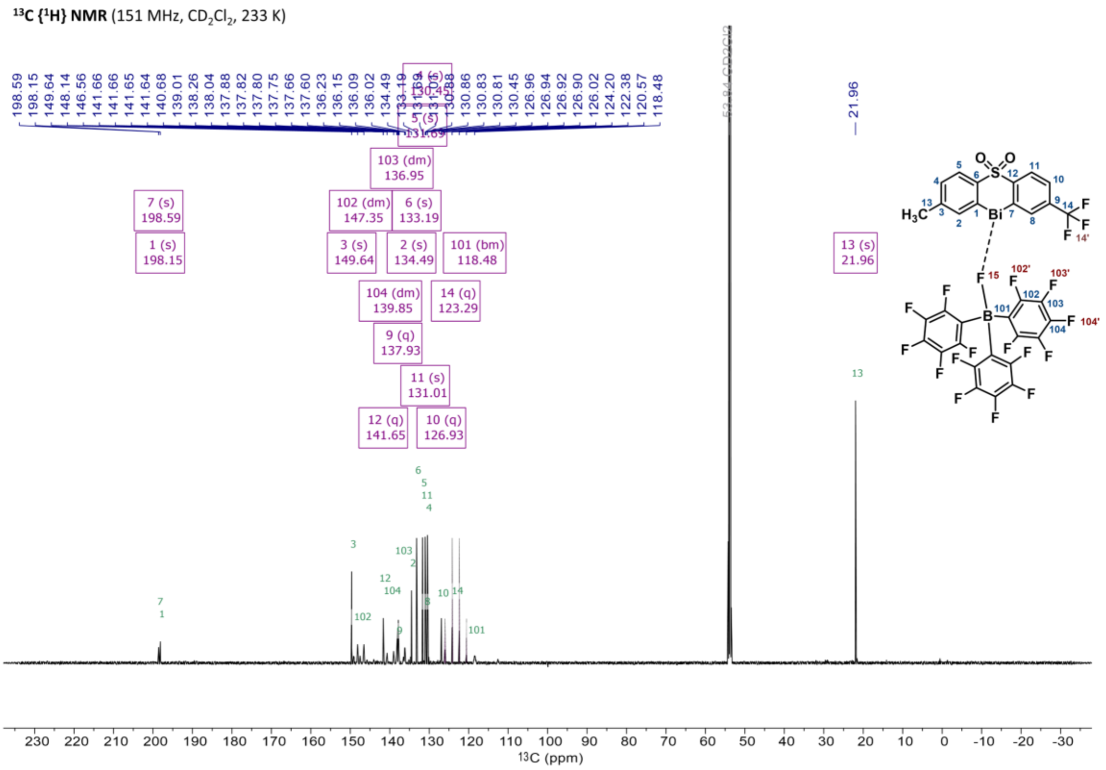

Figure S72. ^{13}C{^{1}H} NMR spectrum of **[Bi-2]•B(Ar^F)$_3$F**.

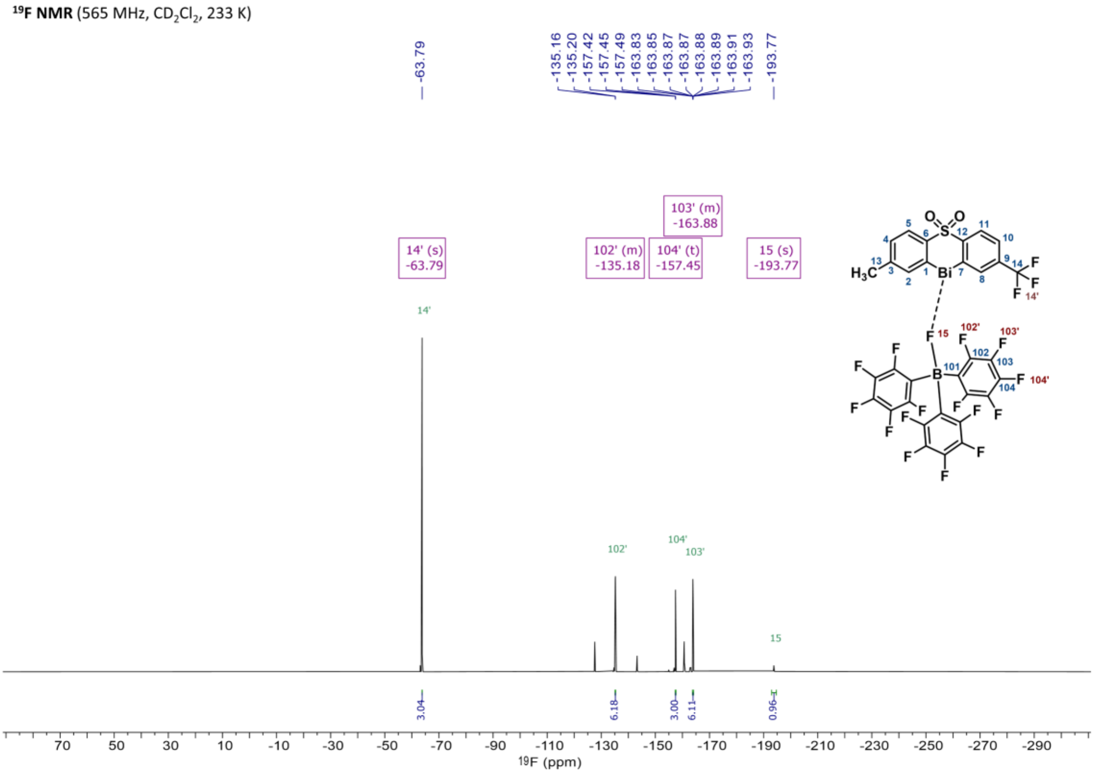

Figure S73. ^{19}F NMR spectrum of **[Bi-2]•B(Ar^F)$_3$F**.

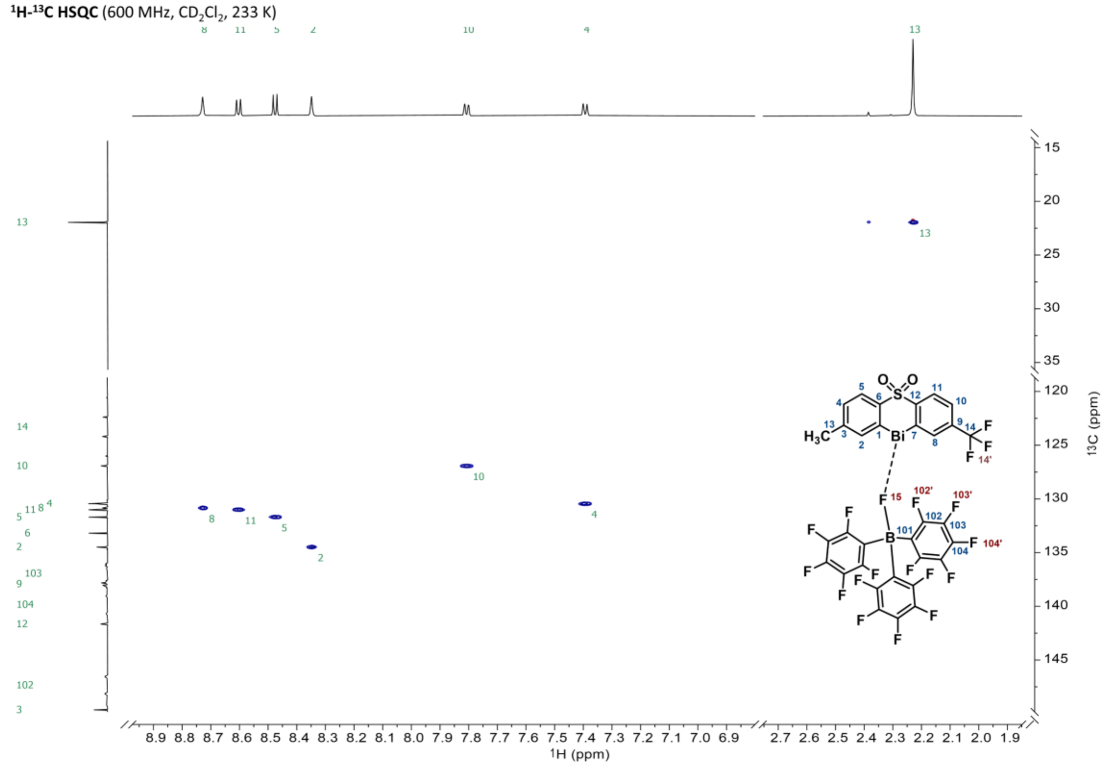

Figure S74. ^{1}H-^{13}C HSQC spectrum of **[Bi-2]•B(Ar^F)$_3$F**.

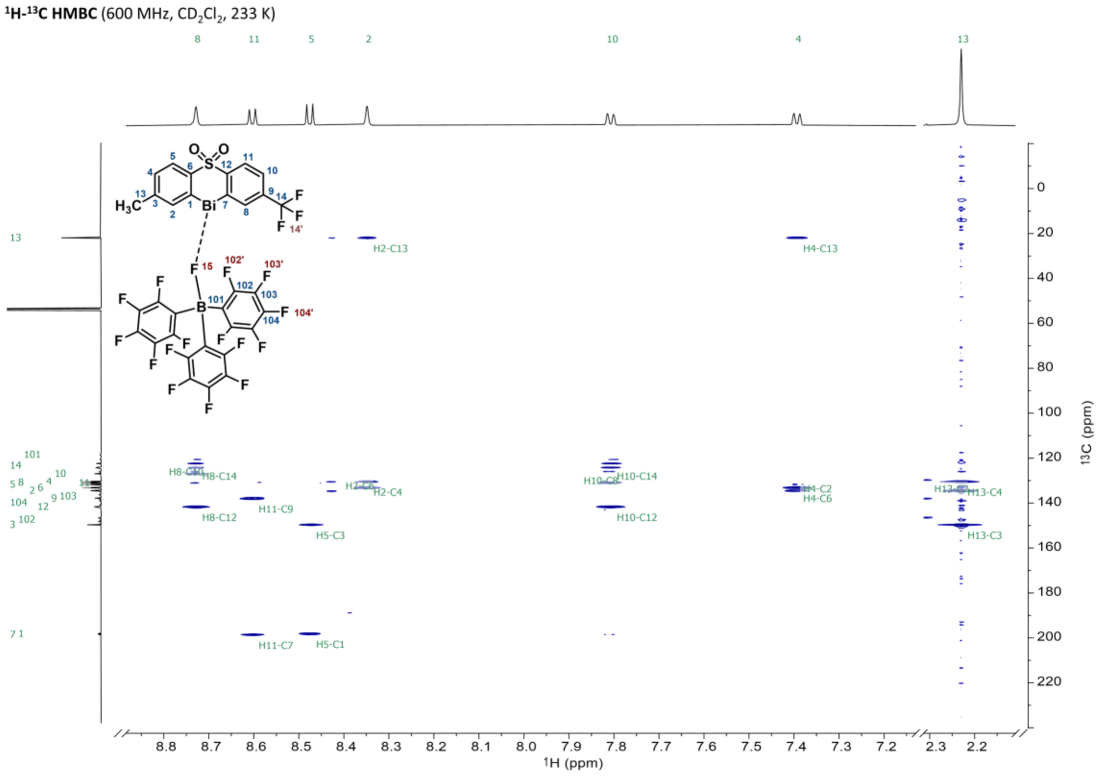

Figure S75. ^{1}H-^{13}C HMBC spectrum of **[Bi-2]•B(Ar^{F})$_3$F**.

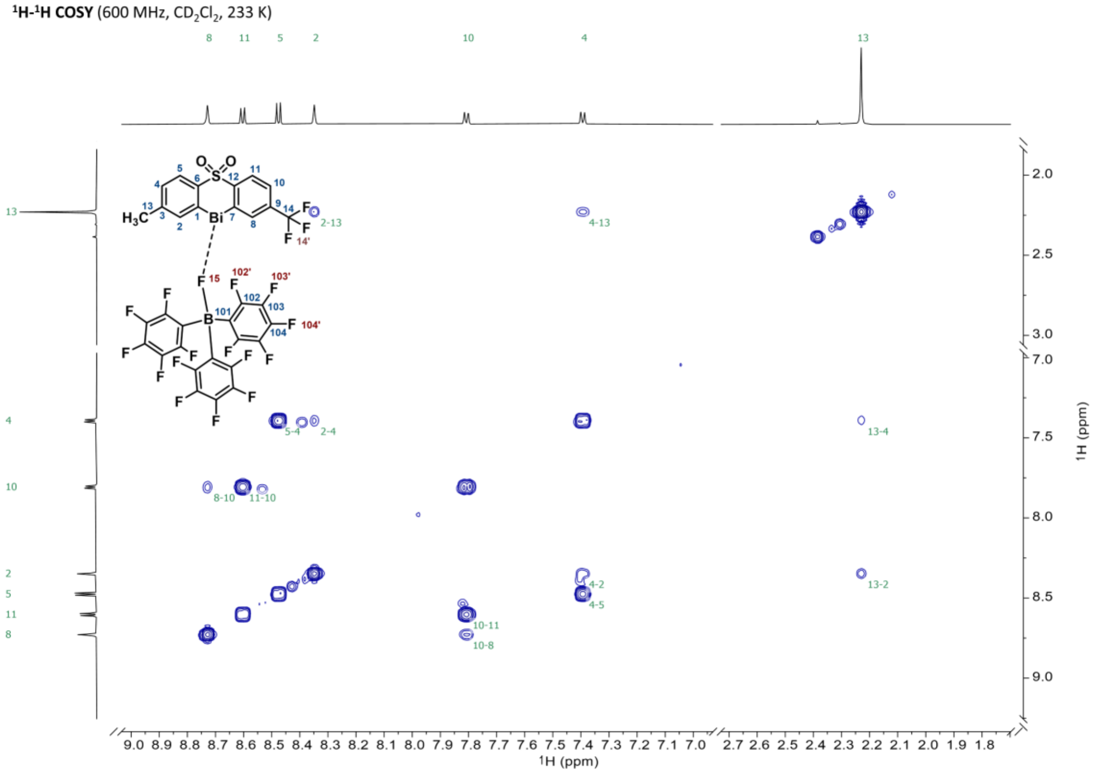

Figure S76. ^{1}H-^{1}H COSY spectrum of **[Bi-2]•B(Ar^{F})$_3$F**.

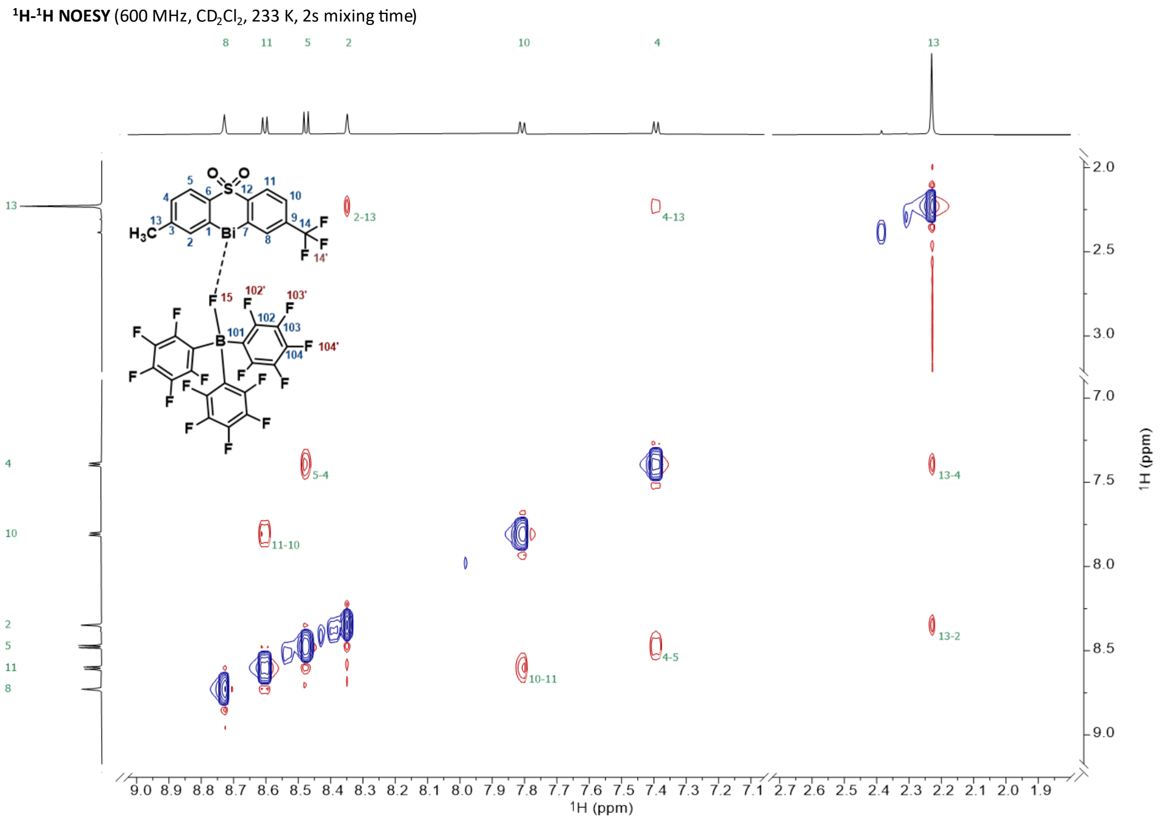

Figure S77. ^{1}H-^{1}H NOESY spectrum of **[Bi-2]•B(Ar^{F})$_3$F**.

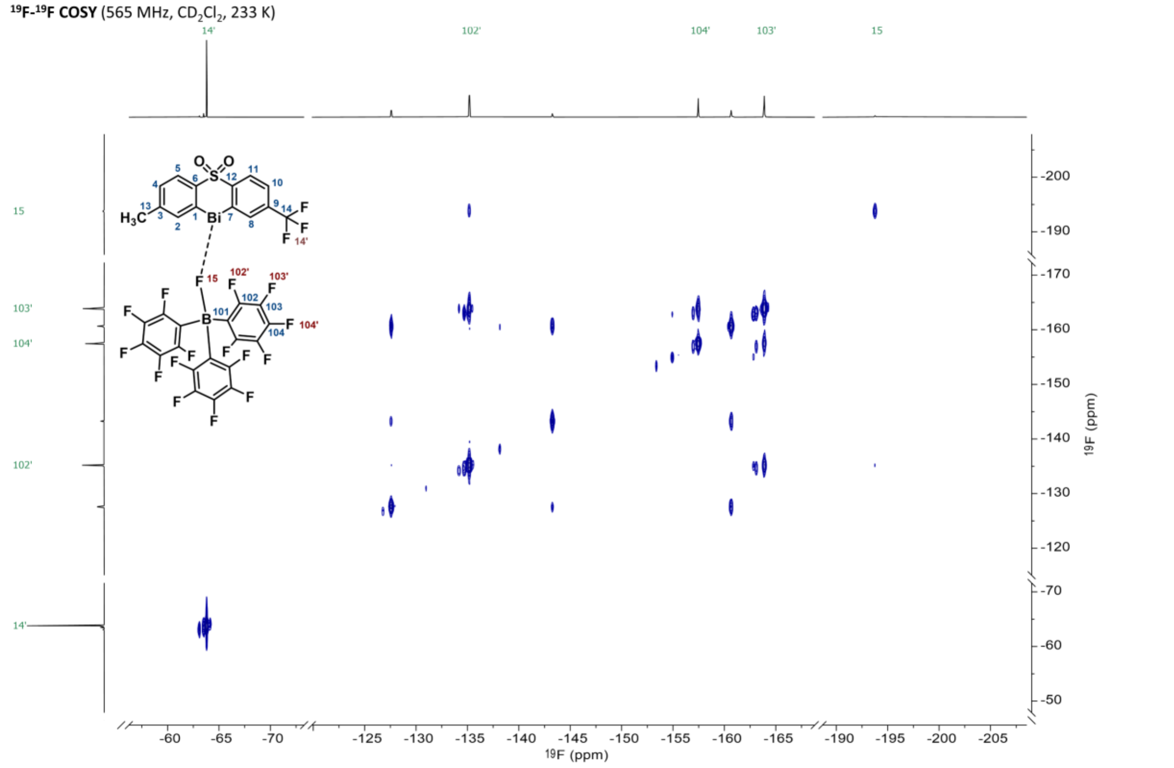

Figure S78. ^{19}F-^{19}F COSY spectrum of **[Bi-2]•B(Ar^{F})$_3$F**.

10.7 [Bi-3]•B(Ar^F)$_3$F

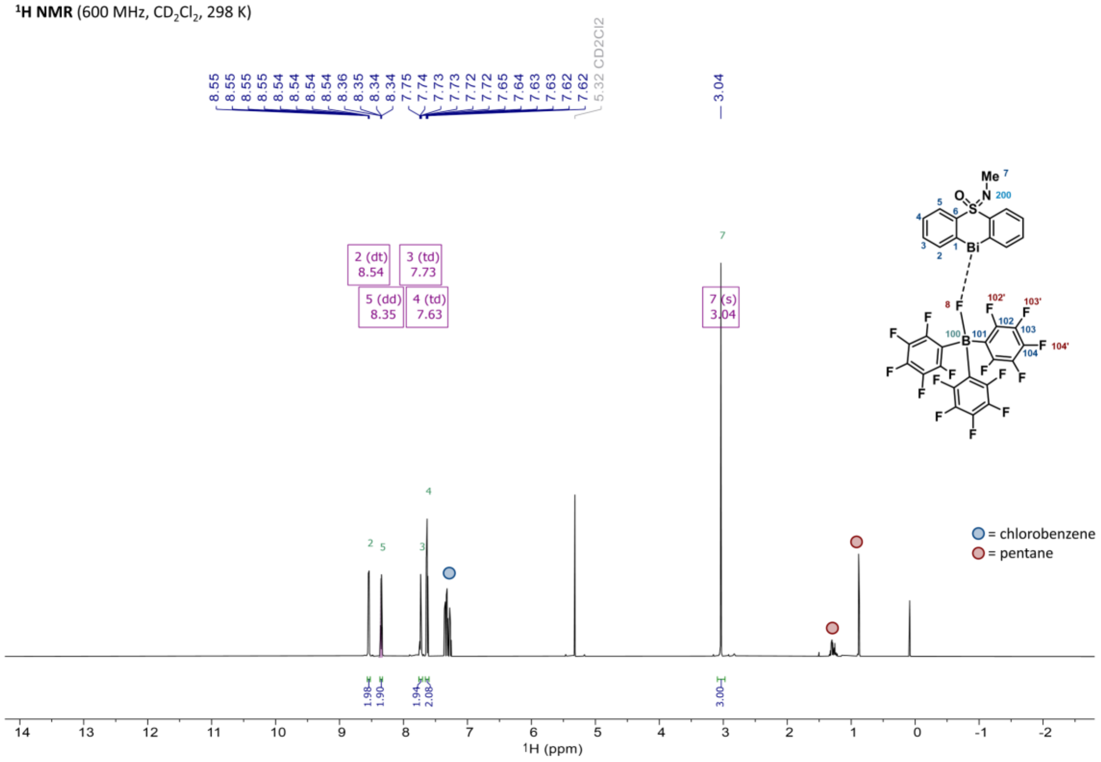

Figure S79. ^{1}H NMR spectrum of **[Bi-3]•B(Ar^F)$_3$F**.

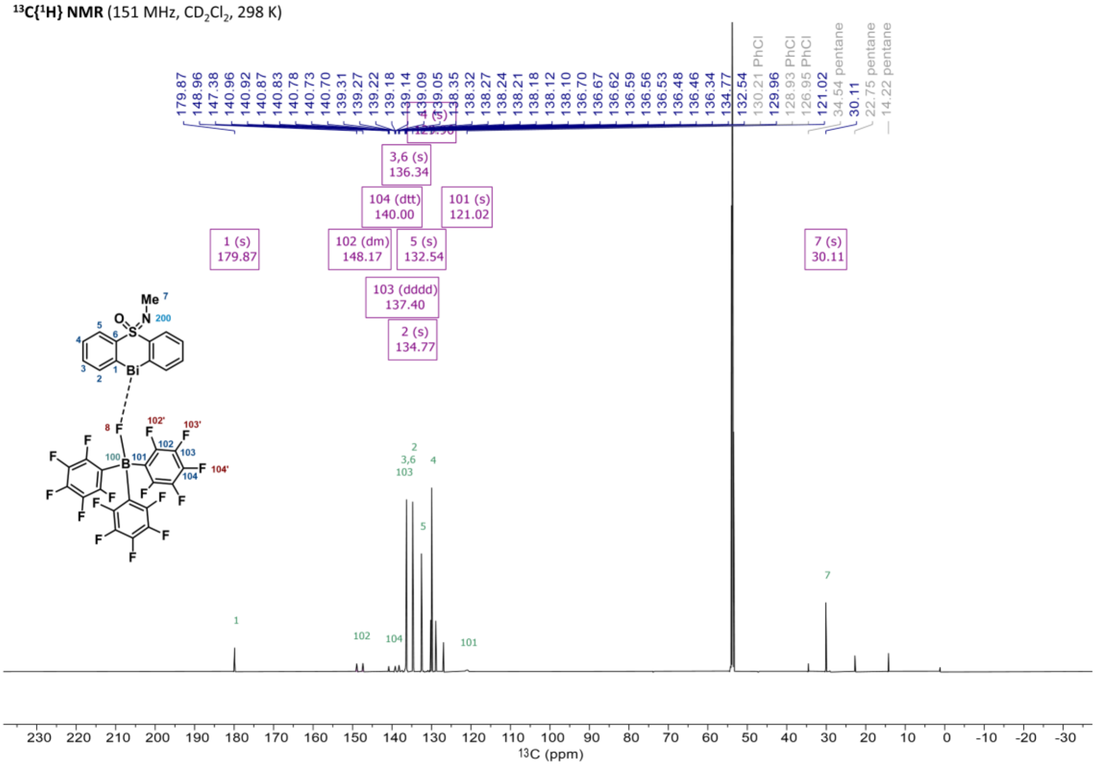

Figure S80. ^{13}C{^{1}H} NMR spectrum of **[Bi-3]•B(Ar^F)$_3$F**.

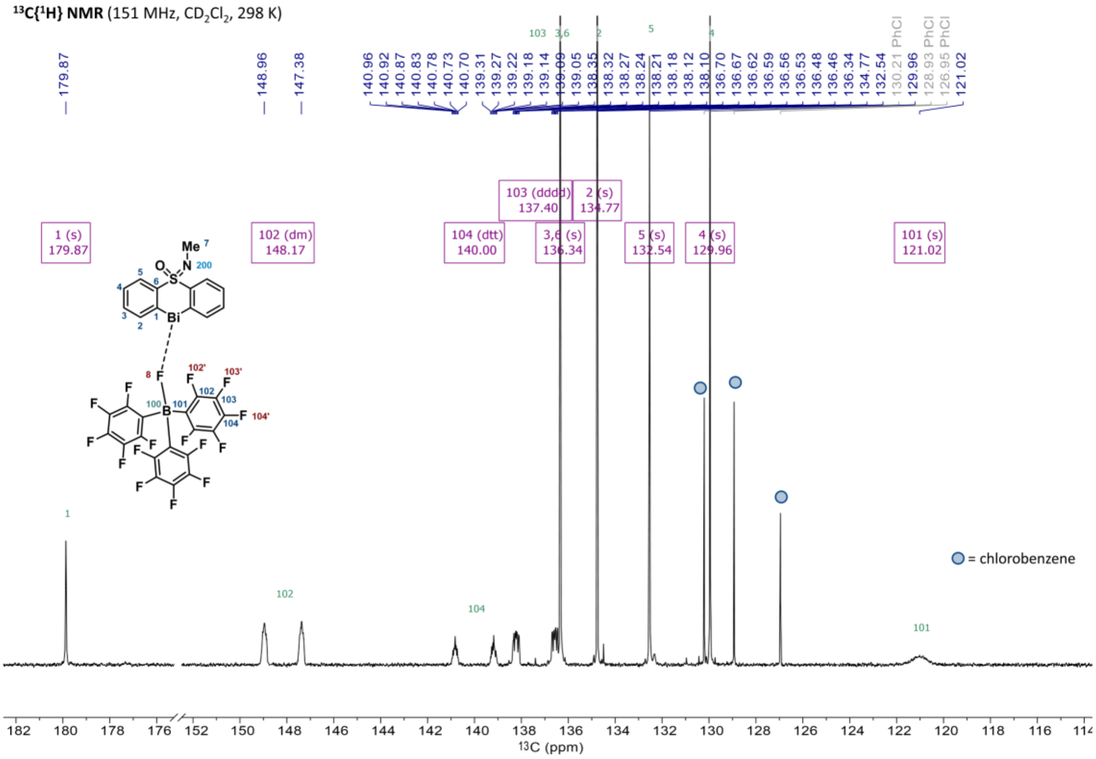

Figure S81. Zoom-in in the $^{13}C\{^{1}H\}$ NMR spectrum of **[Bi-3]•B(Ar^{F})$_3$F**.

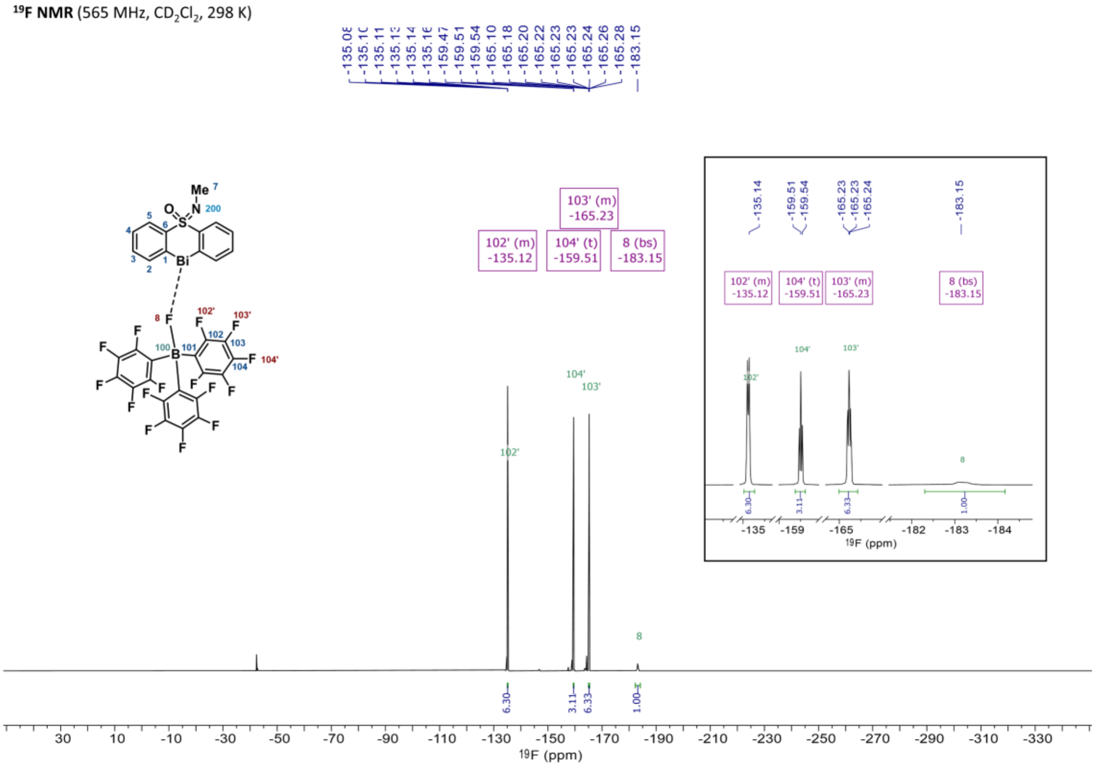

Figure S82. ^{19}F NMR spectrum of **[Bi-3]•B(Ar^{F})$_3$F**.

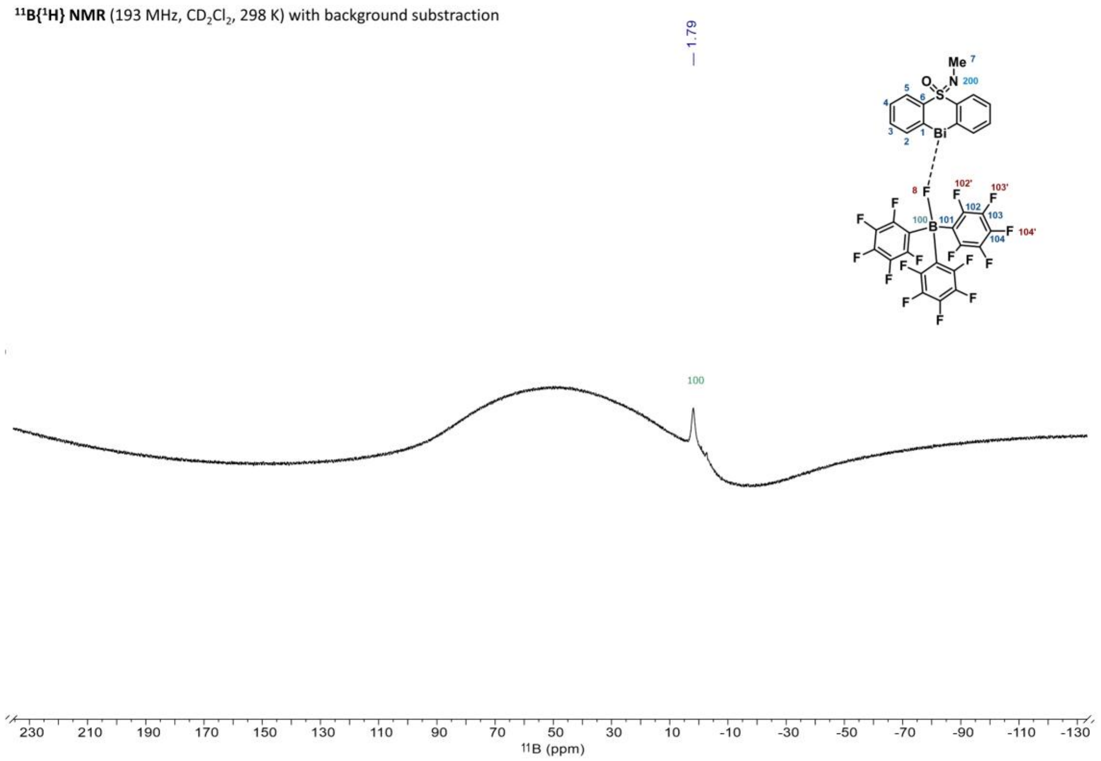

Figure S83. ^{11}B{^{1}H} NMR spectrum of **[Bi-3]•B(Ar^{F})$_3$F**.

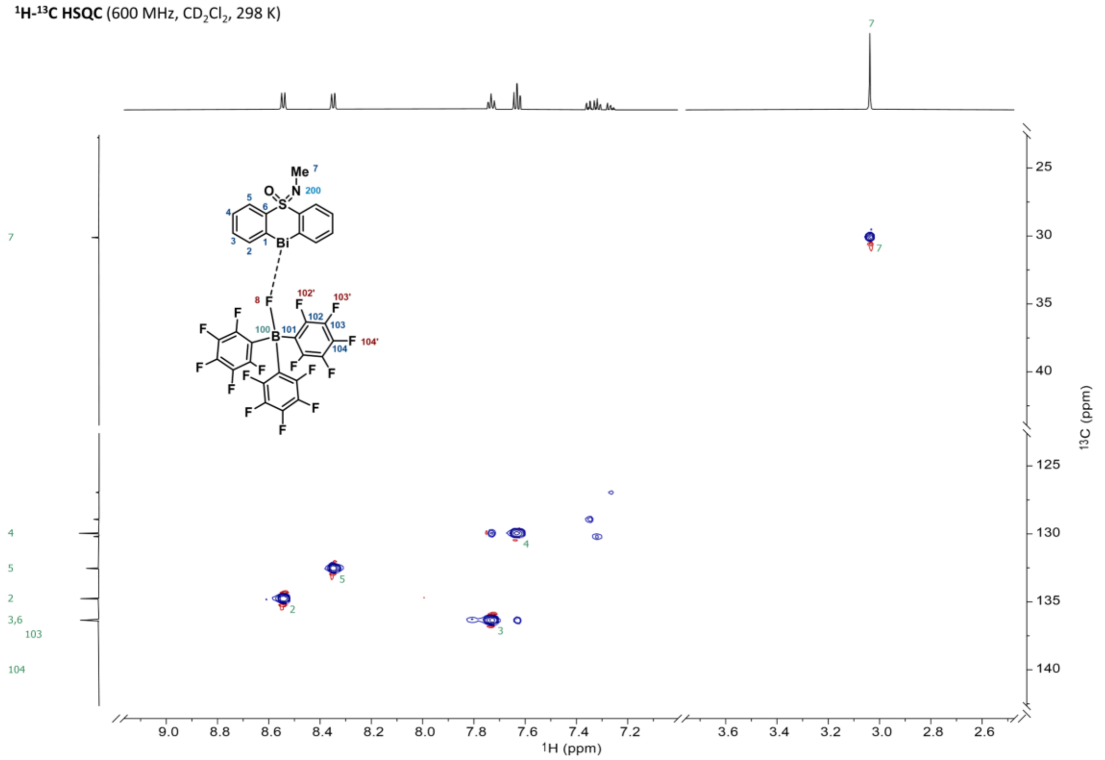

Figure S84. ^{1}H-^{13}C HSQC spectrum of **[Bi-3]•B(Ar^{F})$_3$F**.

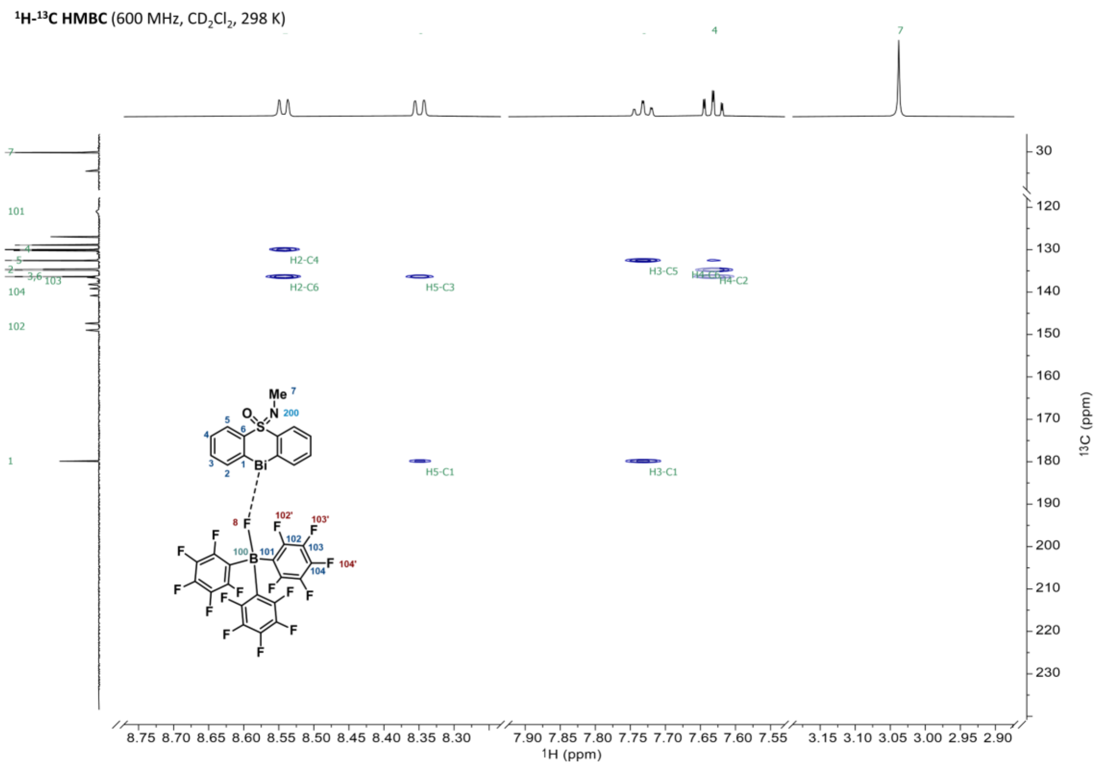

Figure S85. ^{1}H-^{13}C HMBC spectrum of **[Bi-3]•B(Ar^{F})$_3$F**.

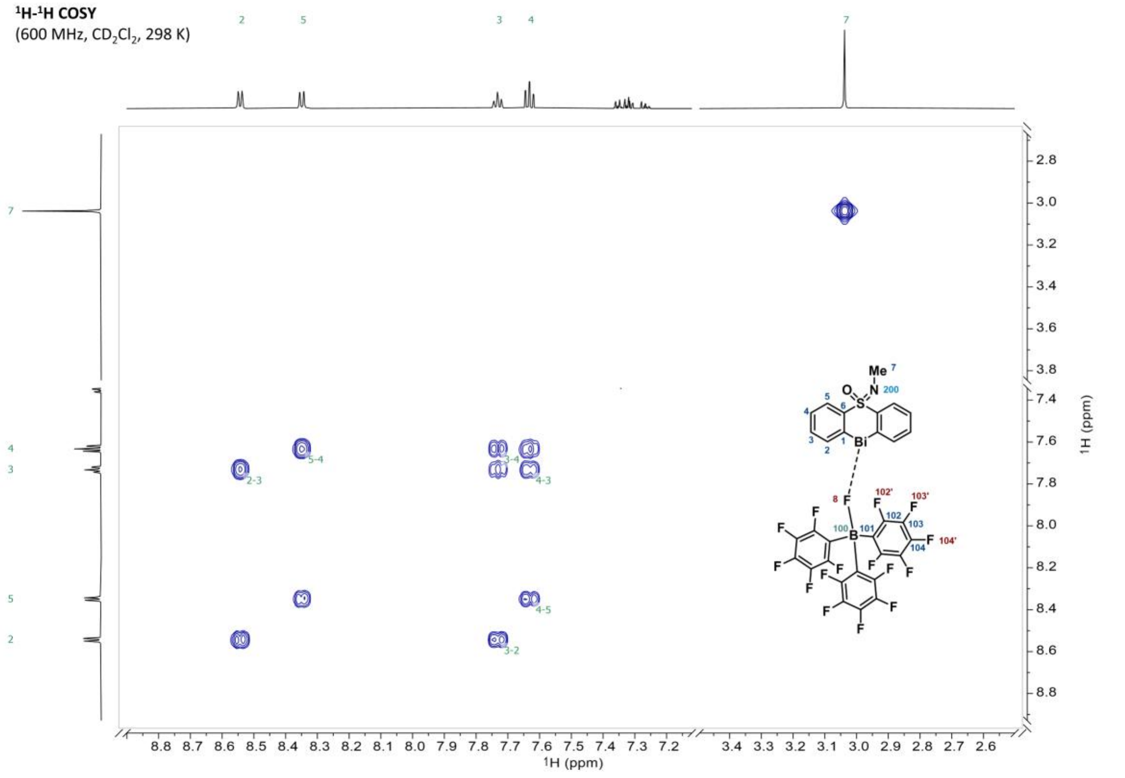

Figure S86. ^{1}H-^{1}H COSY spectrum of **[Bi-3]•B(Ar^{F})$_3$F**.

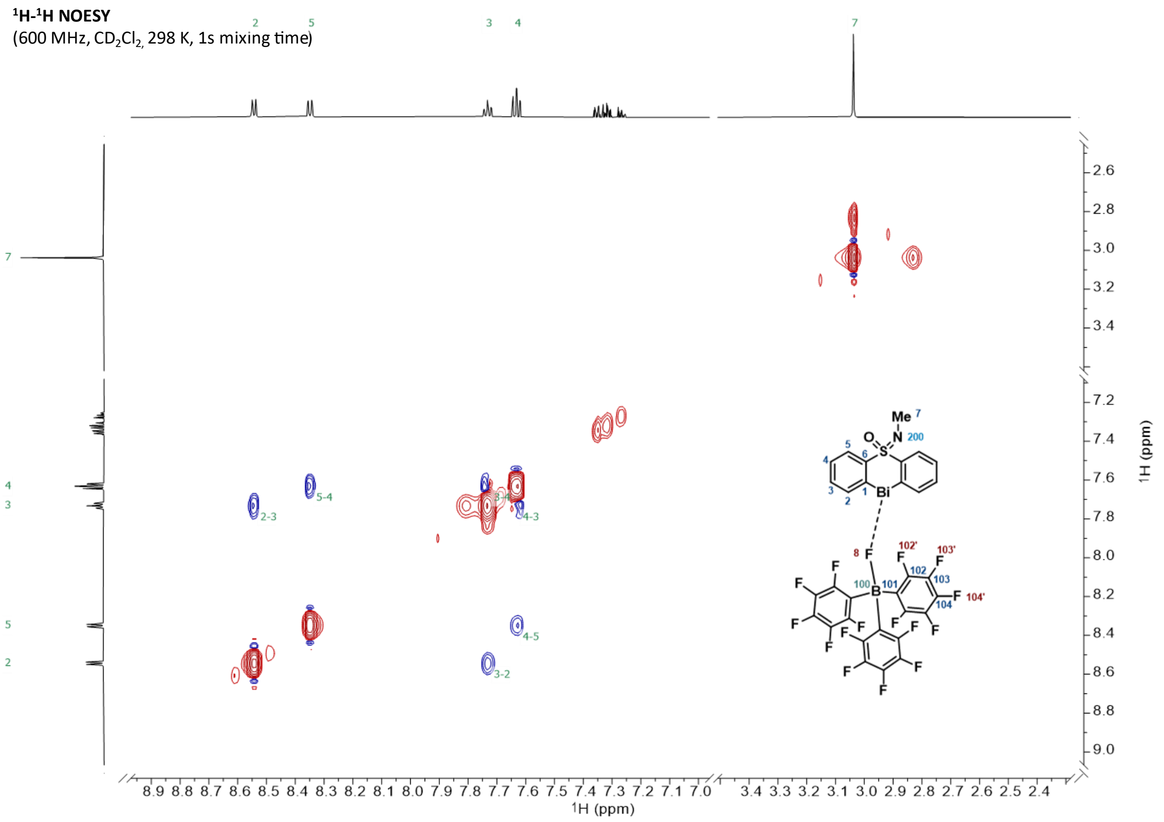

Figure S87. ^{1}H-^{1}H NOESY spectrum of **[Bi-3]•B(Ar^{F})$_{3}$F**.

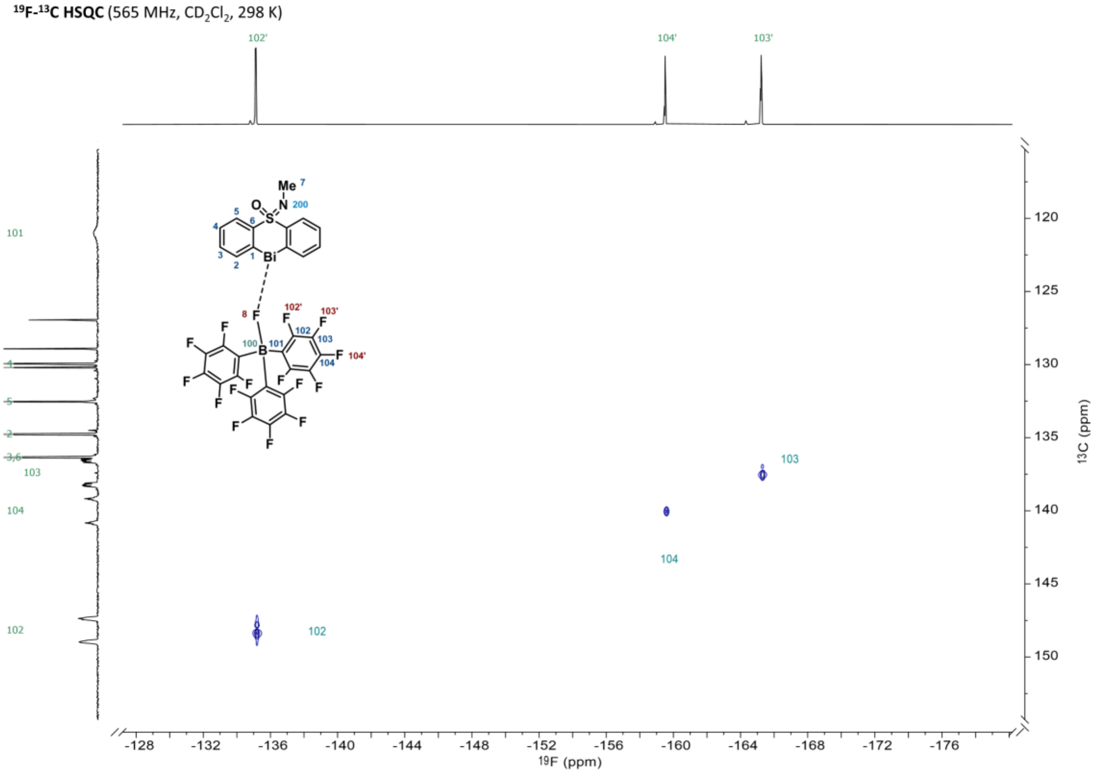

Figure S88. ^{19}F-^{13}C HSQC spectrum of **[Bi-3]•B(Ar^{F})$_{3}$F**.

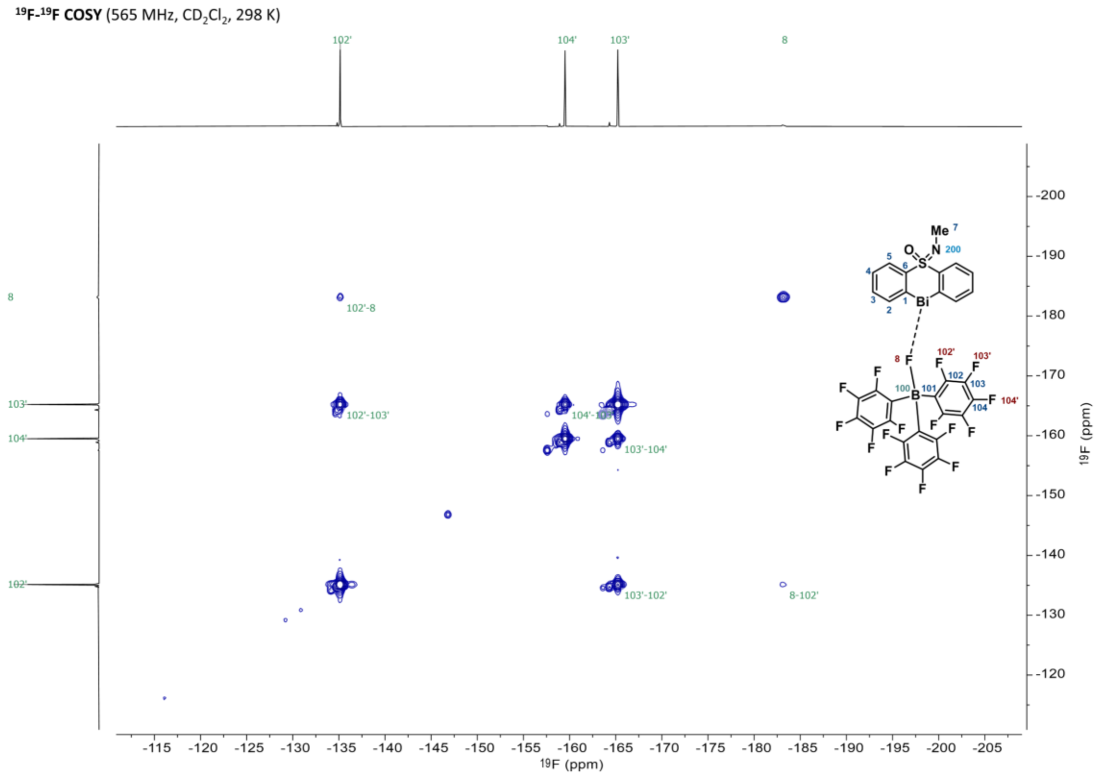

Figure S89. ^{19}F-^{19}F COSY spectrum of **[Bi-3]•B(Ar^F)$_3$F**.

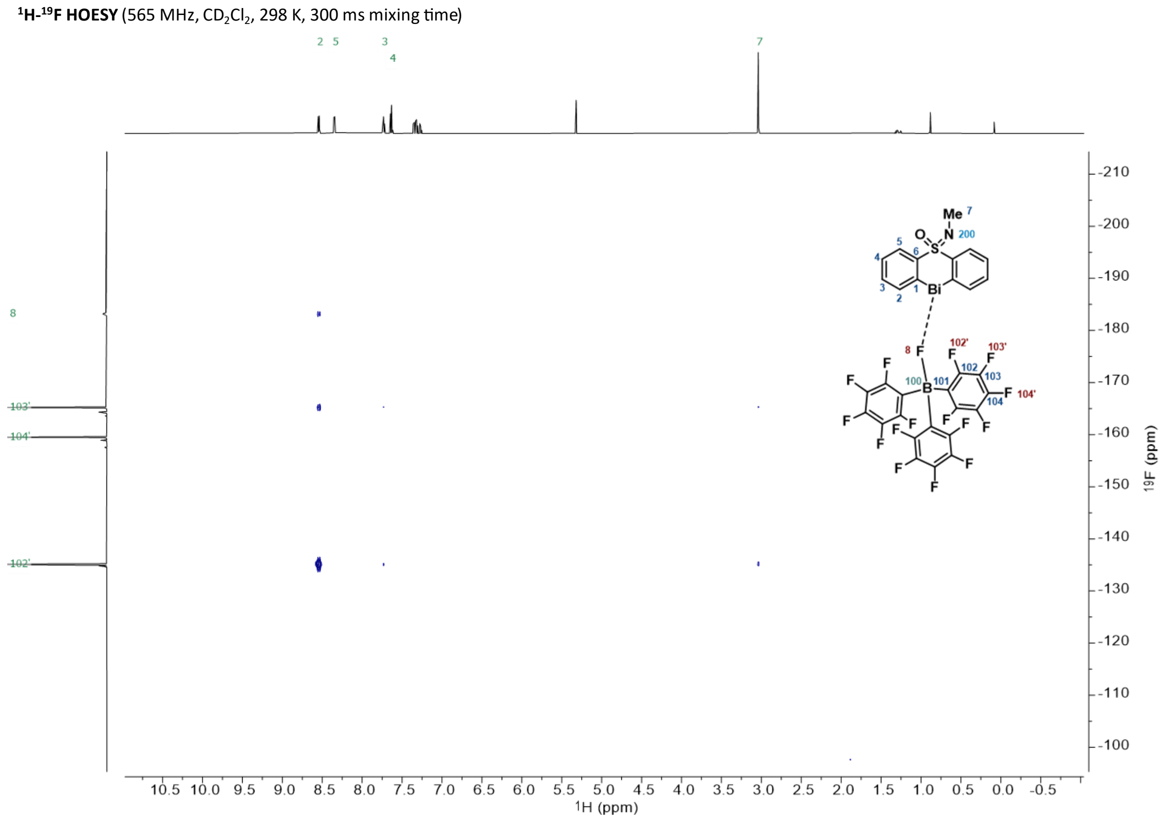

^{1}H-^{19}F HOESY (500 MHz, CD_2Cl_2, 298 K, 300 ms mixing time)

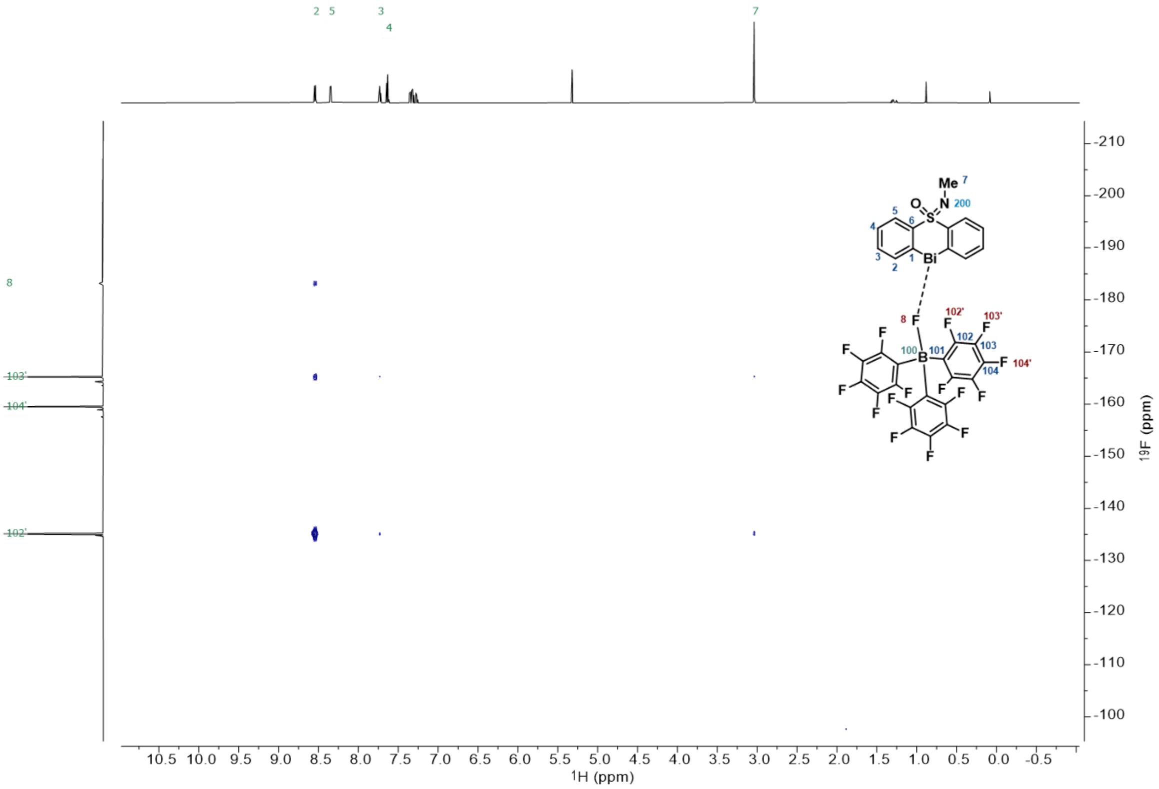

Figure S90. ^{1}H-^{19}F HOESY spectrum of **[Bi-3]•B(Ar^F)$_3$F**.

^{1}H-^{15}N HMBC (600 MHz, CD_2Cl_2, 298 K)

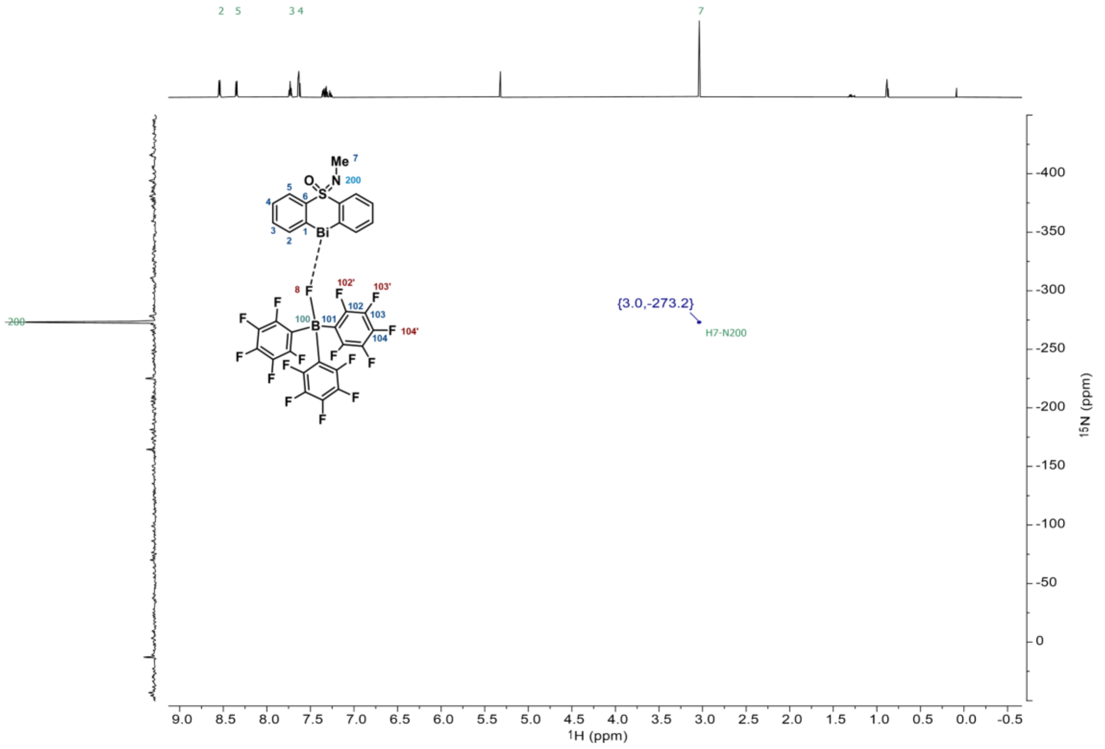

Figure S91. ^{1}H-^{15}N HMBC spectrum of **[Bi-3]•B(Ar^F)$_3$F**.

10.8 [Bi-2]•B(Ar^F)$_3$OH

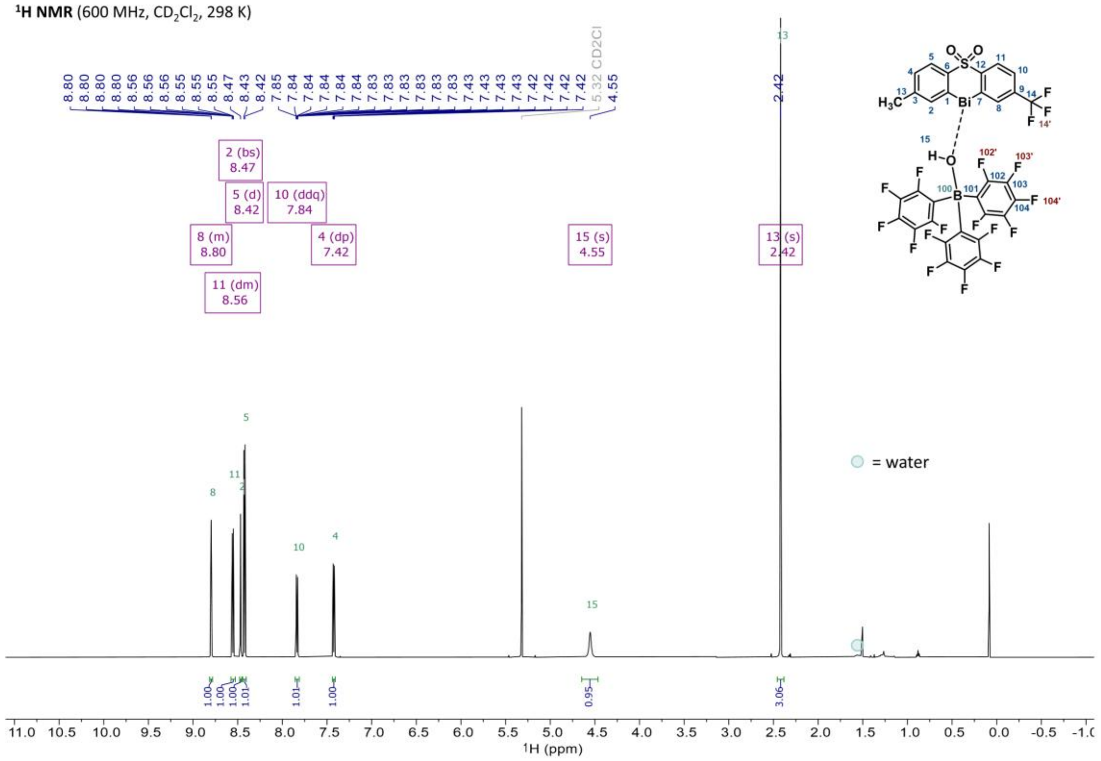

Figure S92. ^{1}H NMR spectrum of **[Bi-2]•B(Ar^F)$_3$OH**.

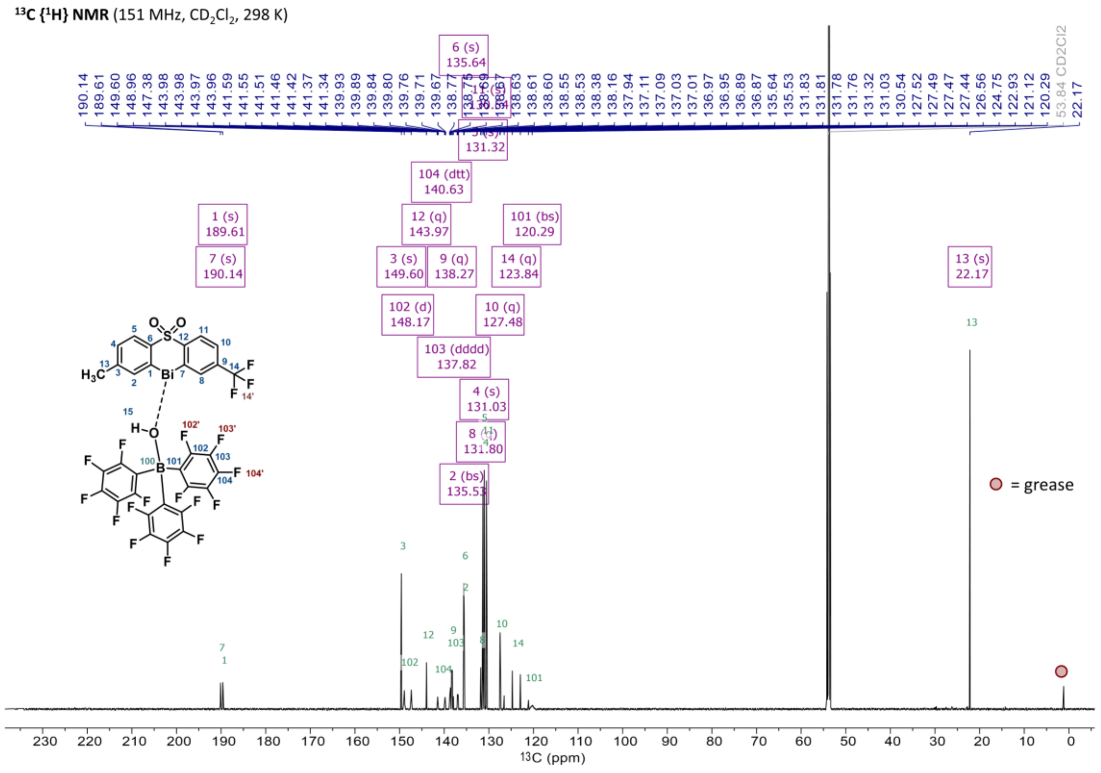

Figure S93. ^{13}C{^{1}H} NMR spectrum of **[Bi-2]•B(Ar^F)$_3$OH**.

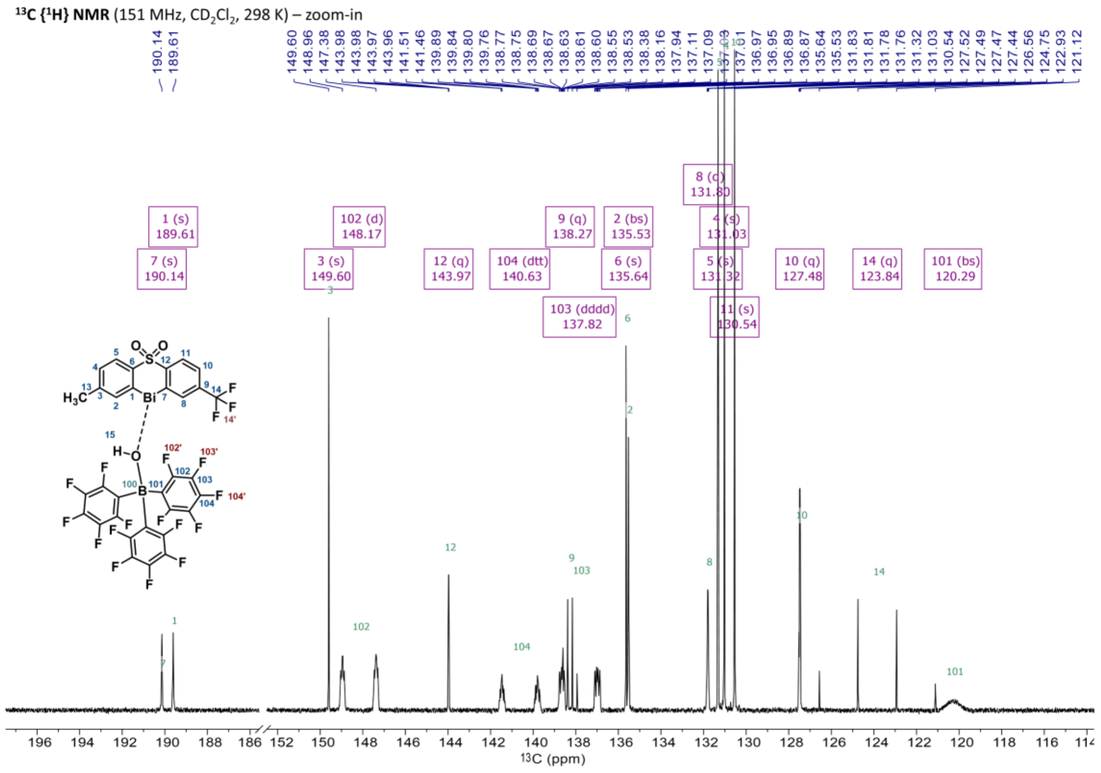

Figure S94. Zoom-in in the $^{13}C\{^{1}H\}$ NMR spectrum of **[Bi-2]•B(Ar^{F})$_3$OH**.

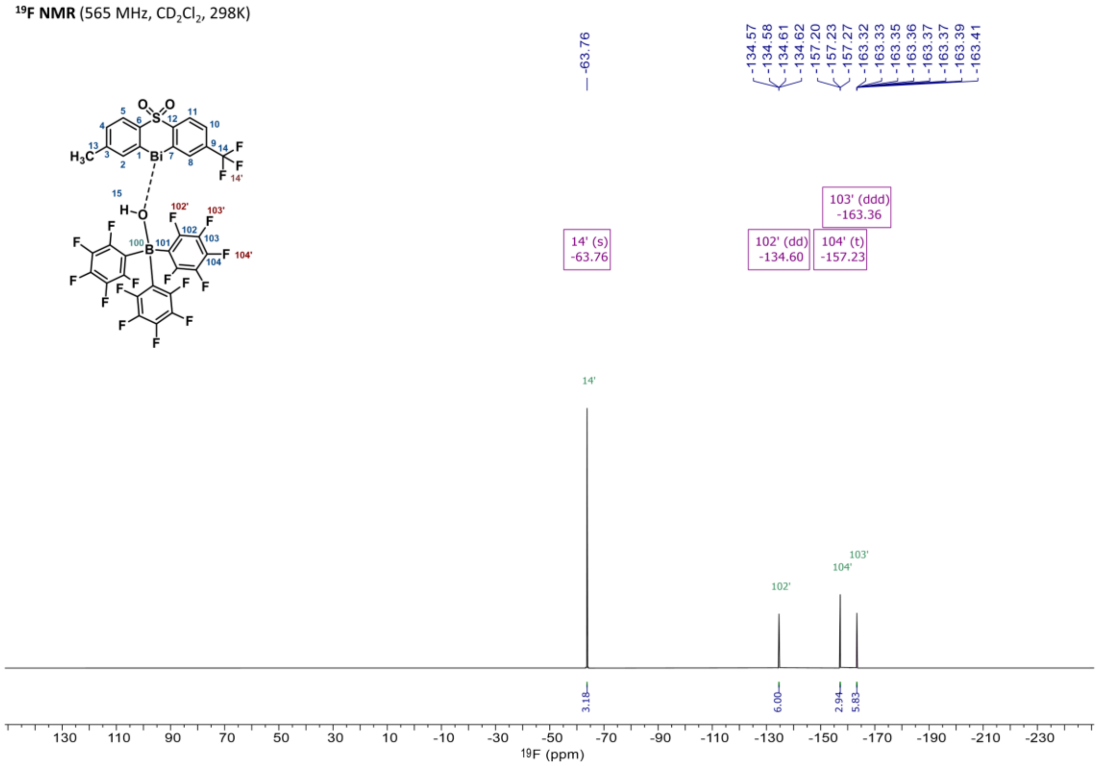

Figure S95. ^{19}F NMR spectrum of **[Bi-2]•B(Ar^{F})$_3$OH**.

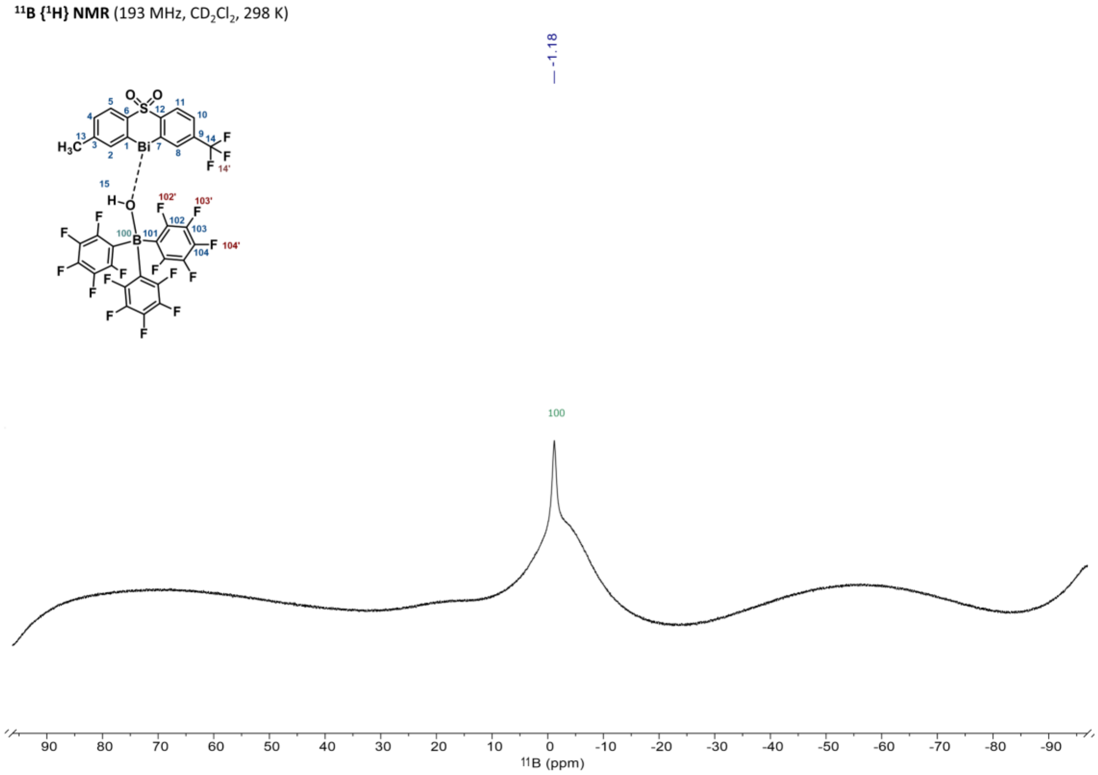

Figure S96. ^{11}B{^{1}H} NMR spectrum of **[Bi-2]•B(Ar^{F})$_{3}$OH**.

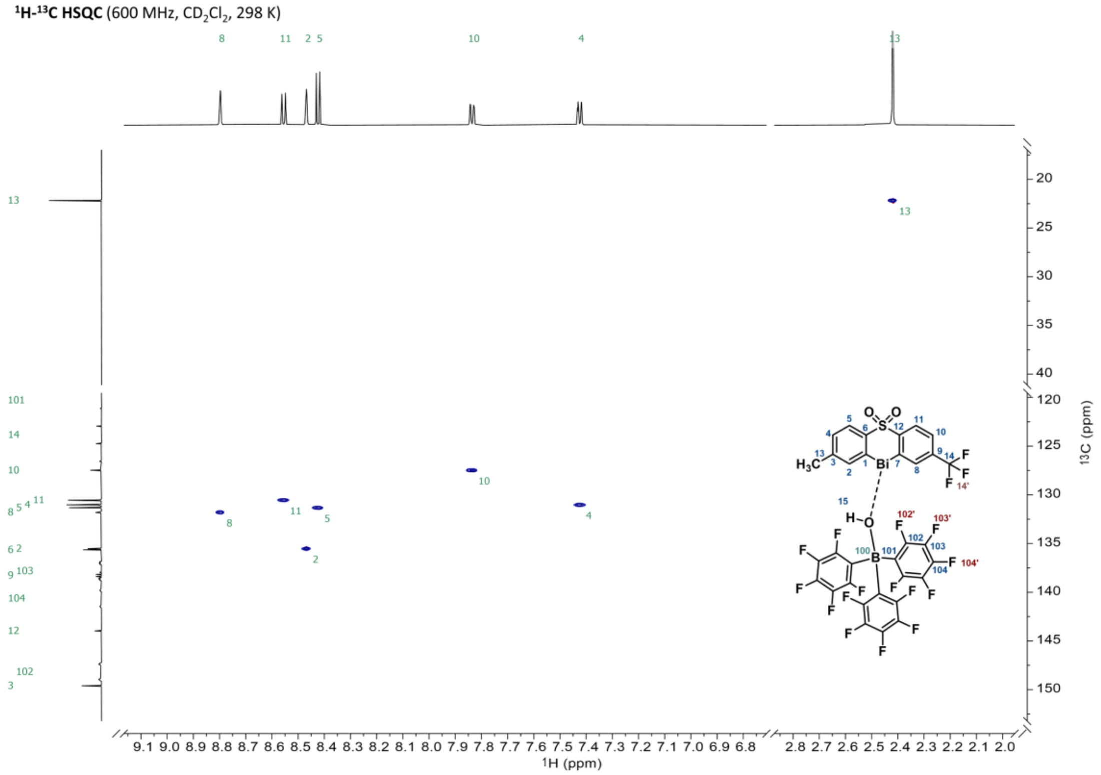

Figure S97. ^{1}H-^{13}C HSQC spectrum of **[Bi-2]•B(Ar^{F})$_{3}$OH**.

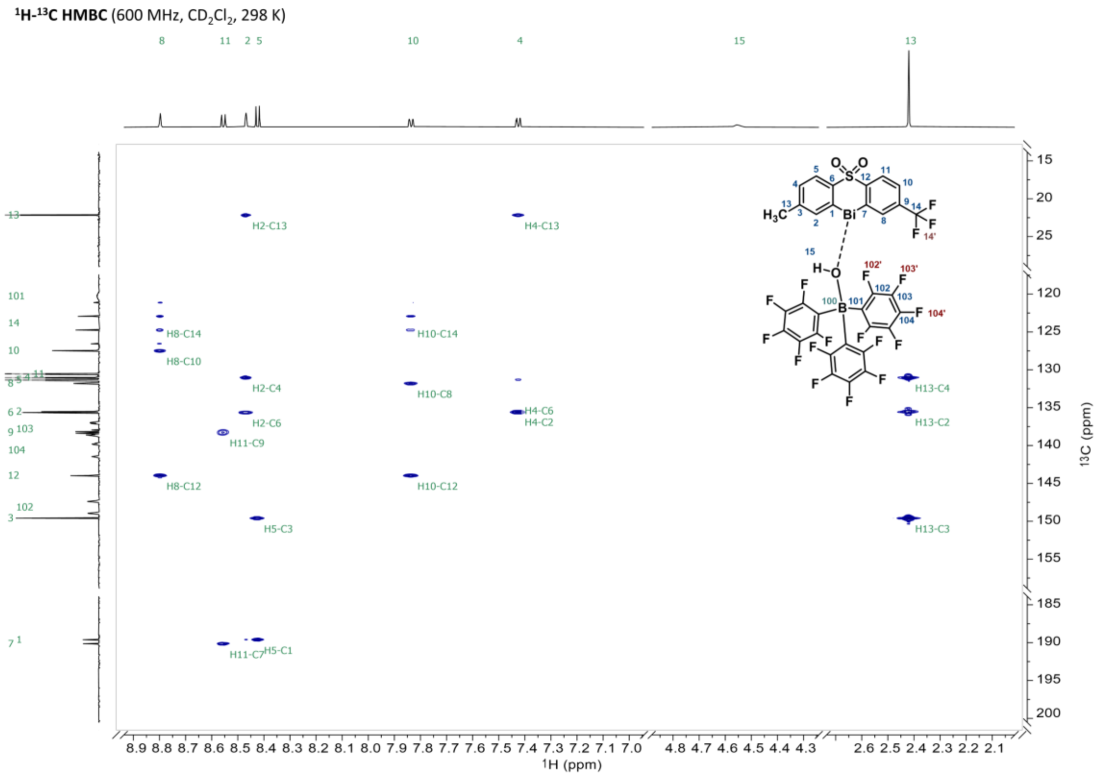

Figure S98. ^{1}H-^{13}C HMBC spectrum of **[Bi-2]•B(Ar^F)$_3$F**.

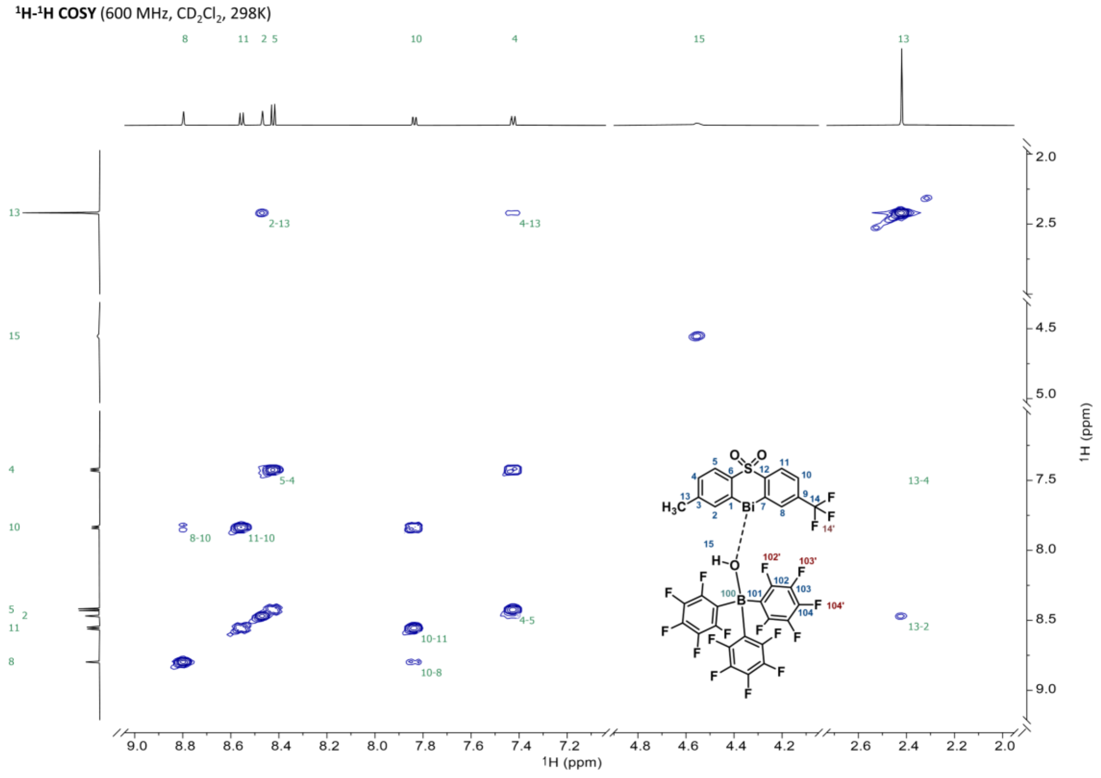

Figure S99. ^{1}H-^{1}H COSY spectrum of **[Bi-2]•B(Ar^F)$_3$OH**.

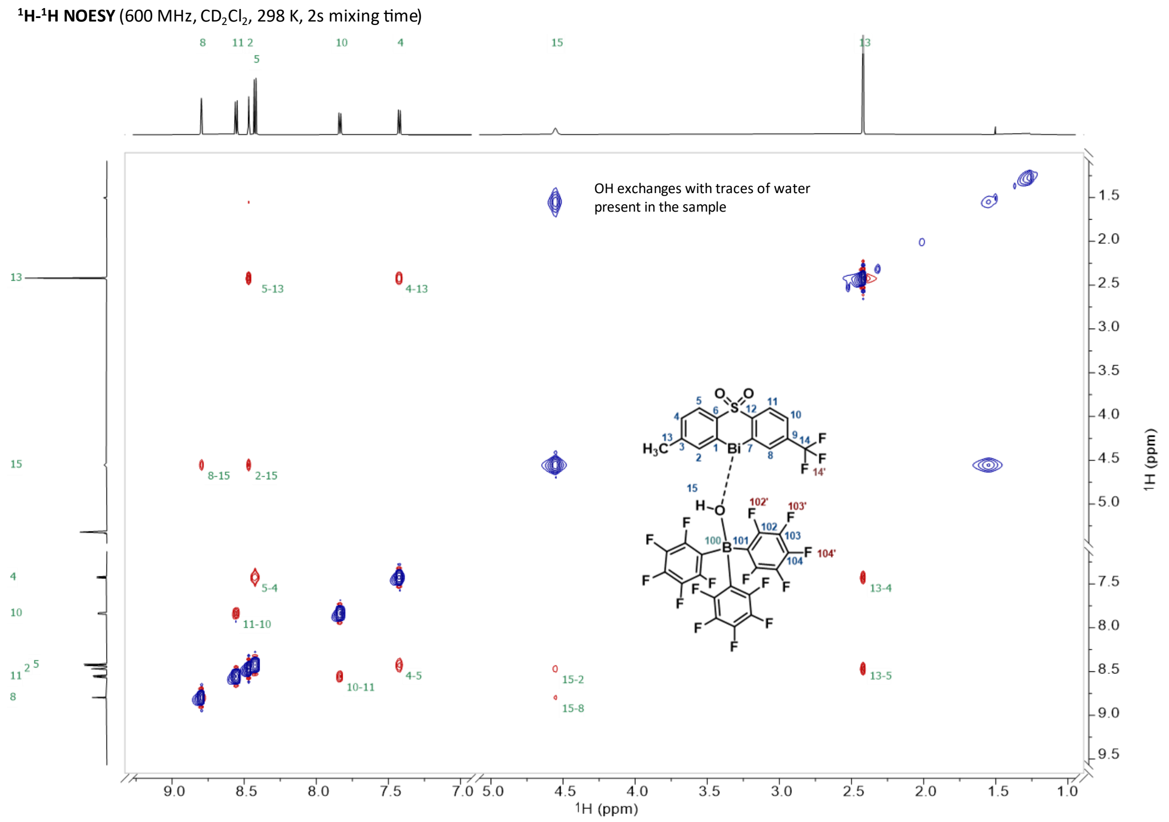

Figure S100. ^{1}H-^{1}H NOESY spectrum of **[Bi-2]•B(Ar^F)$_3$OH**.

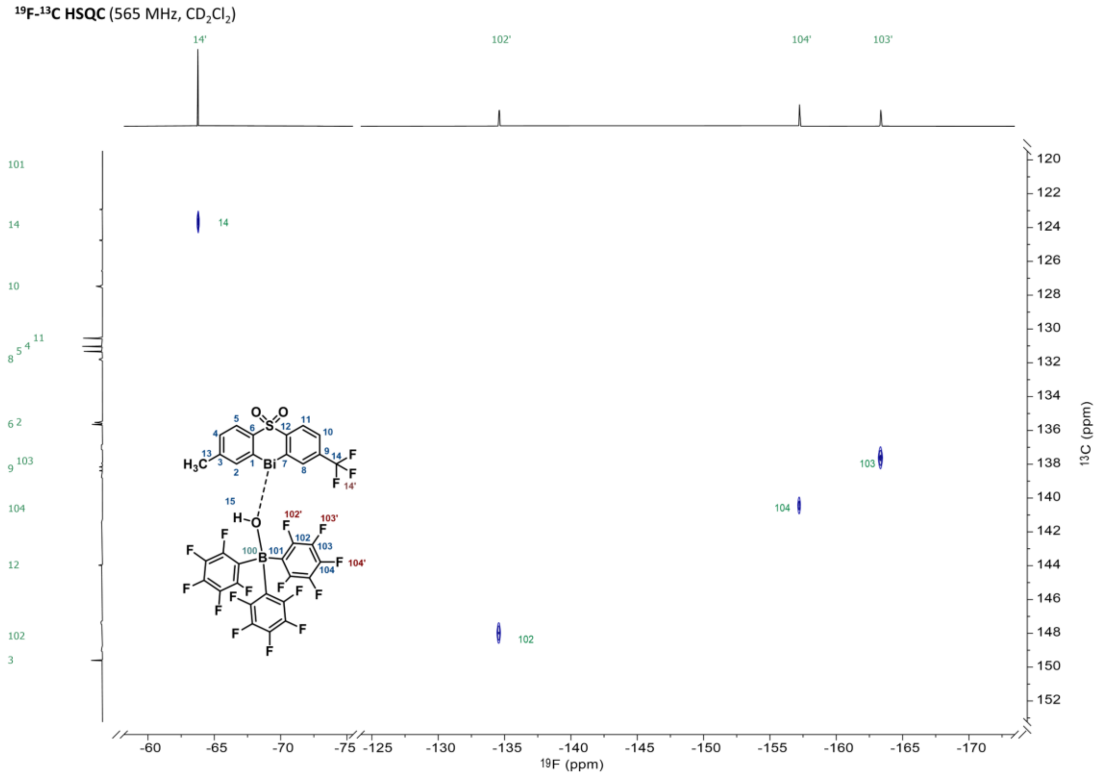

Figure S101. ^{19}F-^{13}C HSQC spectrum of **[Bi-2]•B(Ar^F)$_3$OH**

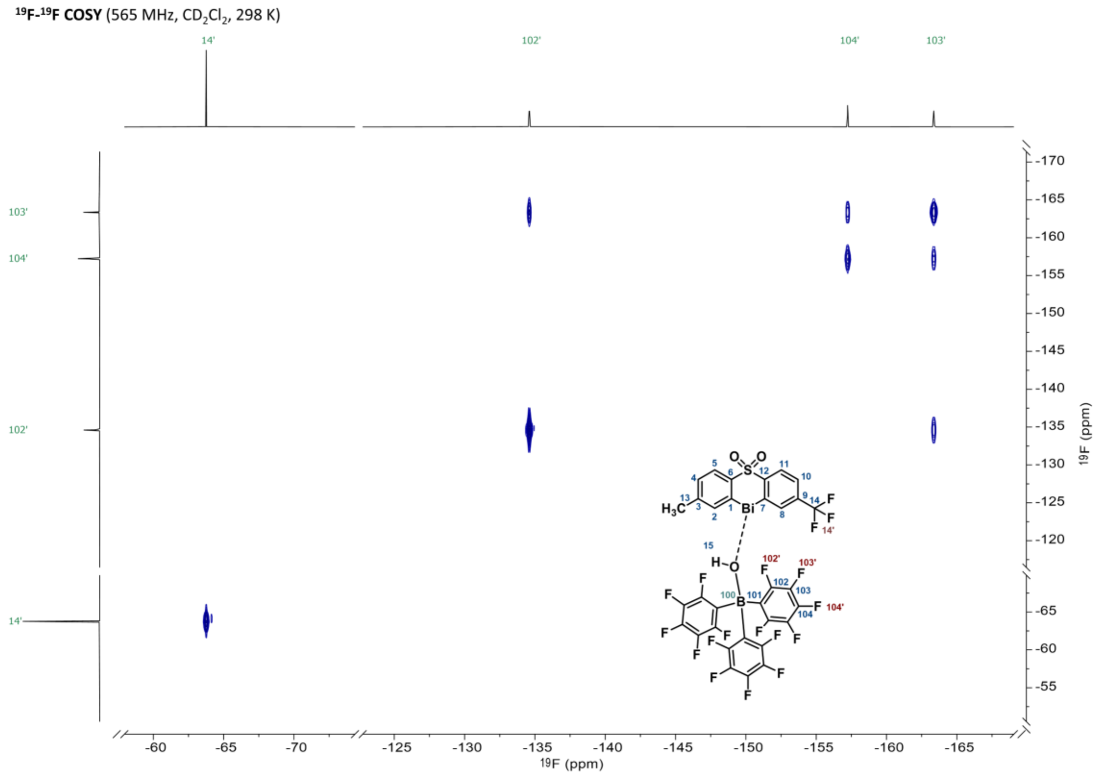

Figure S102. ^{19}F-^{19}F COSY spectrum of **[Bi-2]•B(Ar^F)$_3$OH**.

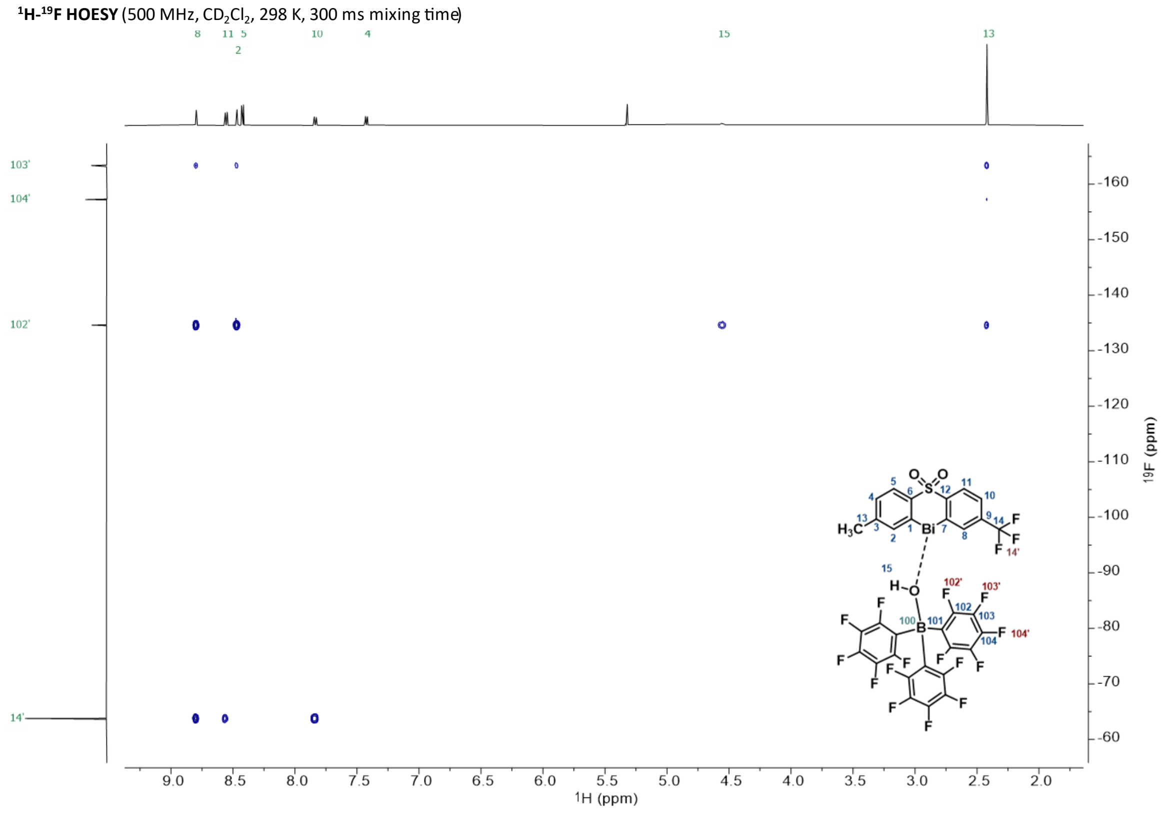

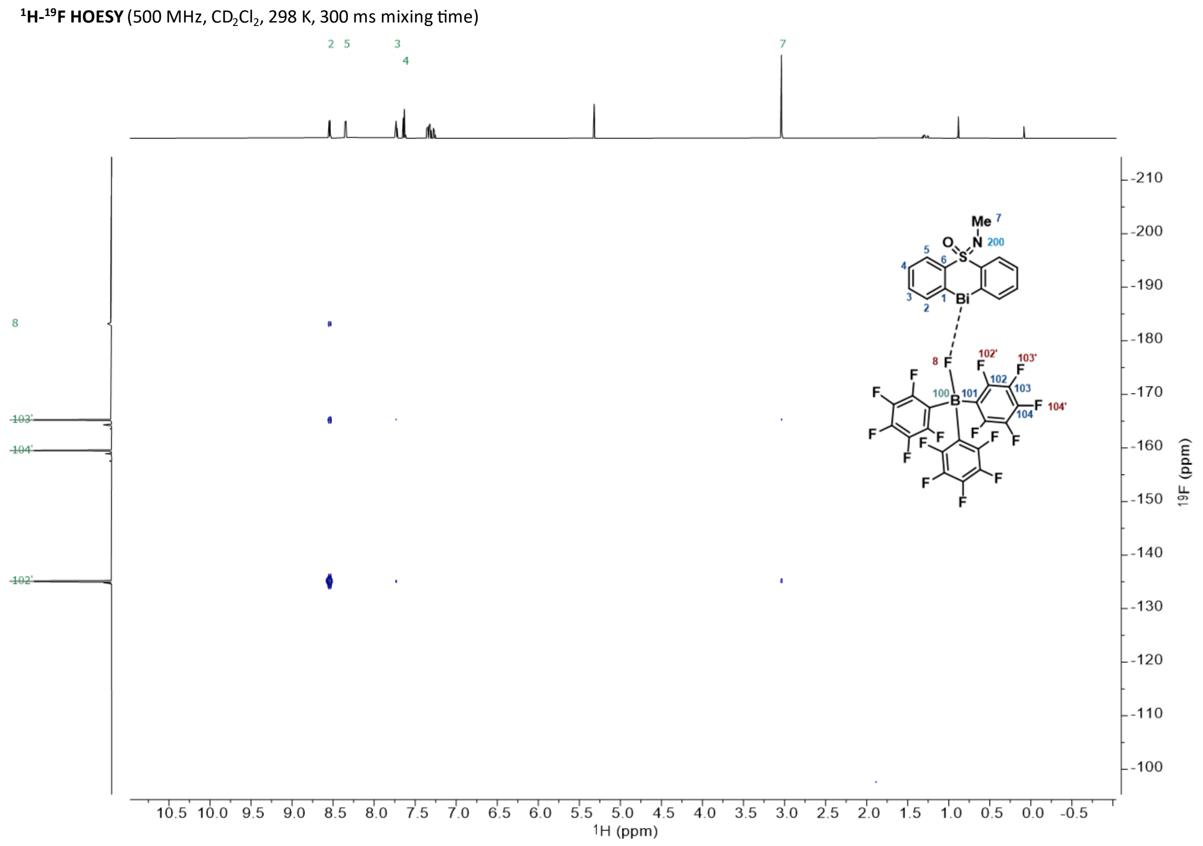

Figure S103. ^{1}H-^{19}F HOESY spectrum of **[Bi-2]•B(Ar^F)$_3$OH**.

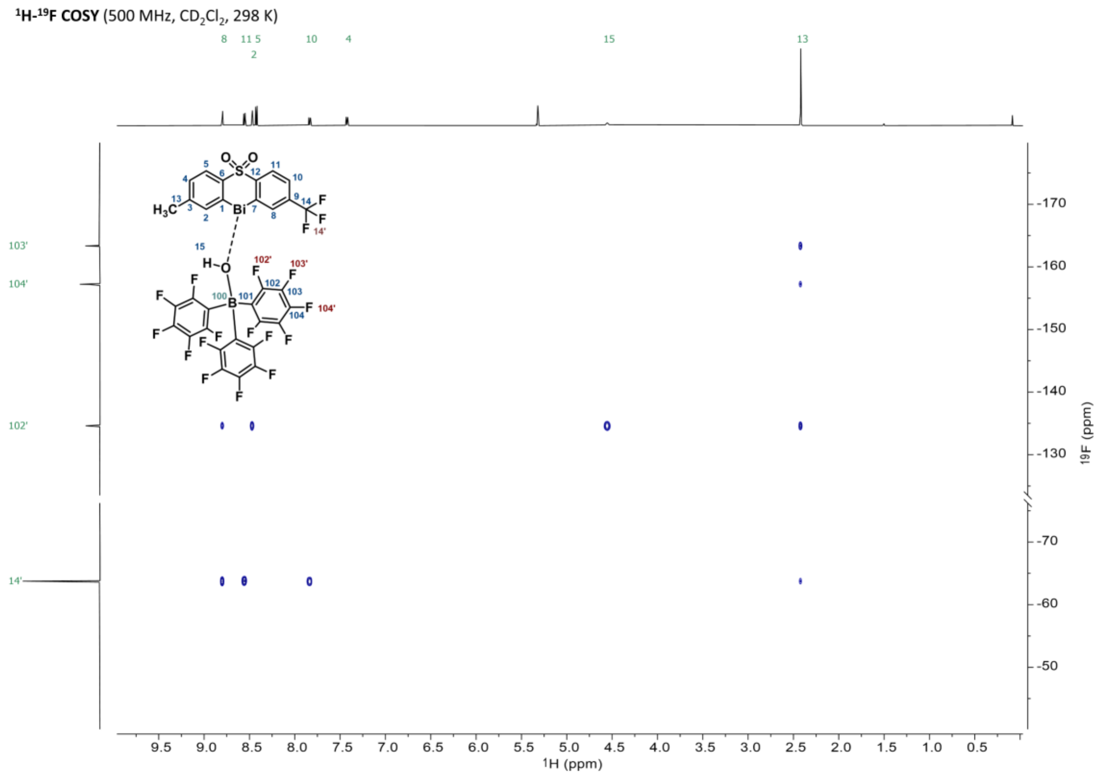

Figure S104. ^{1}H-^{19}F COSY spectrum of **[Bi-2]•B(Ar^F)$_3$OH**.

10.9 [Bi-4]•B(Ar^F)_3OH

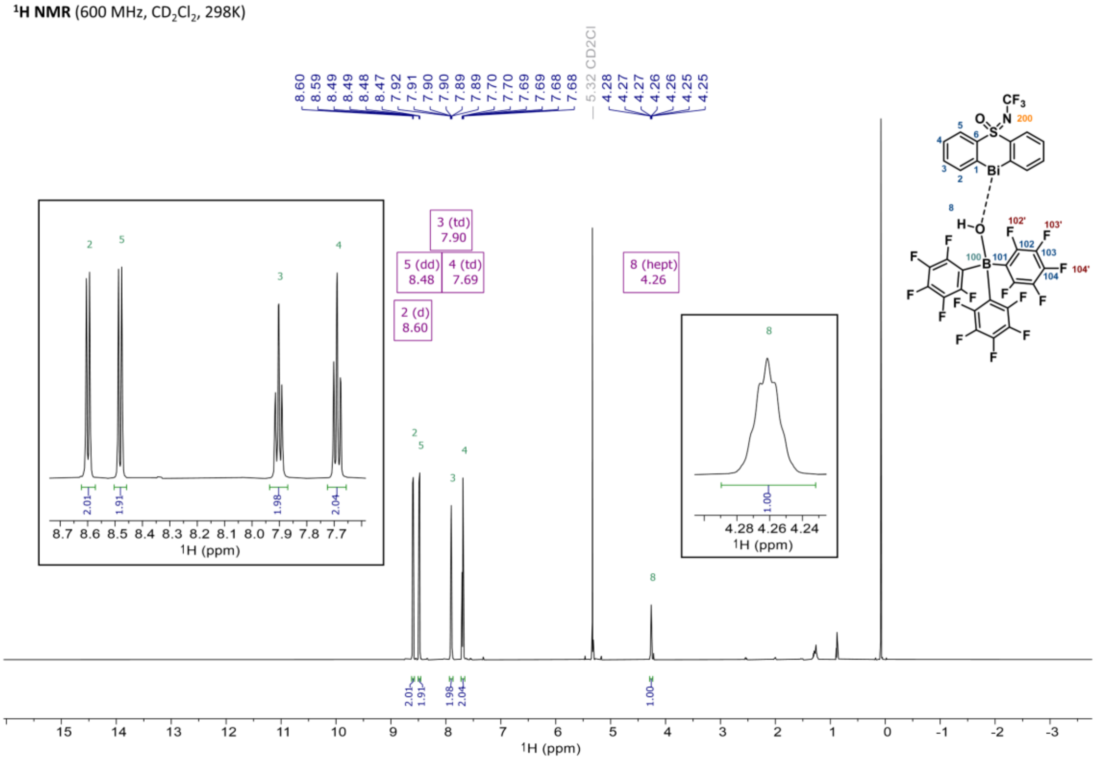

Figure S105. ¹H NMR spectrum of **[Bi-4]•B(Ar^F)₃OH**.

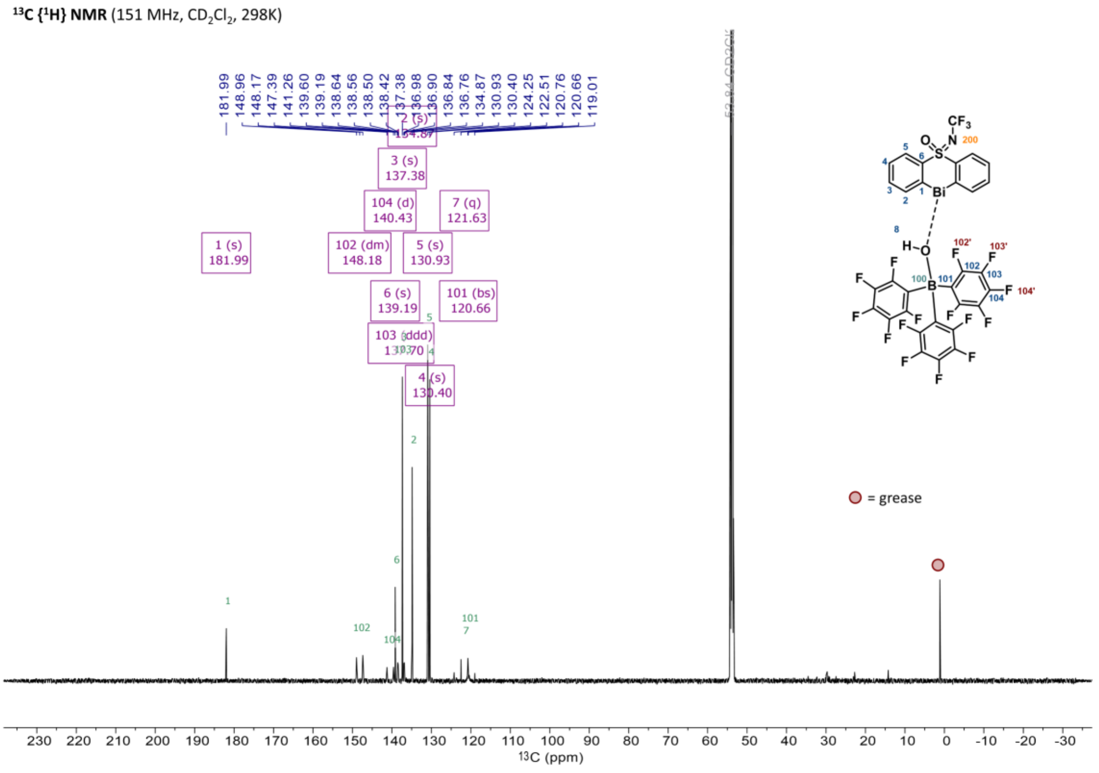

Figure S106. ¹³C {¹H} NMR spectrum of **[Bi-4]•B(Ar^F)₃OH**..

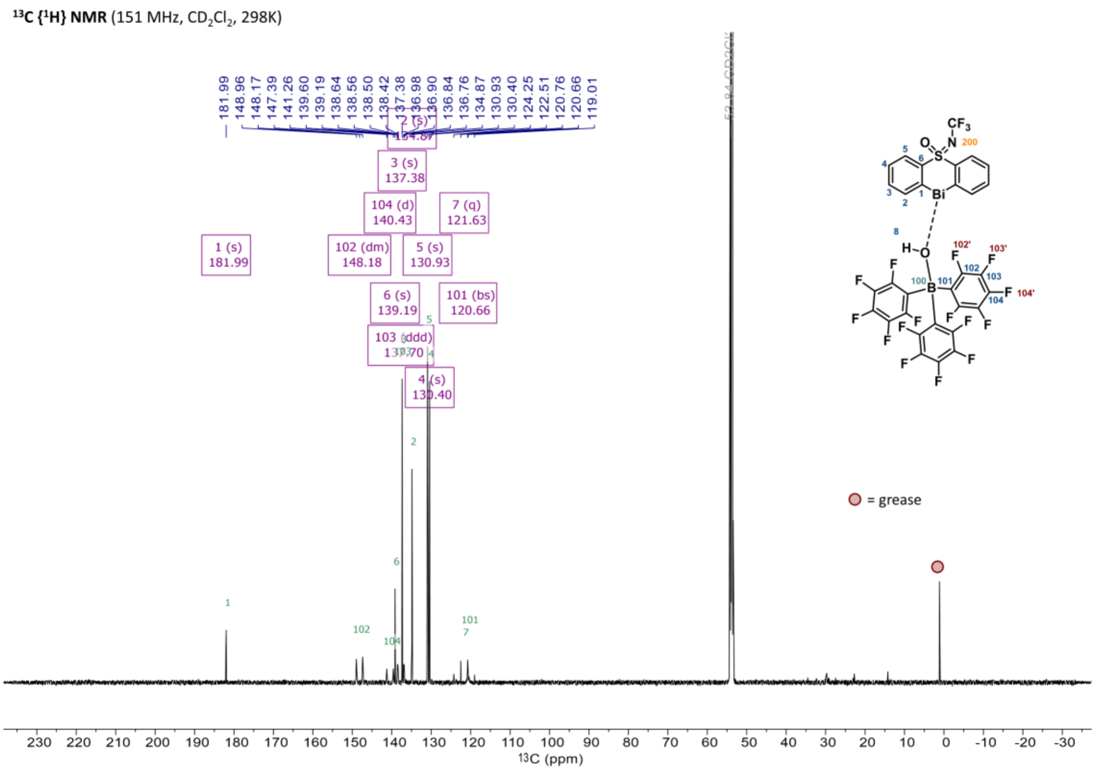

Figure S107. Zoom-in in the $^{13}C\{^1H\}$ NMR spectrum of **[Bi-4]•B(Ar^F)$_3$OH**.

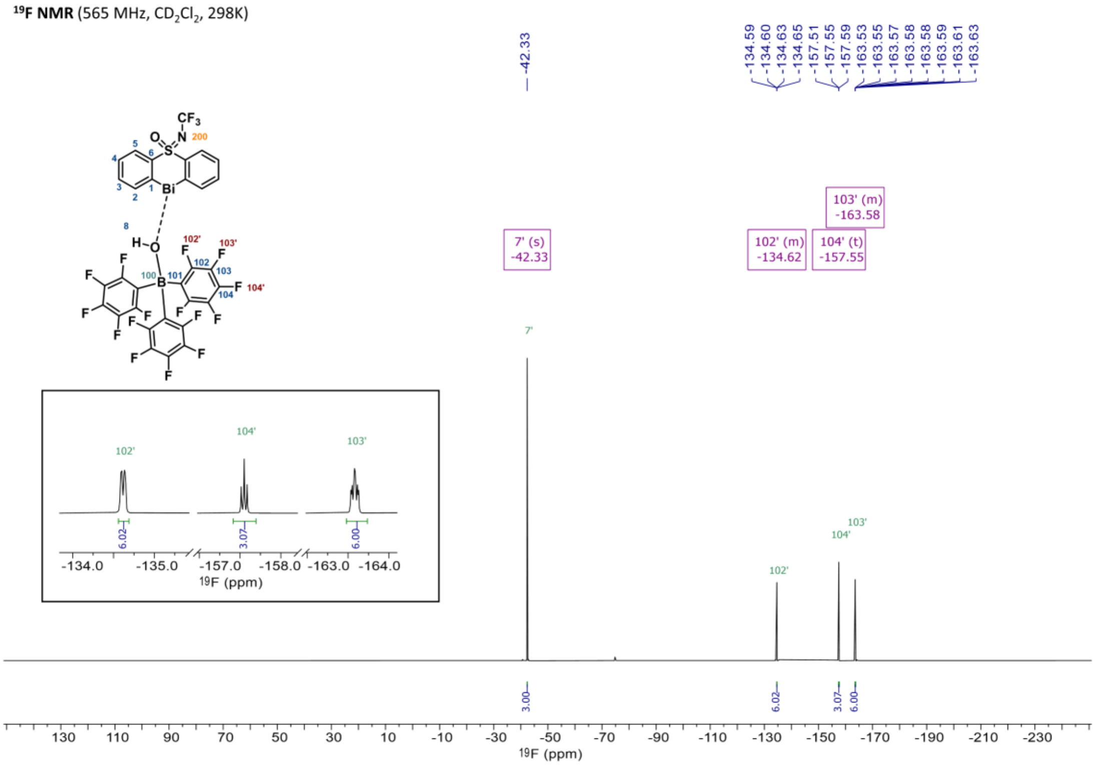

Figure S108. ^{19}F NMR spectrum of **[Bi-4]•B(Ar^F)$_3$OH**.

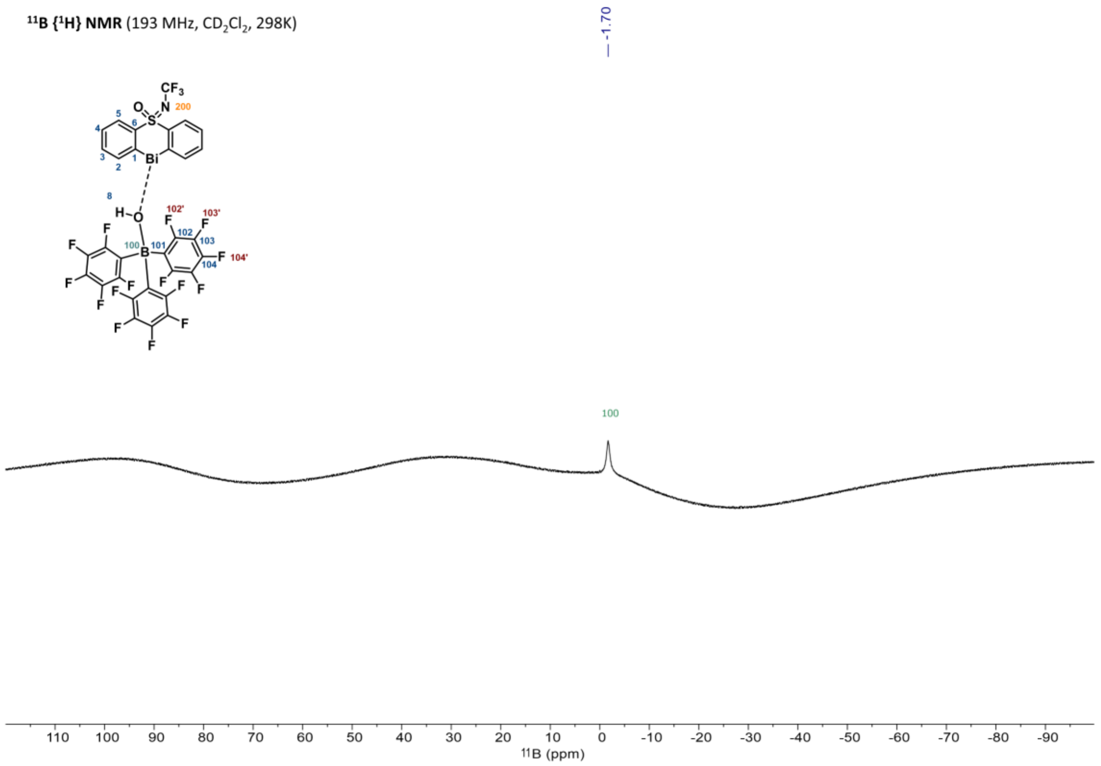

Figure S109. $^{11}B\{^{1}H\}$ NMR spectrum of **[Bi-4]•B(Ar^{F})$_3$OH**.

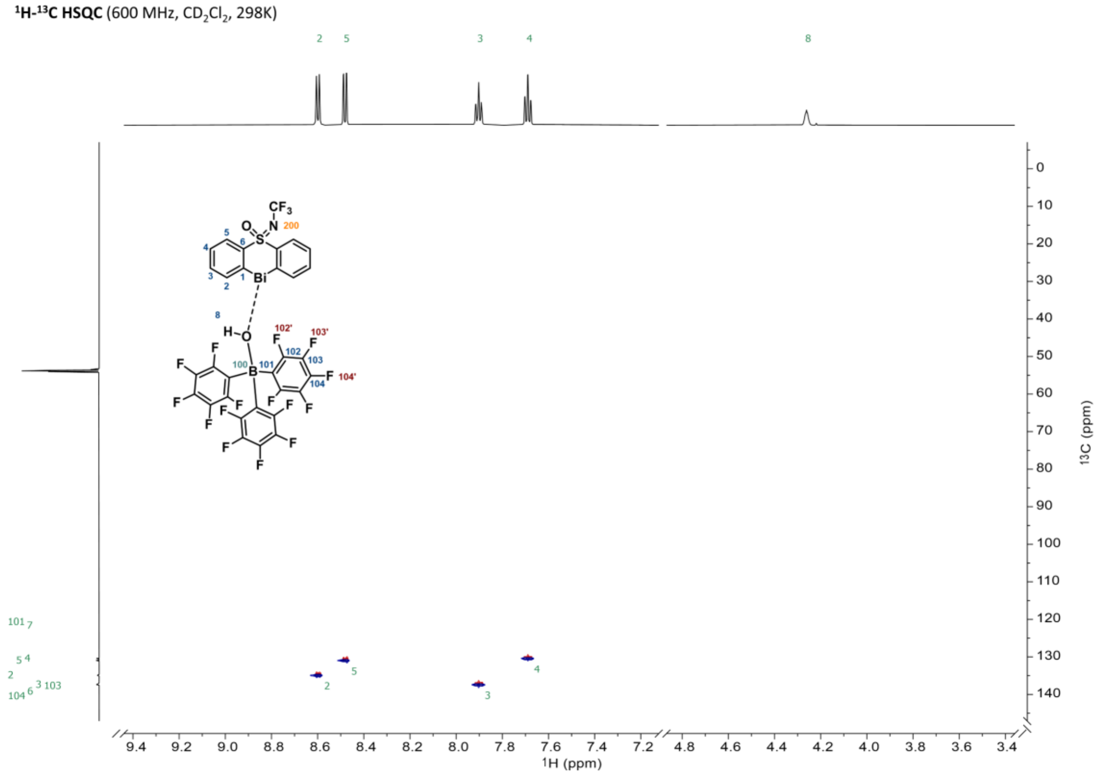

Figure S110. ^{1}H-^{13}C HSQC spectrum of **[Bi-4]•B(Ar^{F})$_3$OH**.

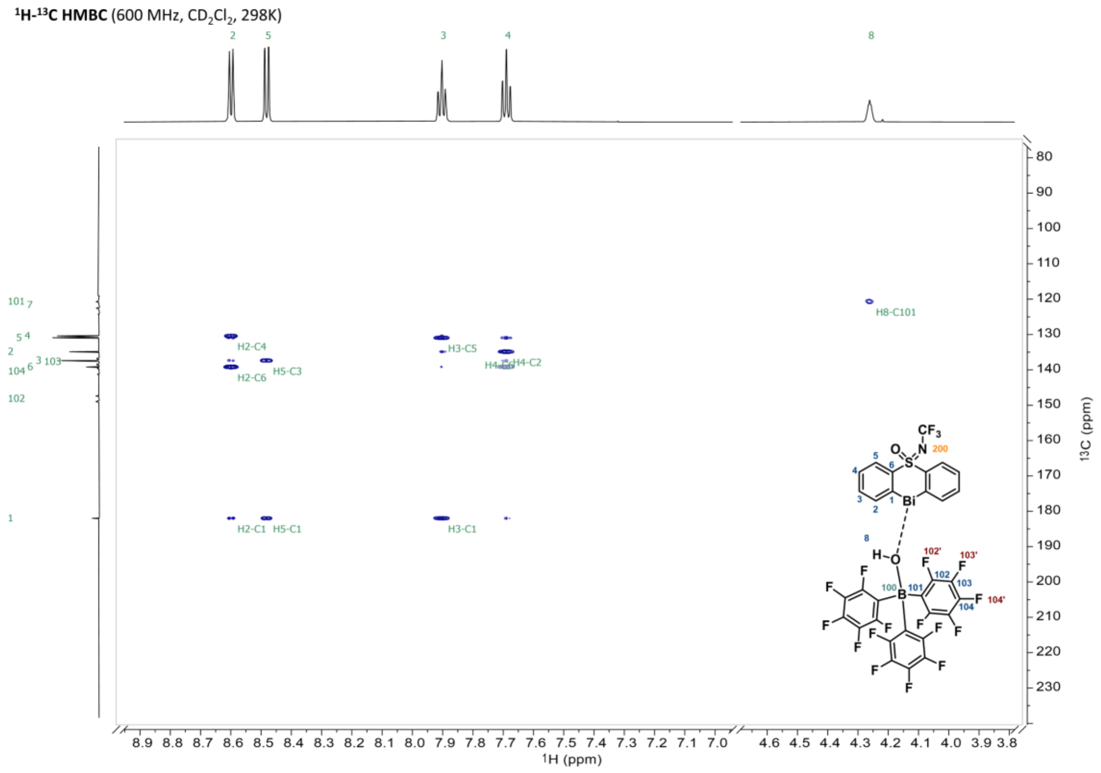

Figure S111. ^{1}H-^{13}C HMBC spectrum of **[Bi-4]•B(Ar^{F})$_3$OH**.

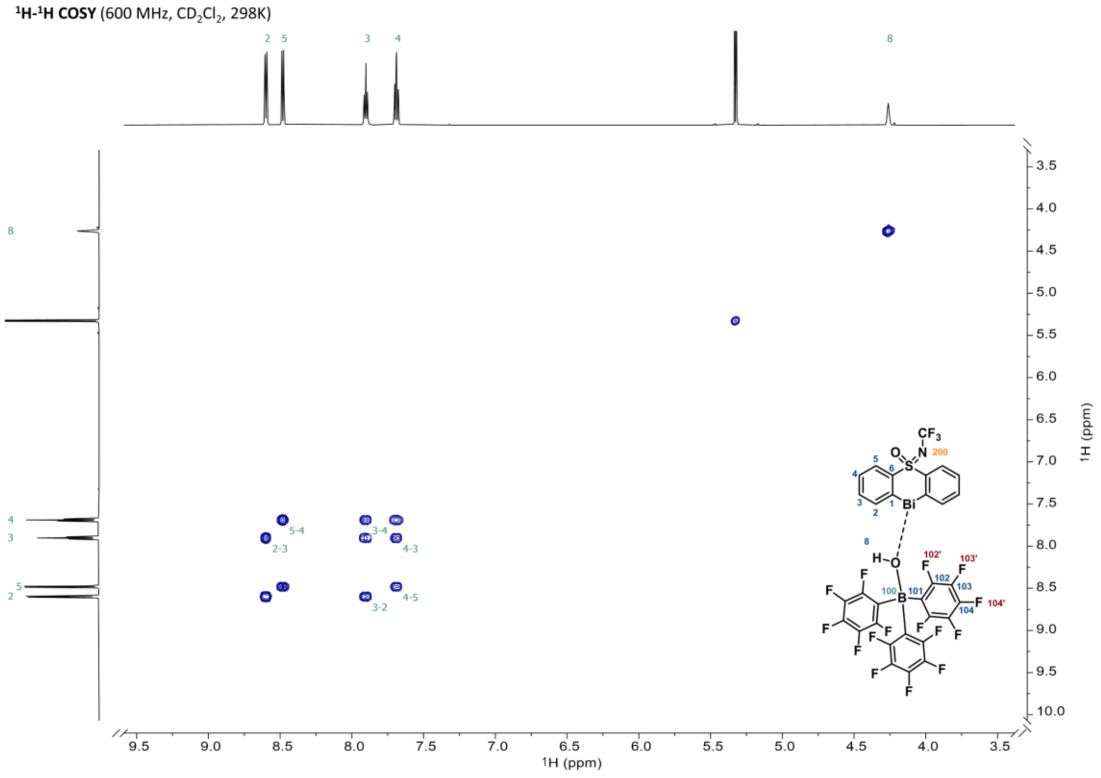

Figure S112. ^{1}H-^{1}H COSY spectrum of **[Bi-4]•B(Ar^{F})$_3$OH**.

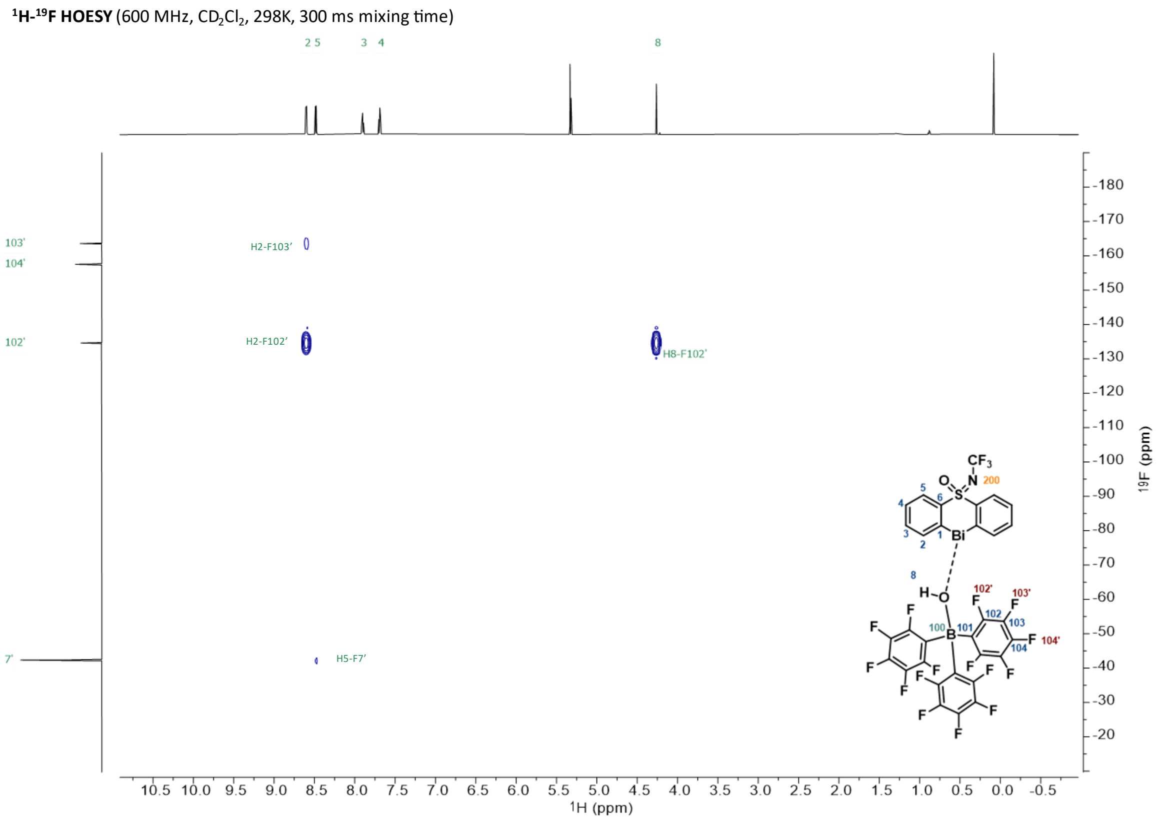

Figure S113. ^{1}H-^{1}H NOESY spectrum of **[Bi-4]•B(Ar^{F})$_{3}$OH**.

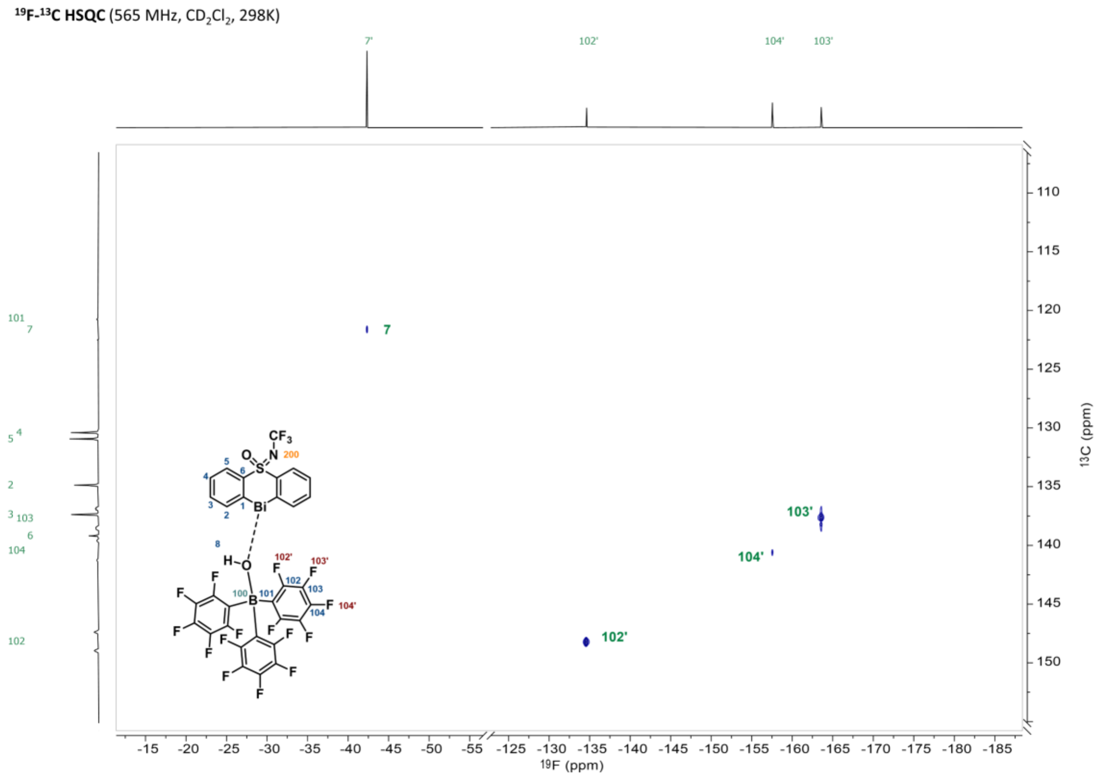

Figure S114. ^{19}F-^{13}C HSQC spectrum of **[Bi-4]•B(Ar^{F})$_{3}$OH**.

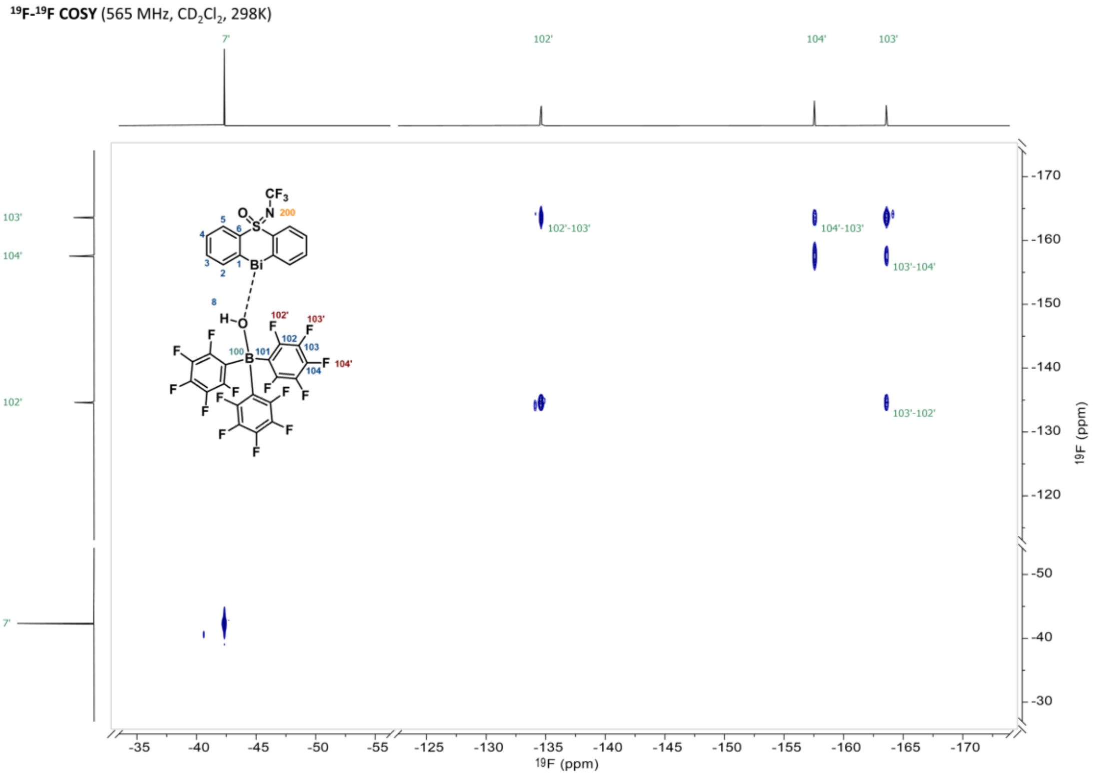

Figure S115. ^{19}F-^{19}F COSY spectrum of **[Bi-4]•B(Ar^F)$_3$OH**

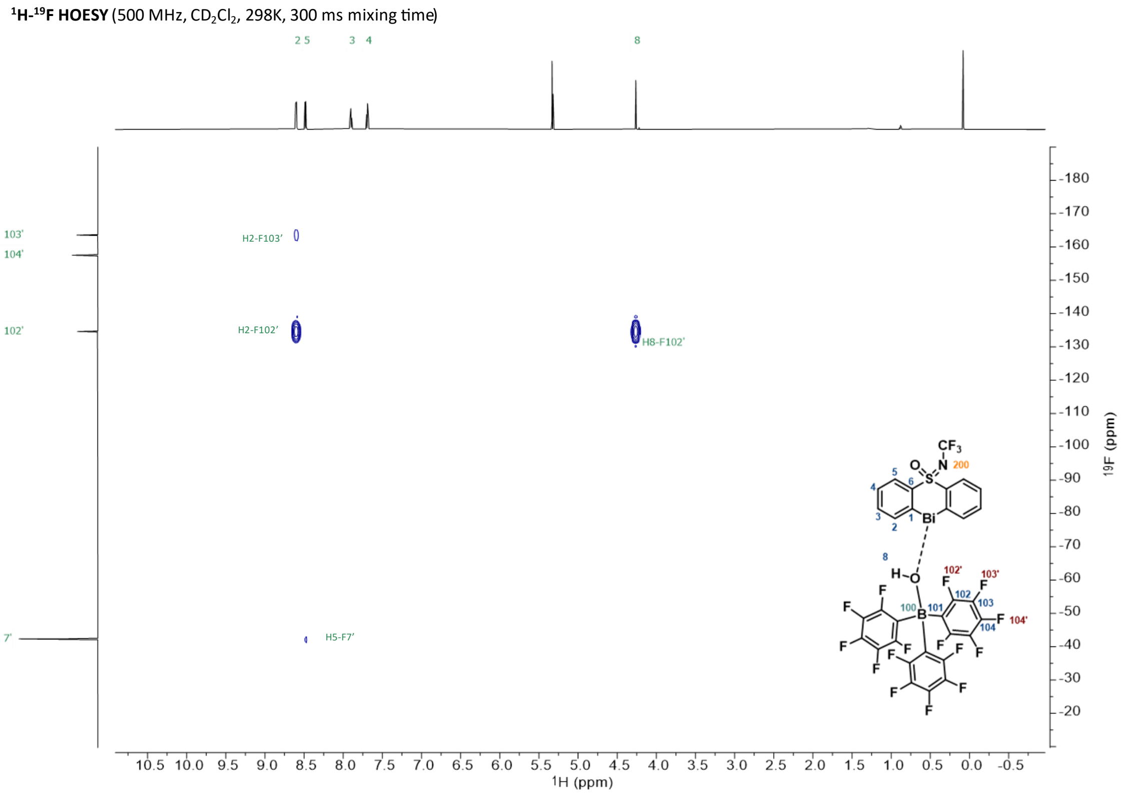

Figure S116. ^{1}H-^{19}F HOESY spectrum of **[Bi-4]•B(Ar^F)$_3$OH**.

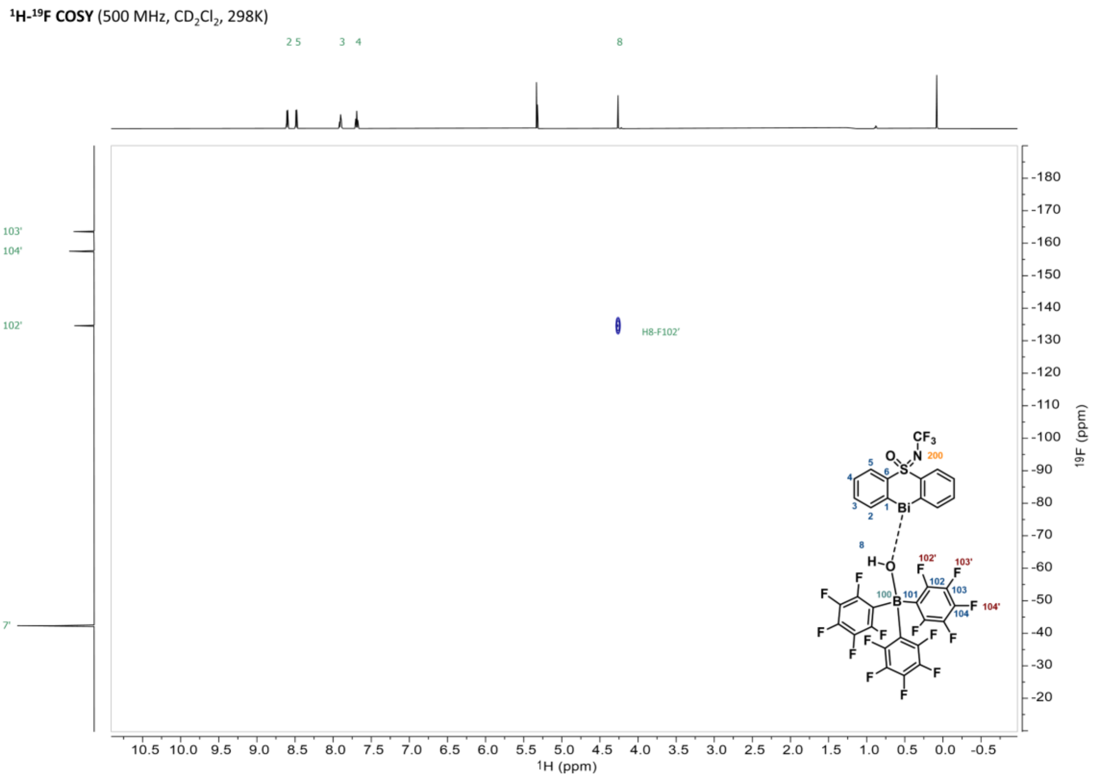

Figure S117. ^{1}H-^{19}F COSY spectrum of **[Bi-4]•B(Ar^{F})$_{3}$OH**.

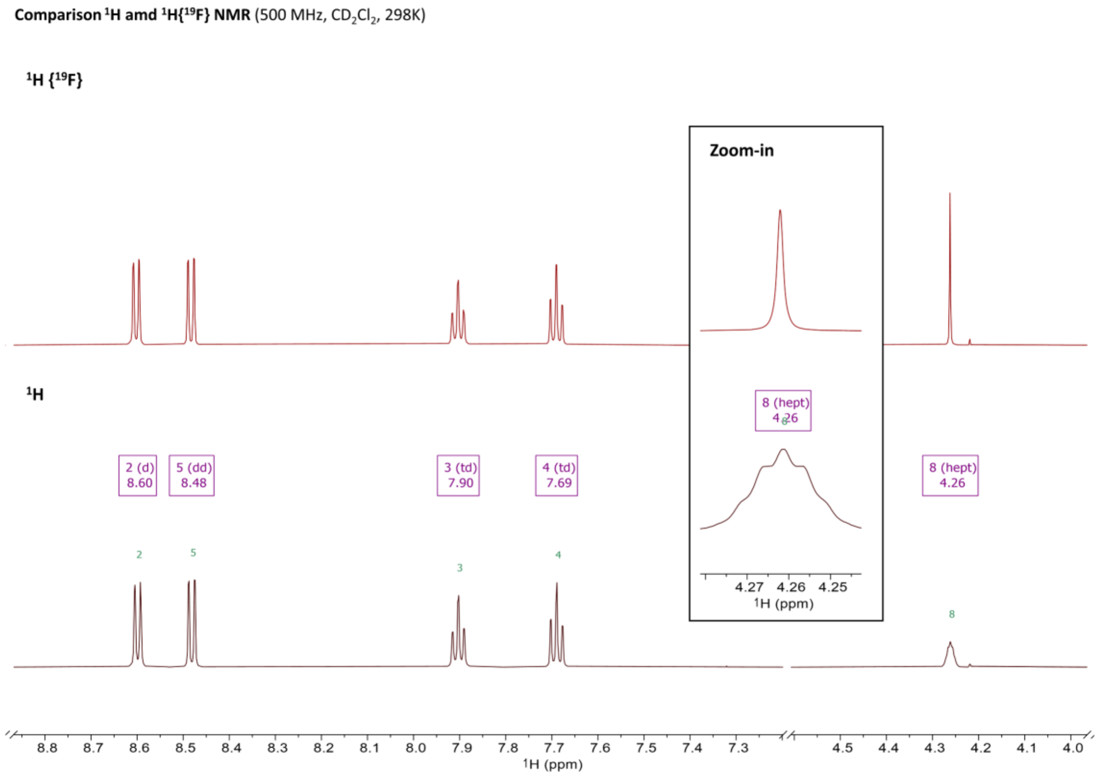

Figure S118. Comparison of ^{1}H and ^{1}H{^{19}F} spectra of **[Bi-4]•B(Ar^{F})$_{3}$OH**.

10.10 [Bi-2]•Ar^F

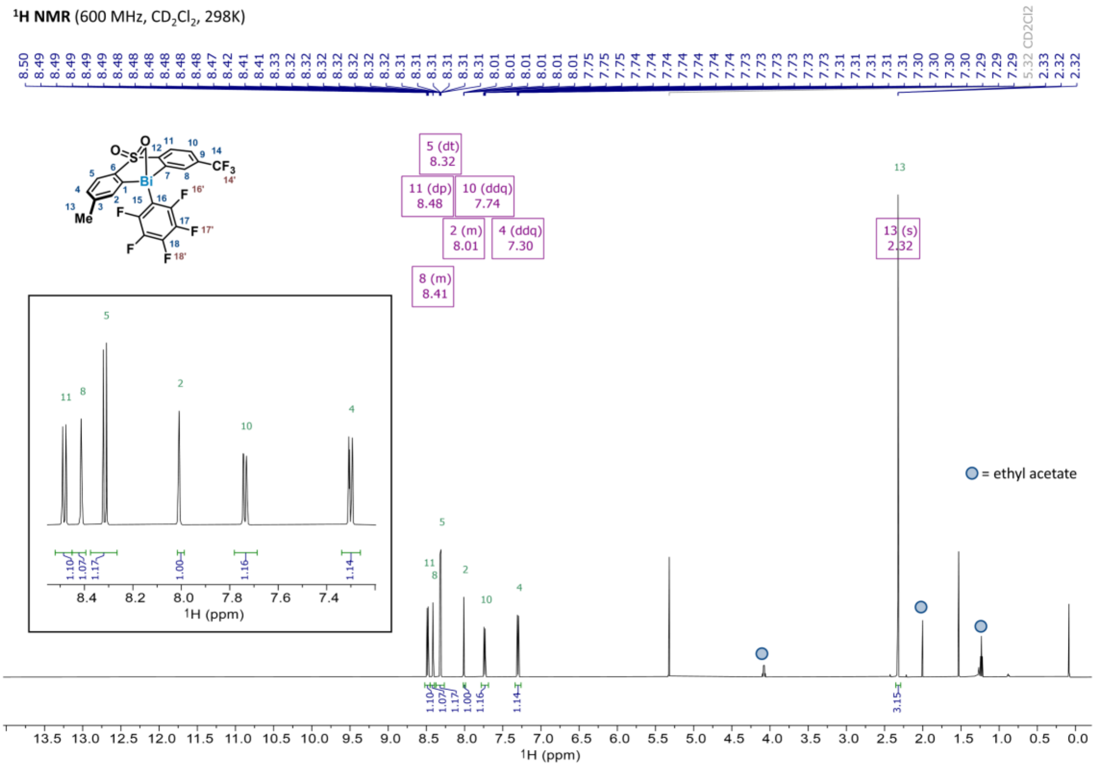

Figure S119. ^{1}H NMR spectrum of **[Bi-2]•Ar^F**.

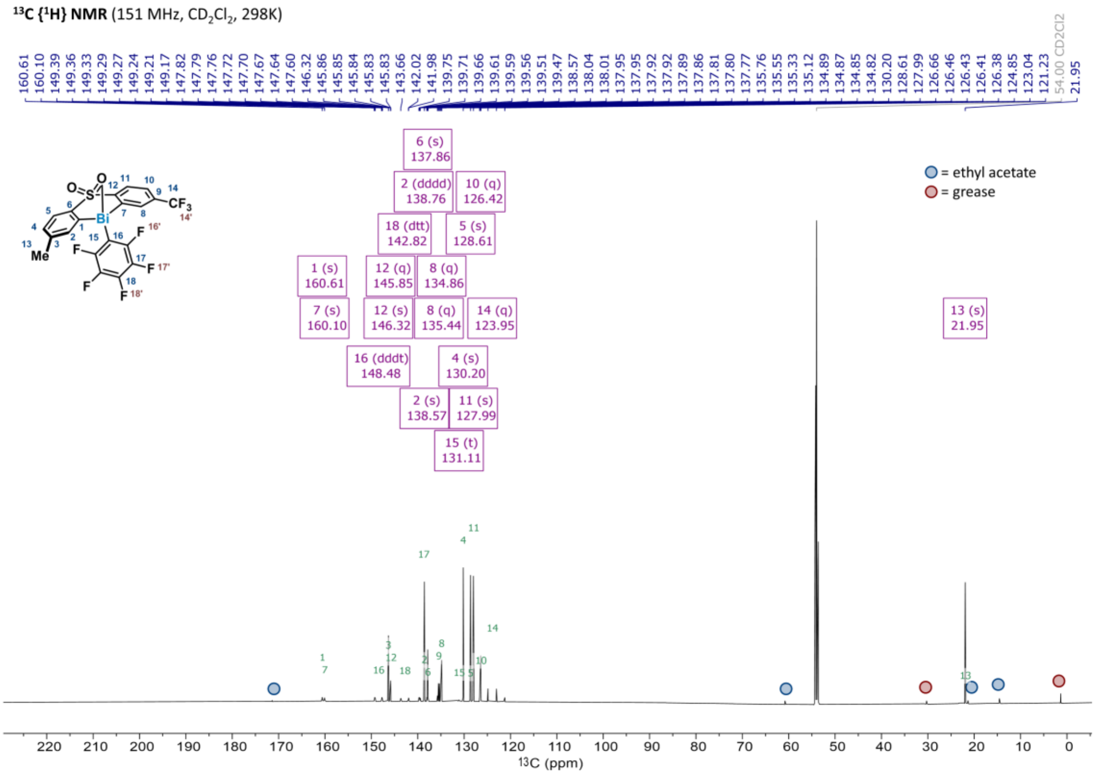

Figure S120. ^{13}C{^{1}H} NMR spectrum of **[Bi-2]•Ar^F**

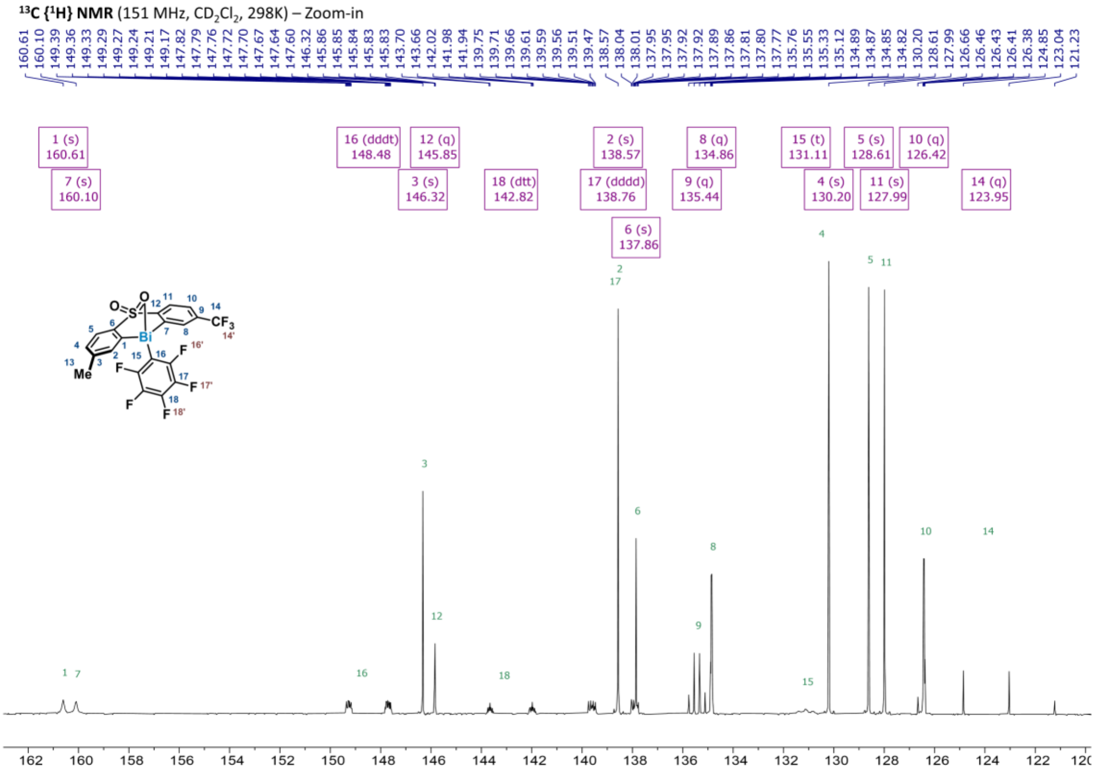

Figure S121. Zoom-in in the ^{13}C{^{1}H} NMR spectrum of **[Bi-2]•Ar^F**.

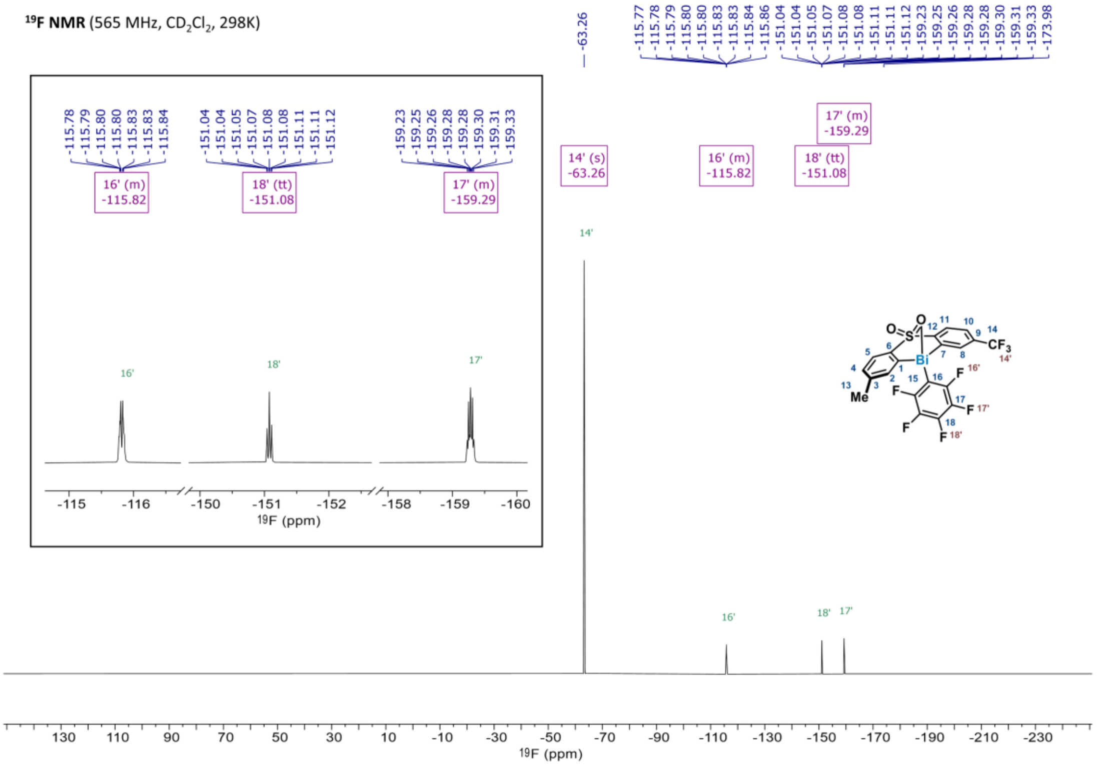

Figure S122. ^{19}F NMR spectrum of **[Bi-2]•Ar^F**.

10.11 [Bi-4]•Ar^F

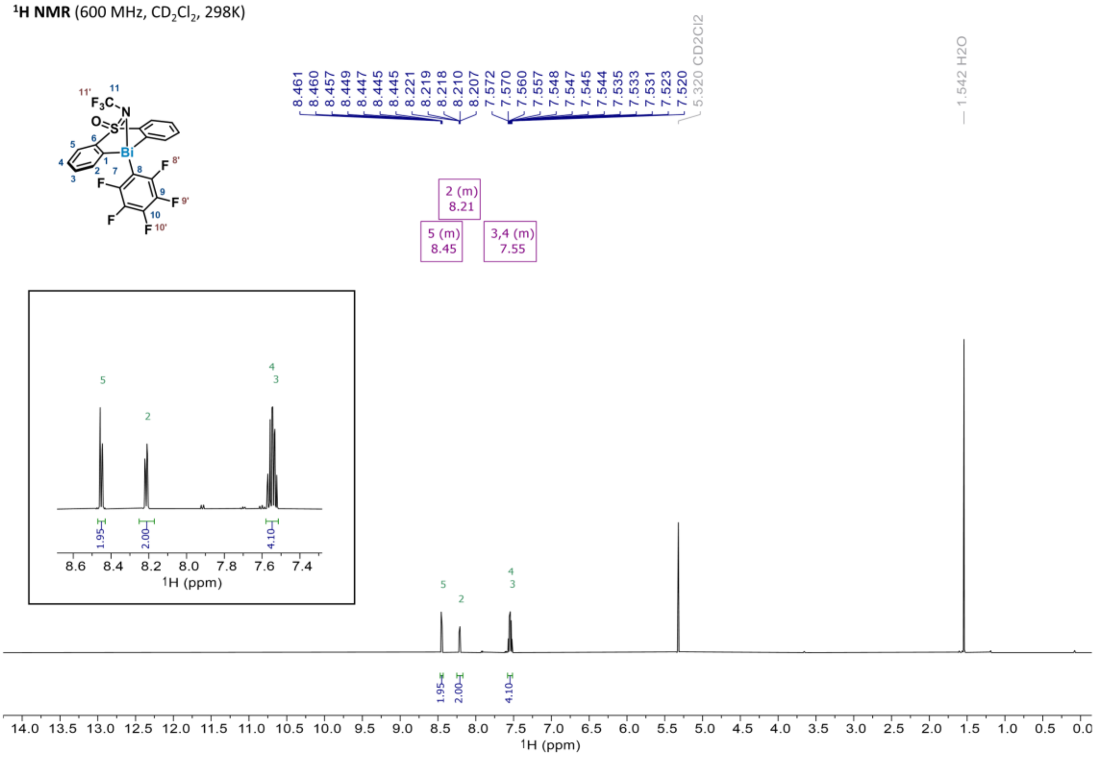

Figure S123. ^{1}H NMR spectrum of **[Bi-4]•Ar^F**.

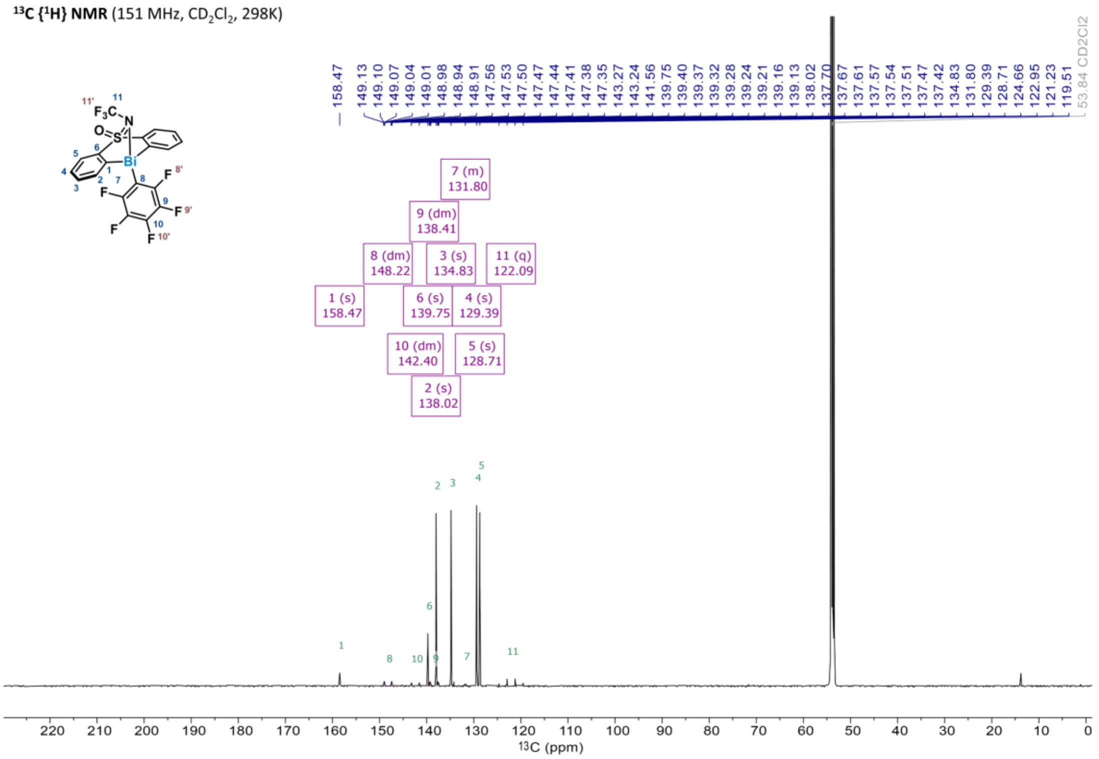

Figure S124. ^{13}C{^{1}H} NMR spectrum of **[Bi-4]•Ar^F**

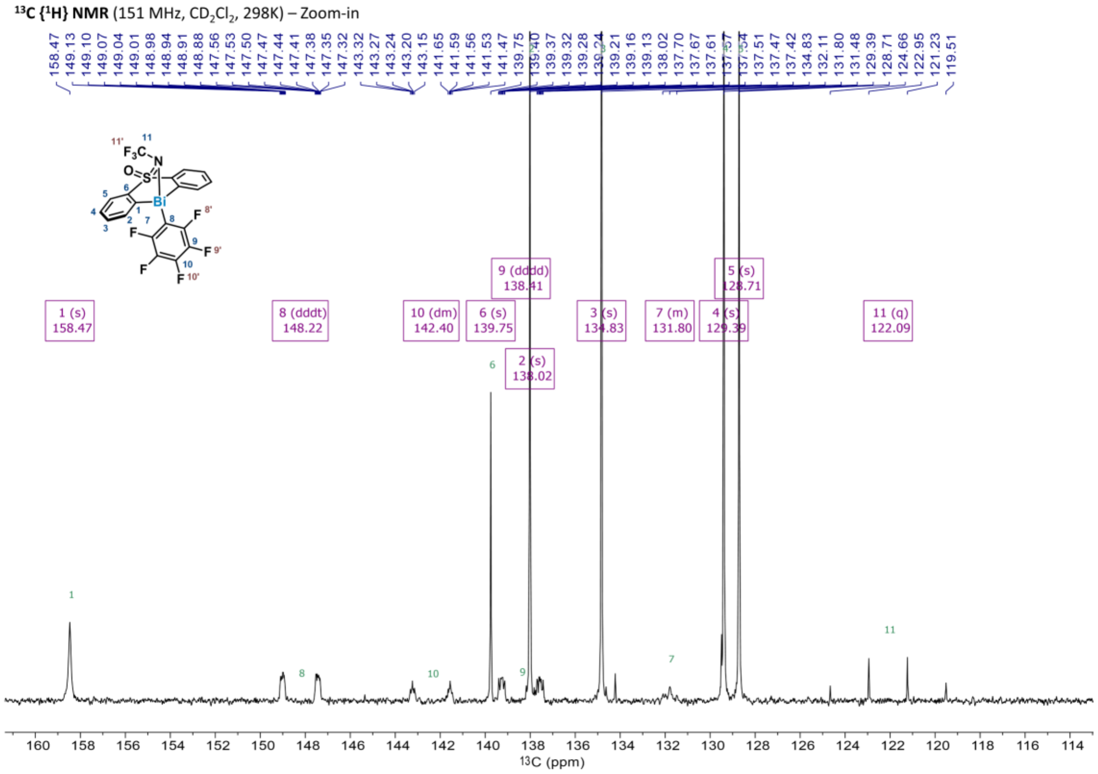

Figure S125. Zoom-in in the $^{13}C\{^{1}H\}$ NMR spectrum of **[Bi-4]•Ar^{F}**.

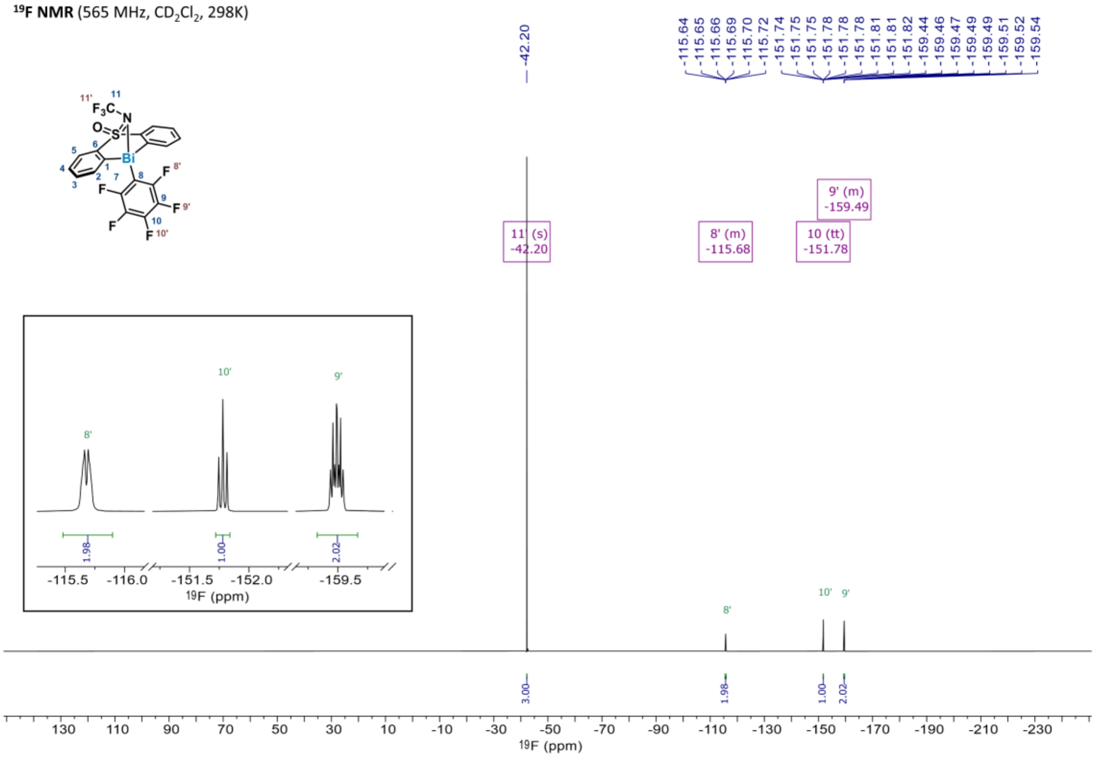

Figure S126. ^{19}F NMR spectrum of **[Bi-4]•Ar^{F}**.
